# Eschar dissolution and the immunoregulator effect of keratinase on burn wounds

**DOI:** 10.1038/s41598-023-39765-4

**Published:** 2023-08-14

**Authors:** Yan Xu, Kai Hu, Chenyang Liu, Pan Du, Feifan Zhou, Yichi Lu, Qiuyan Fu, Jianmin Xu, Guozhong Lyu

**Affiliations:** 1 Wuxi Clinical Medical College of Nanjing University of Traditional Chinese Medicine, Wuxi, 214041 China; 2 Affiliated Hospital of Jiangnan University, Wuxi, 214041 China; 3 Nanjing University of Chinese Medicine, Nanjing, 210023 China; 4 Jiangnan University, Wuxi, 214122 China; 5 Medical College of Nantong University, Nantong, 226000 China

**Keywords:** Cell polarity, Inflammation, Trauma

## Abstract

At present, enzyme debridement preparation has shown a good curative effect on eschar removal of burn wounds. Keratinase has shown great potential in enzymatic debridement because of its good fibrin-degrading ability. In this study, the debridement of keratinase was examined by using a third degree burn wound model in rats. We observed the wound, and keratinase shortened the time of eschar dissolution after debridement. Histopathology and immunofluorescence staining showed that the eschar in the keratinase group became thinner, inflammatory cell infiltration in the wound increased, the fluorescence intensity of the macrophage surface marker CD68 increased, and the CD163/CD86 ratio increased. In bone marrow-derived macrophages (BMDMs), there was no significant difference in the activity of CCK-8 in cells in the keratinase group compared with the control group. The fluorescence intensity of the keratinase group was higher than that of the control group. At 12 h, the cell scratches were obviously closed. The number of migrated Transwell cells increased. Flow cytometry and immunofluorescence analysis showed increased expression of CD206 and Arg-1 and decreased expression of CD86 and iNOS. The gene expression of the Arg-1, iNOS and IL-10 was increased, as shown by qPCR. The secretion of IL-10 was increased and TNF-α was decreased, as shown by ELISA. We concluded that keratinase dissolution of eschar not only has a hydrolytic effect on eschar but may also affect immune regulation to enhance the migration and phagocytosis of macrophages, promote the polarization of macrophages, and further enhance the effect of eschar dissolution. Therefore, keratinase may have good prospects for the debridement of burn wounds.

## Introduction

The main components of wound eschar are proteins such as collagen, fibrin, elastin, and fibronectin^1^. Eschar removal after burns can inhibit inflammation and reduce the possibility of infection to achieve faster and better treatment of burn wounds^2^. Currently, surgical debridement is still the most commonly used method of wound debridement in clinical practice, and its effect depends on the experience of the surgeon and their subjective judgment of wound contamination and necrotic tissue. Incomplete debridement is the root cause of postoperative wound infection. In contrast, thorough debridement often occurs at the expense of adjacent normal tissues, leading to large tissue defects, delayed healing and even organ dysfunction^3,4^. Enzyme debridement refers to a method that uses exogenous enzymes with proteolytic effects to decompose and remove deactivated tissues without damaging adjacent normal tissues^5^. Many enzymes have been studied, including some plant enzymes, such as bromelain and papain. Bacteria-derived enzymes such as collagenase and matrix metalloproteinases derived from *Clostridium tissoides* and *Bacillus subtilis* have also been studied. In addition, other enzymes have been reported in several animal studies, including streptokinase/chain polysaccharide enzymes, plasminase and deoxyribonuclease. When selecting enzyme debridement preparations, safety, efficacy and cost effectiveness should be considered^6^.

Bromelain and collagenase are well-studied debridement enzymes. However, there are certain risks in their use, including the possibility of partial damage to the skin around the wound, severe pain, bleeding, cellulitis and other adverse reactions^7^. Therefore, we propose to substitute an enzymatic debridement agent with simple preparation, inexpensive mass production costs, high safety and better efficacy.

Keratinase is a proteolytic enzyme that can be produced by a variety of microorganisms, including bacteria, fungi and actinomycetes^8,9^,By constructing a genetically engineered strain with heterologous expression of keratinase, the production capacity of keratinase can be effectively increased, and the goal of inexpensive mass production can be achieved^10^. Keratinases have been used on the skin. They are added to skin care cosmetics and have the effects of whitening, fickling, and inhibiting wrinkling and skin aging^11^. They also treat hyperkeratosis (such as corns, calli, and psoriasis) and acne. This indicates that keratinase is biologically safe. In addition, it has been reported that keratinases have different degradative effects on a variety of natural protein substrates, and they can dissolve collagen, which is the major component of eschar^12–14^. Therefore, it is reasonable to choose keratinase as a safe, inexpensive and effective enzymatic debridement agent. This study compared keratinase and bromelain in eschar dissolution in burn wounds and examined the mechanism of keratinase based on immune regulation of eschar dissolution.

This study investigated the potential use of keratinase in enzymatic debridement. First, the scab dissolving effects of keratinase and bromelain on burn wounds were compared, and cell activity was further characterized by CCK-8 assays. FITC-labeled glucan particles were used to characterize phagocytosis. Cell migration was evaluated by scratch assays and Transwell assays. Macrophage phenotype was characterized by flow cytometry and immunofluorescence analysis of macrophage markers. Finally, the gene and protein expression levels of related factors were measured by ELISA and qPCR to verify the results.

The role of keratinase in eschar lysis and immune regulation of burn wounds is the main innovation of this study. At the same time, we also have new thoughts on the animal model of burn and the evaluation of the degree of eschar lysis. In conclusion, we found that keratinase is promising as a new enzymatic debridement agent.

## Materials and methods

### Chemicals and reagents

Keratinase (Provided by Professor Shi Jinsong, School of Life Science and Health Engineering, Jiangnan University, School of Engineering, Jiangnan University), F12 medium, fetal bovine serum, 0.25% EDTA trypsin (Gibco, USA), streptomycin and penicillin (Thermo, USA), phosphate-buffered saline (PBS), CCK-8 kits, RNA rapid extraction kits (Yishan, China), 0.8 μm Transwell chambers, cell culture-grade DMSO, DAPI, Alexa Fluor 488 sheep anti-mouse, bromelain (Sigma, USA), FITC-dextran (MW4000) (MCE, USA), pentobarbital sodium, TNF-α and IL10 ELISA kit (Lianke Bio, China), fluorescence quantitative PCR kits, reverse transcription kits (TaKaRa, Japan), Arg-1 antibodies, iNOS antibodies, CD86 antibodies, CD206 antibodies, CD163 antibodies (Bio Legend, USA), H&E staining kits, Masson staining kits (Yeason, China), 4% paraformaldehyde, Tris–HCl, anti-fluorescence quenching sealing solution, immunohistochemical blocking solution (Beyotime, China), and primers (Sangon Biotech, China) were used.

### Keratinase activity assay

The activity of keratinase was measured with 1% soluble keratin as a substrate. The method of determination was performed as described by Yamamura et al.^15^ with some changes. The specific method was as follows: 0.5 ml of substrate was added to 0.5 ml of suitably diluted Tris–HCl (pH 9.0) and incubated at 50 °C for 20 min in a water bath. After that, 1 ml of 4% TCA solution was added to terminate the reaction, and the sample was centrifuged at 12,000 rpm for 5 min. Then, 5 ml of 0.4 M Na_2_CO_3_ and 1 ml of folinphenol reagent were added to 1 ml of the supernatant after centrifugation. The mixture was heated in a water bath at 40 °C for 20 min, and the color was developed. In the control group, the enzyme solution was mixed with the TCA solution, and then the substrate was added after incubation in a water bath at 50 °C for 20 min. The other procedures were the same as those in the experimental group. To determine enzyme activity, the enzyme solution hydrolyzed the substrate such that each 0.01 increase in the absorbance value at 660 nm was one unit of enzyme activity (U ml^−1^). The activity of keratinase was measured in a pH 7 buffer system at 37 °C for different times (0 h, 0.5 h, 1 h, 2 h, 4 h, 8 h, 12 h). All experiments measured data from three parallel reactions, and each treatment used three replicates.

### In vivo experiments

Twenty 6-week-old male Sprague‒Dawley rats were used. The back hair was removed with a shaver and then completely removed with depilatory cream the day before the experiment. On the same day as the experiment, a hollow EP tube with a diameter of 5 cm was placed against the back skin of each rat, and 10 ml of 98 °C water was quickly poured into the tube. After 30 s, the hot water was withdrawn with a syringe. Saline was used to clean the wounds, and each wound was covered with 3 cm^2^ of double-layer gauze and divided into one of three groups. (1) Saline (1 ml) was added to the double-layer gauze. (2) Bromelain enzyme preparations (20 U/1 ml) were added to the double-layer gauze. (3) Keratinase enzyme preparations (20 U/1 ml) were added to the double-layer gauze. The wound was first covered with gauze with enzyme or normal saline. In order to maintain enzyme activity and moisten the wound, Vaseline gauze was covered on the gauze. The activity of keratinase remained approximately 20% after 12 h. Therefore, in order to maintain a high level of enzyme activity on the wound, dressing changes were selected every 12 h until the wound eschar dissolved (approximately 7–14 days). At 1, 3, 5, 7, and 10 days, the dissolution of wound eschar was observed. The was animals were sacrificed by the spinal dislocation method, and complete wound and tissue samples 1 mm around the wound were collected for H&E staining, Masson's trichrome staining, and immunofluorescence staining.

#### Histopathological examination

The wound samples were taken 1, 3, and 7 days after modeling, fixed in 4% formaldehyde, and embedded in paraffin in PBS buffer (pH 7.2). Tissue sections (5 microns thick) were stained with H&E and modified Masson's trichrome according to the instructions of the kit^16^.

#### Tissue immunofluorescence analysis

The detailed steps for immunofluorescence analysis were as follows: Day 3 tissue sections were deparaffinized and hydrated. The samples were then immersed in sodium citrate antigen repair solution at 100 °C for 25 min for antigen repair. The samples were blocked with immunohistochemical blocking solution for 60 min at room temperature. CD68, CD163, and CD86 primary antibodies were prepared according to the manufacturer's instructions and incubated with the samples overnight at 4 °C. The next day, 3 washed with PBST for 5 min each time were performed, and the secondary antibodies were added and incubated for 90 min at room temperature. The samples were counterstained with DAPI. Images were taken with an orthostatic fluorescence microscope and analyzed with ImageJ.

### In vitro experiments

#### Cell culture

Bone marrow-derived macrophages (BMDMs) were isolated and cultured^17^. Mouse bone marrow cells were isolated from the tibia and femur and washed with DMEM/F12. Cultures were grown in DMEM/F12 containing 10% fetal bovine serum and 10 ng/ml macrophage colony stimulating factor at 37 °C in a constant temperature incubator with 5% carbon dioxide. Before further experiments, the macrophage marker F4/80 was identified by flow cytometry.

#### Cell viability assay

Cell activity was measured using a CCK-8 kit according to the manufacturer's instructions. In brief, extracted MØ cells (1 × 10^4^ cells/well) were seeded in 96-well plates, placed in the incubator for 24 h, and then incubated with 20 U/ml of enzymes for 4 h. CCK-8 reagent was added to the cells in the dark and incubated for 1 h. The Spectra Max microplate reader (Thermo Science, Hudson, NH, USA) was used to measure the absorbance at 450 nm.

#### Cell phagocytosis assay

The extracted MØ cells (1 × 10^4^ cells/well) were seeded on 96-well plates, placed in the incubator for 24 h, and then incubated with 20 U/ml enzymes for 4 h. After being washed three times with PBS, FITC-labeled dextran fluorescent particles were added and incubated in the dark for 1 h. Following three washes with PBS, the plates were fixed with 4.0% (w/v) paraformaldehyde and imaged with a Leica inverted fluorescence microscope, and the mean fluorescence intensity was analyzed by ImageJ.

Cell migration was assessed by a scratch assay. MØ macrophages (2 × 10^6^/well) were cultured in 6-well plates, and after 24 h, cells in the center of the well were scraped with a sterile 200 μl pipette tip to create a cell-free area, and the scratched areas were photographed with an inverted microscope after 6 and 12 h of incubation with 20 U/ml enzymes. The scratch area was measured using ImageJ software^18^. For the Transwell migration assay, MØ cells were seeded in the upper chamber of a 24-well Transwell plate with a diameter of 0.8 μm at a density of 3 × 10^4^/well, and then DMEM and 20 U/ml enzymes were added to the lower chamber. After 12 h of culture, the upper chamber was removed, and the cells on surface of the upper chamber were wiped off. The cells on the outer surface were stained with 0.1% crystal violet for 15 min, washed three times with water, observed under an optical microscope and photographed.

#### Flow cytometry

We used flow cytometry and single staining to identify M1/M2 macrophages. First, MØ cells were incubated with DMEM and 20 U/ml enzyme for 4 h, and then the cells in each group were collected, washed twice with precooled PBS, and resuspended. The cell density was adjusted to 1 × 10^6^/ml. The cell suspensions were place in 1.5 ml EP tubes at a density of 100 µl/tube, and CD206-PE and CD86-PE antibodies were added and incubated for 40 min at 4 °C. After centrifugation at 400×*g* for 3 min, the supernatant was discarded, the cells were washed twice with PBS and resuspended in 100 μl of PBS, the positive expression rate of the antibody was detected. FlowJo software was used to analyze the results.

#### Cell immunofluorescence analysis

MØ cells (2 × 10^5^/well) were cultured in 12-well plates with cell slides for 24 h, incubated with 20 U/ml enzyme for 4 h, washed 3 times with PBST, permeabilized with 0.5% Triton X-100 (Sigma) for 1 h, blocked for 1 h at room temperature, and incubated with primary antibody overnight. The cells were then incubated with secondary antibodies for 2 h at room temperature. The samples were then treated with DAPI for 5 min to counterstain nuclei. For quantitative analysis, images were obtained with a Leica inverted fluorescence microscope, three photographs were taken in random fields, and the mean fluorescence intensity was analyzed using ImageJ.

#### RNA extraction and quantitative real‑time PCR (qPCR)

Total RNA was extracted from MØ cells using the RNA Rapid Extraction Kit. The concentration of RNA was measured using a NanoDrop system from Thermo Fisher Scientific (USA). Then, cDNA was amplified from 2 μg of total RNA using a fluorescent quantitative PCR kit, and qPCR was performed on a Light Cycler 480II (Roche, Rheinland) using SYBR green. GAPDH was used as an internal reference. The relative expression of each gene to the GAPDH value was calculated using the 2-ΔΔCT formula^19^. The primer sequences are listed in Table 1.

**Table 1:** Primers used for qPCR.

Gene	Sequences (5′–3′)	Product size (bp)
iNOS	Forward: ATCTTGGAGCGAGTTGTGGATTGTC	146
	Reverse: TAGGTGAGGGCTTGGCTGAGTG	
Arg-1	Forward: AGACAGCAGAGGAGGTGAAGAGTAC	118
	Reverse: AAGGTAGTCAGTCCCTGGCTTATGG	
IL-10	Forward: AGAGAAGCATGGCCCAGAAATCAAG	136
	Reverse: CTTCACCTGCTCCACTGCCTTG	
TNF-α	Forward: CACTACAGGCTCCGAGATGAACAAC	145
	Reverse: TGTCGTTGCTTGGTTCTCCTTGTAC	
GAPDH	Forward: TGACATCAAGAAGGTGGTGAAGCAG	224
	Reverse: GTGTCGCTGTTGAAGTCAGAGGAG	

#### ELISA

After the successful induction of macrophages, the cells were incubated with 20 U/ml enzyme and DMEM for 4 h. The old medium was discarded, the cells were washed three times with PBS, and DMEM was added and incubated for 12 h. The supernatant of each group of macrophages was collected and centrifuged at 350*g* for 5 min to remove cell debris. The samples were stored at − 80 °C, and ELISA was performed according to the instructions of the kit.

#### Statistical analysis

All results are expressed as the mean ± SEM and were analyzed using GraphPad Version 6 (GraphPad Prism Software, Inc., USA). Statistical significance was determined using a paired t test or one-way analysis of variance (ANOVA). P < 0.05 was considered significant. Each experiment was performed in triplicate and repeated at least three times.

### Ethics statement

All methods have been reported in accordance with the ARRIVE guidelines (https://arriveguidelines.org) and the American Veterinary Medical Association (AVMA) Guidelines for the Euthanasia of Animals (2020). All experimental animal procedures were approved by the Animal Ethics Committee of Jiangnan University (Animal Ethics Approval number: JN. No. 20220915Mø401230[354]). All methods were carried out in accordance with relevant guidelines and regulations.

## Results

### Enzyme activity of keratinase

**Figure 1:**
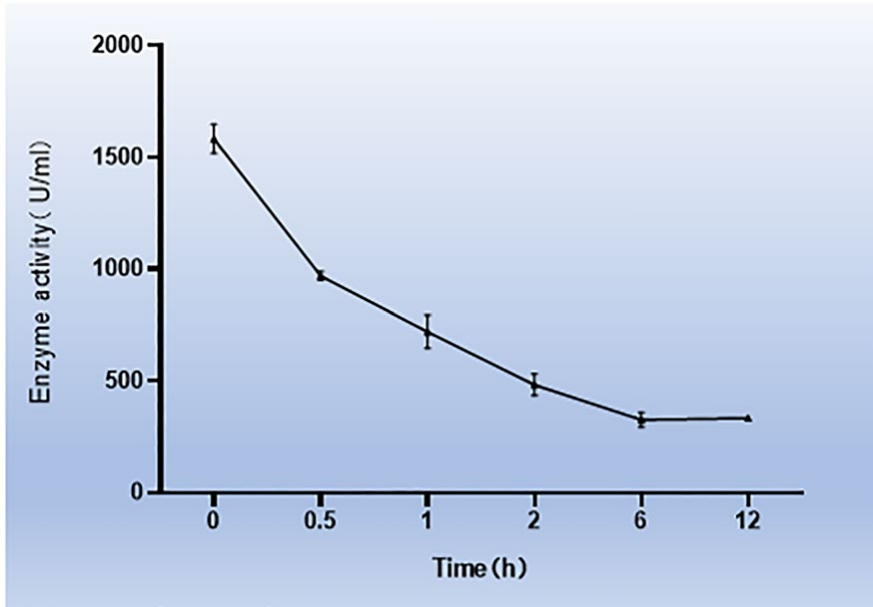
Enzymatic activity of keratinase. Enzymatic activity of keratinase at different times. The data represent the mean ± SEM (N = 3, *P < 0.05, **P < 0.01, ***P < 0.001). Images were processed with GraphPad Prism Version 9.0 software.

First, the enzyme activity of keratinase at 37 °C was determined by the folin-phenol method. The results showed that the activity of keratinase decreased with time at 37 °C, and the activity of keratinase remained approximately 20% after 12 h. (Fig. 1A).

### In vivo experiments

#### Keratinase can promote the dissolution of eschar

**Figure 2:**
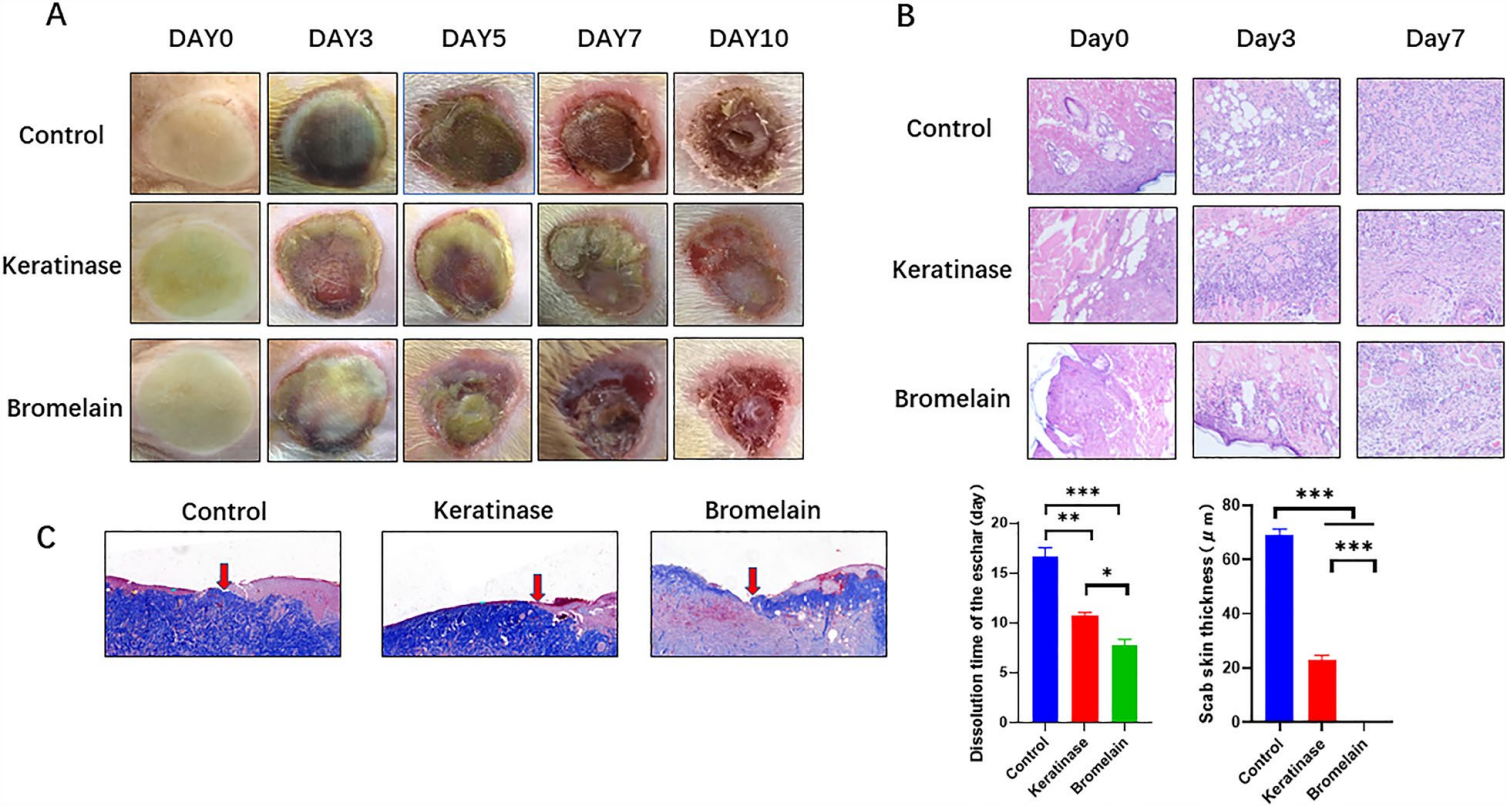
The burn wounds of the rats on days 0, 3, and 7 were removed, fixed, dehydrated, embedded in sections and subjected to relevant pathological examinations. (**A**) Wound eschar dissolution was observed on days 0, 3, 5, 7, and 10. The time to complete dissolution of the eschar in each group was determined. (**B**) H&E staining of burn wounds on days 0, 3, and 7. (**C**) Masson staining of burn wounds on day 7. The remaining eschar thickness was also measured. The data represent the mean ± SEM (N = 3, *P < 0.05, **P < 0.01, ***P < 0.001). Images were processed with GraphPad Prism Version 9.0 and Photoshop Version 7.0 software.

After observing the wound eschar lysis time of rats, it was found that the average time of eschar removal in the control group was 17.67 days, and the average times of eschar removal in the keratin and bromelain groups were 10.67 and 7.67 days, respectively. The time of eschar removal after enzyme debridement was significantly reduced compared with that in the control group. Figure 2A shows the eschar dissolution in wounds in the different groups. On day 0, the skin of the wound was pale and slightly swollen after modeling, and the boundary of the scalded area was clear. The scalded wound could be clearly distinguished from normal skin by the naked eye. On day 3 in the control group, the wound color was deepened with large, purplish red blood ecchymosis, and the wound was harder than normal skin. In the keratinase group, the eschar margin was raised, the center of the wound showed small flaky eschar that became thin and sunken, and the tissue was slightly soft. In the bromelain group, some tissues at the wound edge were loose and edematous, the wound surface was soft, and there was a small amount of blood ecchymosis on the wound edge. On day 7, the control group had warped edges, and the wound edges were trimmed during the dressing change. Part of the eschar in the keratinase group was dissolved, and the granulation of the new base was fresh. In the bromelain group, most of the eschar dissolved, and wound granulation edema was obvious. On day 10 in the control group, the wound edge was slightly warped and pruned, and the eschar at the center of the wound edge was hard. The crusts in the keratinase group were basically dissolved, and the granulation was fresh. In the bromelain group, the eschar was completely dissolved, granulation edema was reduced, and bleeding was reduced. Therefore, keratinase can promote the dissolution of burn wound eschar, with no obvious bleeding or infection (Fig. 2A).

H&E staining was used to analyze the wound tissue on days 0, 3 and 7. Before the first day of administration, epidermal layer cells and hair follicle epithelial nuclei were coyoted, deep dermal tissue was damaged, collagen fibers were swollen and fused, and a large number of subcutaneous fat cells were fused. Vascular dilatation and congestion were observed throughout the dermis; sebaceous glands, sweat glands and hair follicles were destroyed; and the structure was not clear. Third degree burns indicated that the model was successful (Fig. 2B).

Then, we stained sections with Masson’s trichrome on the 7th day, and we found that the epidermal layer in the experimental group was shed, the thickness of the eschar gradually became thinner after enzymatic debridement, and granulation continuously grew in the basal and subcutaneous parts of the dermis. We measured the eschar thickness to determine the debridement effect, and the results showed that the thickness in the keratinase group was lower than that in the bromelain group and the control group (Fig. 2C).

#### Keratinase can promote the recruitment of macrophages in the wound in the early stage and promote their polarization to the M2 phenotype

On the third day, H&E staining showed that the thrombus was structured and separated by a large number of proliferating vascular endothelial cells and their cords, and a local lumen with a dilated capillary network formed, with a small increase in nearby fibroblasts. In addition, a large number of mononuclear/macrophages, neutrophils and lymphocytes had infiltrated. The experimental group had more inflammatory cell infiltration than the control group. By analyzing the results of H&E staining on the 7th day, we found more inflammatory cell infiltration in the control group, while the inflammatory cells in the keratinase and bromelain groups were reduced and accompanied by a large number of fibroblasts and vascular proliferation.

**Figure 3:**
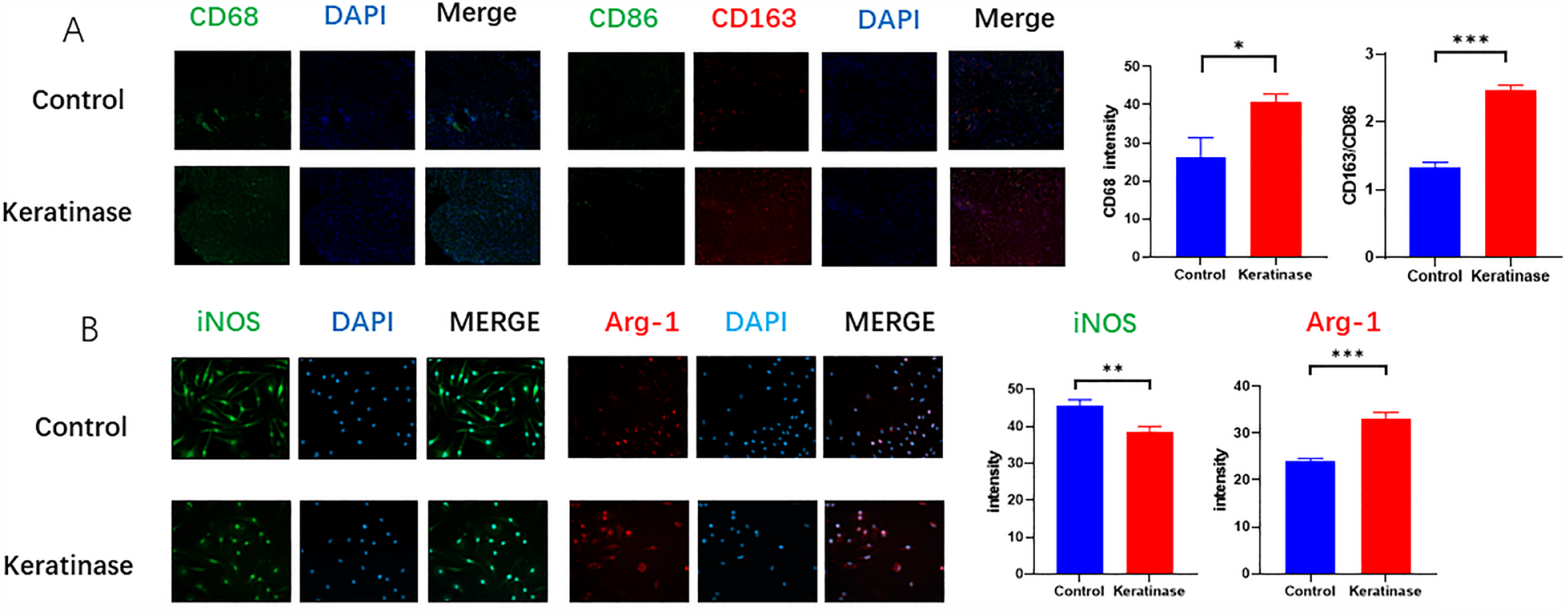
Effect of keratinase on the polarization of BMDMs and wound macrophages. (**A**) Immunofluorescence was used to determine the fluorescence intensity of Arg-1 and iNOS in BMDMs. (**B**) Immunofluorescence staining was performed to observe macrophage-related markers in the wound tissue on day 7. The data represent the mean ± SEM (N = 3, *P < 0.05, **P < 0.01, ***P < 0.001).Images were processed with GraphPad Prism Version 9.0 and Photoshop Version 7.0 software.

The immunofluorescence results showed that macrophages in the wound tissue were labeled with CD68. The fluorescence intensity in the keratinase group was 40.88 ± 1.98 and that in the control group was 26.03 ± 5.29. The keratinase group was higher than the control group, and the difference was statistically significant. These results indicated that keratinase promoted the recruitment of macrophages in the early stage of the wound (Fig. 3A).

On the 7th day, the sections were stained with immunofluorescence, and M1 and M2 macrophages in the wound tissue were labeled with CD163 and CD86, respectively. By calculating the fluorescence intensity ratio of CD163/CD86, the proportions of M2 macrophages in the keratinase group and the control group were found to be 2.47 ± 0.07 and 1.33 ± 0.08, respectively. The difference was statistically significant. These results suggest that keratinase can promote the M2 polarization of macrophages in wounds after promoting the early recruitment of macrophages (Fig. 3A).

### In vitro experiments

#### Keratinase can enhance macrophage migration and phagocytosis

Macrophages dominate inflammation in burn wounds. We further examined the influence of enzymes on the main biological functions of macrophages, including recruitment, migration, phagocytosis, and polarization, and the mechanism by which enzymes regulate subsequent inflammation of wounds.

**Figure 4:**
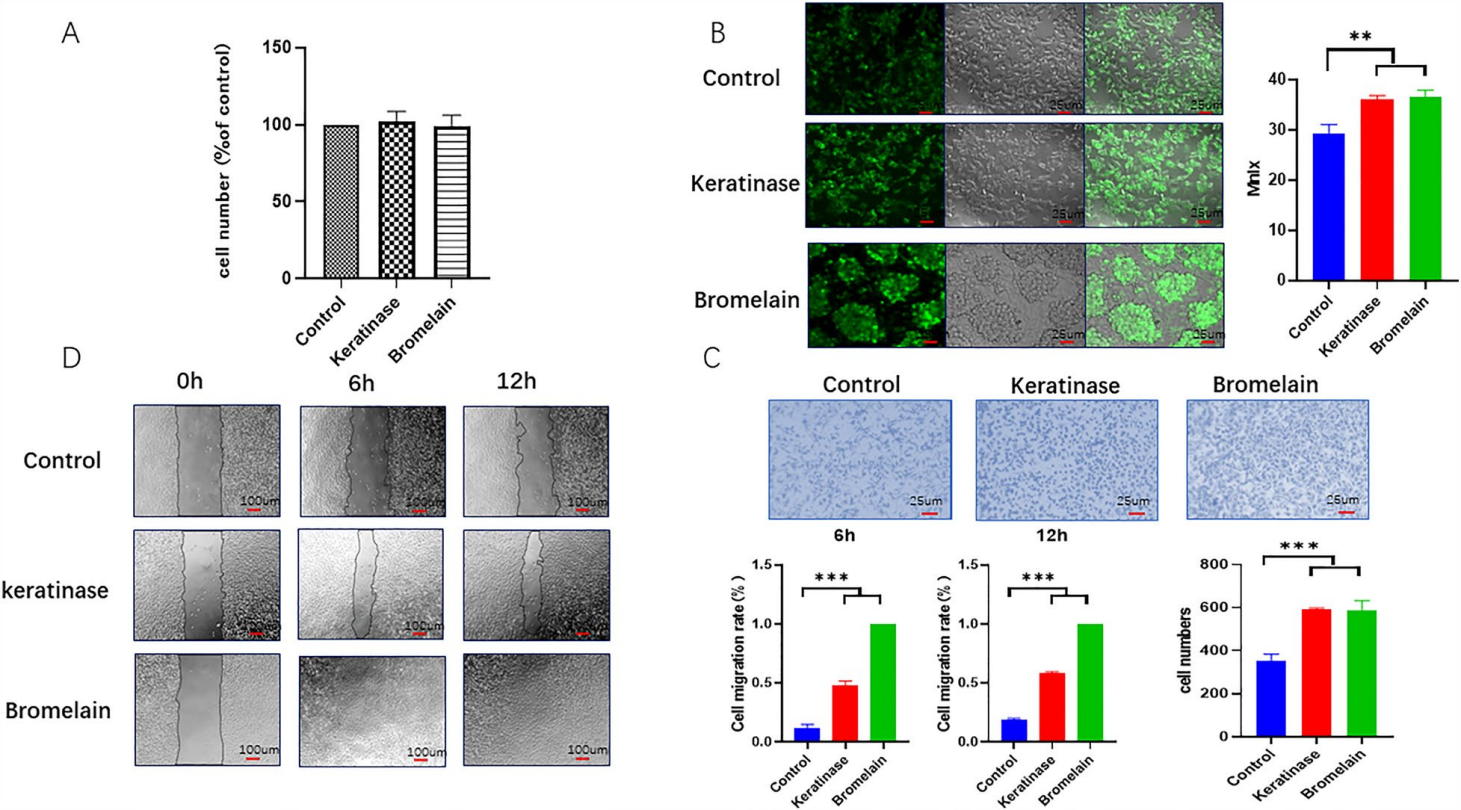
Enzymatic debridement agents regulate the proliferation, migration and phagocytic activities of BMDMs. (**A**) Effects of an enzymatic debridement agent on the proliferation of BMDMs. (**B**) Effect of an enzymatic debridement agent on the phagocytosis of BMDMs. (**C**) Effect of an enzymatic debridement agent on the migration of BMDMs, as determined by a cell wound scratch assay. (**D**) Effect of an enzymatic debridement agent on the migration of BMDMs according to the Transwell assay. The data represent the mean ± SEM (N = 3, *P < 0.05, **P < 0.01, ***P < 0.001). Images were processed with GraphPad Prism Version 9.0 and Photoshop Version 7.0 software.

In vitro, we extracted BMDMs and identified them by the flow cytometry and marker F4/80, and the positive rate was 95%. The cytotoxicity of the enzymes on MØ macrophages was detected by CCK-8 assays. Compared with that in the control group, there was no significant difference in cell activity between the keratinase group and the bromelain group, indicating that neither keratinase nor bromelain was toxic to macrophages (Fig. 4A). The effect of the enzyme on the phagocytic activity of macrophages was detected by a FITC-labeled dextran particle phagocytosis assay. The results showed that the phagocytic activity of macrophages was significantly increased after treatment with the two enzymes, and the difference was statistically significant (Fig. 4B). Finally, the effect of the enzymes on the migration of macrophages was detected by the Transwell method. The results showed that the number of macrophages in the experimental group was significantly higher than that in the control group, and the difference was statistically significant (Fig. 4C). We also calculated the migration area by the cell scratch method, and the results were consistent with the Transwell assay; the difference was statistically significant (Fig. 4D). These results showed that the biological functions of macrophages were enhanced by enzyme treatment, and the effects of keratinase and bromelain were similar.

#### Keratinase can promote macrophage polarization and increase the gene and protein expression of related factors

**Figure 5:**
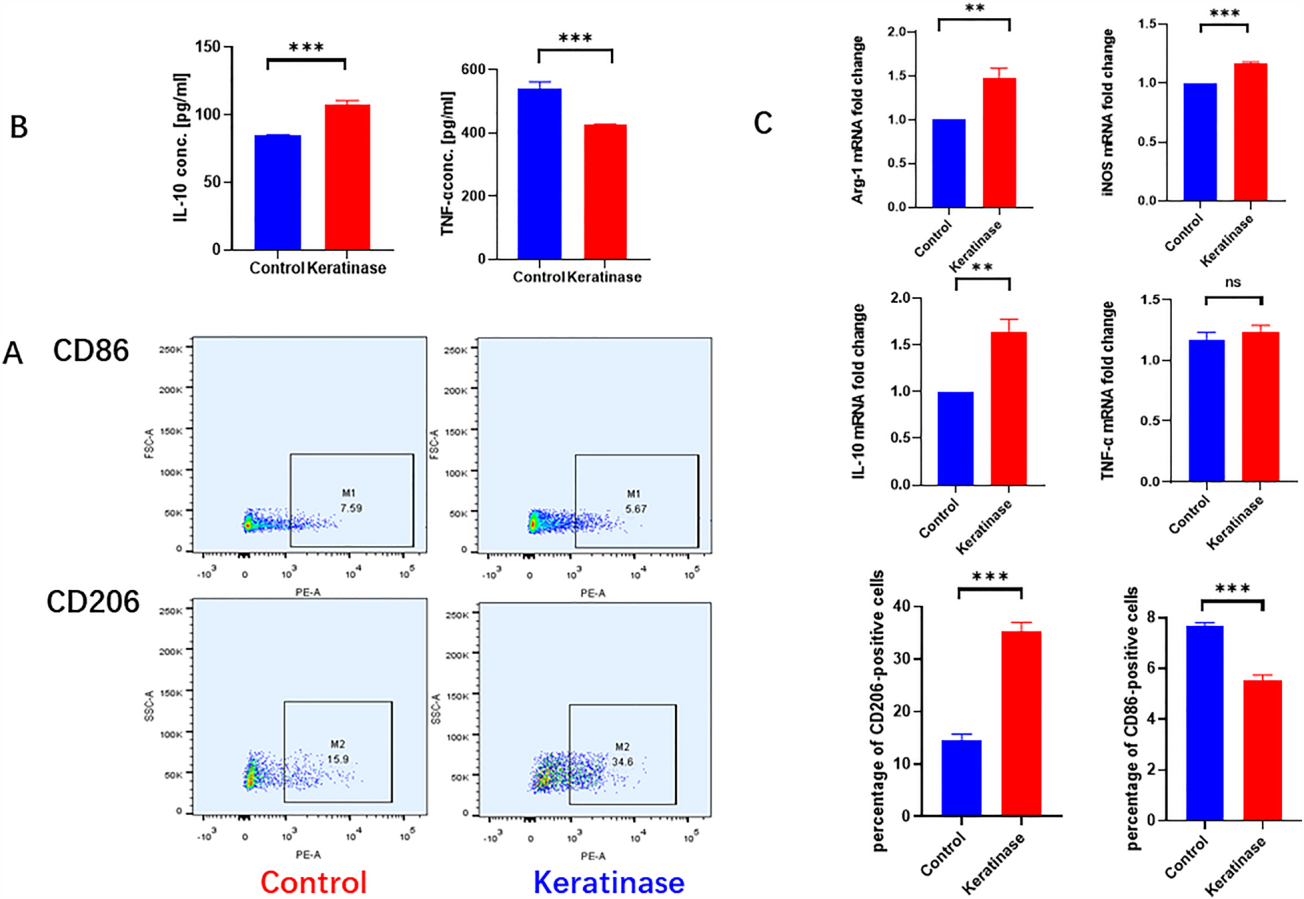
Effect of keratinase on the polarization of BMDMs. (**A**) Secreted cytokines were detected by ELISA. (**B**) qPCR was used to detect the expression of related genes. (**C**) Macrophage-specific markers were detected by flow cytometry. The data represent the mean ± SEM (N = 3, *P < 0.05, **P < 0.01, ***P < 0.001). Images were processed with GraphPad Prism Version 9.0 and Photoshop Version 7.0 software.

Flow cytometry and immunofluorescence were used to identify the phenotype of macrophages. Arg-1 and CD206 were used in this study as markers of M2 macrophages, and iNOS and CD86 were used as markers of M1 macrophages. The flow cytometry results showed that the positive number of M2 macrophages in the keratinase group was significantly higher than that in the control group, and the positive number of M1 macrophages was lower than that in the control group. The differences were all statistically significant (Fig. 5A). The immunofluorescence results showed that the intensity of Arg-1 in the keratinase group was significantly higher than that in the control group, and the intensity of iNOS was lower than that in the control group. The difference was statistically significant (Fig. 3B). These results supported the in vivo results.

Next, we measured the cytokines secreted by macrophages. TNF-α and IL10 are the hallmark cytokines of M1 and M2 macrophages, respectively. The results showed that keratinase significantly reduced the secretion of TNF-α and increased the secretion of IL-10 (Fig. 5B). Finally, we determined the gene expression of MØ macrophages. The results showed that the gene expression of Arg-1, iNOS and IL-10 was significantly increased in the keratinase group, and the difference was statistically significant, while the gene expression of TNF-α was not significantly changed (Fig. 5C).

## Discussion

At present, it is believed that necrotic tissue after burn injury is the root cause of immune disorders. Due to the abnormal immune response, the local inflammatory response in the wound and the dissemination of inflammatory factors secreted by neutrophils and mononuclear phagocytes after burn induce a systemic inflammatory response. Early removal of burn eschar is the key to improving the wound healing rate. At present, there are many methods for the removal of burn eschar, such as eschar excision, tangential excision, MEBT/MEBO liquefaction, and enzyme preparation debridement^20^. It is worth noting that enzyme preparations may also play a role in regulating the function of immune cells in wounds while removing the eschar of wounds. Engwerda^21^ et al. found that bromelain could regulate the innate immune response and that bromelain could enhance IFN-g-mediated production of nitric oxide (NO) and TNF-α by macrophages. On the other hand, Amitava Das et al.^22^ found that Clostridium collagenase could induce the polarization of M2 macrophages to regulate the wound immune response and promote wound healing. The mechanism may be related to collagen peptides, which are the degradation products of Clostridium collagenase. In conclusion, enzymatic debridement agents not only degrade eschar on wounds but also regulate the immune response in wounds through degradation products or themselves.

Macrophages have been shown to play critical regulatory roles at all stages of wound inflammation, repair, and fibrosis, and they can secrete relevant chemokines, matrix metalloproteinases, and other inflammatory mediators that drive the initial cellular response after injury^23,24^. Macrophages exhibit remarkable phenotypic diversity, and their secretory profiles and functional properties are influenced by microenvironmental factors. Two major macrophage populations are classically activated (M1) and alternatively activated (M2) macrophages, with the former producing proinflammatory mediators and the latter exhibiting immunomodulatory properties^25,26^. M1 macrophages exhibit enhanced antimicrobial properties, while M2 macrophages have enhanced phagocytosis and play a key role in removing cell debris, apoptotic cells and necrotic tissue, thereby allowing tissue repair and regeneration^27^. The large amounts of IL-10 and TGF-β secreted by M2 macrophages exert strong anti-inflammatory effects against apoptotic cells and promote tissue repair, wound healing, angiogenesis and fibrosis^1,28^.

Keratinase is a biological enzyme with a wide range of sources that can degrade the main components of eschar, such as collagen, fibrin, elastin, fibronectin, and hemoglobin, as substrates. The heterologous expression and gene mining technology of keratinase can make it a safe, robust, economical and environmentally friendly enzyme debridement agent. A new keratinase was produced by using a transgenic strain. The activity of keratinase in the human body was measured at 37 °C. Keratinase activity was high and could be maintained for a long time at 37 °C.

Therefore, we used keratinase, bromelain and control groups to compare the effect on eschar lysis in scalding wounds. The results showed that keratinase had an obvious debridement effect and biological safety.

We conducted experiments to compare the dissolution time and thickness of the eschar and found that the dissolution time after keratinase treatment was shorter than that in the saline group and was slightly longer than that in the bromelain group. However, in the current study, some wounds in the bromelain group oozed significantly. Masson staining showed that after enzymatic debridement, the epidermis fell off, the eschar gradually became thinner, and granulation continued to grow at the base of the dermis and under the skin. The effect of debridement was determined by measuring eschar thickness, which was significantly smaller in the keratinase group than in the control group. In addition, H&E staining showed that the infiltration of inflammatory cells in the wound was significantly increased at the early stage after enzymatic debridement. Tissue immunofluorescence showed that CD68 was highly expressed in macrophages in the wound after enzymatic debridement, which also supported the results of H&E staining. We further hypothesized that keratinase could enhance wound immunity by promoting the recruitment of macrophages and enhancing the functional properties of macrophages.

Thus, we performed a CCK-8 assay to examine the effect of the enzyme on the activity of macrophages, and the results showed that keratinase and bromelain had no obvious cytotoxicity. We also performed Transwell and cell wound scratch assays, and the results showed that enzyme treatment could promote the migration of macrophages. In the scratch assay, macrophages in the keratinase group were close to closure after 12 h, and macrophages in the bromelain group were completely closed. Finally, we used FITC-dextran to examine the phagocytic activity of macrophages. The results showed that the phagocytic activity of macrophages in the keratinase treatment groups was enhanced. Under a microscope, the morphological changes of macrophages in the keratinase treatment group were observed, and pseudopodia were obviously formed. Macrophages in the bromelain group gathered in sheets and changed in morphology. We hypothesized that keratinase could not only enhance the migration, recruitment, and phagocytosis of macrophages in the wound but also polarize macrophages into a more highly phagocytic M2 type, thereby eliminating necrotic cells.

Further experiments were performed to identify macrophage phenotype. An increase in the proportion of M2 macrophages in the keratinase group was observed in both cell and tissue immunofluorescence assays, which was consistent with the flow cytometry results. Finally, we determined the gene and protein expression levels of related factors by ELISA and qPCR. The results showed that the gene expression of IL-10 and Arg-1 was increased, the secretion level of IL10 was increased, and the secretion level of TNF-α was decreased in the keratinase group. These results suggest that keratinase can promote the polarization of macrophages to the M2 phenotype. This polarization enhances the phagocytic ability of wound macrophages and accelerates the phagocytosis and decomposition of necrotic tissue and apoptotic cells in wounds. Furthermore, the cytokines released by keratinase can also regulate inflammation, providing a good microenvironment for wound healing.

This study is somewhat innovative. First, we used keratinase, which has not been used in the field of enzymatic debridement, as the research object, and investigated its effect on eschar lysis. It was found that keratinase is a promising enzyme preparation.

Second, we considered the choice of animal model. At present, mice, rats, pigs, and so on have been used as burn models in clinical practice. The skin of mice has epidermis and dermis, but it is thinner than that of humans, and the body size of mice is small, which leads to the establishment of III degree burn model and easy death. Pig is an ideal burn model animal, and previous studies have used porcine skin burn model as the subject of enzymatic debridement preparation^29^. However, the high cost and operation complexity of pigs as animal models limit the use of pigs in animal experiments.

In this study, we used rats as a III degree scald model animal. Rats share many physiological and pathological features similar to humans. Most importantly, as a "skin laxative animal", the rat's skin is elastic and does not have a strong attachment to the subcutaneous structures, which makes the rat burn model even in burn depth when injured as a hydrothermal solution/steam^30^. Moreover, rats are larger than mice and less likely to die from trauma. Therefore, we selected rats as the animal experimental model based on the comprehensive selection of various aspects.

We found that in the process of eschar dissolution on rat burn wounds, the eschar dissolution was irregular, and the degree of dissolution was difficult to quantify. Therefore, the dissolution effect of the eschar was evaluated by the time of complete dissolution and the thickness of the eschar was measured by Masson staining of pathological sections, as shown in the figure.

We also explored the possible immunomodulatory properties of keratinases. The regulatory effect of keratinase on macrophages was verified by in vitro and in vivo experiments. Previous studies have shown that both collagenase and bromelain have certain effects on the physiological functions of macrophages^21,31^. In our study, we also found that the effect of keratinase on eschar dissolution may not only be substrate dissolution, but also promote macrophages and regulate wound immune function.

## Conclusion

We found that keratinase could be used as a new enzymatic debridement agent, which has the same effect as bromelain and is safer. Moreover, we found that the debridement effect of keratinase was not only related to its own degradation of the eschar but also may be due to its ability to enhance the function of macrophages in the wound, polarizing macrophages into more phagocytic M2 cells. However, the mechanism of macrophage polarization has not been fully elucidated and needs further exploration.

### Supplementary Information

Supplementary Information.

## Data Availability

The datasets used and/or analysed during the current study available from the corresponding author on reasonable request.
