# Supplementary figures and images for: Eschar dissolution and the immunoregulator effect of keratinase on burn wounds (part 2 of 2)
Source: Sci Rep. 2023 Aug 14;13:13238. doi: 10.1038/s41598-023-39765-4 (PMC10425458; doi:10.1038/s41598-023-39765-4)

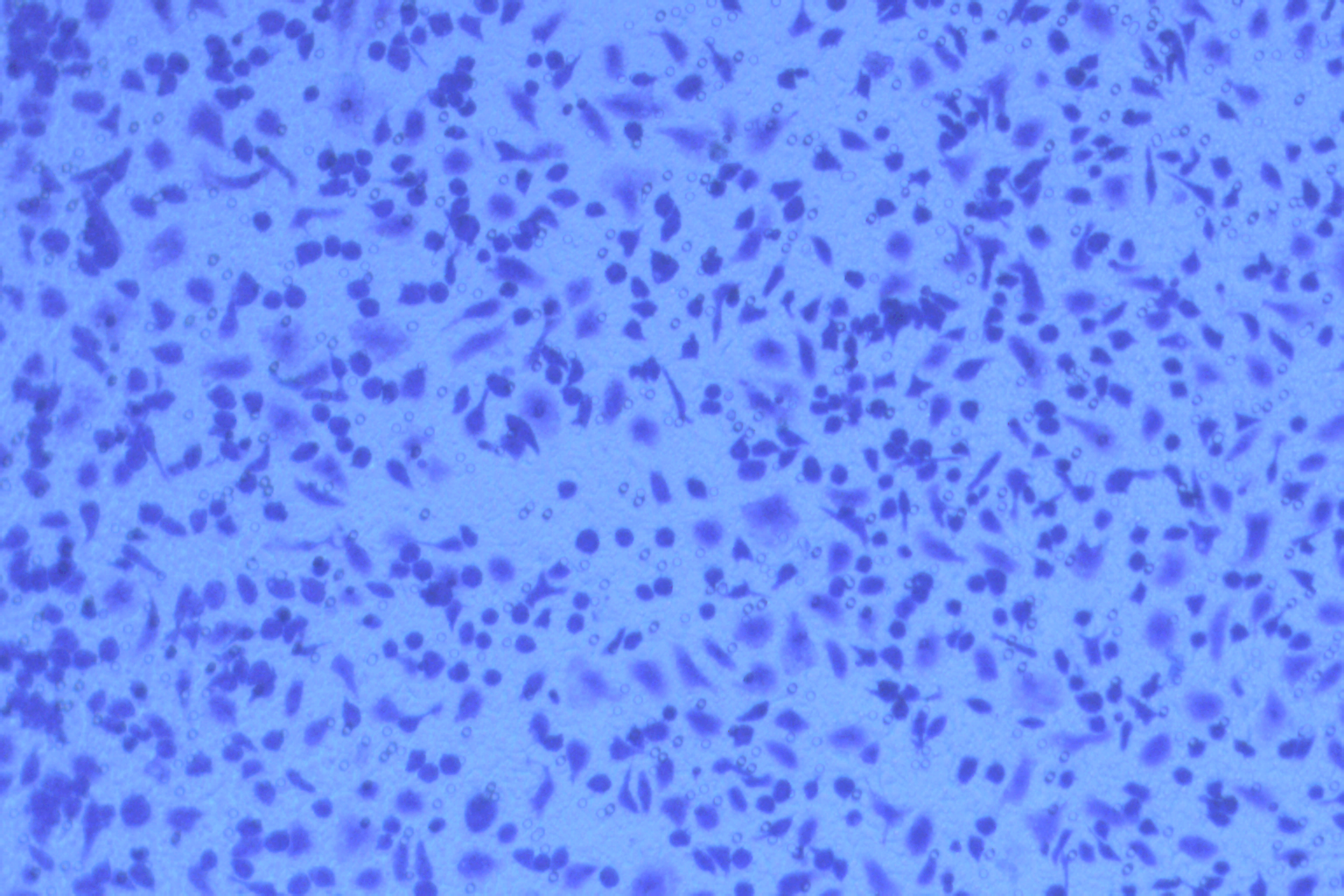

Supplement: Supplementary file 1 — Supplementary Information. [file 41598_2023_39765_MOESM1_ESM.zip › ╘¡╩╝╩2╛▌╒√└φ/transwell/keratinase (2).bmp]

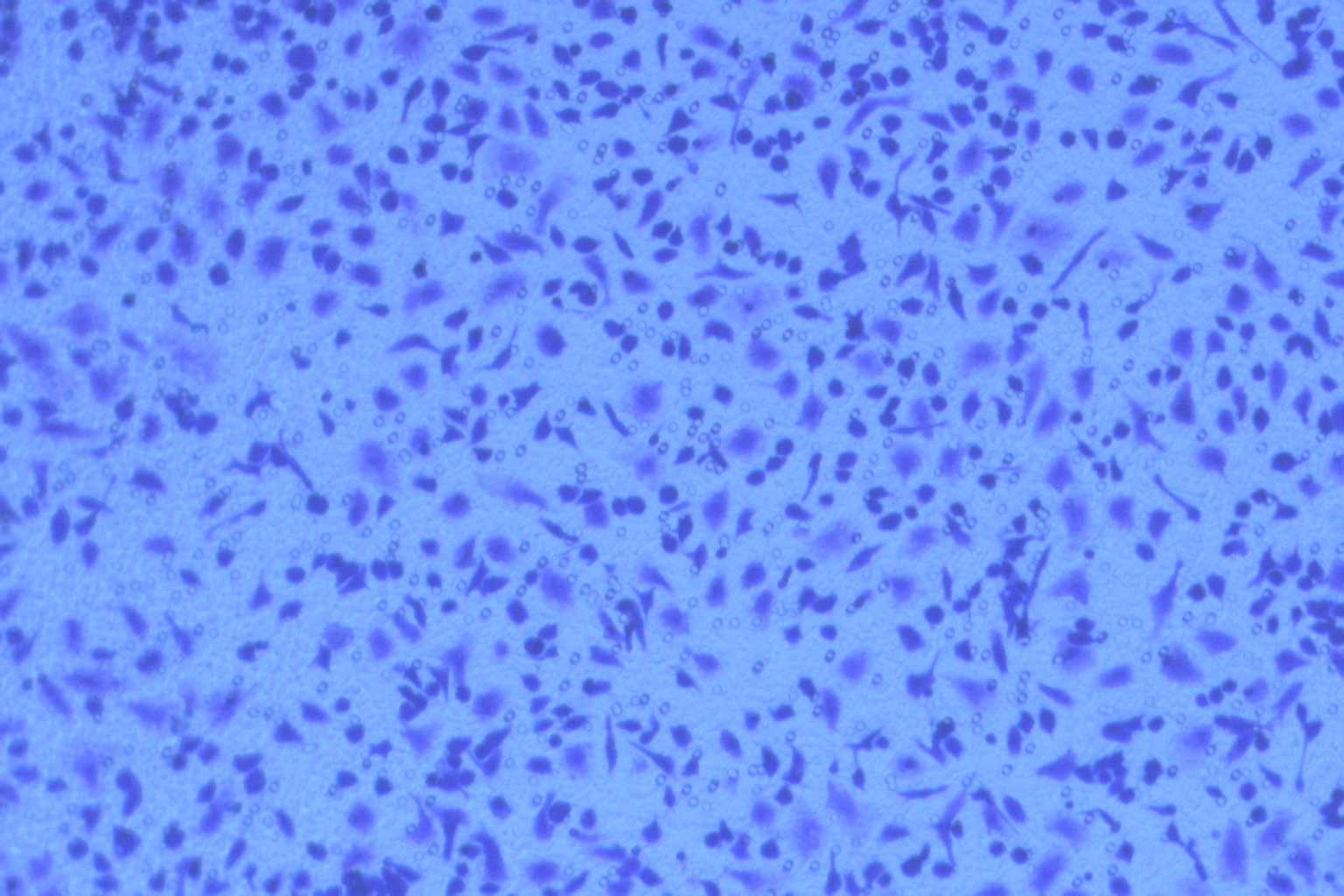

Supplement: Supplementary file 1 — Supplementary Information. [file 41598_2023_39765_MOESM1_ESM.zip › ╘¡╩╝╩2╛▌╒√└φ/transwell/keratinase (3).bmp]
