# Supplementary figures and images for: Eschar dissolution and the immunoregulator effect of keratinase on burn wounds (part 1 of 2)
Source: Sci Rep. 2023 Aug 14;13:13238. doi: 10.1038/s41598-023-39765-4 (PMC10425458; doi:10.1038/s41598-023-39765-4)

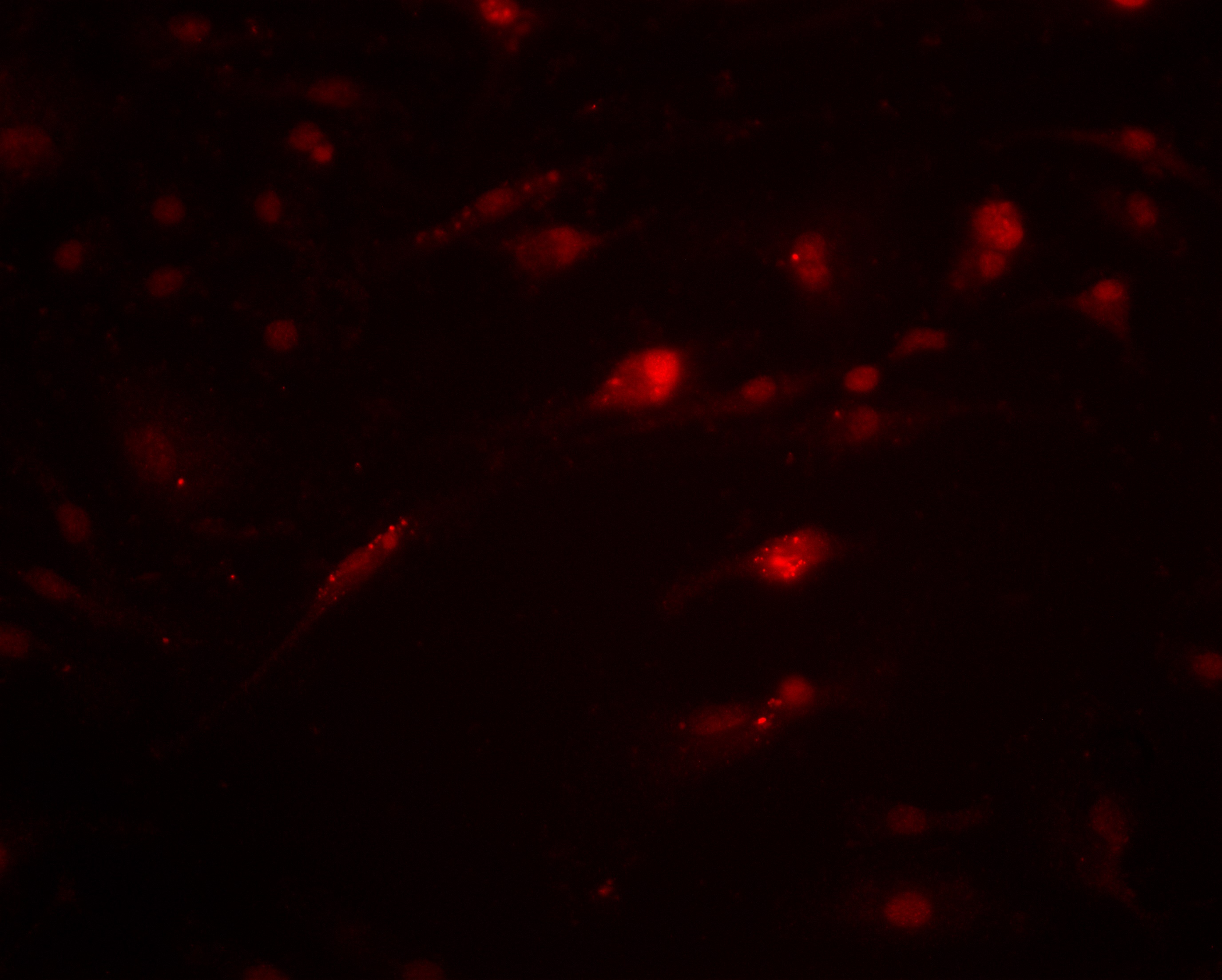

Supplement: Supplementary file 1 — Supplementary Information. [file 41598_2023_39765_MOESM1_ESM.zip › ╘¡╩╝╩2╛▌╒√└φ/cell immunofluorescence/Arg-1/control.603/c2 (1).jpg]

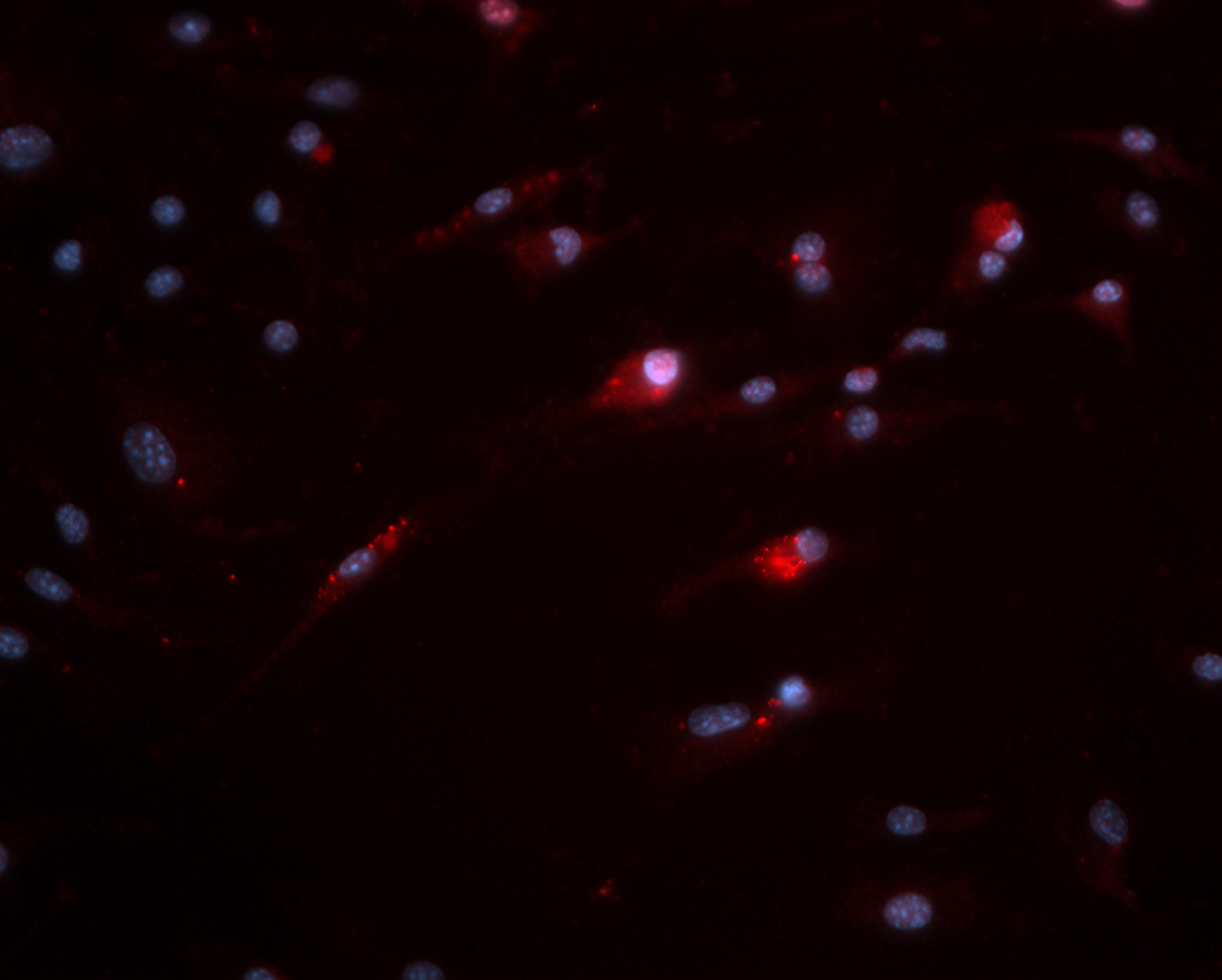

Supplement: Supplementary file 1 — Supplementary Information. [file 41598_2023_39765_MOESM1_ESM.zip › ╘¡╩╝╩2╛▌╒√└φ/cell immunofluorescence/Arg-1/control.603/c2 (2).jpg]

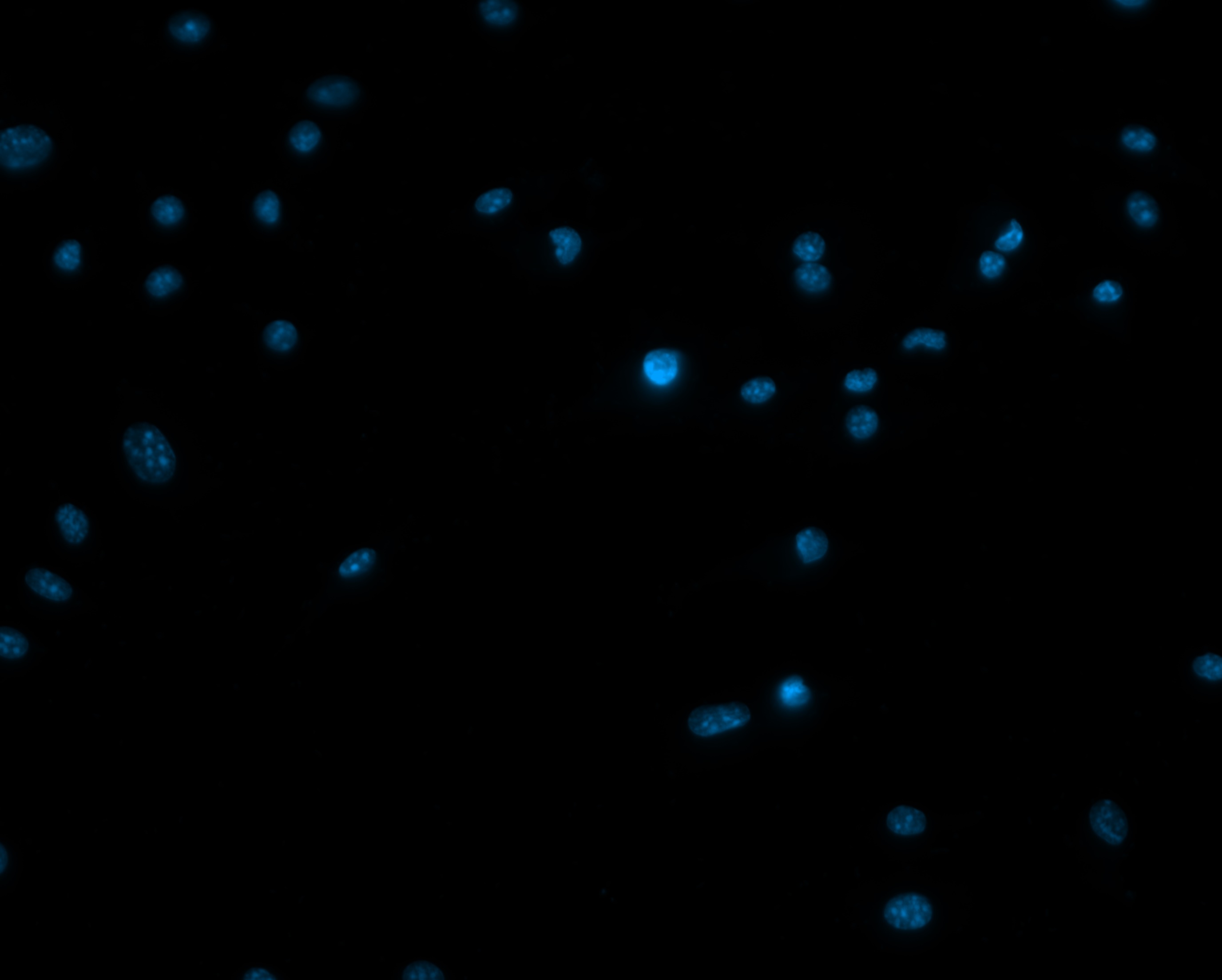

Supplement: Supplementary file 1 — Supplementary Information. [file 41598_2023_39765_MOESM1_ESM.zip › ╘¡╩╝╩2╛▌╒√└φ/cell immunofluorescence/Arg-1/control.603/c2 (3).jpg]

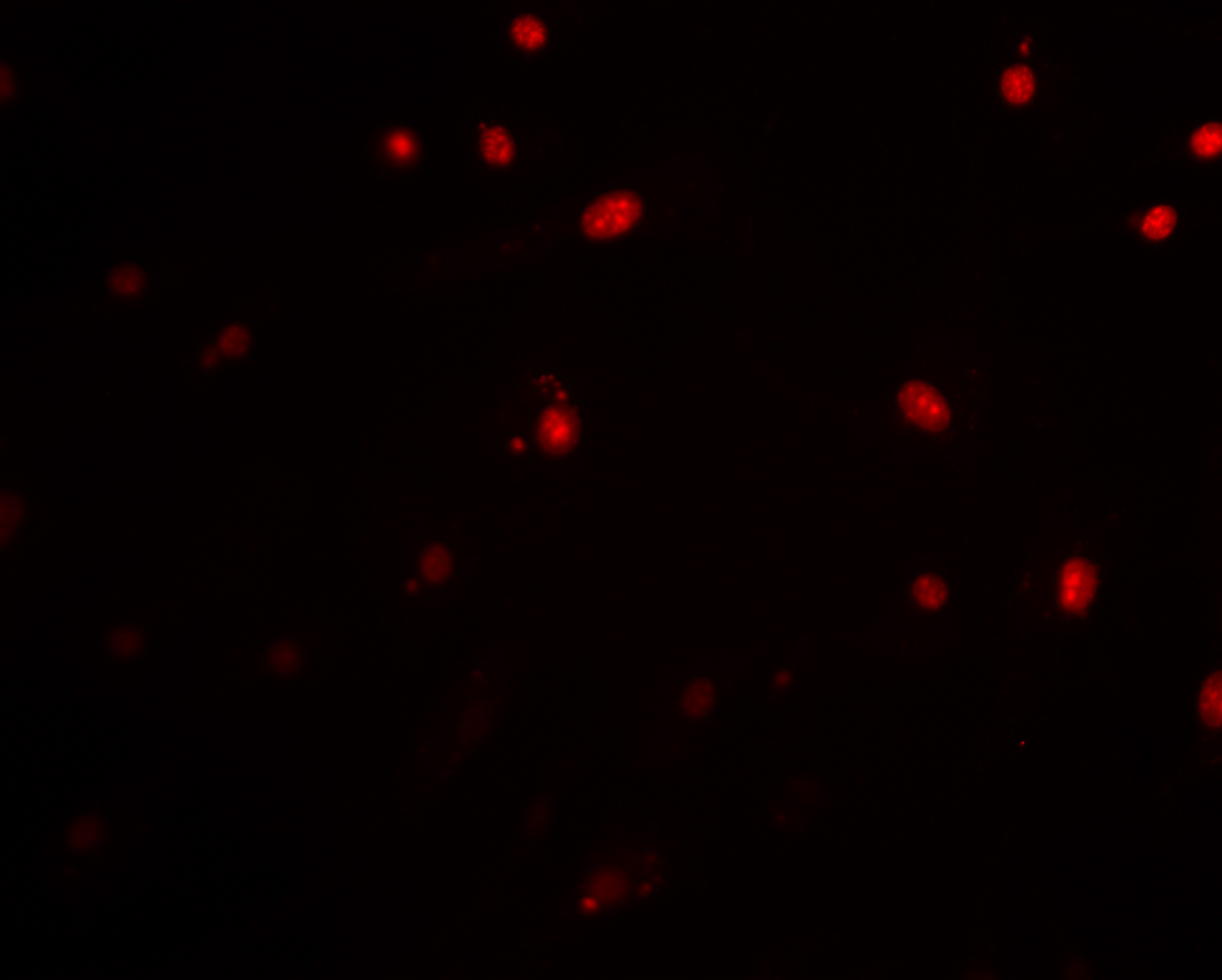

Supplement: Supplementary file 1 — Supplementary Information. [file 41598_2023_39765_MOESM1_ESM.zip › ╘¡╩╝╩2╛▌╒√└φ/cell immunofluorescence/Arg-1/control.889/c3 (1).jpg]

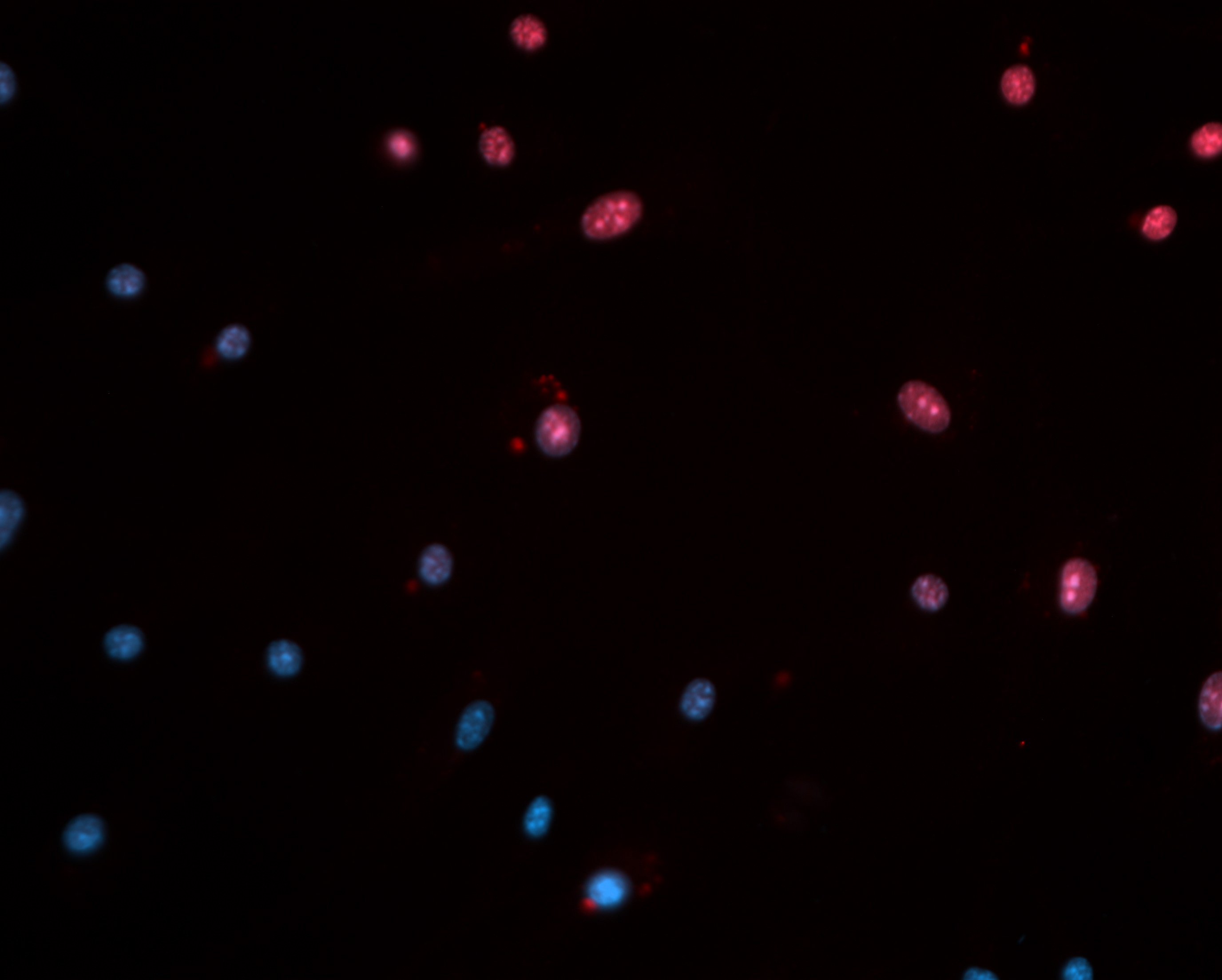

Supplement: Supplementary file 1 — Supplementary Information. [file 41598_2023_39765_MOESM1_ESM.zip › ╘¡╩╝╩2╛▌╒√└φ/cell immunofluorescence/Arg-1/control.889/c3 (2).jpg]

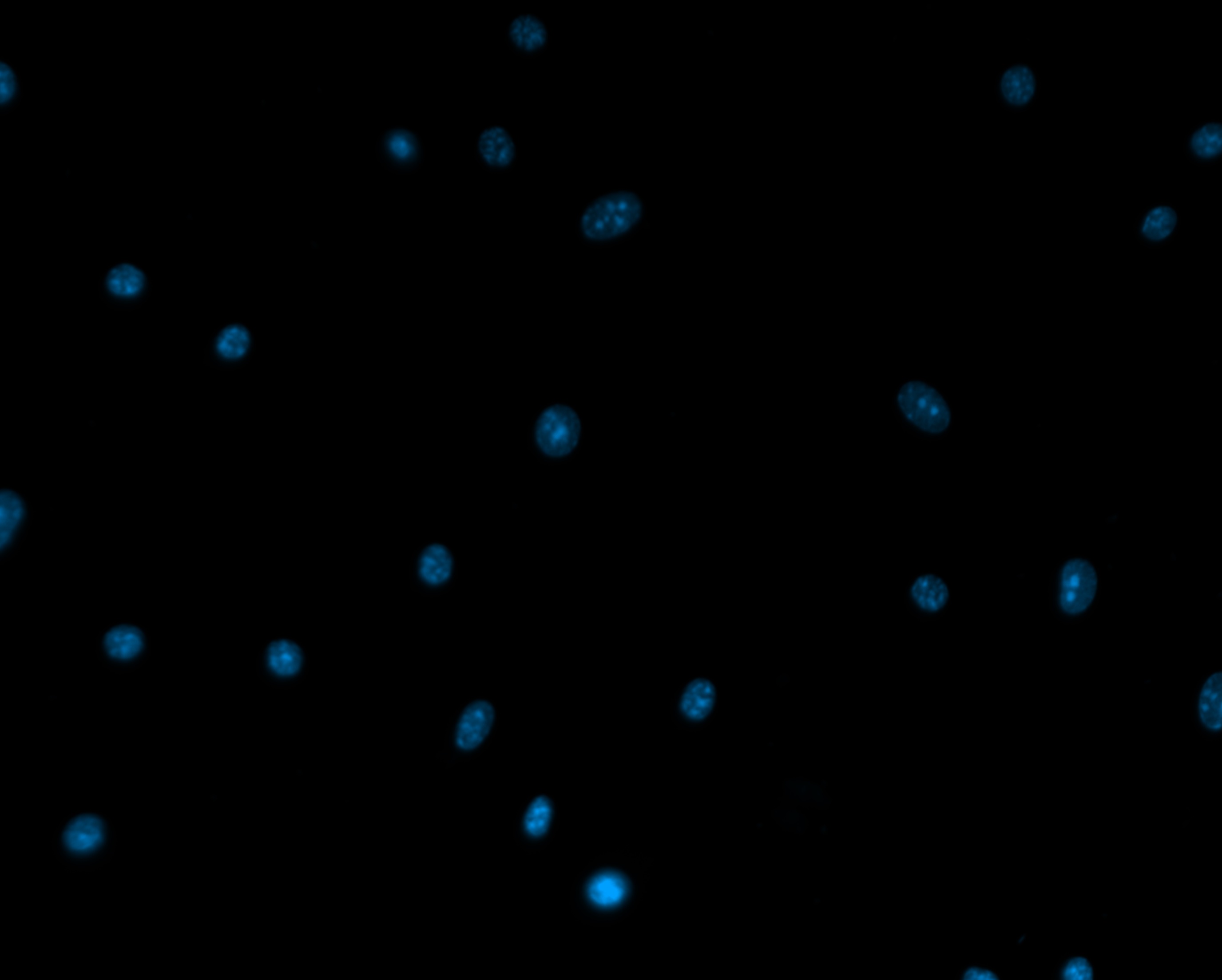

Supplement: Supplementary file 1 — Supplementary Information. [file 41598_2023_39765_MOESM1_ESM.zip › ╘¡╩╝╩2╛▌╒√└φ/cell immunofluorescence/Arg-1/control.889/c3 (3).jpg]

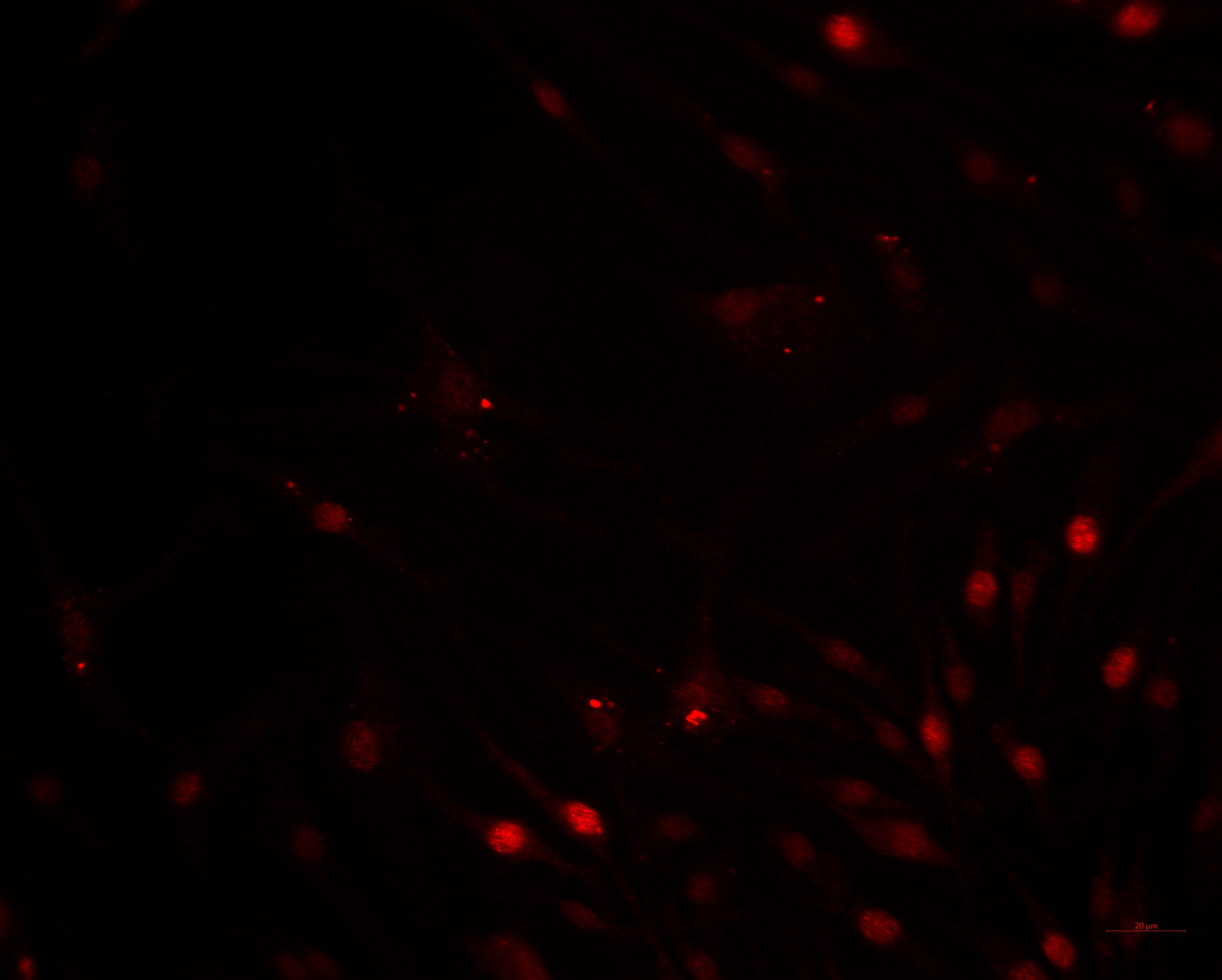

Supplement: Supplementary file 1 — Supplementary Information. [file 41598_2023_39765_MOESM1_ESM.zip › ╘¡╩╝╩2╛▌╒√└φ/cell immunofluorescence/Arg-1/control/c (1).jpg]

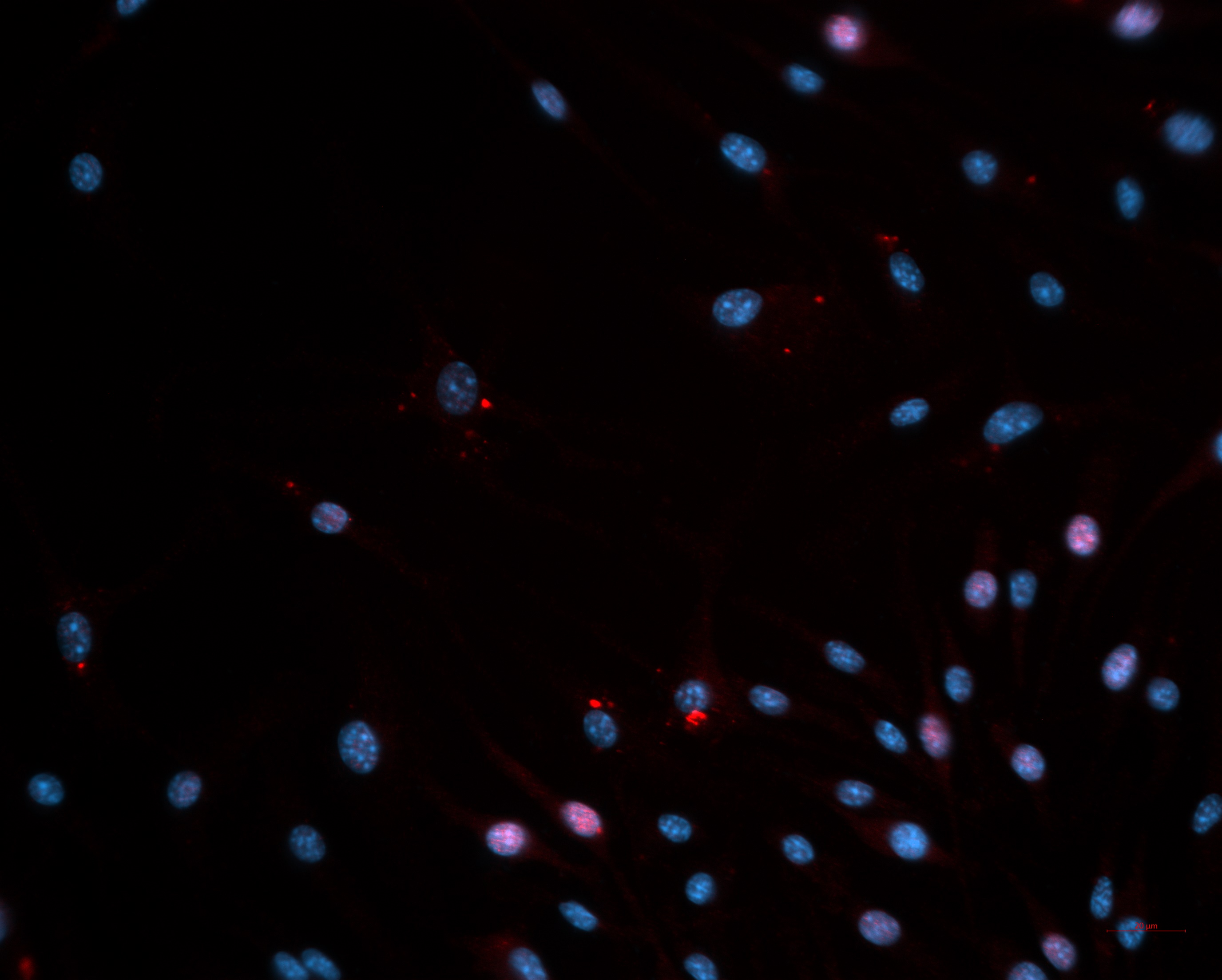

Supplement: Supplementary file 1 — Supplementary Information. [file 41598_2023_39765_MOESM1_ESM.zip › ╘¡╩╝╩2╛▌╒√└φ/cell immunofluorescence/Arg-1/control/c (2).jpg]

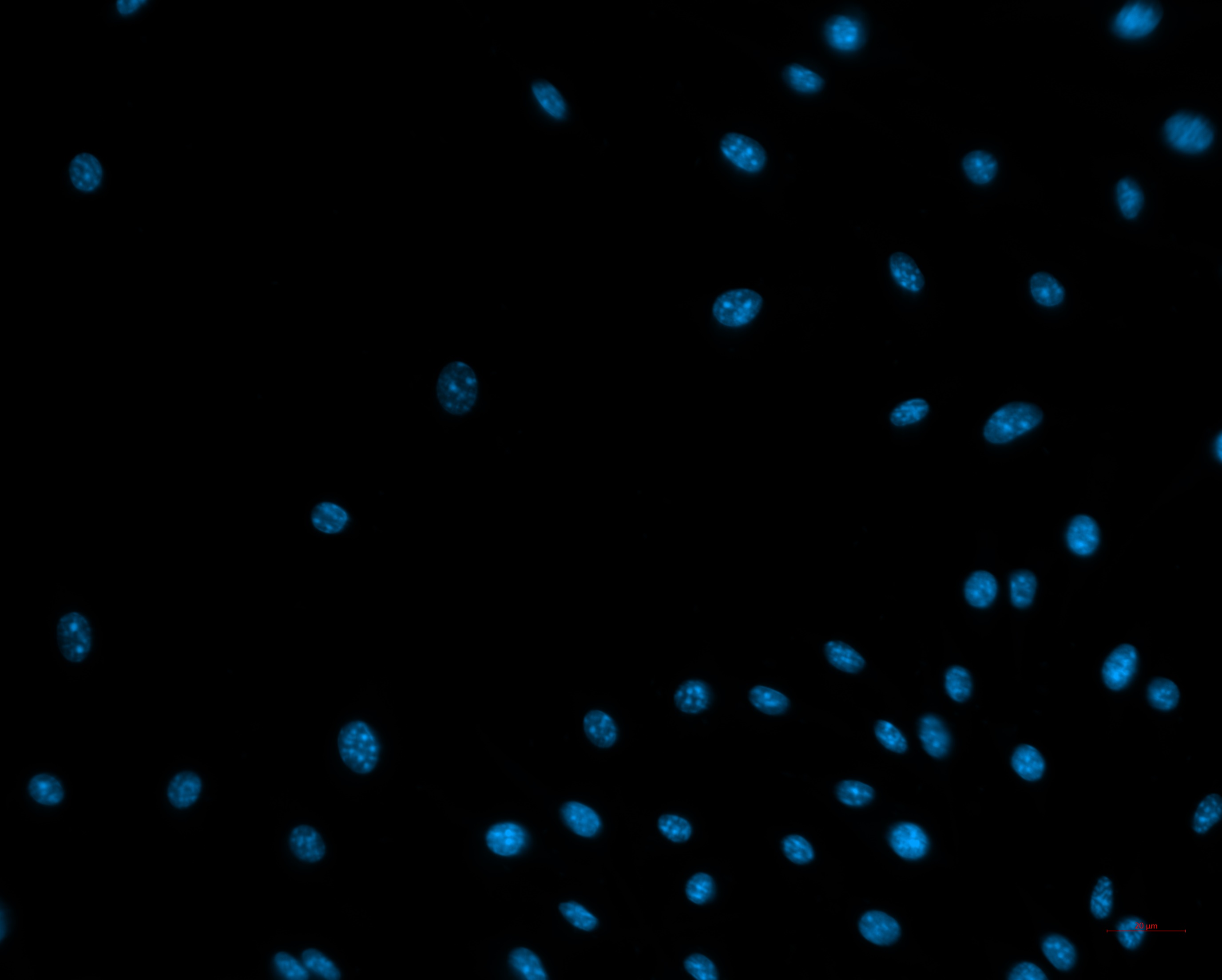

Supplement: Supplementary file 1 — Supplementary Information. [file 41598_2023_39765_MOESM1_ESM.zip › ╘¡╩╝╩2╛▌╒√└φ/cell immunofluorescence/Arg-1/control/c (3).jpg]

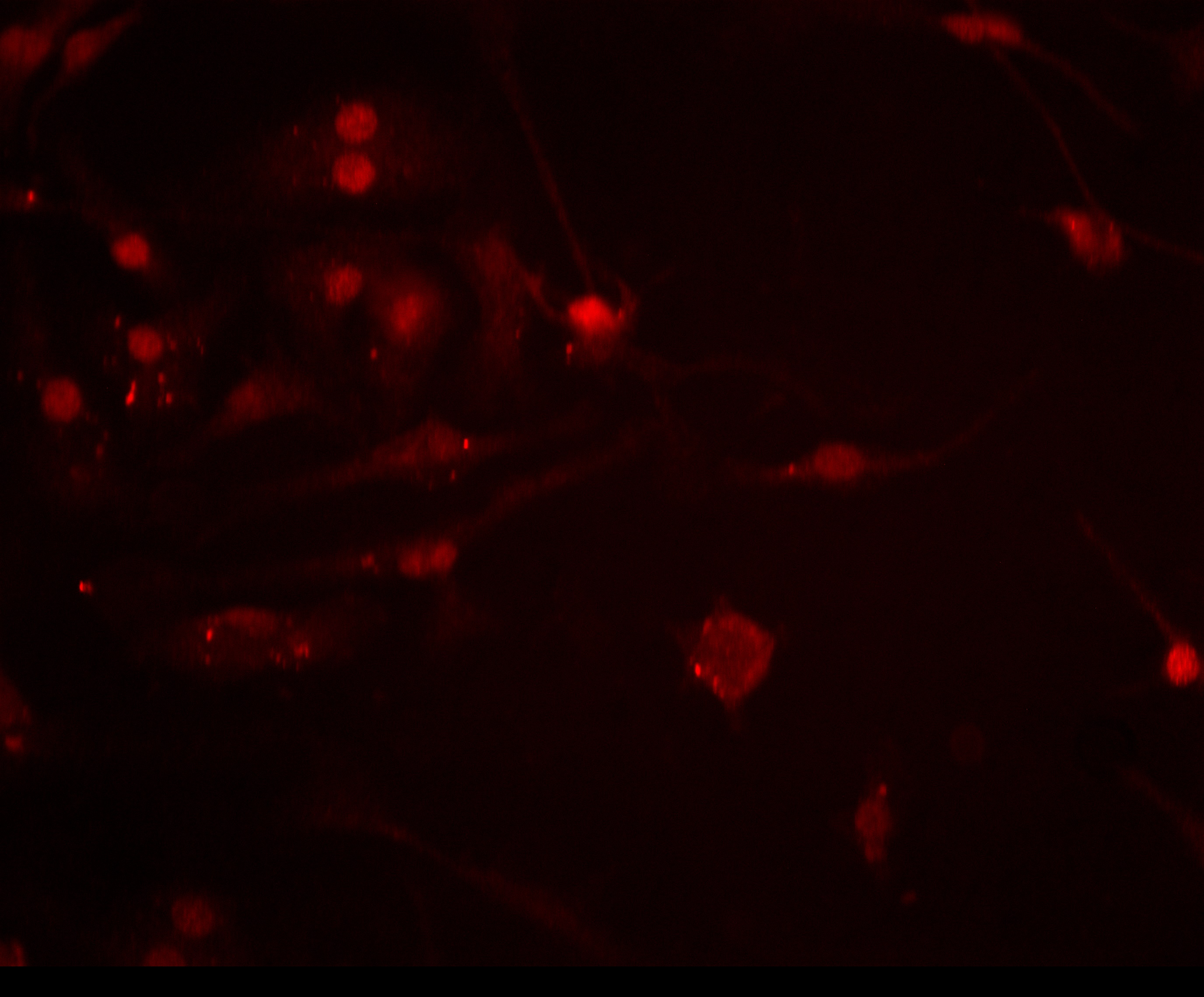

Supplement: Supplementary file 1 — Supplementary Information. [file 41598_2023_39765_MOESM1_ESM.zip › ╘¡╩╝╩2╛▌╒√└φ/cell immunofluorescence/Arg-1/keratinase.101/k (1).jpg]

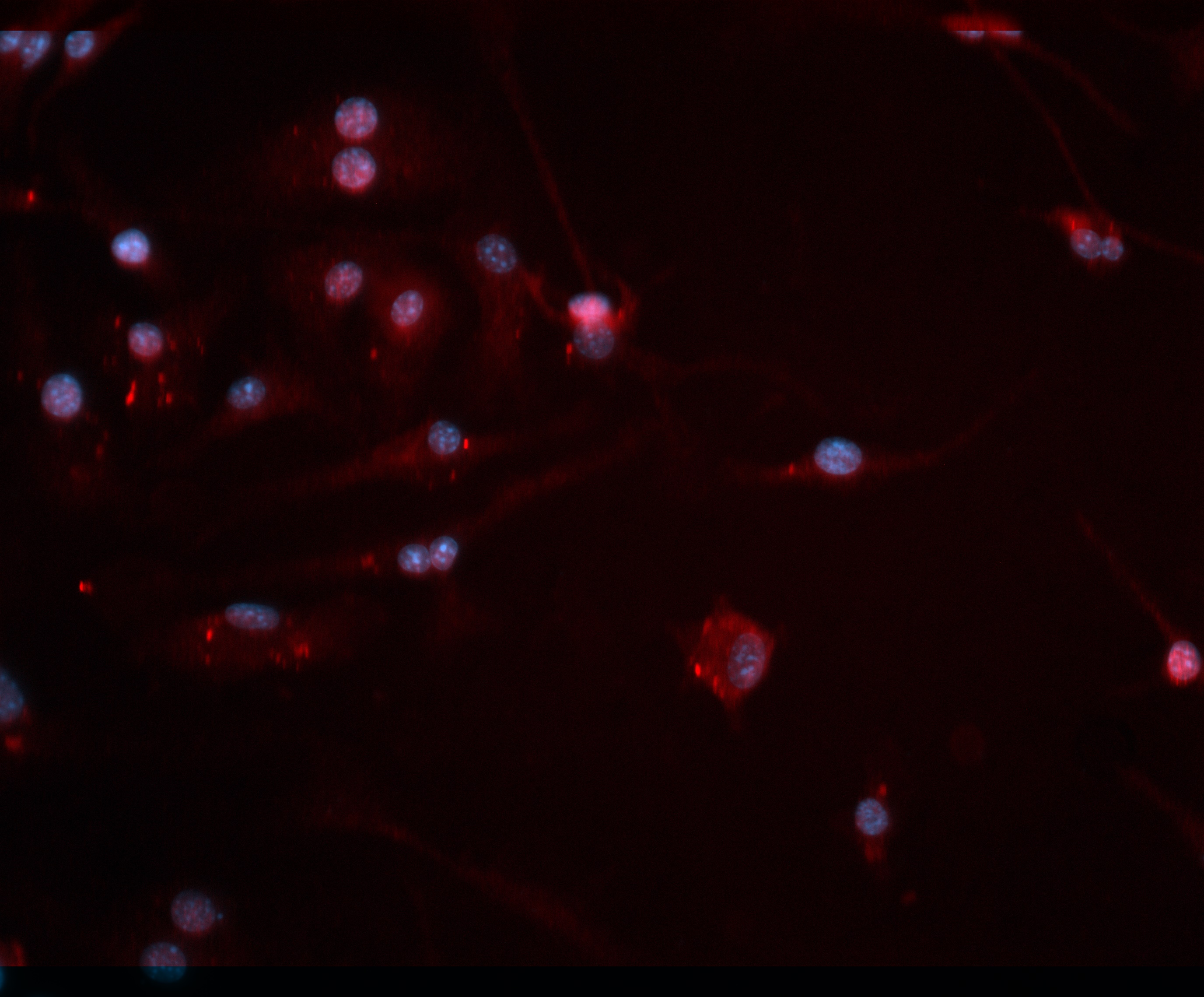

Supplement: Supplementary file 1 — Supplementary Information. [file 41598_2023_39765_MOESM1_ESM.zip › ╘¡╩╝╩2╛▌╒√└φ/cell immunofluorescence/Arg-1/keratinase.101/k (2).jpg]

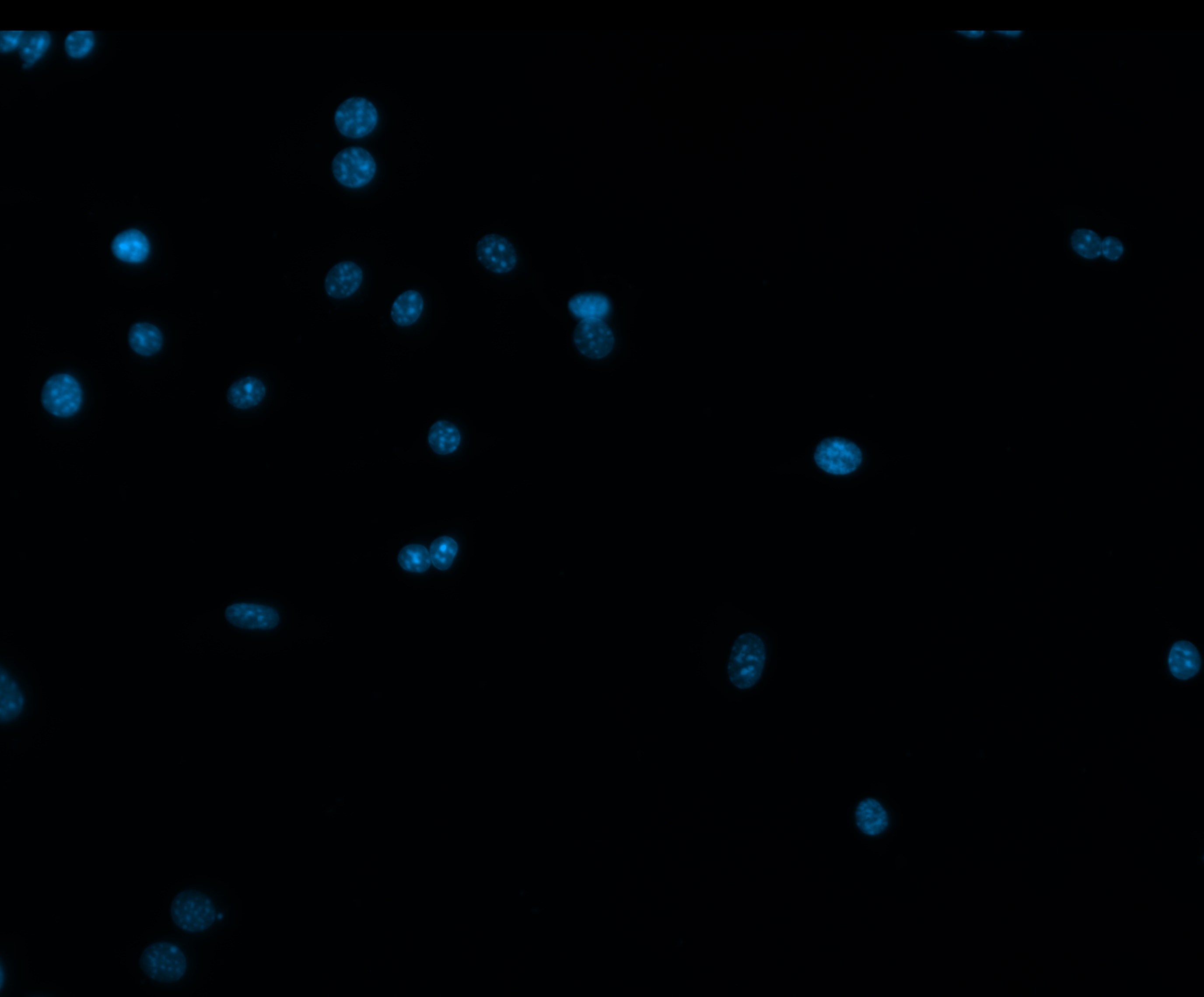

Supplement: Supplementary file 1 — Supplementary Information. [file 41598_2023_39765_MOESM1_ESM.zip › ╘¡╩╝╩2╛▌╒√└φ/cell immunofluorescence/Arg-1/keratinase.101/k (3).jpg]

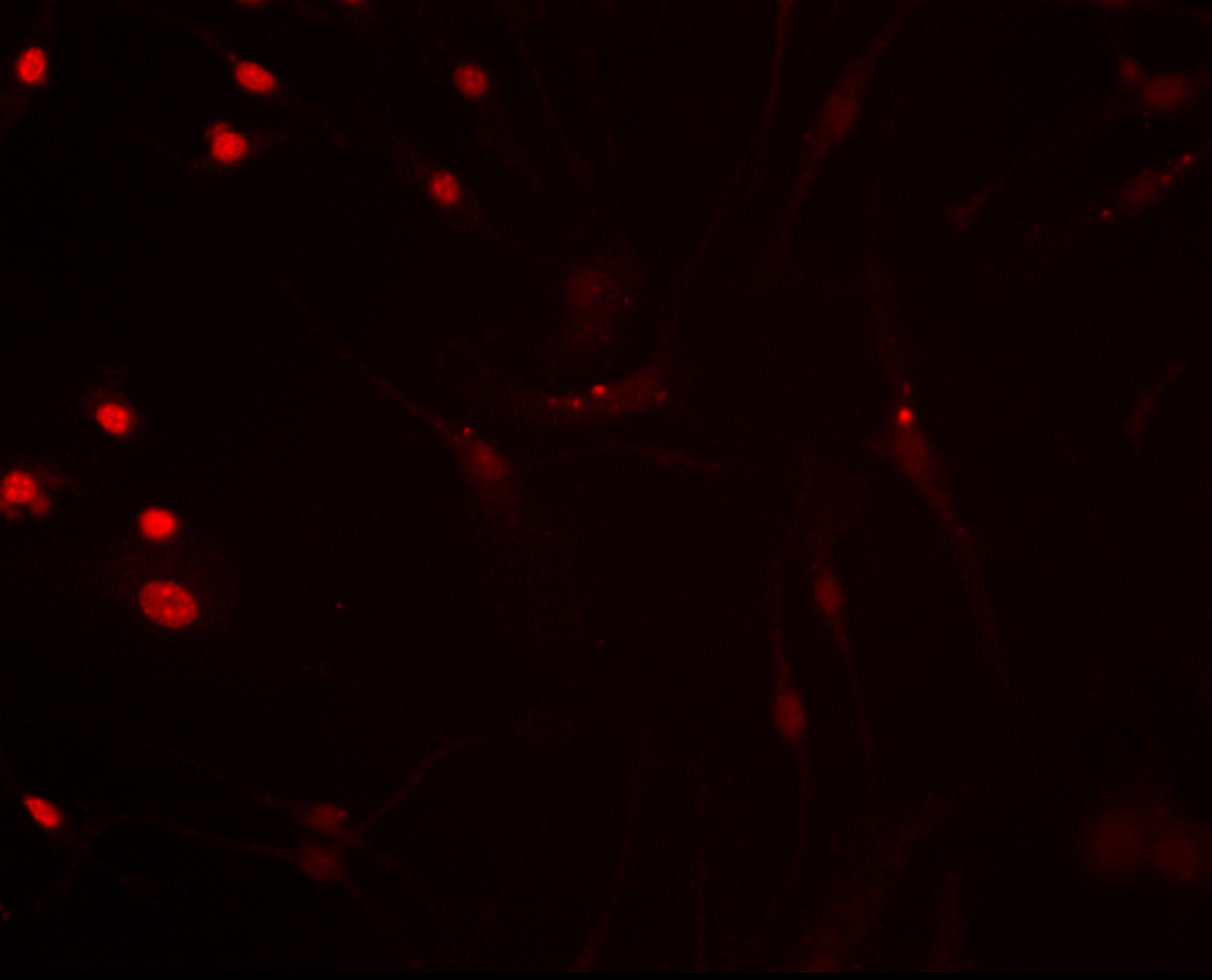

Supplement: Supplementary file 1 — Supplementary Information. [file 41598_2023_39765_MOESM1_ESM.zip › ╘¡╩╝╩2╛▌╒√└φ/cell immunofluorescence/Arg-1/keratinase.282/k2 (1).jpg]

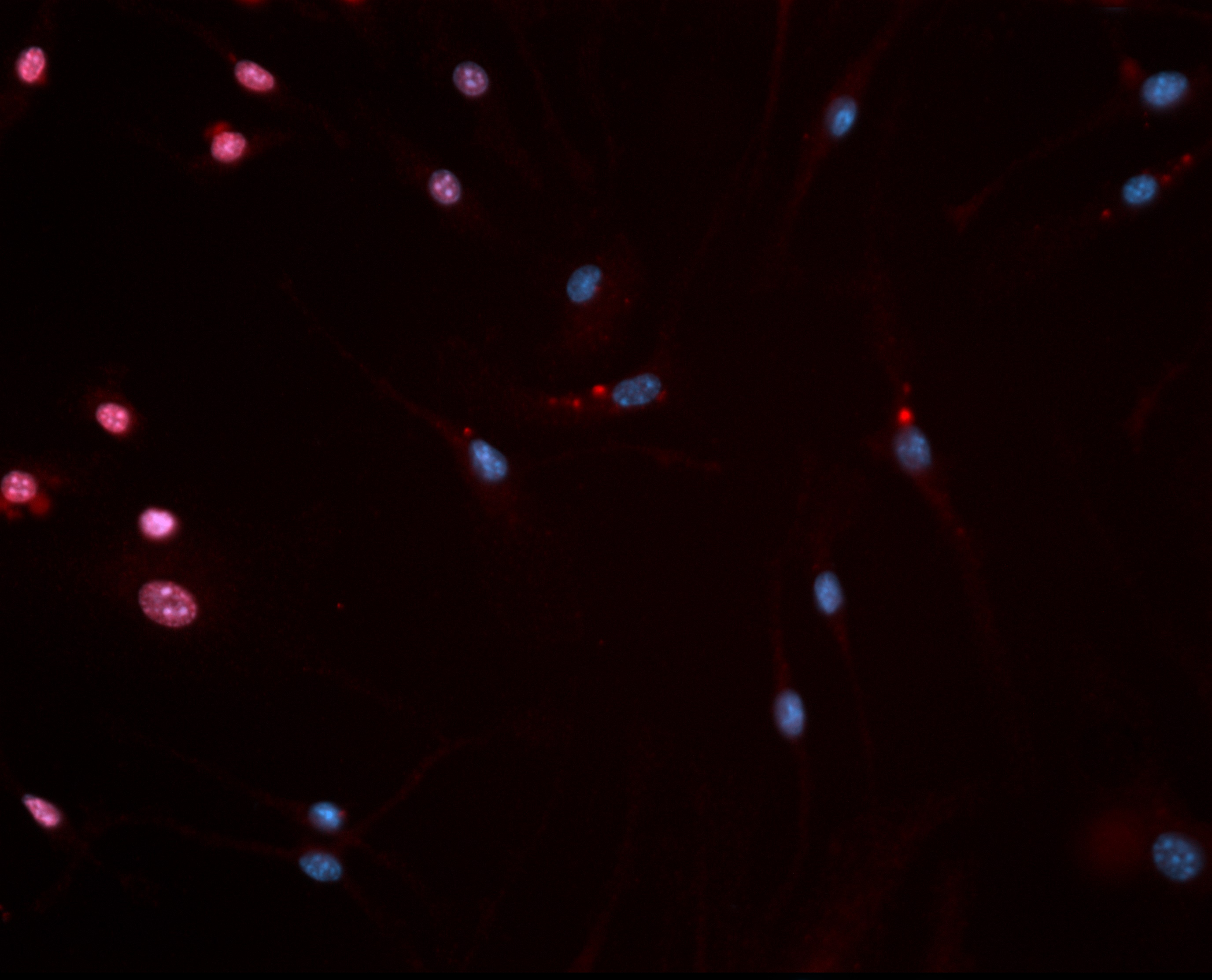

Supplement: Supplementary file 1 — Supplementary Information. [file 41598_2023_39765_MOESM1_ESM.zip › ╘¡╩╝╩2╛▌╒√└φ/cell immunofluorescence/Arg-1/keratinase.282/k2 (2).jpg]

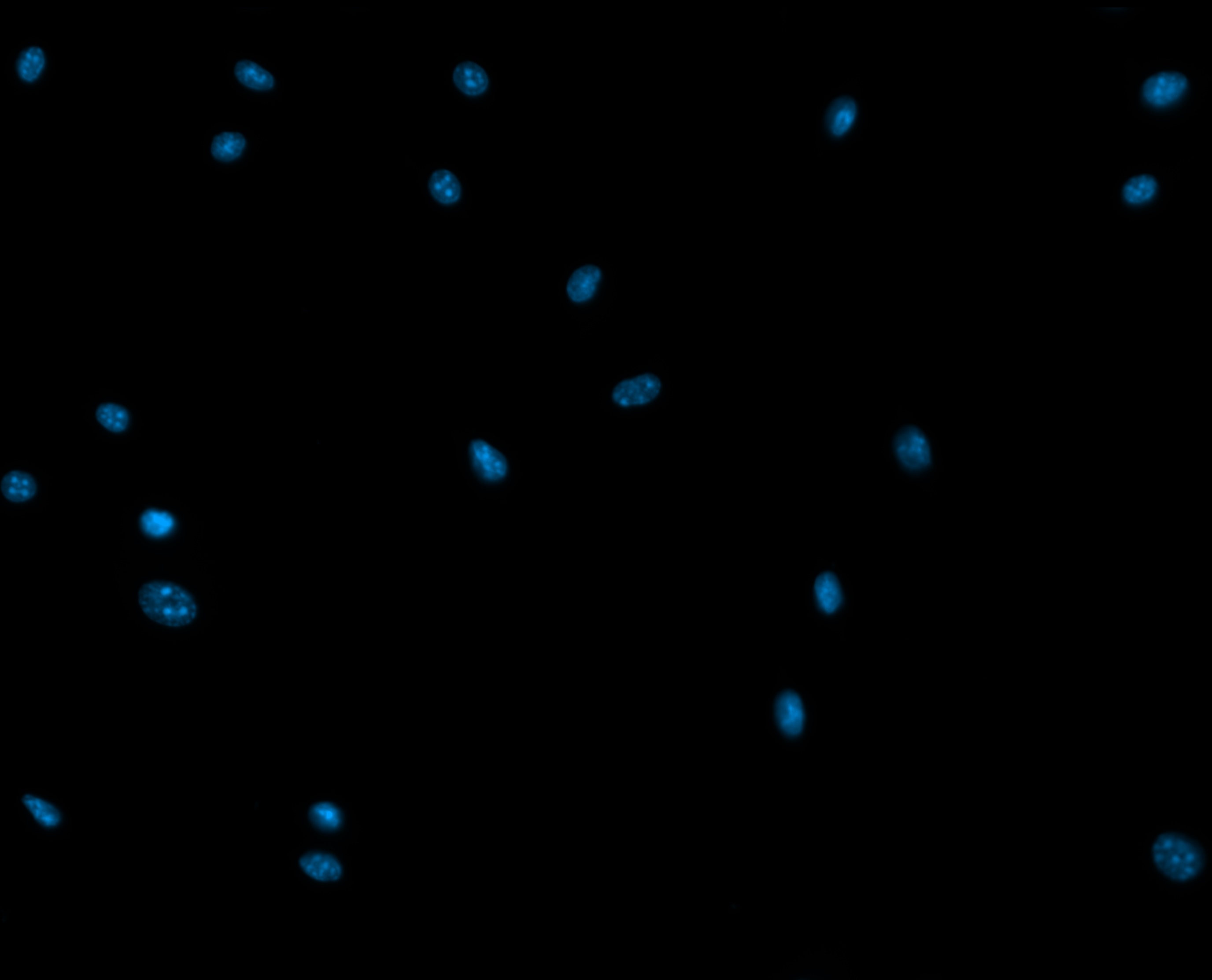

Supplement: Supplementary file 1 — Supplementary Information. [file 41598_2023_39765_MOESM1_ESM.zip › ╘¡╩╝╩2╛▌╒√└φ/cell immunofluorescence/Arg-1/keratinase.282/k2 (3).jpg]

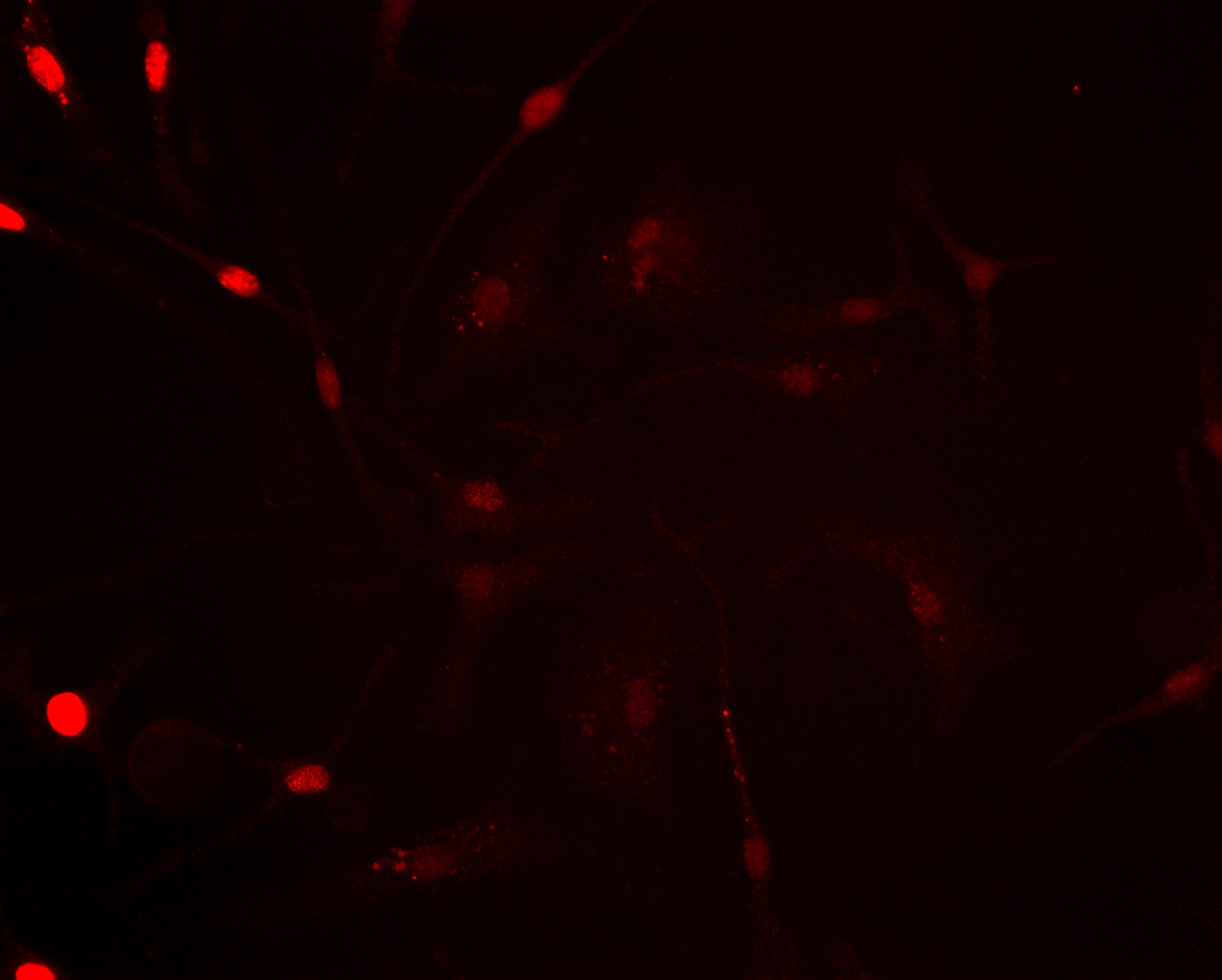

Supplement: Supplementary file 1 — Supplementary Information. [file 41598_2023_39765_MOESM1_ESM.zip › ╘¡╩╝╩2╛▌╒√└φ/cell immunofluorescence/Arg-1/keratinase.9285/k3 (1).jpg]

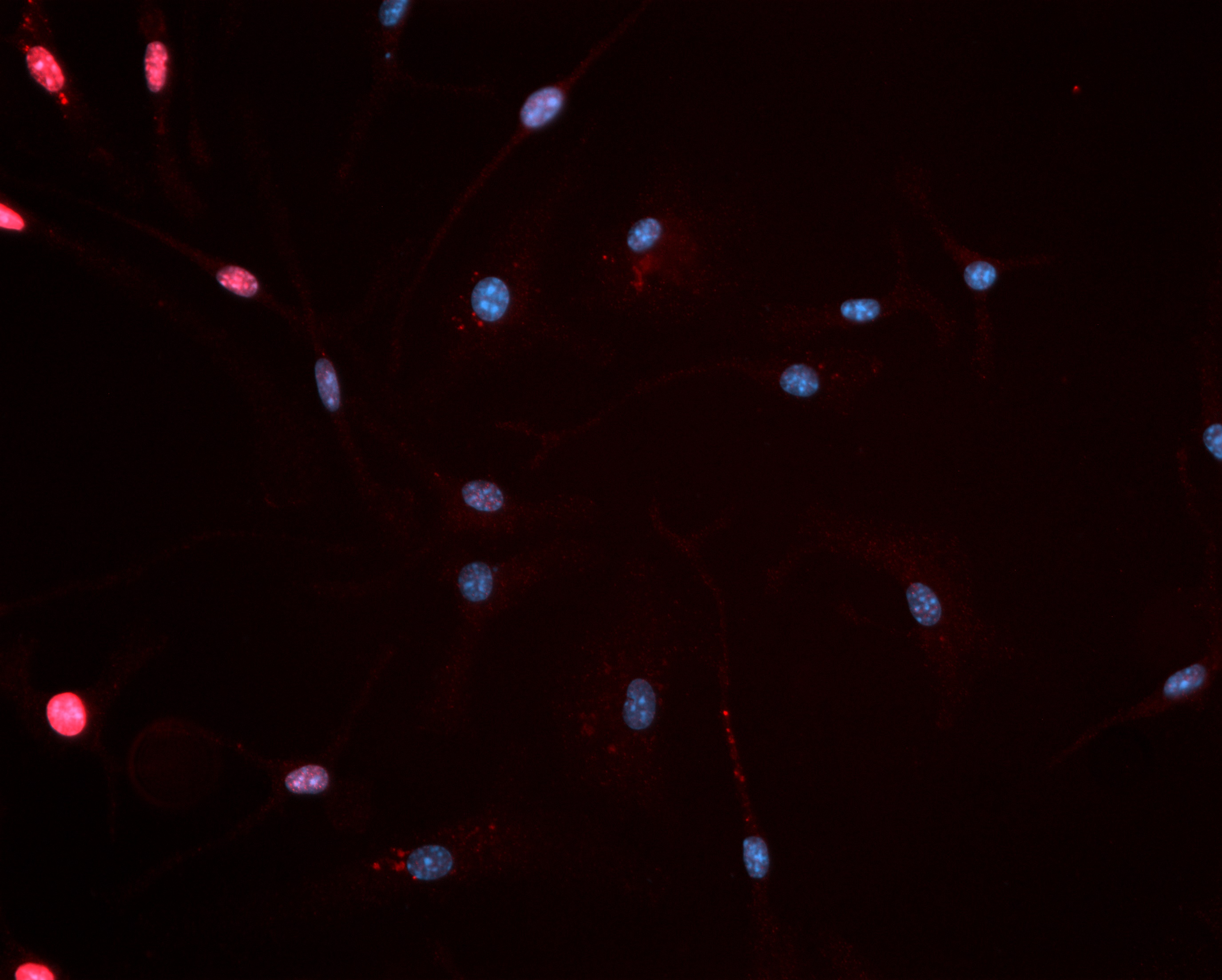

Supplement: Supplementary file 1 — Supplementary Information. [file 41598_2023_39765_MOESM1_ESM.zip › ╘¡╩╝╩2╛▌╒√└φ/cell immunofluorescence/Arg-1/keratinase.9285/k3 (2).jpg]

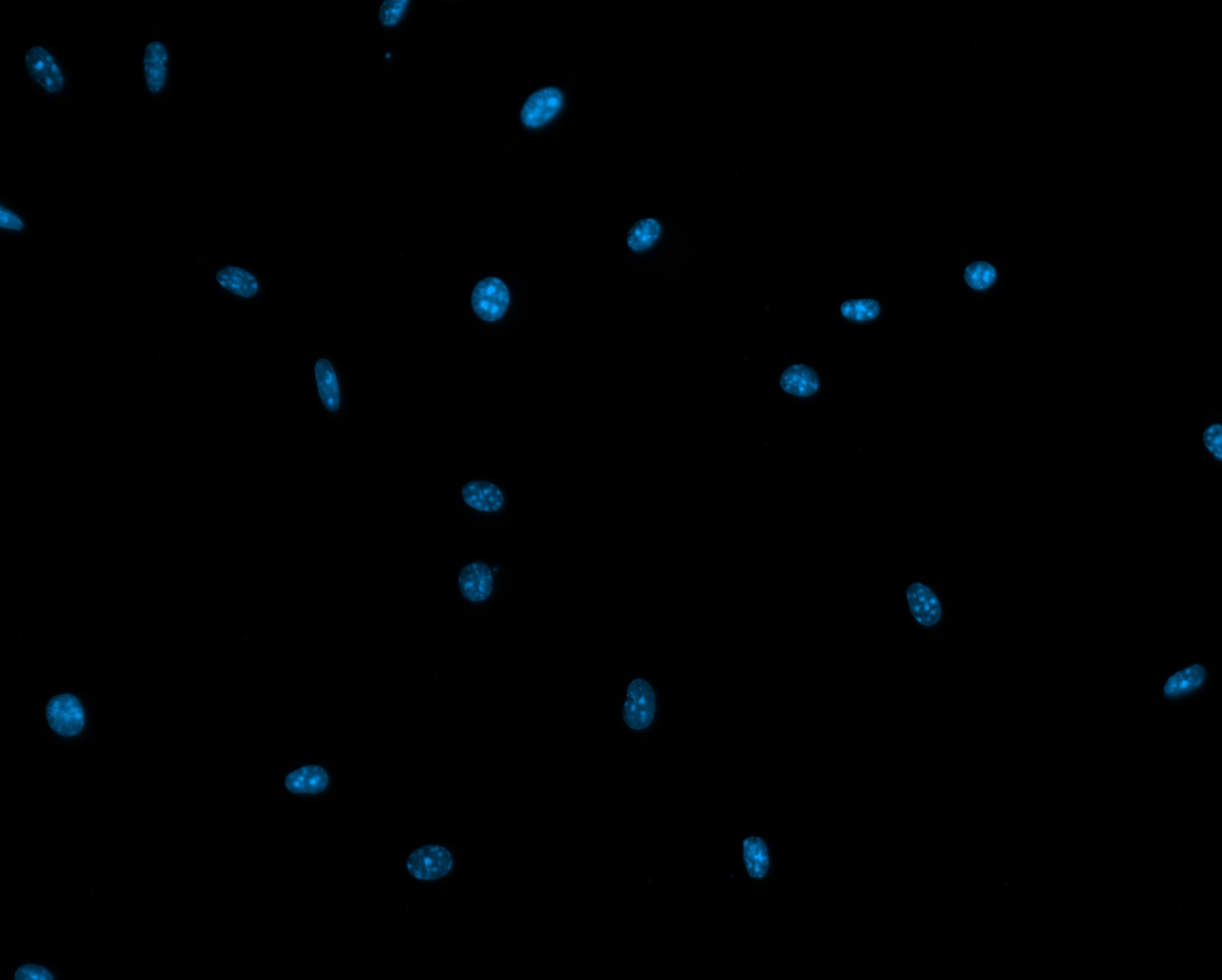

Supplement: Supplementary file 1 — Supplementary Information. [file 41598_2023_39765_MOESM1_ESM.zip › ╘¡╩╝╩2╛▌╒√└φ/cell immunofluorescence/Arg-1/keratinase.9285/k3 (3).jpg]

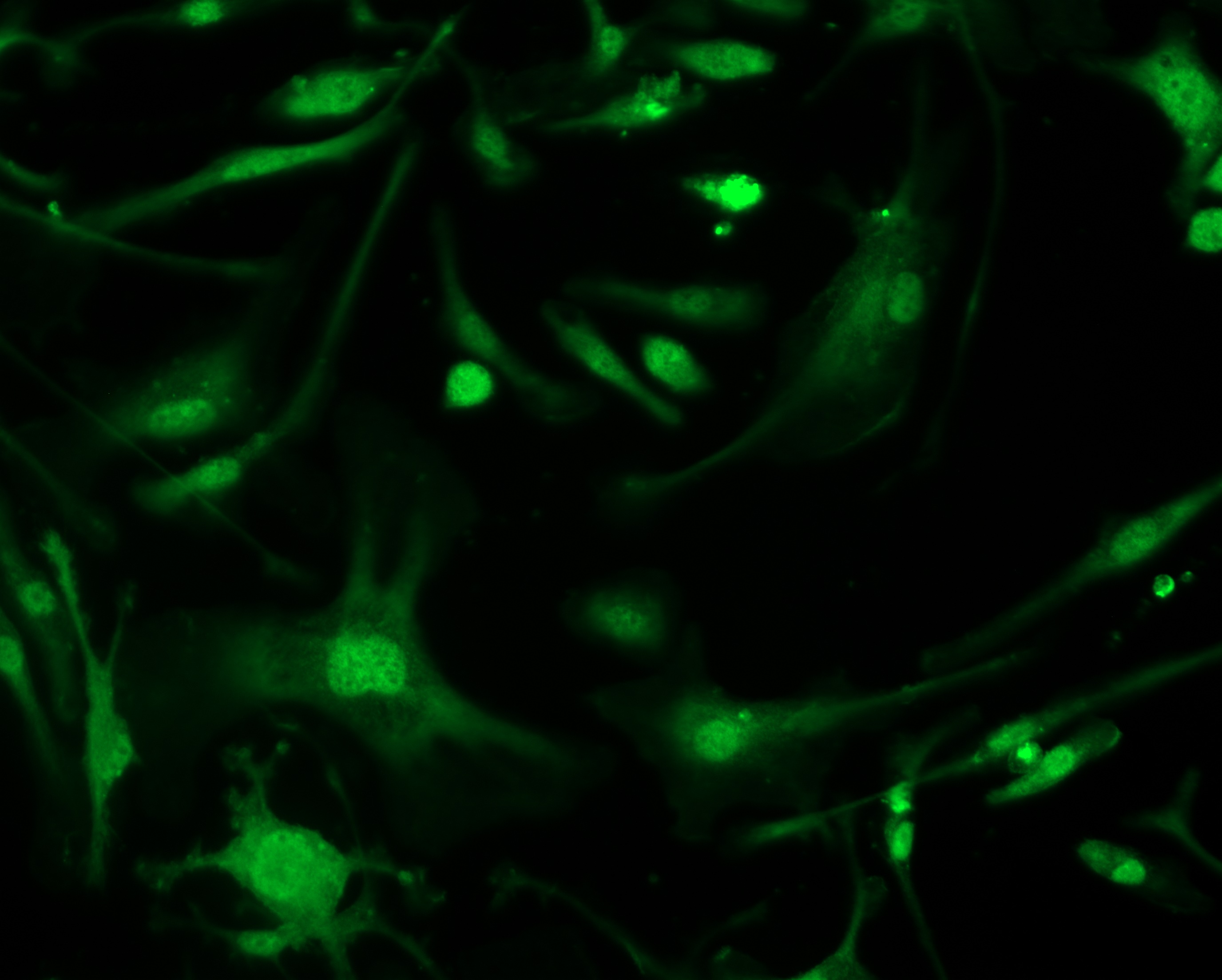

Supplement: Supplementary file 1 — Supplementary Information. [file 41598_2023_39765_MOESM1_ESM.zip › ╘¡╩╝╩2╛▌╒√└φ/cell immunofluorescence/inos/CONTROL.101/B4_c1.jpg]

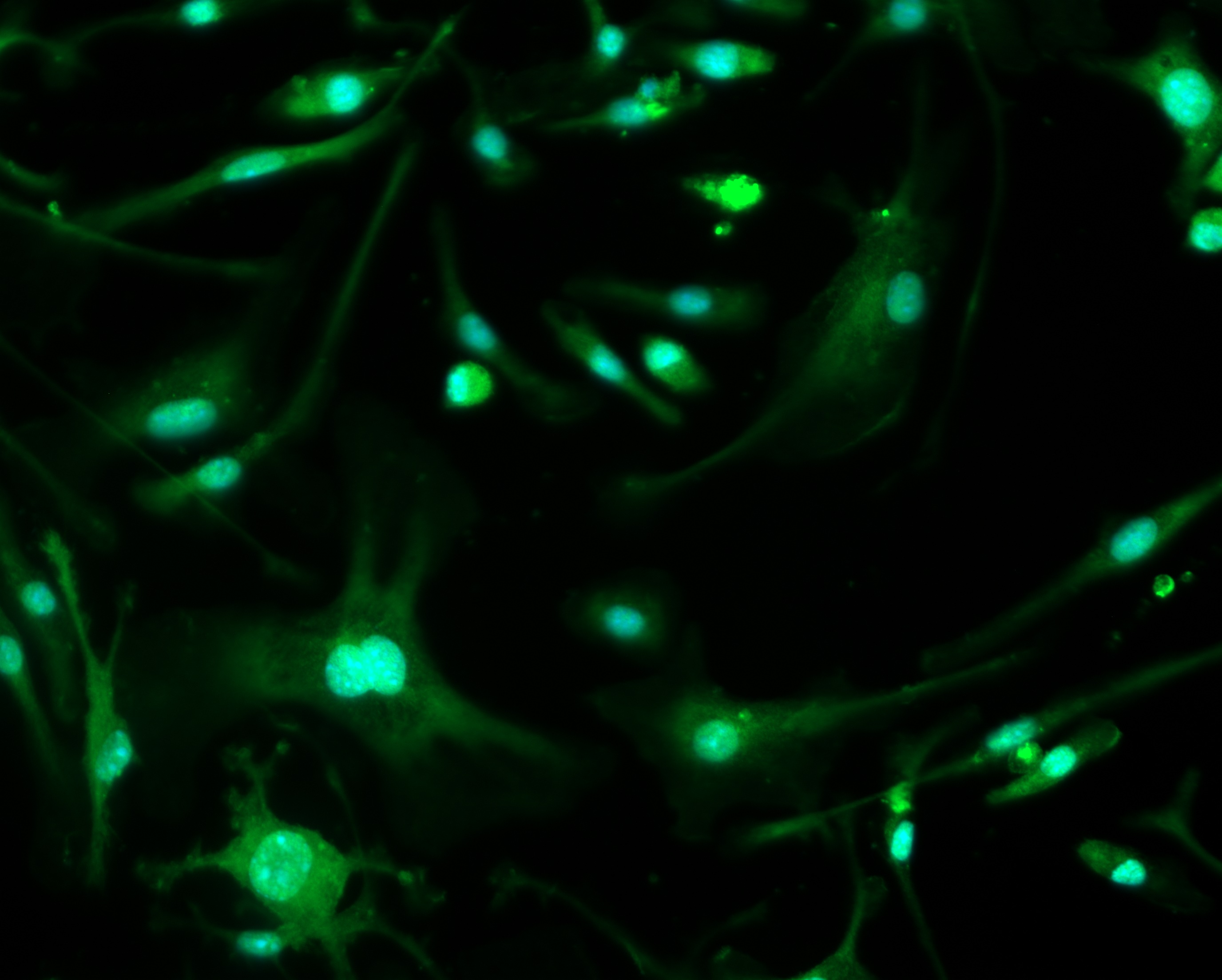

Supplement: Supplementary file 1 — Supplementary Information. [file 41598_2023_39765_MOESM1_ESM.zip › ╘¡╩╝╩2╛▌╒√└φ/cell immunofluorescence/inos/CONTROL.101/B4_c1+2.jpg]

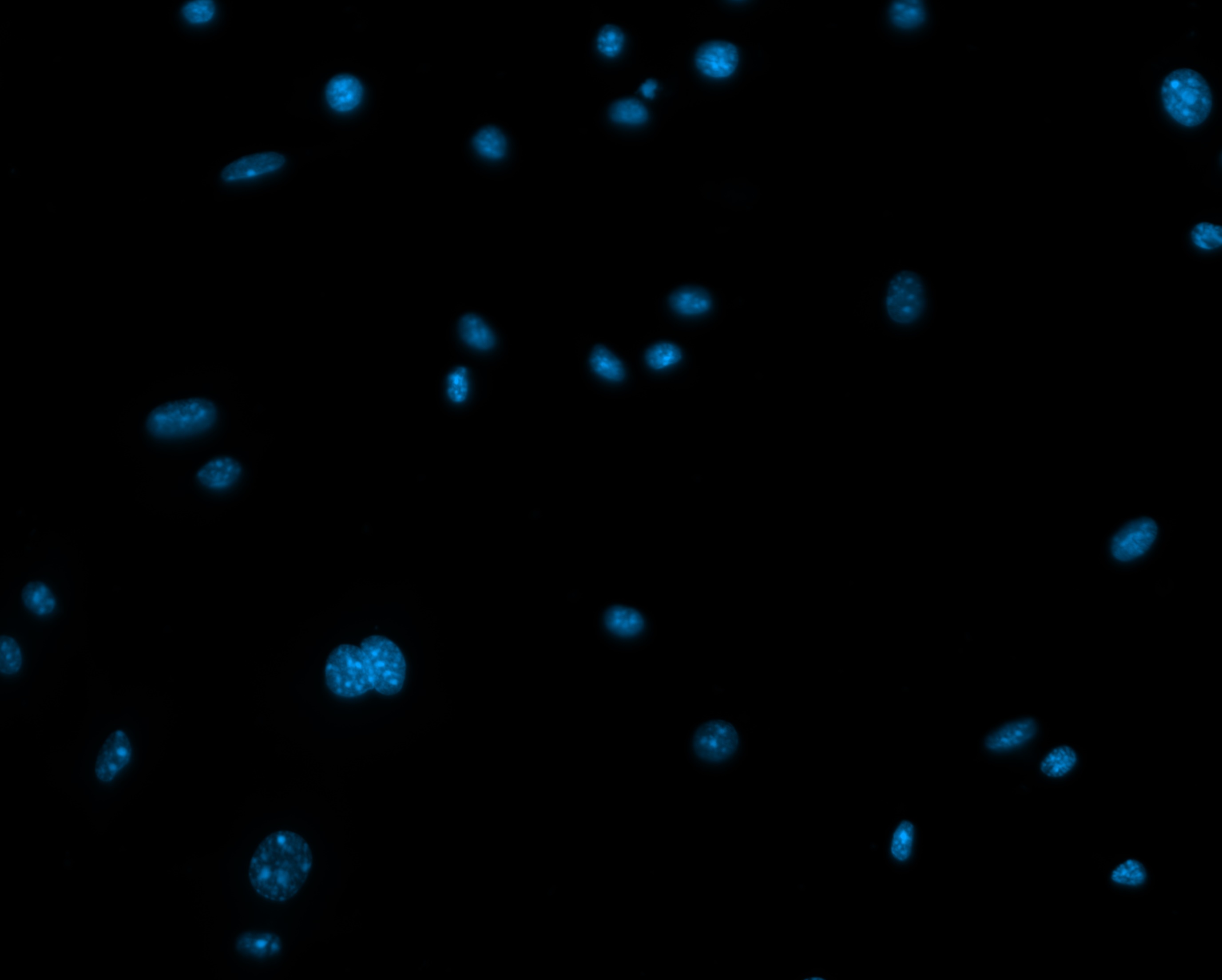

Supplement: Supplementary file 1 — Supplementary Information. [file 41598_2023_39765_MOESM1_ESM.zip › ╘¡╩╝╩2╛▌╒√└φ/cell immunofluorescence/inos/CONTROL.101/B4_c2.jpg]

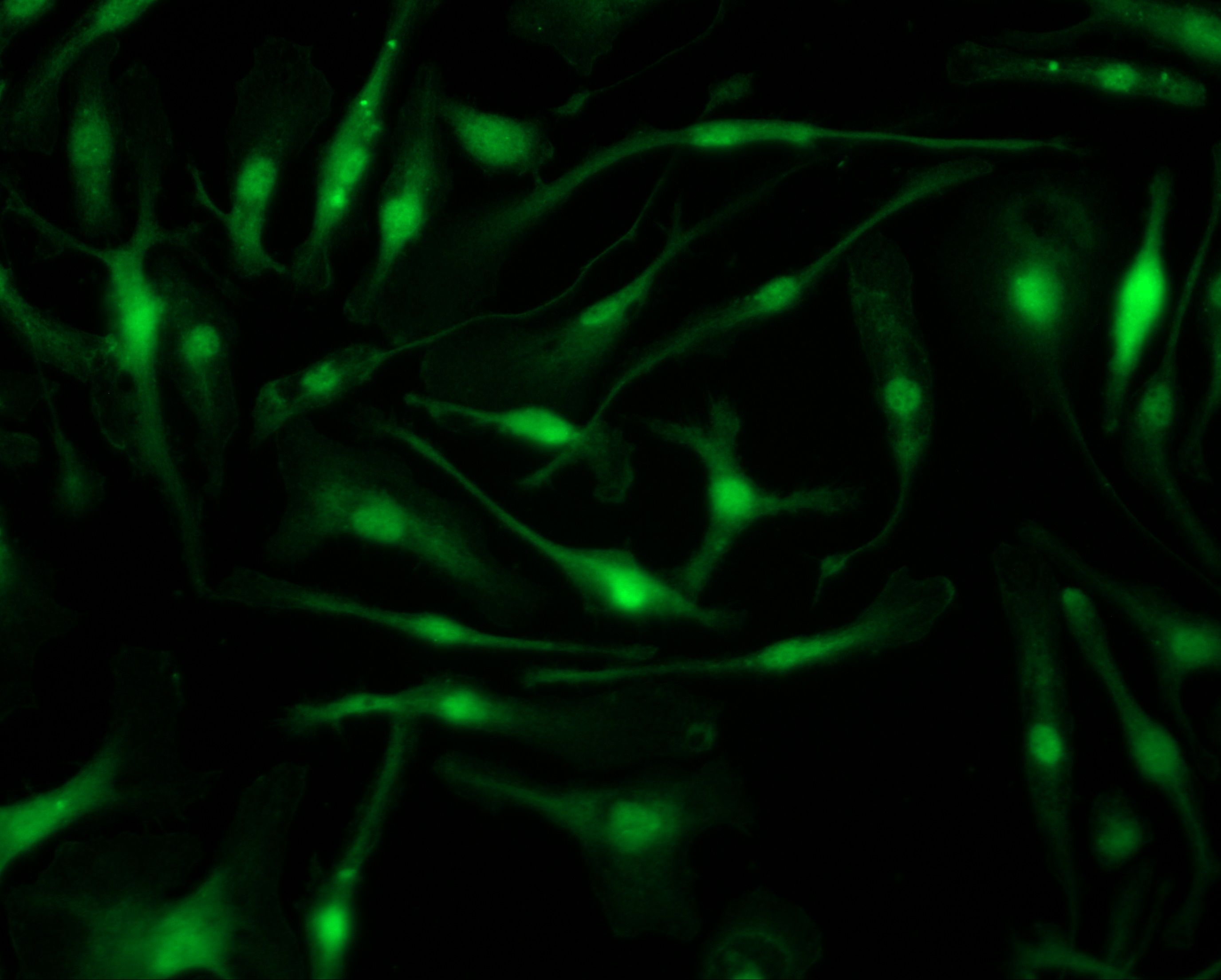

Supplement: Supplementary file 1 — Supplementary Information. [file 41598_2023_39765_MOESM1_ESM.zip › ╘¡╩╝╩2╛▌╒√└φ/cell immunofluorescence/inos/CONTROL.602/B2_c1.jpg]

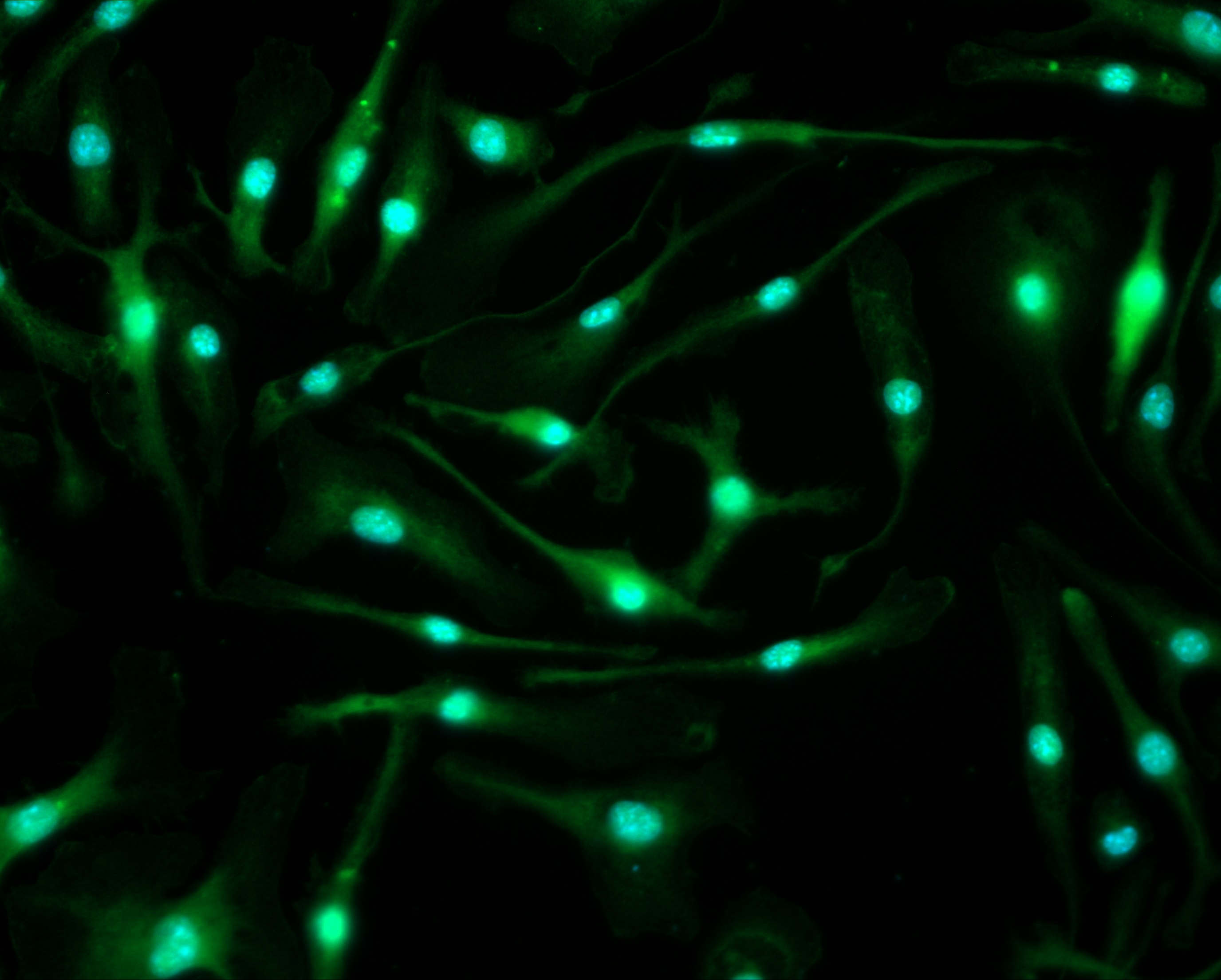

Supplement: Supplementary file 1 — Supplementary Information. [file 41598_2023_39765_MOESM1_ESM.zip › ╘¡╩╝╩2╛▌╒√└φ/cell immunofluorescence/inos/CONTROL.602/B2_c1+2.jpg]

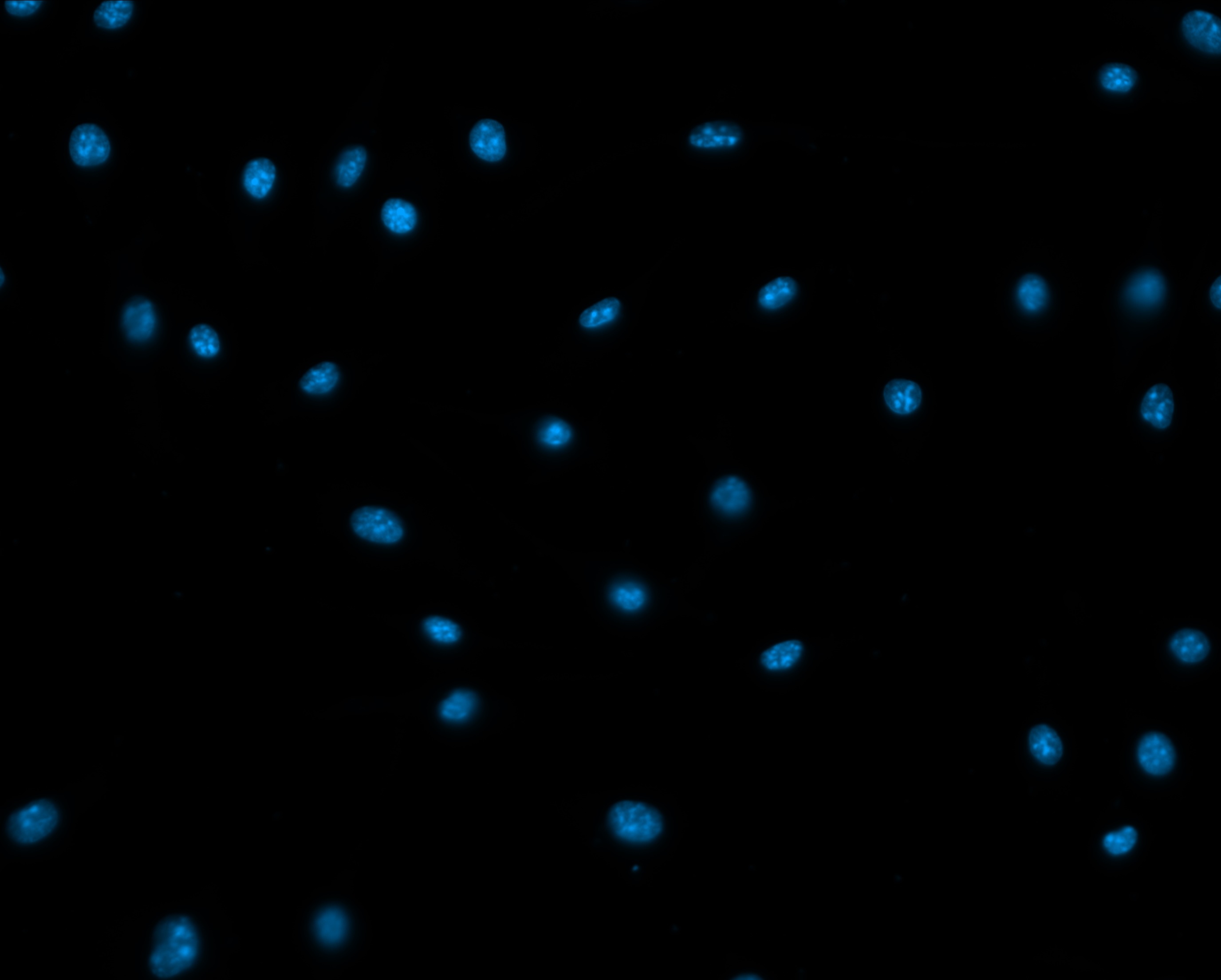

Supplement: Supplementary file 1 — Supplementary Information. [file 41598_2023_39765_MOESM1_ESM.zip › ╘¡╩╝╩2╛▌╒√└φ/cell immunofluorescence/inos/CONTROL.602/B2_c2.jpg]

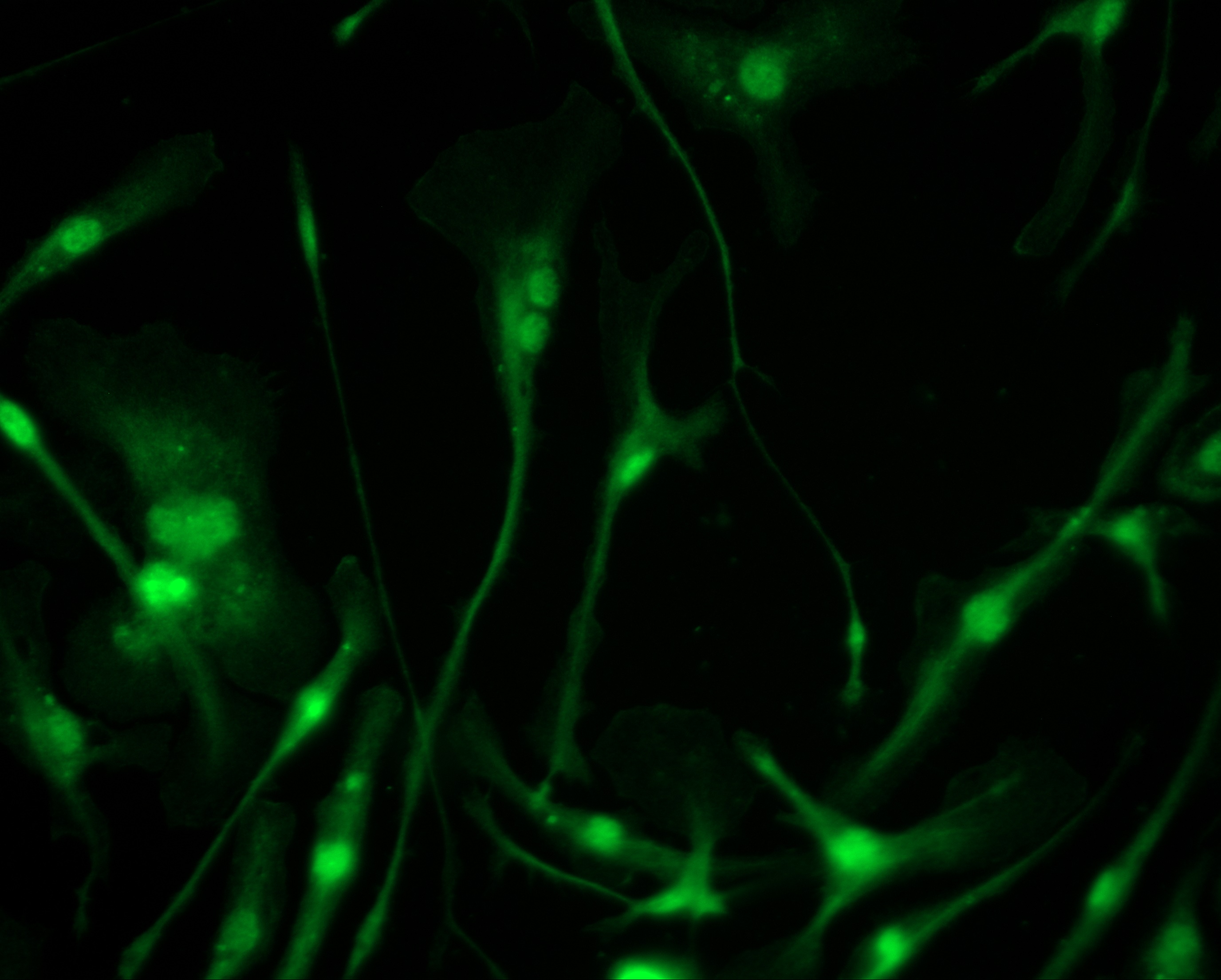

Supplement: Supplementary file 1 — Supplementary Information. [file 41598_2023_39765_MOESM1_ESM.zip › ╘¡╩╝╩2╛▌╒√└φ/cell immunofluorescence/inos/CONTROL.770/B1_c1.jpg]

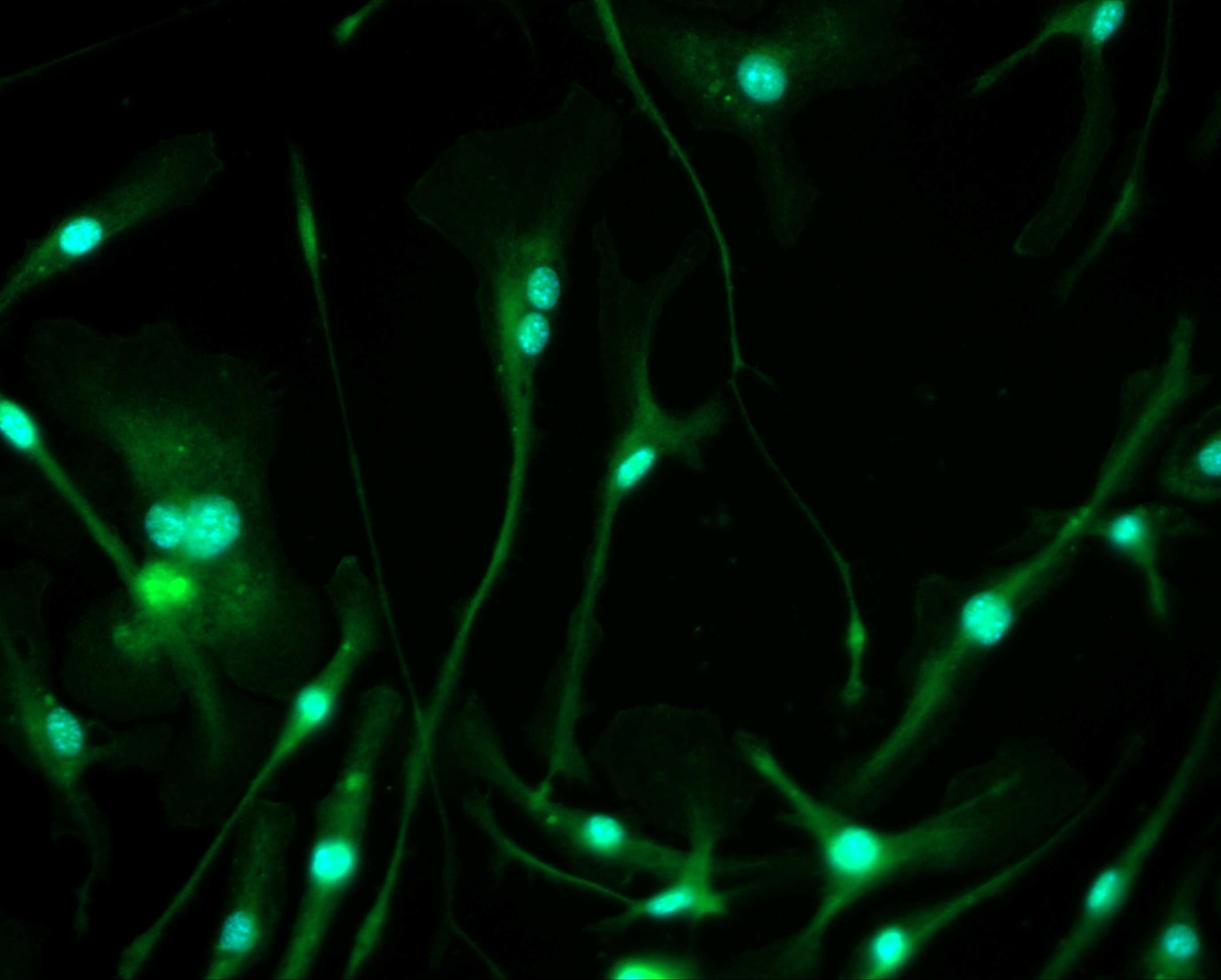

Supplement: Supplementary file 1 — Supplementary Information. [file 41598_2023_39765_MOESM1_ESM.zip › ╘¡╩╝╩2╛▌╒√└φ/cell immunofluorescence/inos/CONTROL.770/B1_c1+2.jpg]

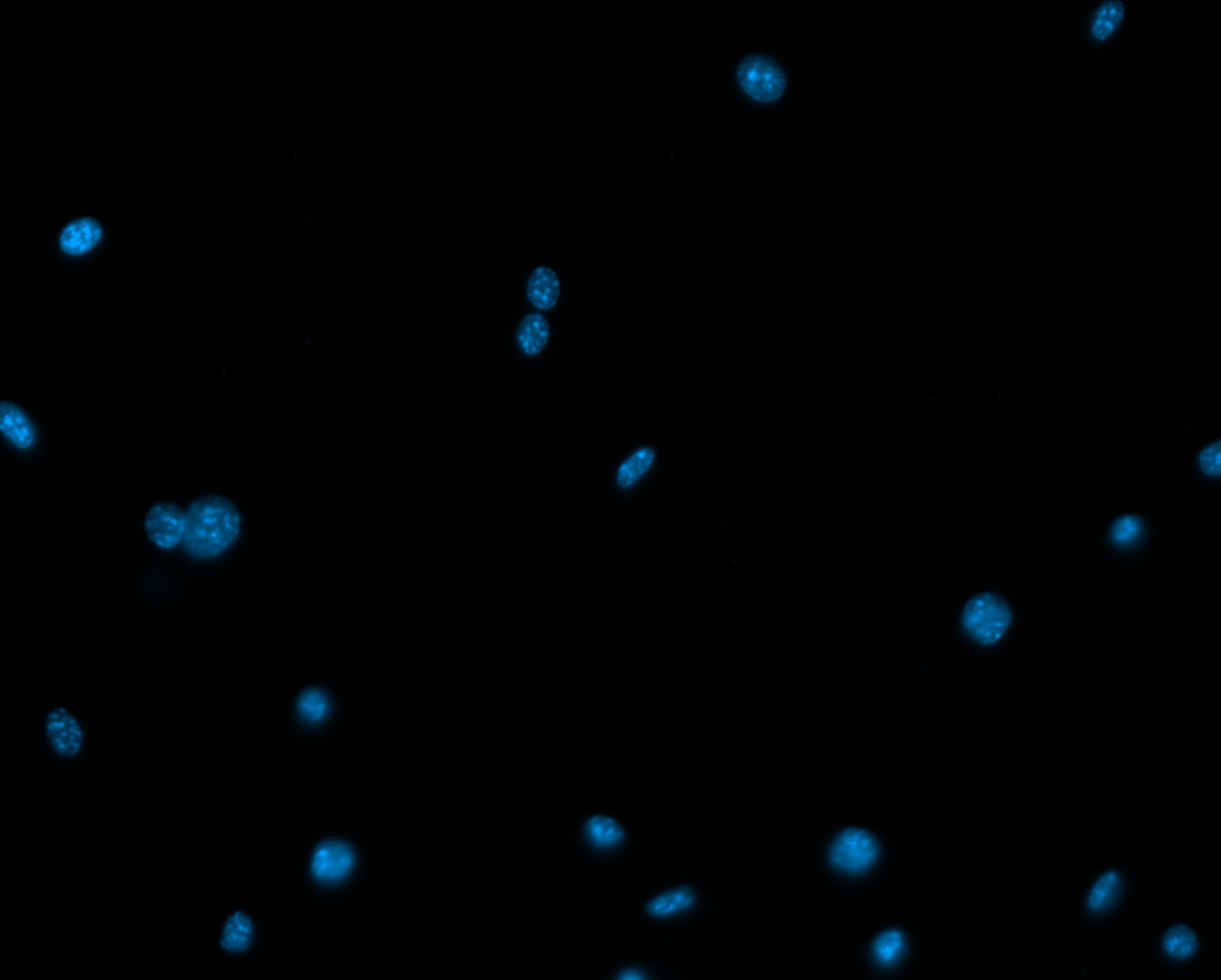

Supplement: Supplementary file 1 — Supplementary Information. [file 41598_2023_39765_MOESM1_ESM.zip › ╘¡╩╝╩2╛▌╒√└φ/cell immunofluorescence/inos/CONTROL.770/B1_c2.jpg]

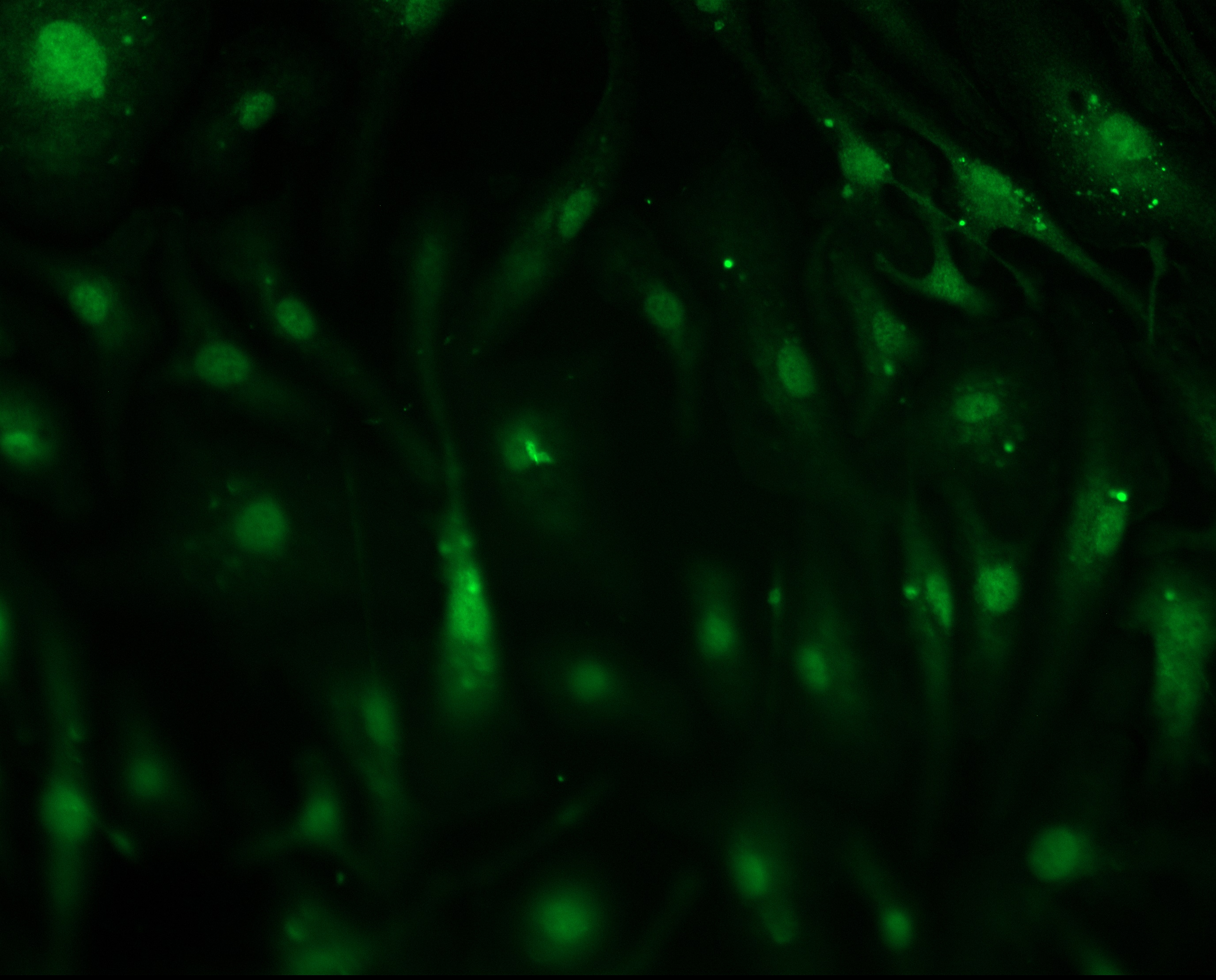

Supplement: Supplementary file 1 — Supplementary Information. [file 41598_2023_39765_MOESM1_ESM.zip › ╘¡╩╝╩2╛▌╒√└φ/cell immunofluorescence/inos/KERATINASE.140/J4_c1.jpg]

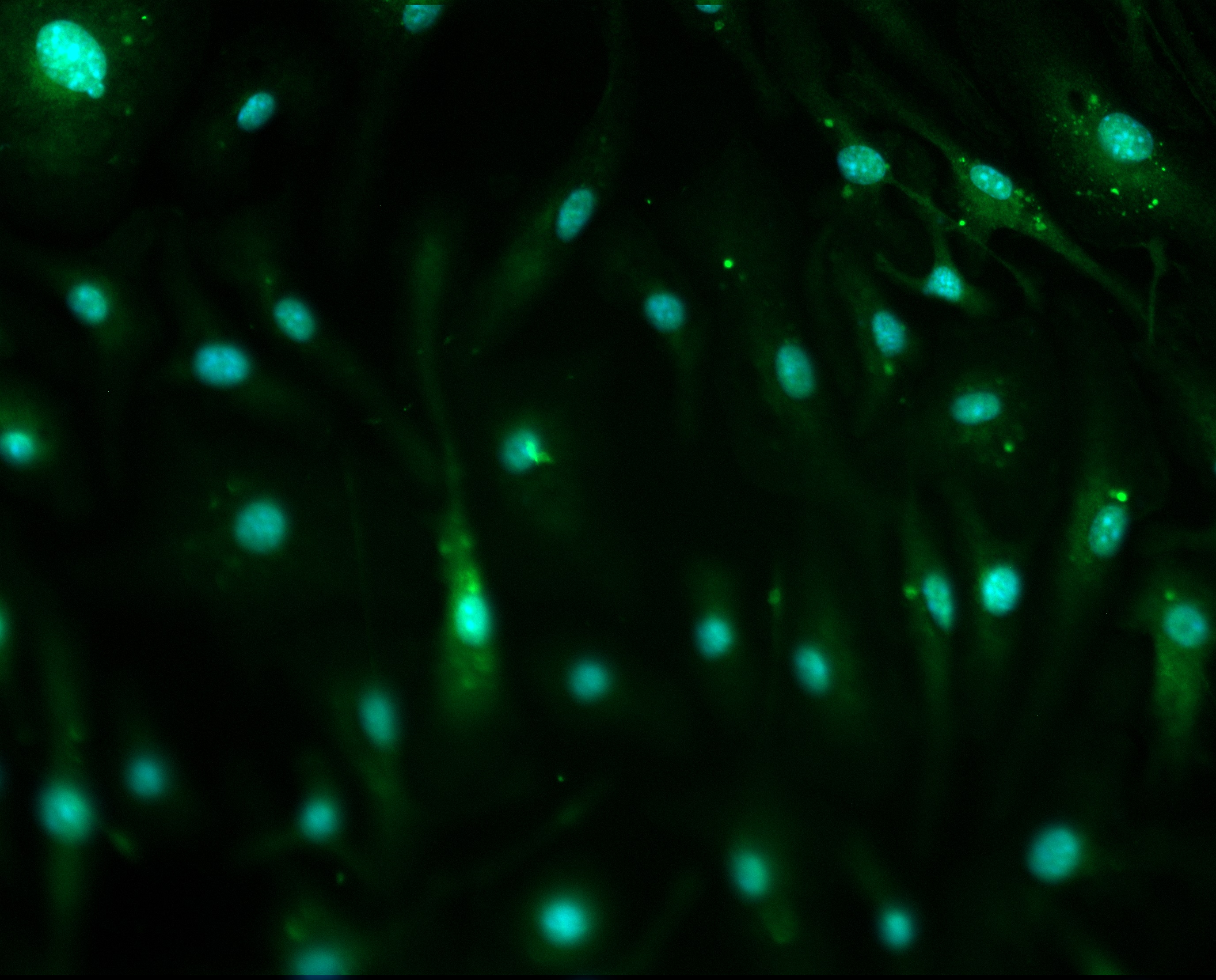

Supplement: Supplementary file 1 — Supplementary Information. [file 41598_2023_39765_MOESM1_ESM.zip › ╘¡╩╝╩2╛▌╒√└φ/cell immunofluorescence/inos/KERATINASE.140/J4_c1+2.jpg]

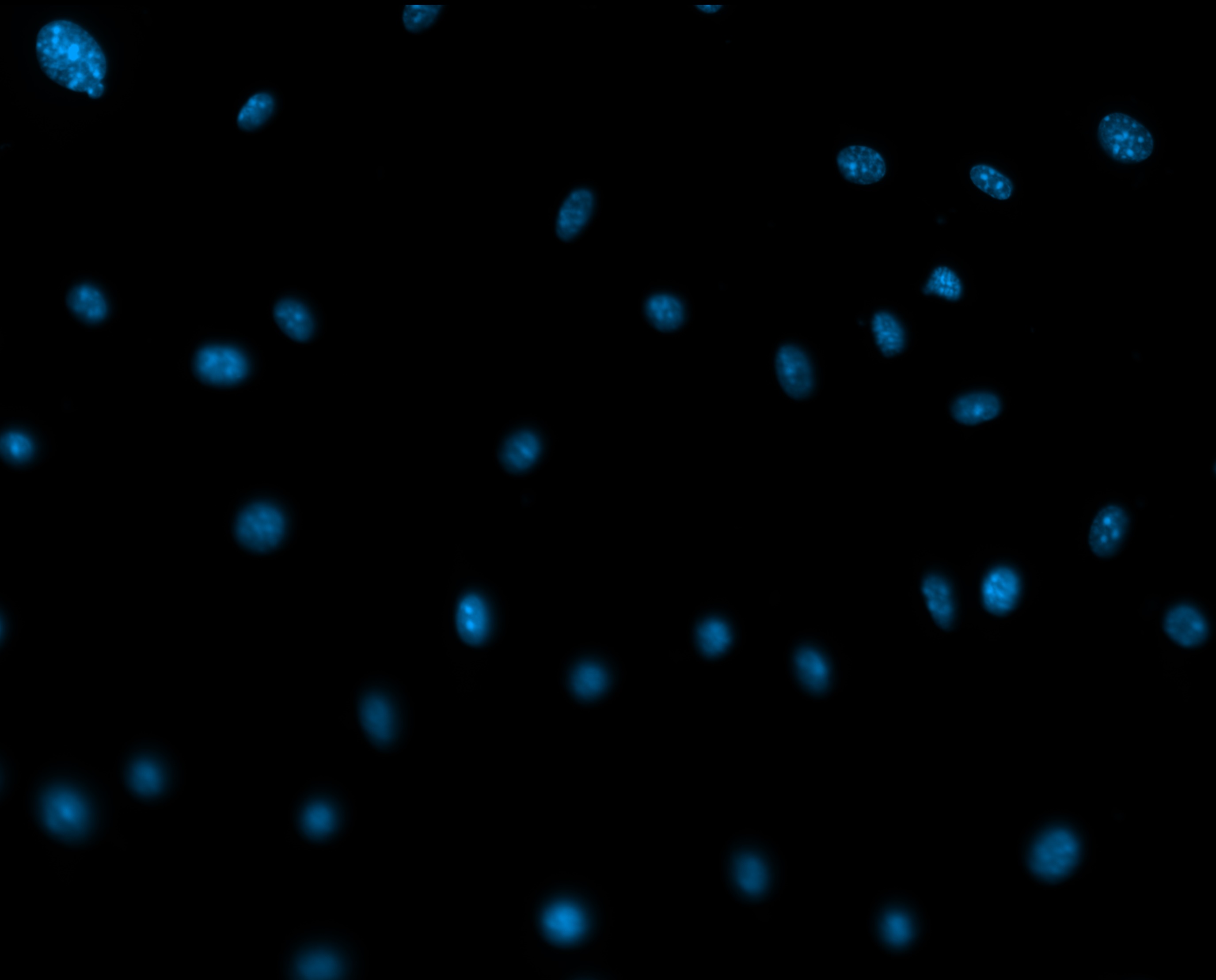

Supplement: Supplementary file 1 — Supplementary Information. [file 41598_2023_39765_MOESM1_ESM.zip › ╘¡╩╝╩2╛▌╒√└φ/cell immunofluorescence/inos/KERATINASE.140/J4_c2.jpg]

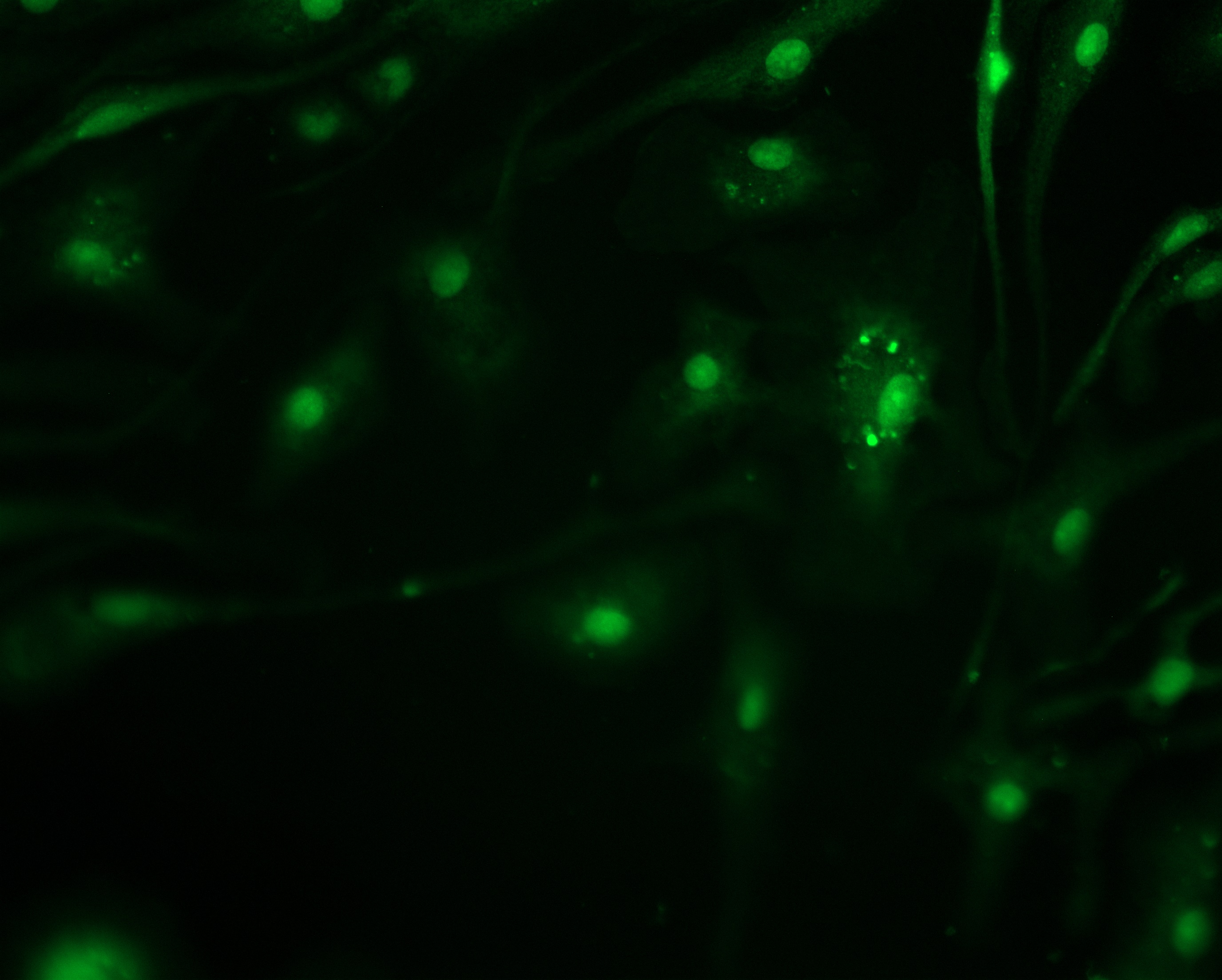

Supplement: Supplementary file 1 — Supplementary Information. [file 41598_2023_39765_MOESM1_ESM.zip › ╘¡╩╝╩2╛▌╒√└φ/cell immunofluorescence/inos/KERATINASE.187/J6_c1.jpg]

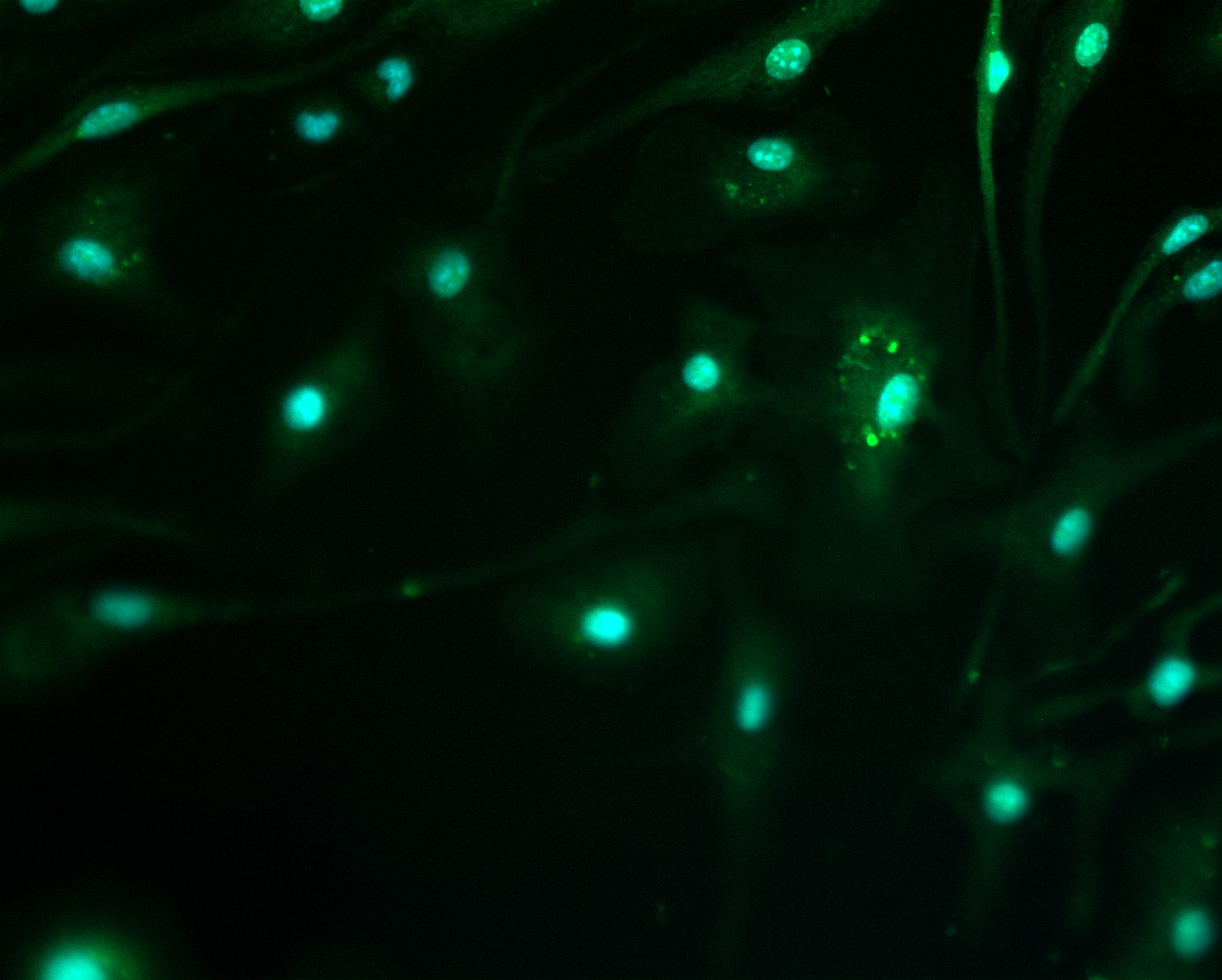

Supplement: Supplementary file 1 — Supplementary Information. [file 41598_2023_39765_MOESM1_ESM.zip › ╘¡╩╝╩2╛▌╒√└φ/cell immunofluorescence/inos/KERATINASE.187/J6_c1+2.jpg]

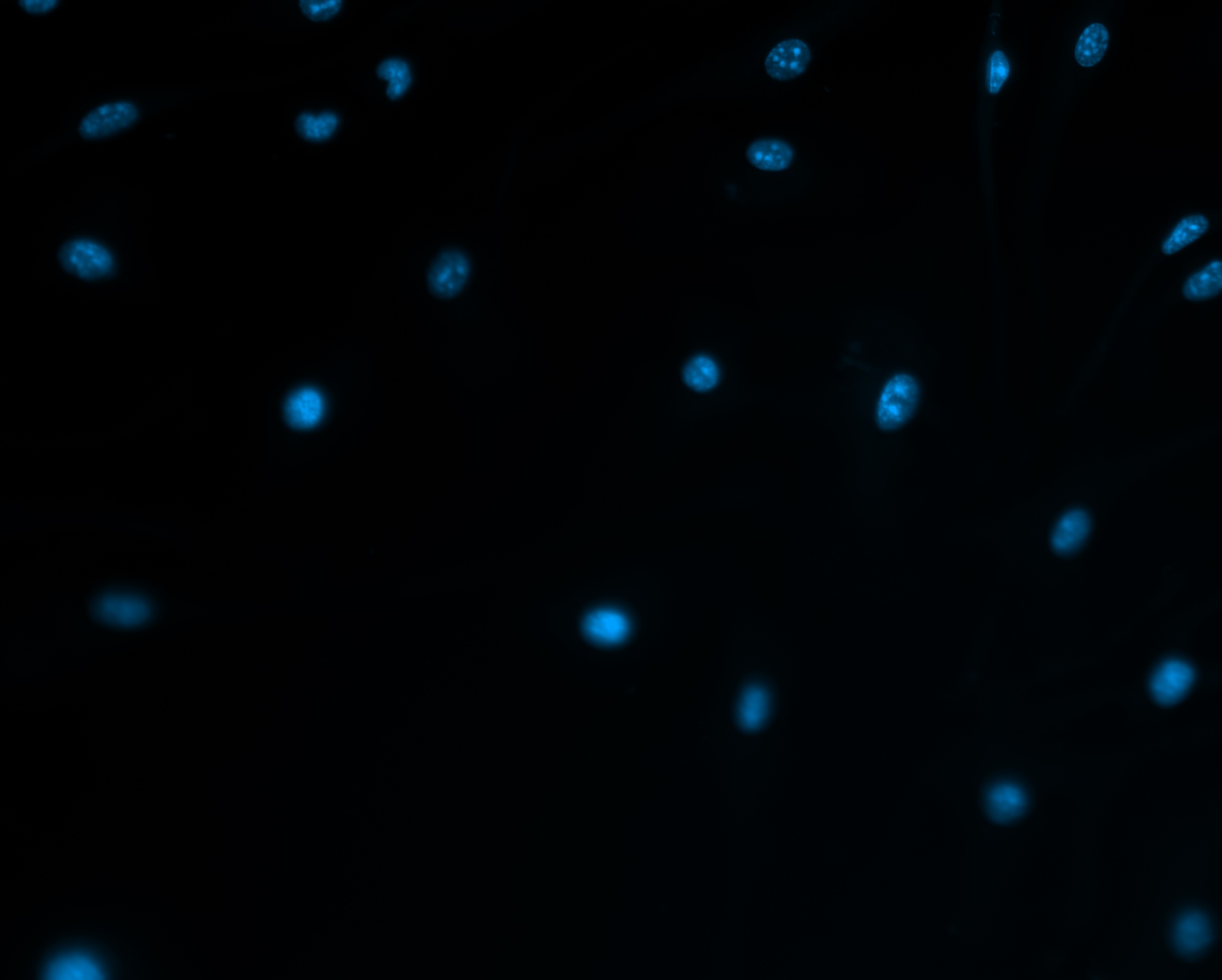

Supplement: Supplementary file 1 — Supplementary Information. [file 41598_2023_39765_MOESM1_ESM.zip › ╘¡╩╝╩2╛▌╒√└φ/cell immunofluorescence/inos/KERATINASE.187/J6_c2.jpg]

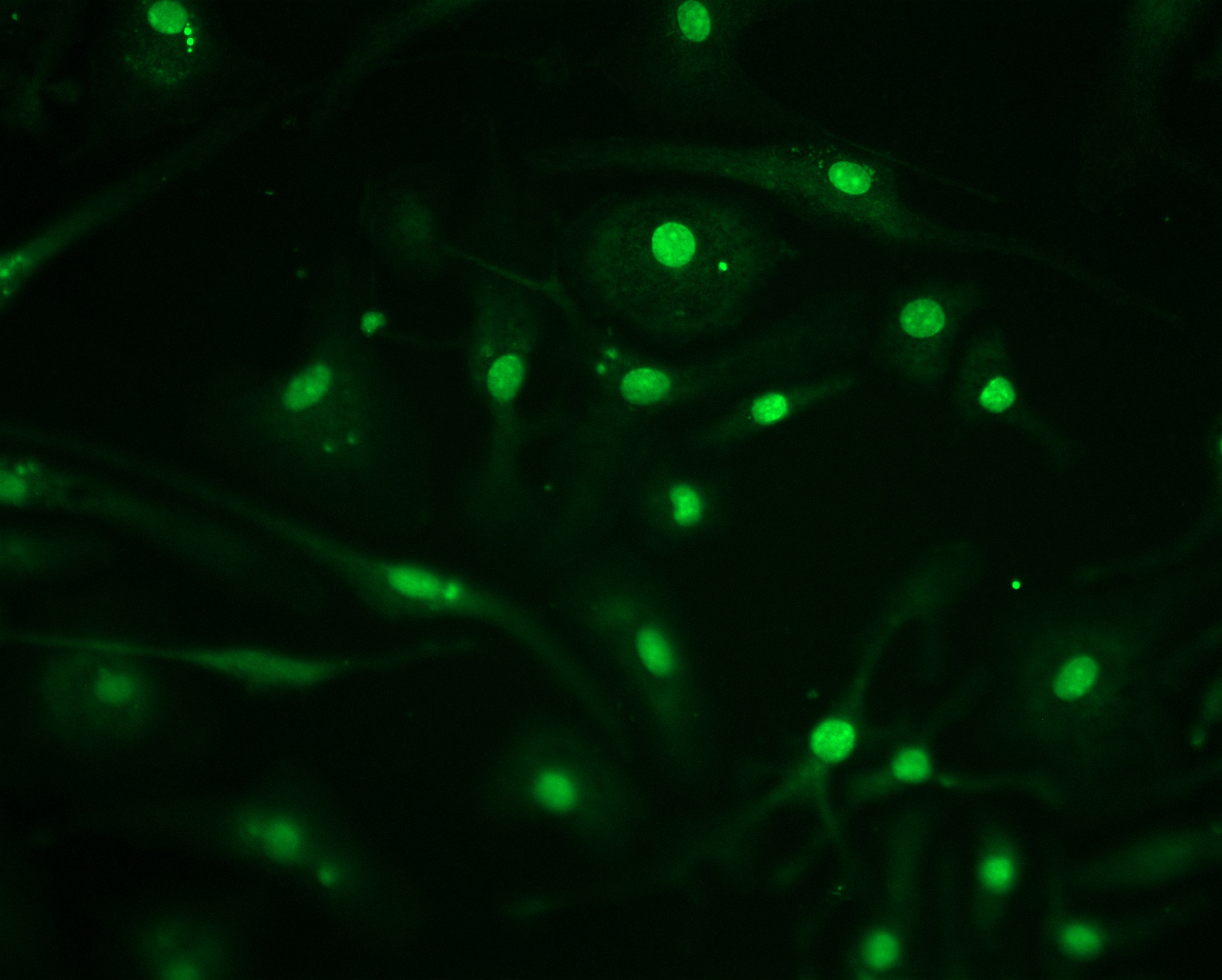

Supplement: Supplementary file 1 — Supplementary Information. [file 41598_2023_39765_MOESM1_ESM.zip › ╘¡╩╝╩2╛▌╒√└φ/cell immunofluorescence/inos/KERATINASE.745/J5_c1.jpg]

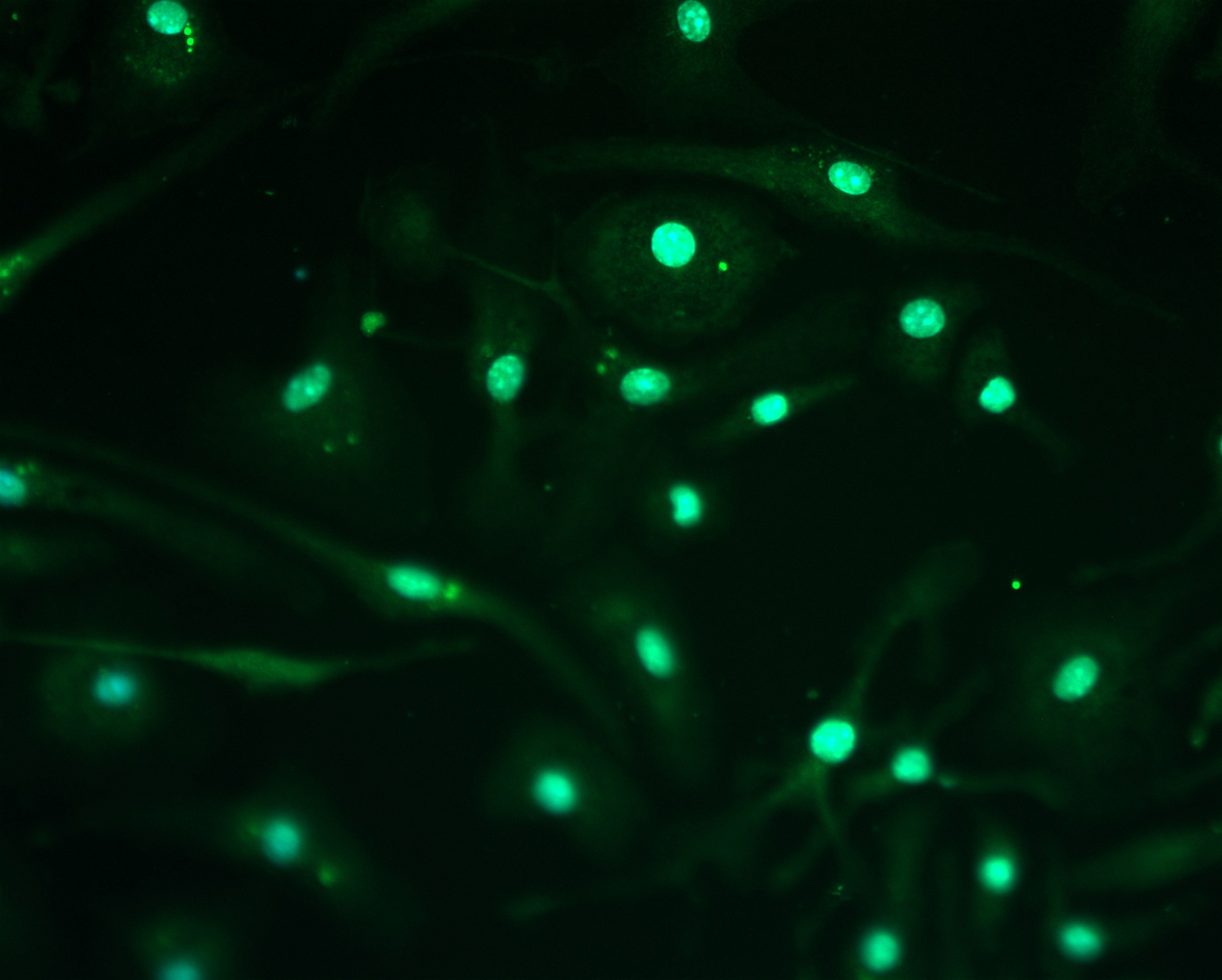

Supplement: Supplementary file 1 — Supplementary Information. [file 41598_2023_39765_MOESM1_ESM.zip › ╘¡╩╝╩2╛▌╒√└φ/cell immunofluorescence/inos/KERATINASE.745/J5_c1+2.jpg]

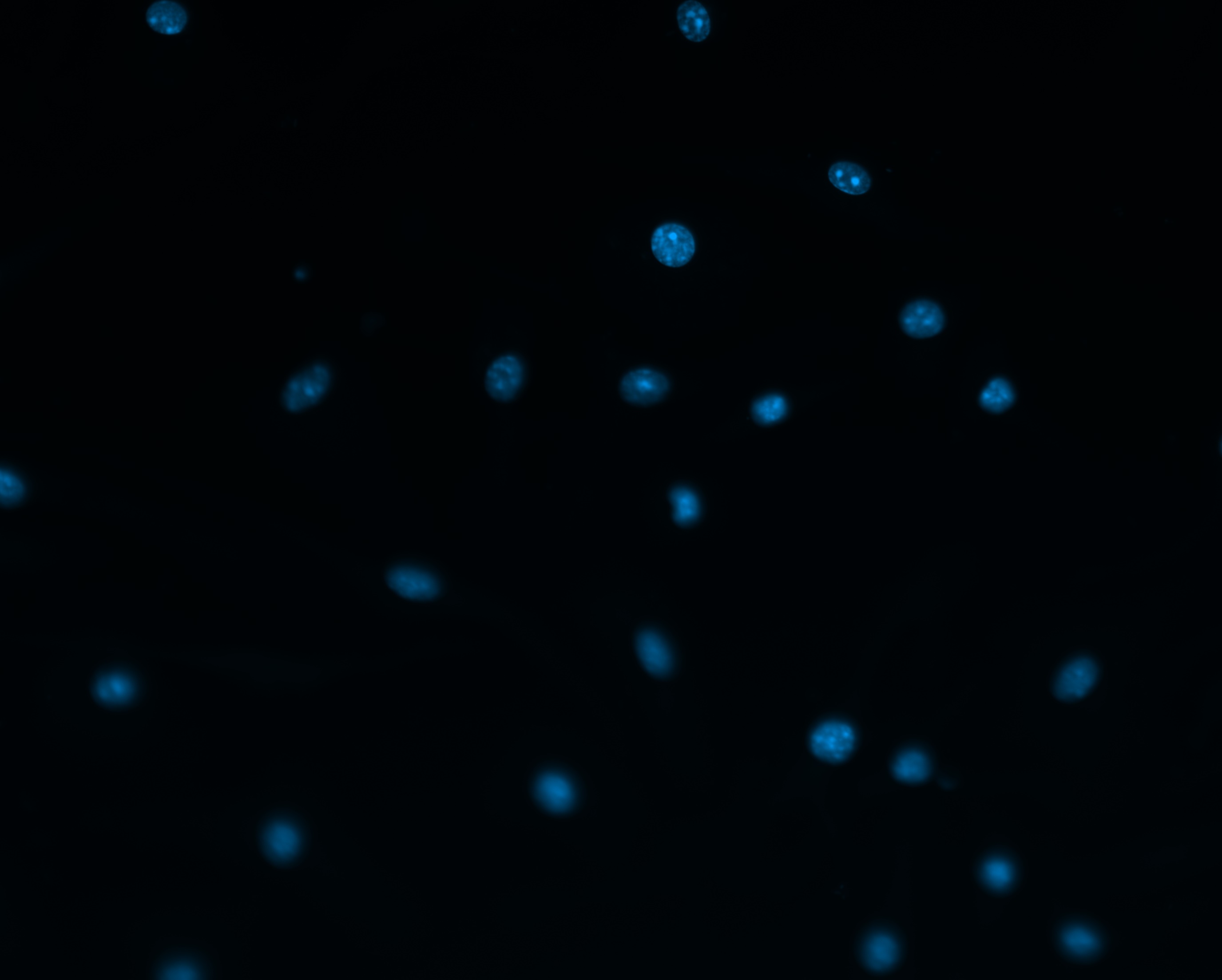

Supplement: Supplementary file 1 — Supplementary Information. [file 41598_2023_39765_MOESM1_ESM.zip › ╘¡╩╝╩2╛▌╒√└φ/cell immunofluorescence/inos/KERATINASE.745/J5_c2.jpg]

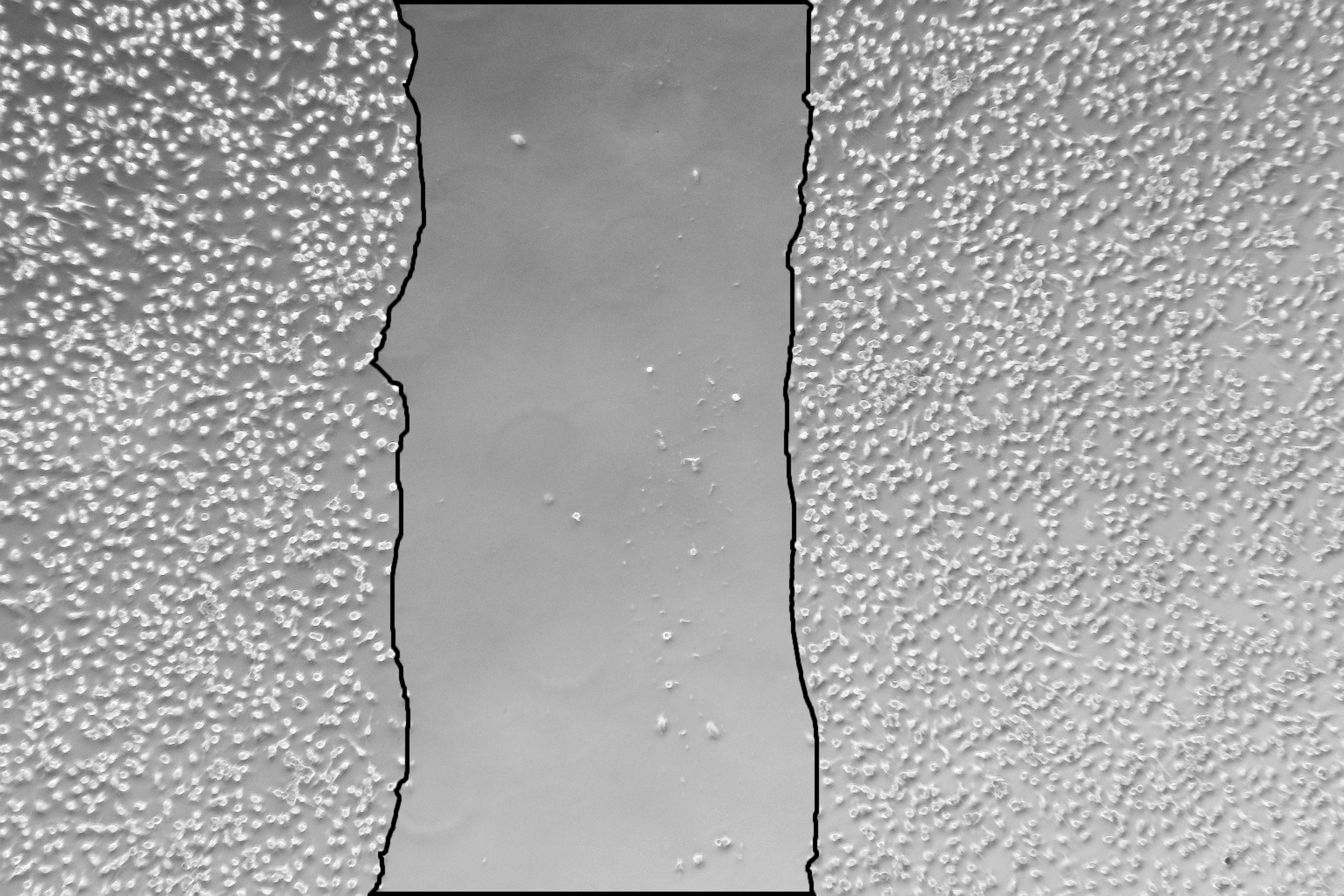

Supplement: Supplementary file 1 — Supplementary Information. [file 41598_2023_39765_MOESM1_ESM.zip › ╘¡╩╝╩2╛▌╒√└φ/scratch/B1 0.png]

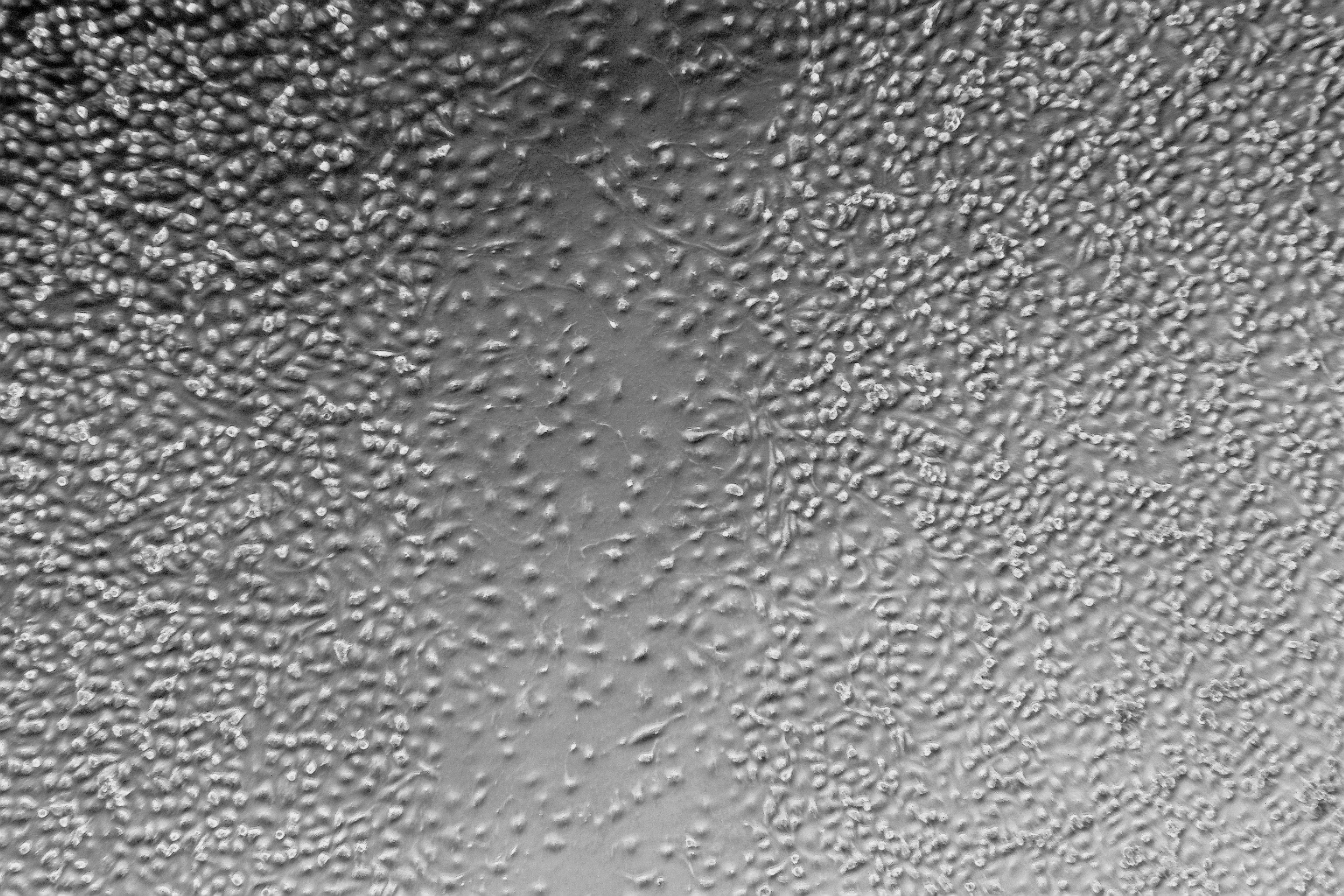

Supplement: Supplementary file 1 — Supplementary Information. [file 41598_2023_39765_MOESM1_ESM.zip › ╘¡╩╝╩2╛▌╒√└φ/scratch/B1 12.png]

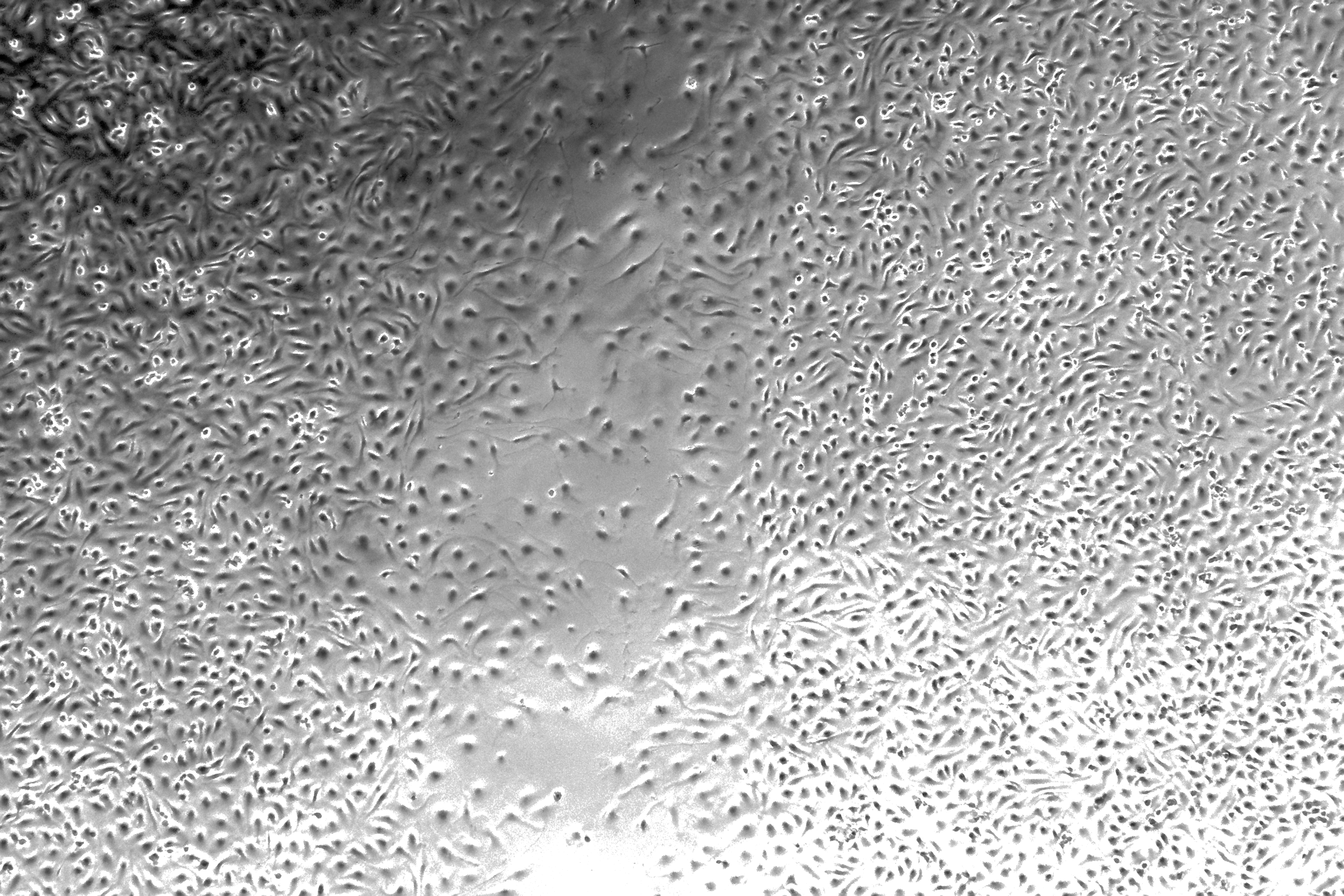

Supplement: Supplementary file 1 — Supplementary Information. [file 41598_2023_39765_MOESM1_ESM.zip › ╘¡╩╝╩2╛▌╒√└φ/scratch/B1 6.png]

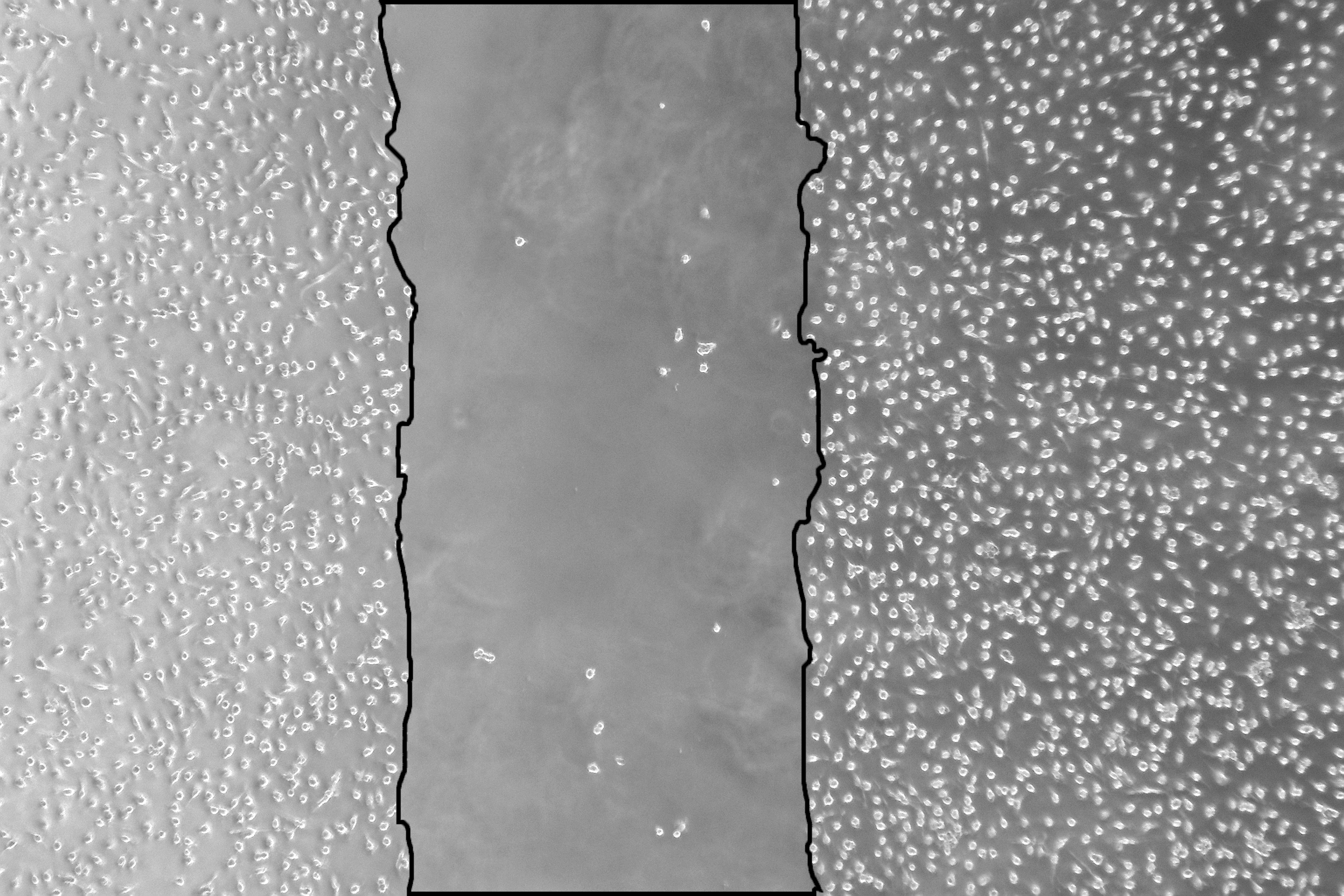

Supplement: Supplementary file 1 — Supplementary Information. [file 41598_2023_39765_MOESM1_ESM.zip › ╘¡╩╝╩2╛▌╒√└φ/scratch/C1 0 1886967.png]

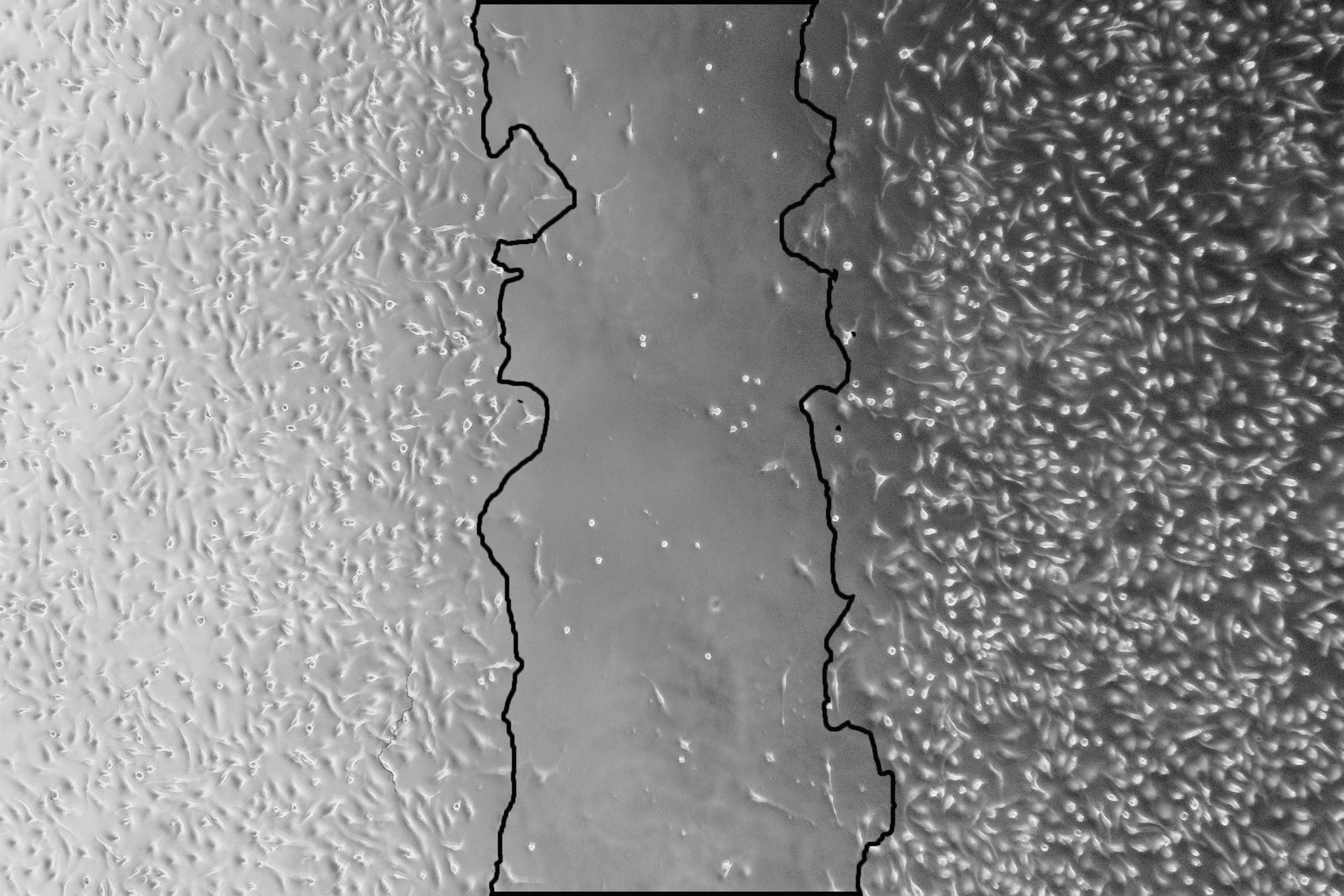

Supplement: Supplementary file 1 — Supplementary Information. [file 41598_2023_39765_MOESM1_ESM.zip › ╘¡╩╝╩2╛▌╒√└φ/scratch/C1 12 1516971.png]

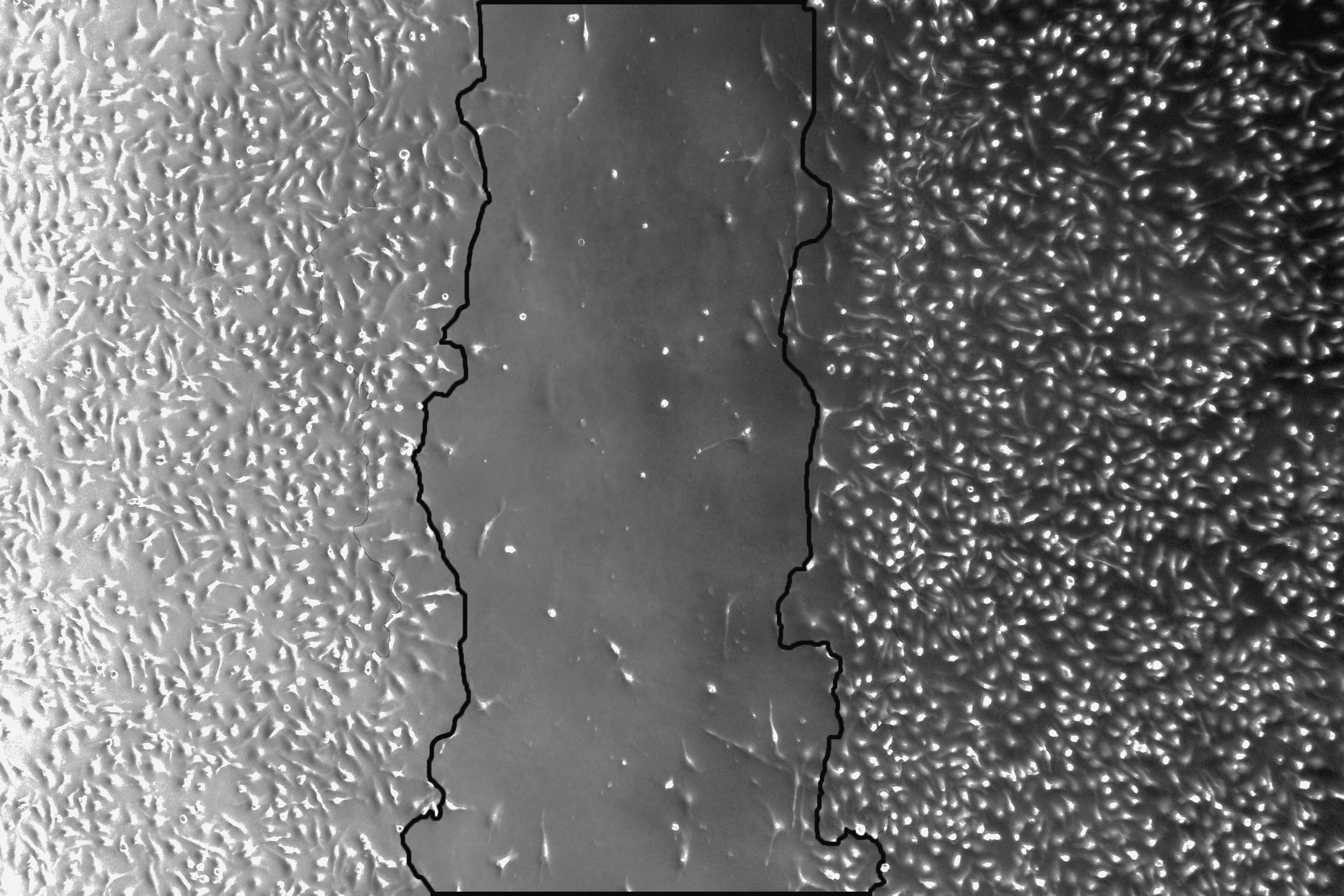

Supplement: Supplementary file 1 — Supplementary Information. [file 41598_2023_39765_MOESM1_ESM.zip › ╘¡╩╝╩2╛▌╒√└φ/scratch/C1 6 1639878.png]

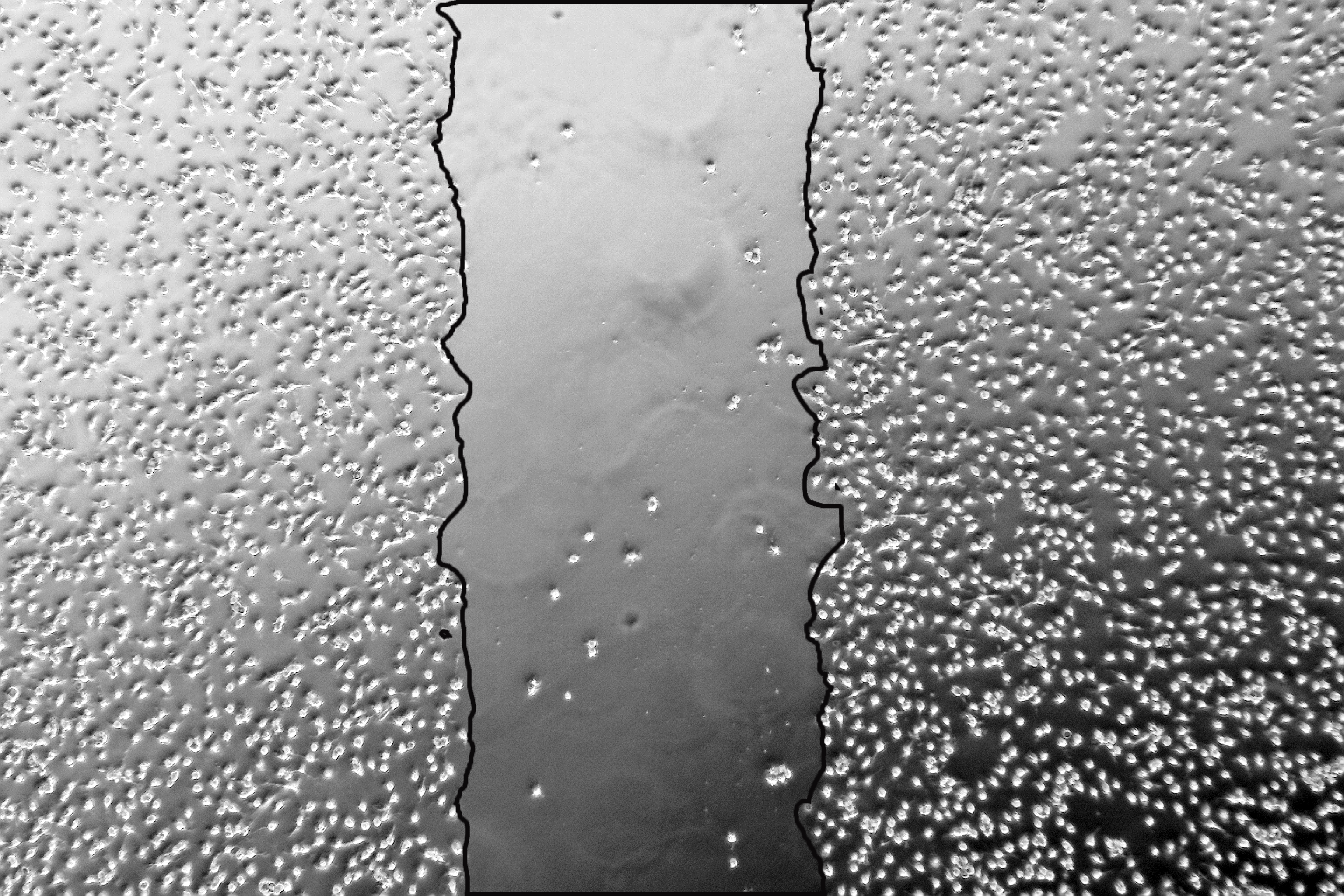

Supplement: Supplementary file 1 — Supplementary Information. [file 41598_2023_39765_MOESM1_ESM.zip › ╘¡╩╝╩2╛▌╒√└φ/scratch/J2 0 1664235.png]

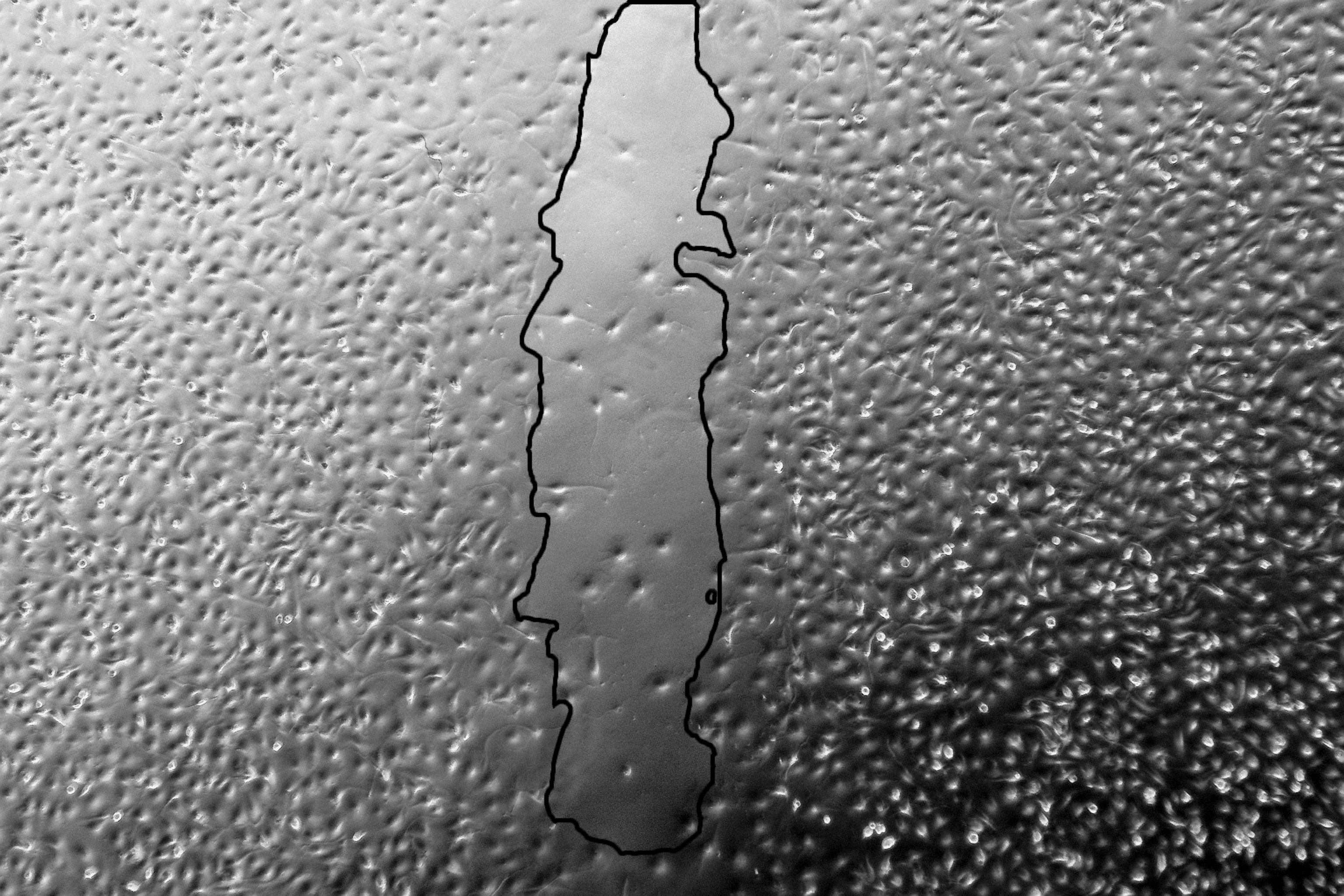

Supplement: Supplementary file 1 — Supplementary Information. [file 41598_2023_39765_MOESM1_ESM.zip › ╘¡╩╝╩2╛▌╒√└φ/scratch/J2 12 685813.png]

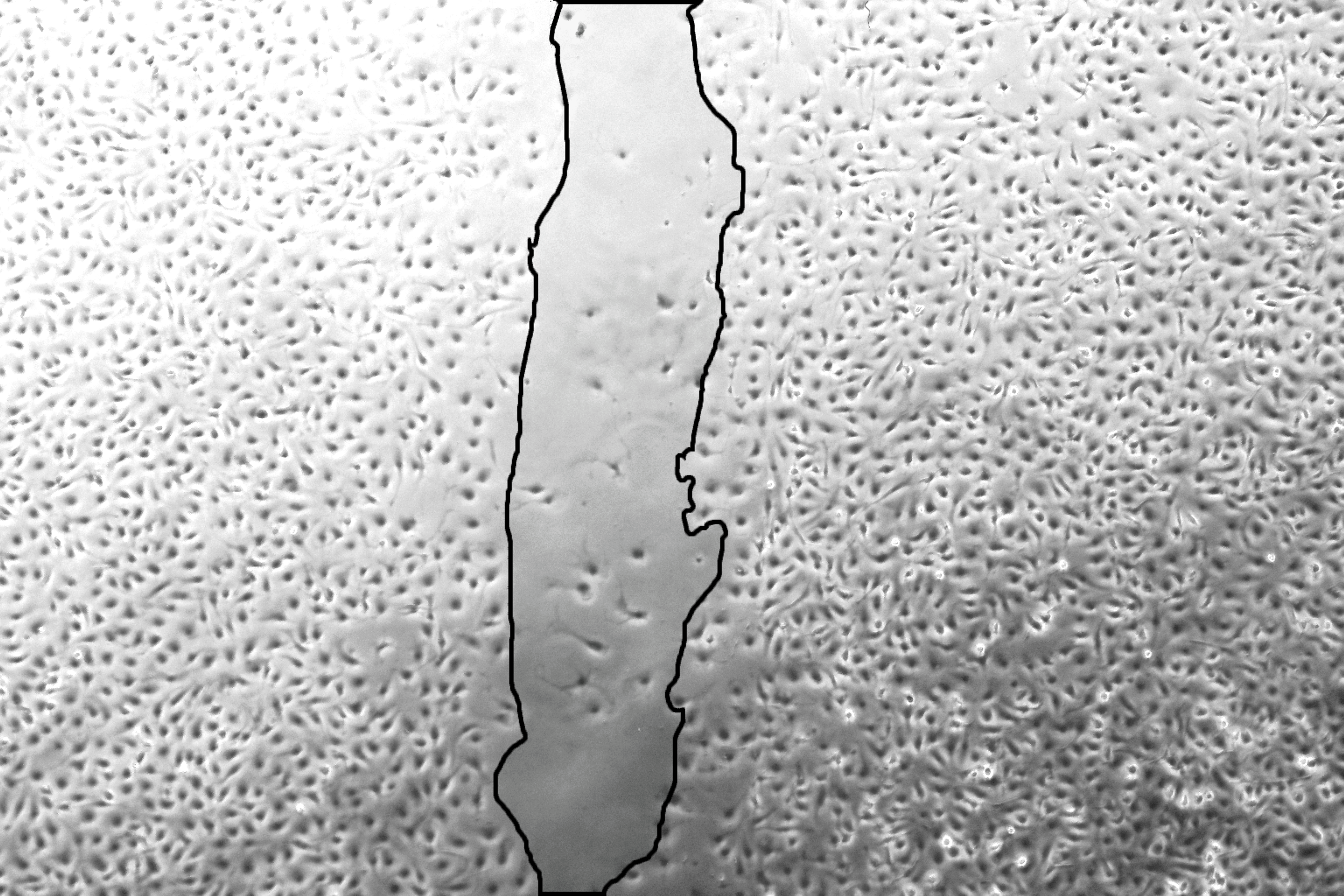

Supplement: Supplementary file 1 — Supplementary Information. [file 41598_2023_39765_MOESM1_ESM.zip › ╘¡╩╝╩2╛▌╒√└φ/scratch/J2 6 788619.png]

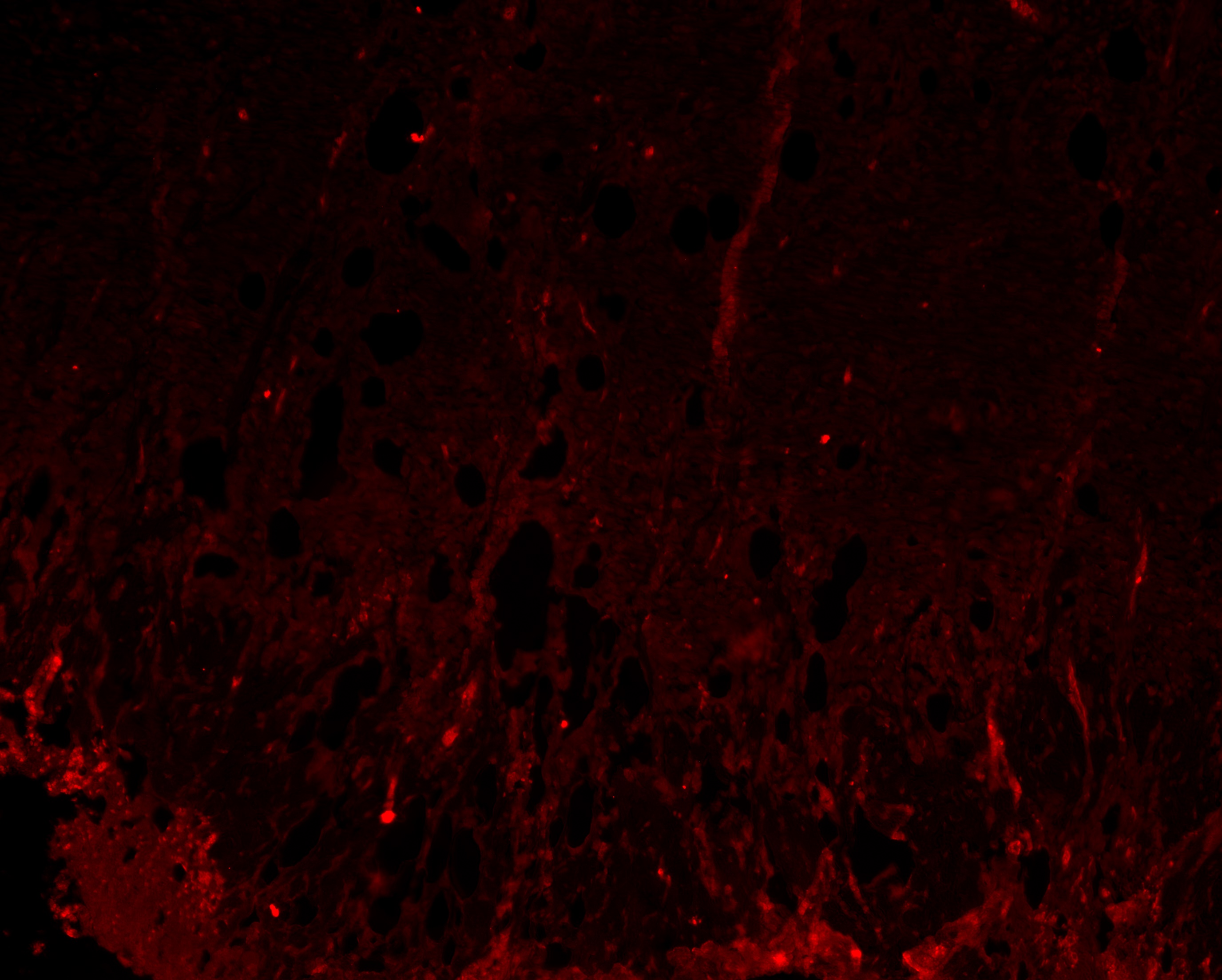

Supplement: Supplementary file 1 — Supplementary Information. [file 41598_2023_39765_MOESM1_ESM.zip › ╘¡╩╝╩2╛▌╒√└φ/tissue immunofluorescence/cd68/control (2)/Snap-4102_c1.jpg]

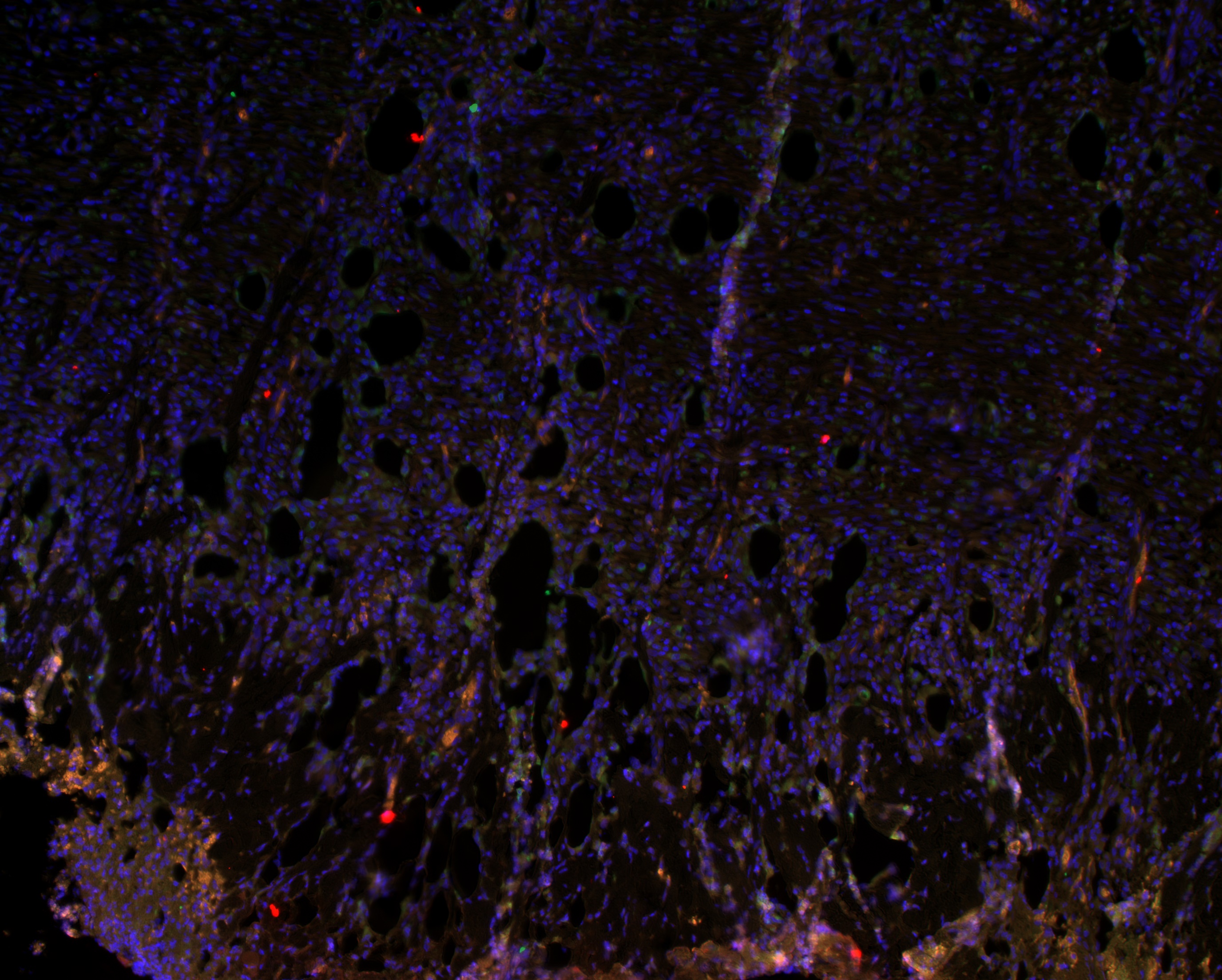

Supplement: Supplementary file 1 — Supplementary Information. [file 41598_2023_39765_MOESM1_ESM.zip › ╘¡╩╝╩2╛▌╒√└φ/tissue immunofluorescence/cd68/control (2)/Snap-4102_c1+2+3.jpg]

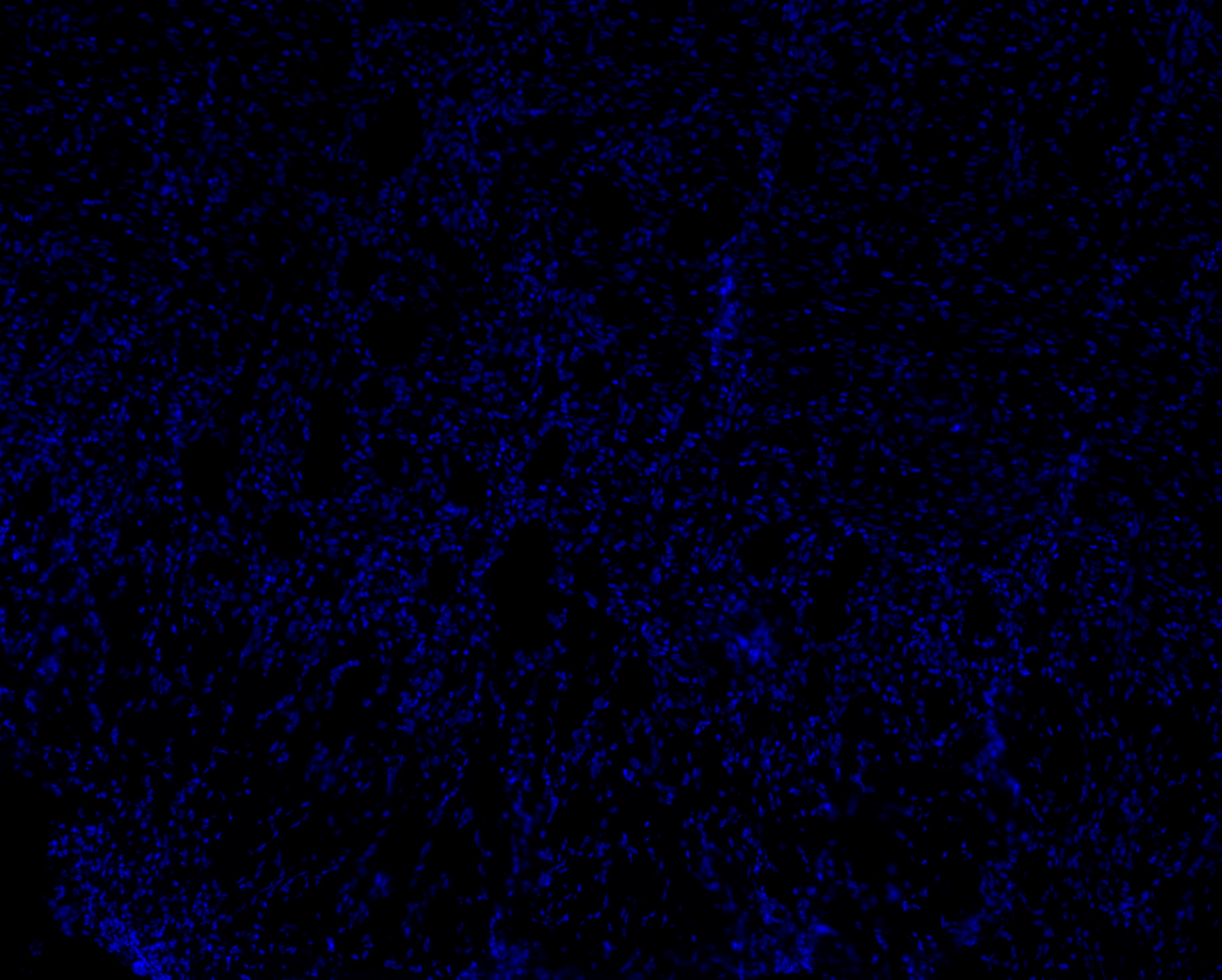

Supplement: Supplementary file 1 — Supplementary Information. [file 41598_2023_39765_MOESM1_ESM.zip › ╘¡╩╝╩2╛▌╒√└φ/tissue immunofluorescence/cd68/control (2)/Snap-4102_c2.jpg]

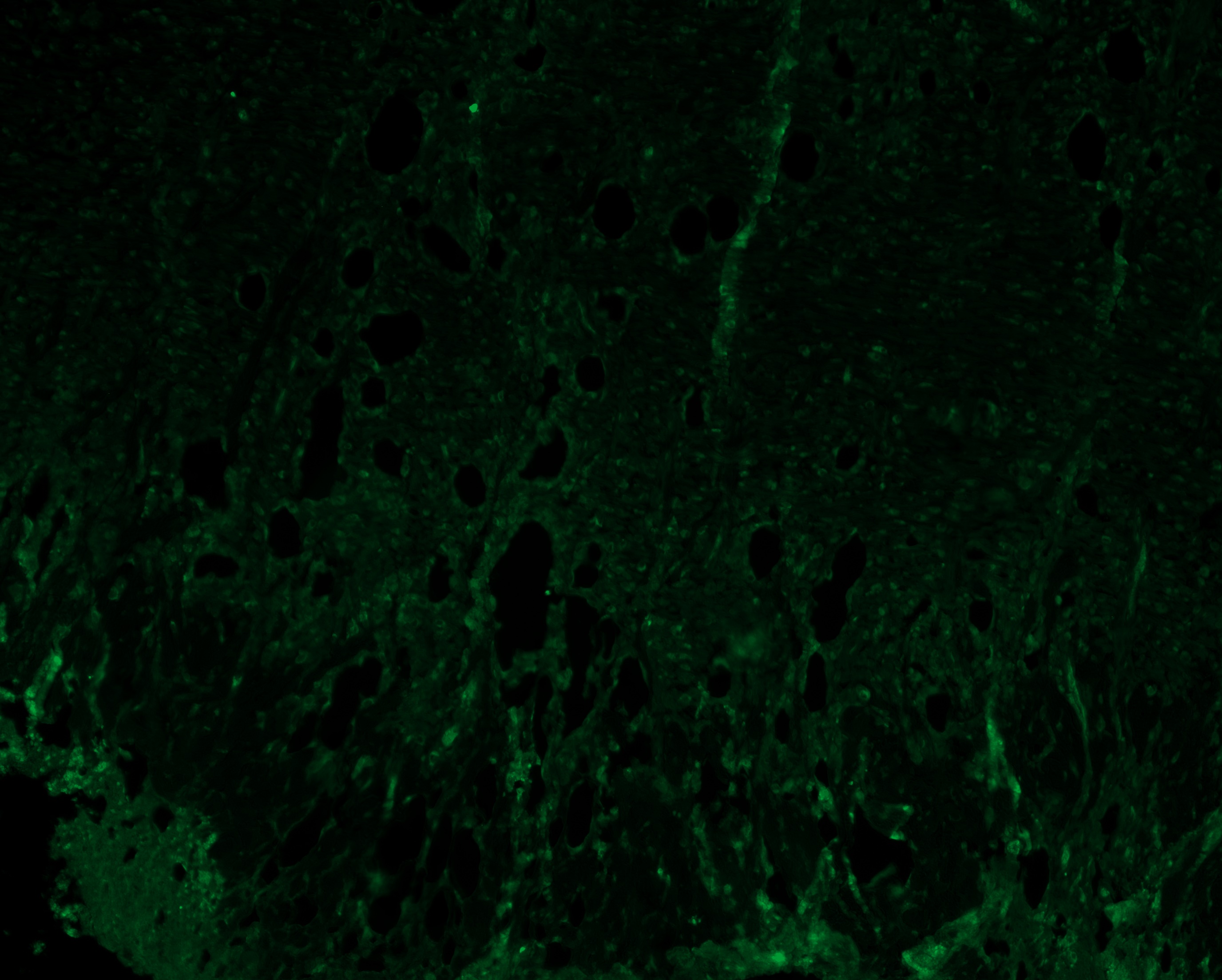

Supplement: Supplementary file 1 — Supplementary Information. [file 41598_2023_39765_MOESM1_ESM.zip › ╘¡╩╝╩2╛▌╒√└φ/tissue immunofluorescence/cd68/control (2)/Snap-4102_c3.jpg]

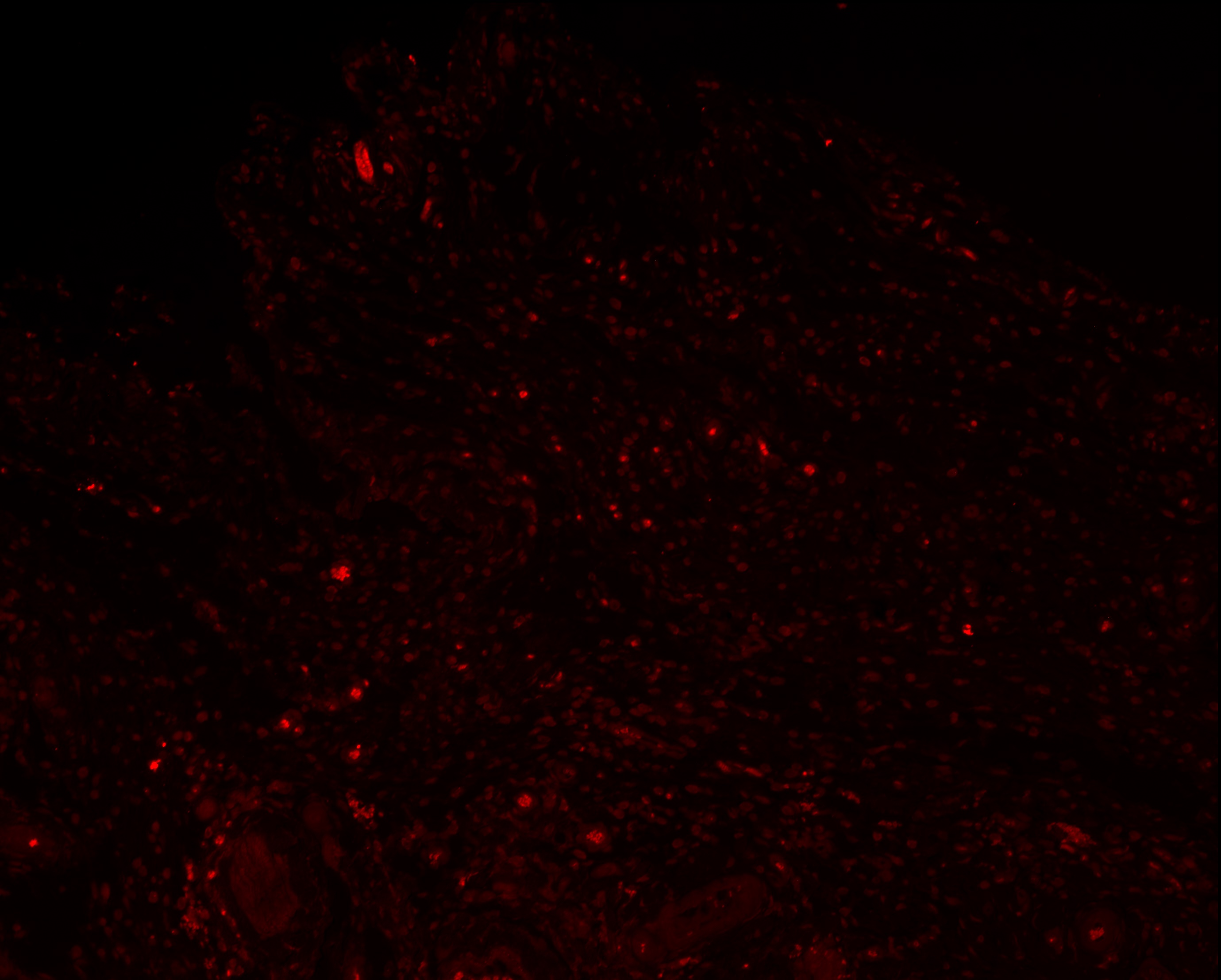

Supplement: Supplementary file 1 — Supplementary Information. [file 41598_2023_39765_MOESM1_ESM.zip › ╘¡╩╝╩2╛▌╒√└φ/tissue immunofluorescence/cd68/control (3)/Snap-4096_c1.jpg]

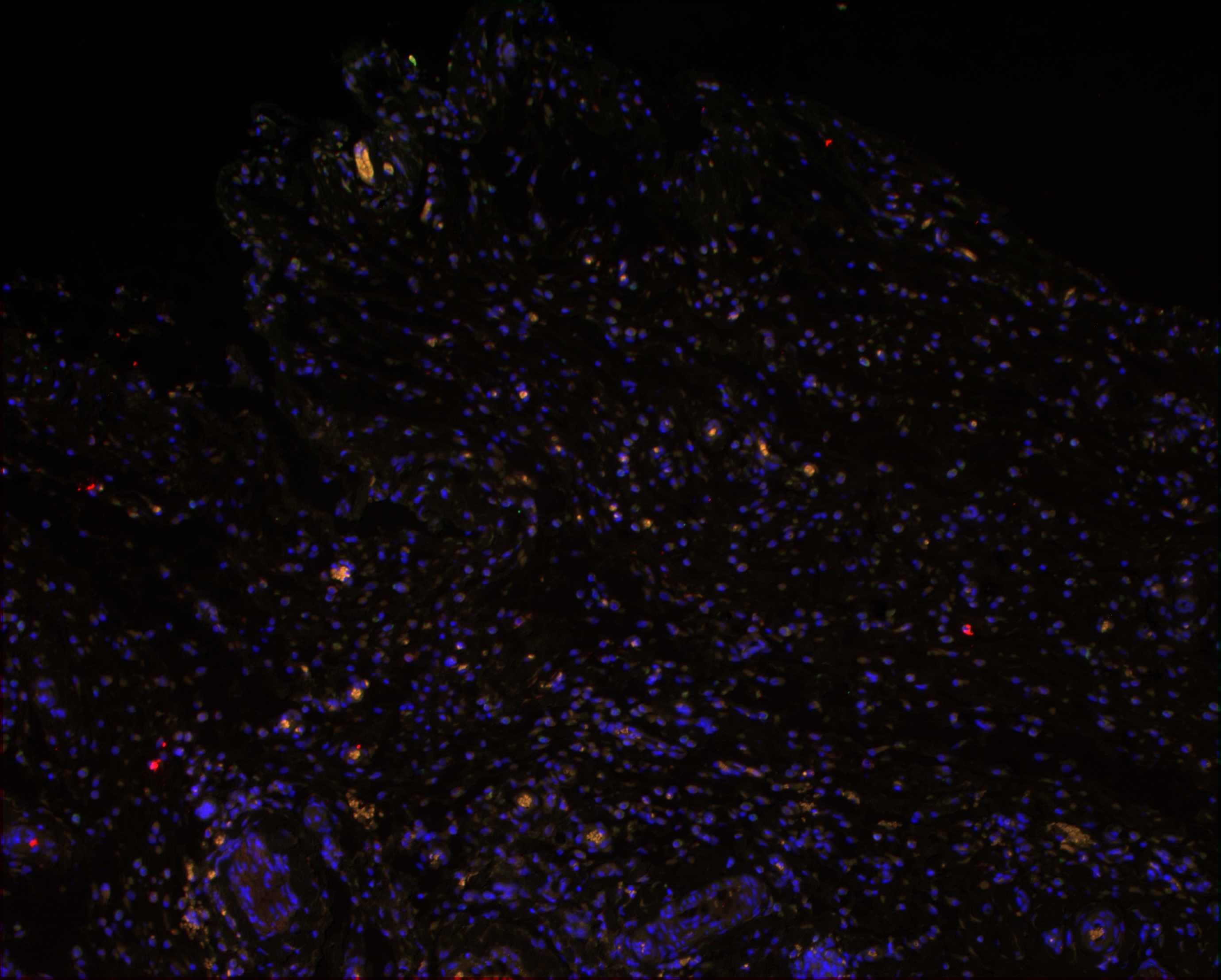

Supplement: Supplementary file 1 — Supplementary Information. [file 41598_2023_39765_MOESM1_ESM.zip › ╘¡╩╝╩2╛▌╒√└φ/tissue immunofluorescence/cd68/control (3)/Snap-4096_c1+2+3.jpg]

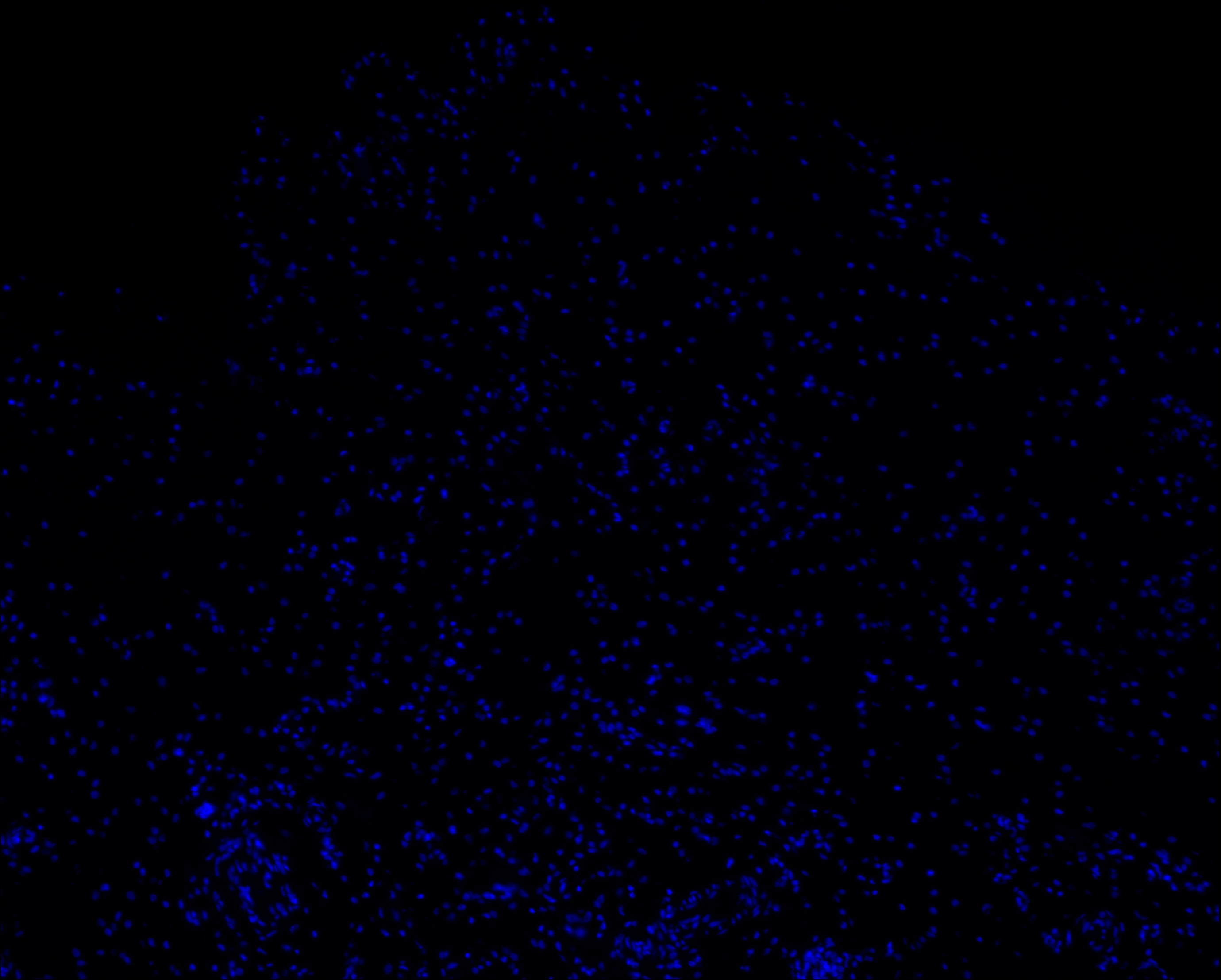

Supplement: Supplementary file 1 — Supplementary Information. [file 41598_2023_39765_MOESM1_ESM.zip › ╘¡╩╝╩2╛▌╒√└φ/tissue immunofluorescence/cd68/control (3)/Snap-4096_c2.jpg]

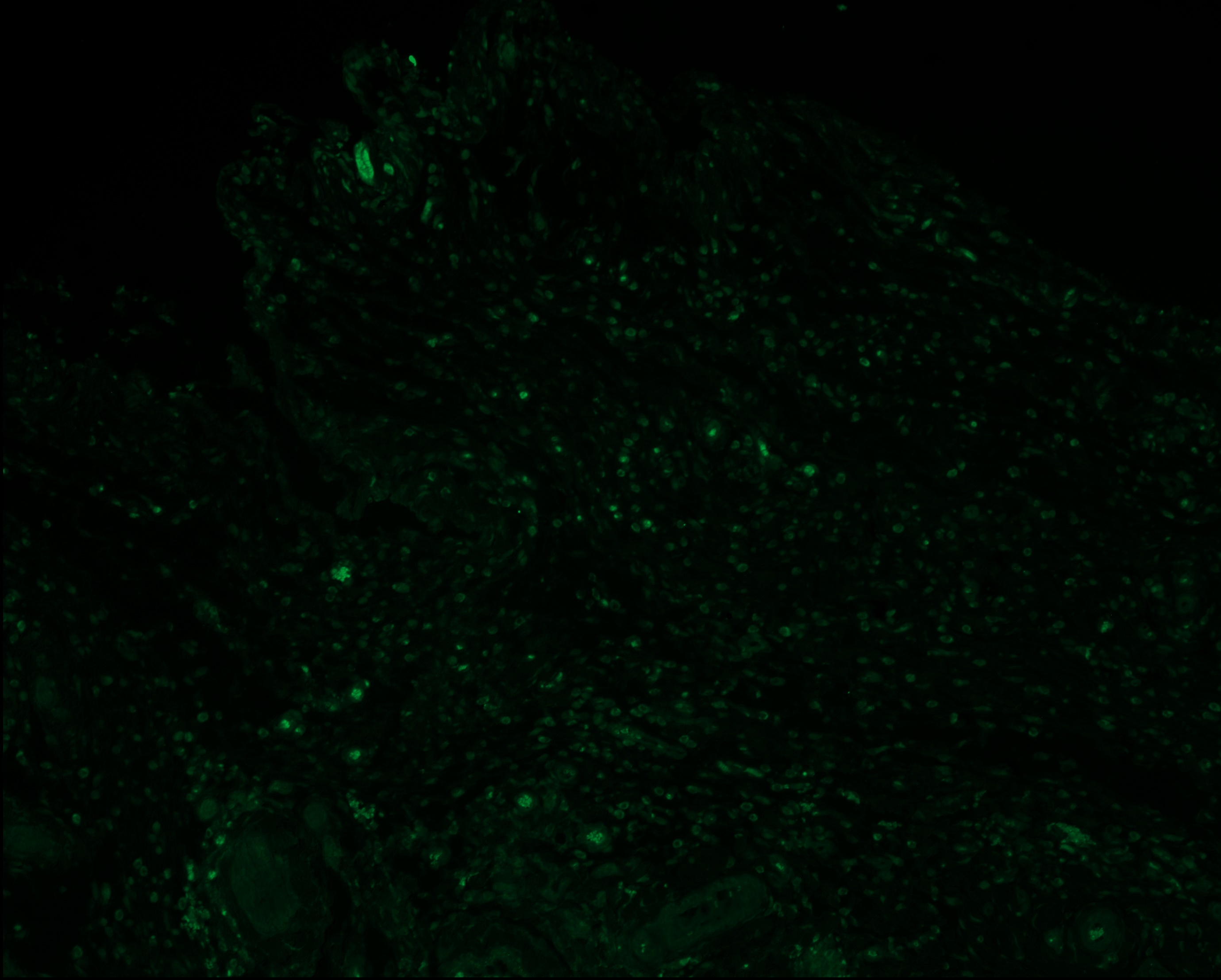

Supplement: Supplementary file 1 — Supplementary Information. [file 41598_2023_39765_MOESM1_ESM.zip › ╘¡╩╝╩2╛▌╒√└φ/tissue immunofluorescence/cd68/control (3)/Snap-4096_c3.jpg]

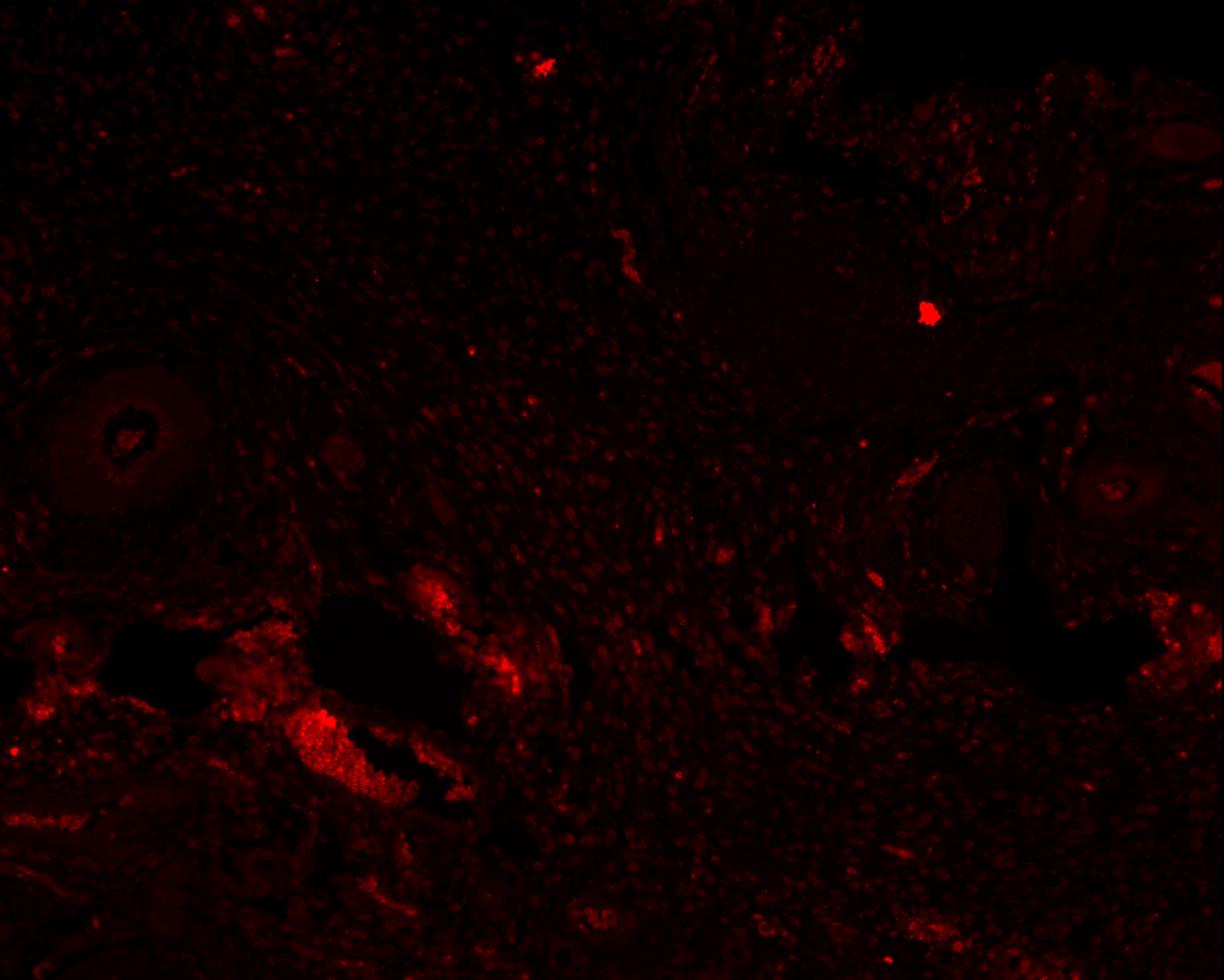

Supplement: Supplementary file 1 — Supplementary Information. [file 41598_2023_39765_MOESM1_ESM.zip › ╘¡╩╝╩2╛▌╒√└φ/tissue immunofluorescence/cd68/control/Snap-4098_c1.jpg]

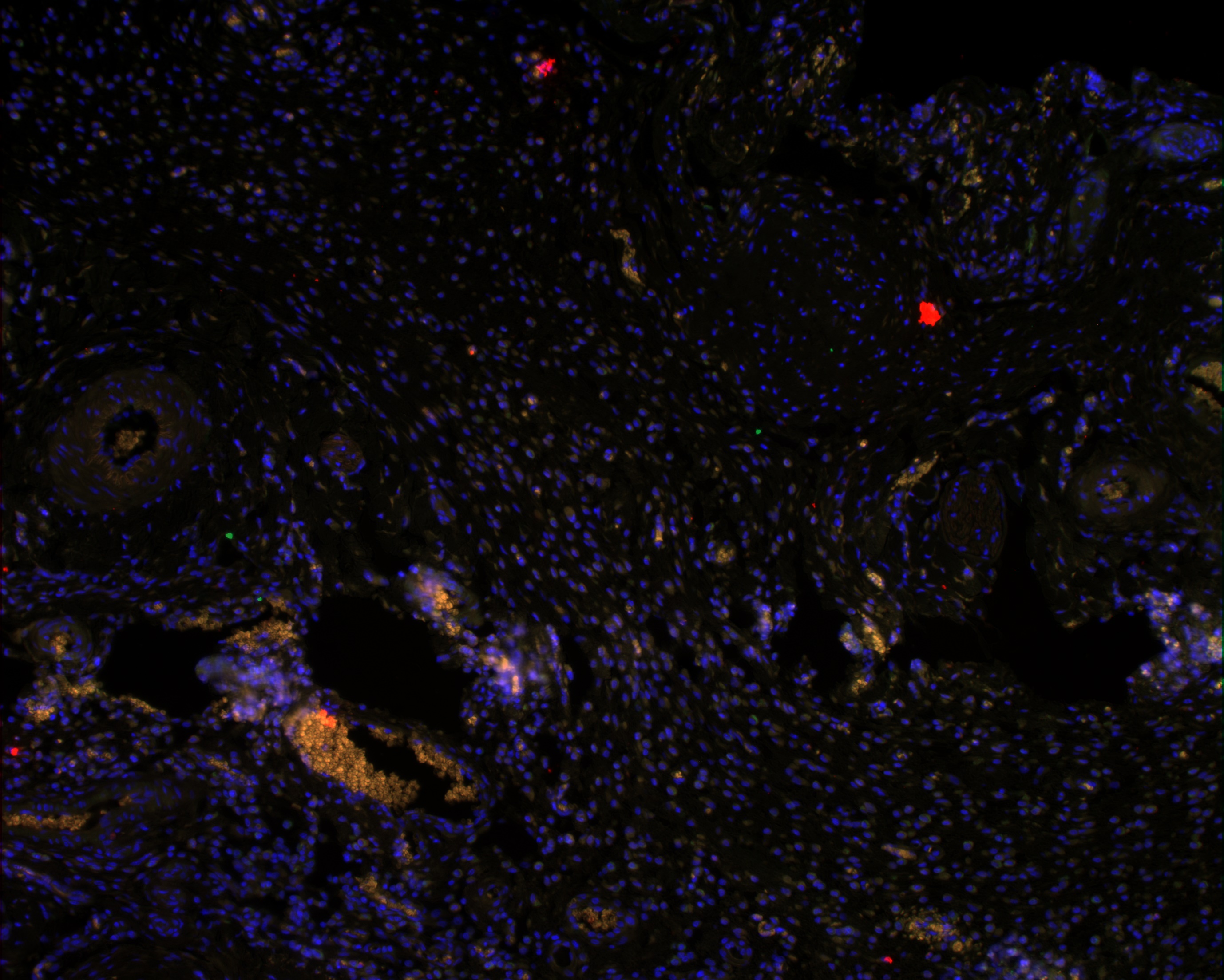

Supplement: Supplementary file 1 — Supplementary Information. [file 41598_2023_39765_MOESM1_ESM.zip › ╘¡╩╝╩2╛▌╒√└φ/tissue immunofluorescence/cd68/control/Snap-4098_c1+2+3.jpg]

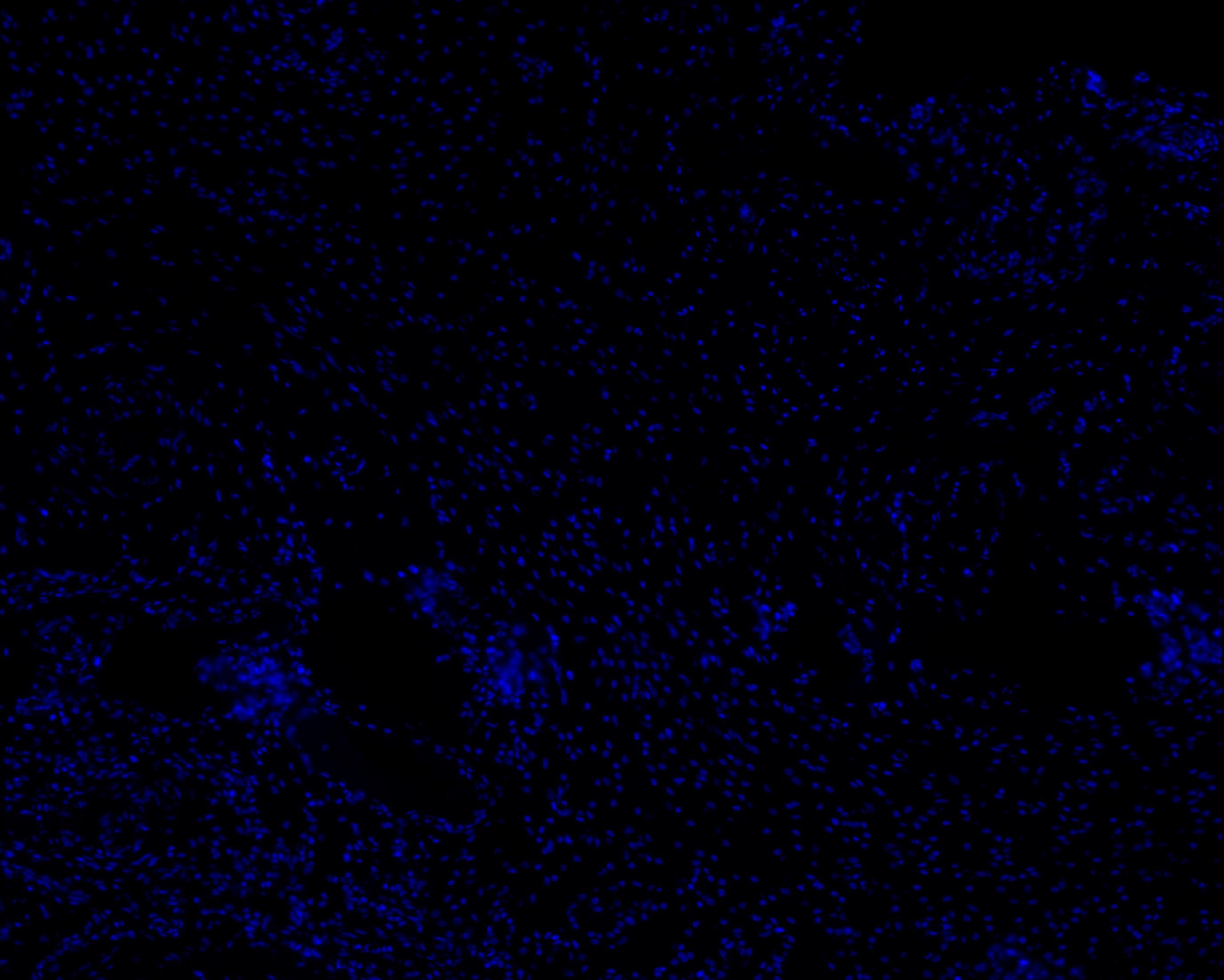

Supplement: Supplementary file 1 — Supplementary Information. [file 41598_2023_39765_MOESM1_ESM.zip › ╘¡╩╝╩2╛▌╒√└φ/tissue immunofluorescence/cd68/control/Snap-4098_c2.jpg]

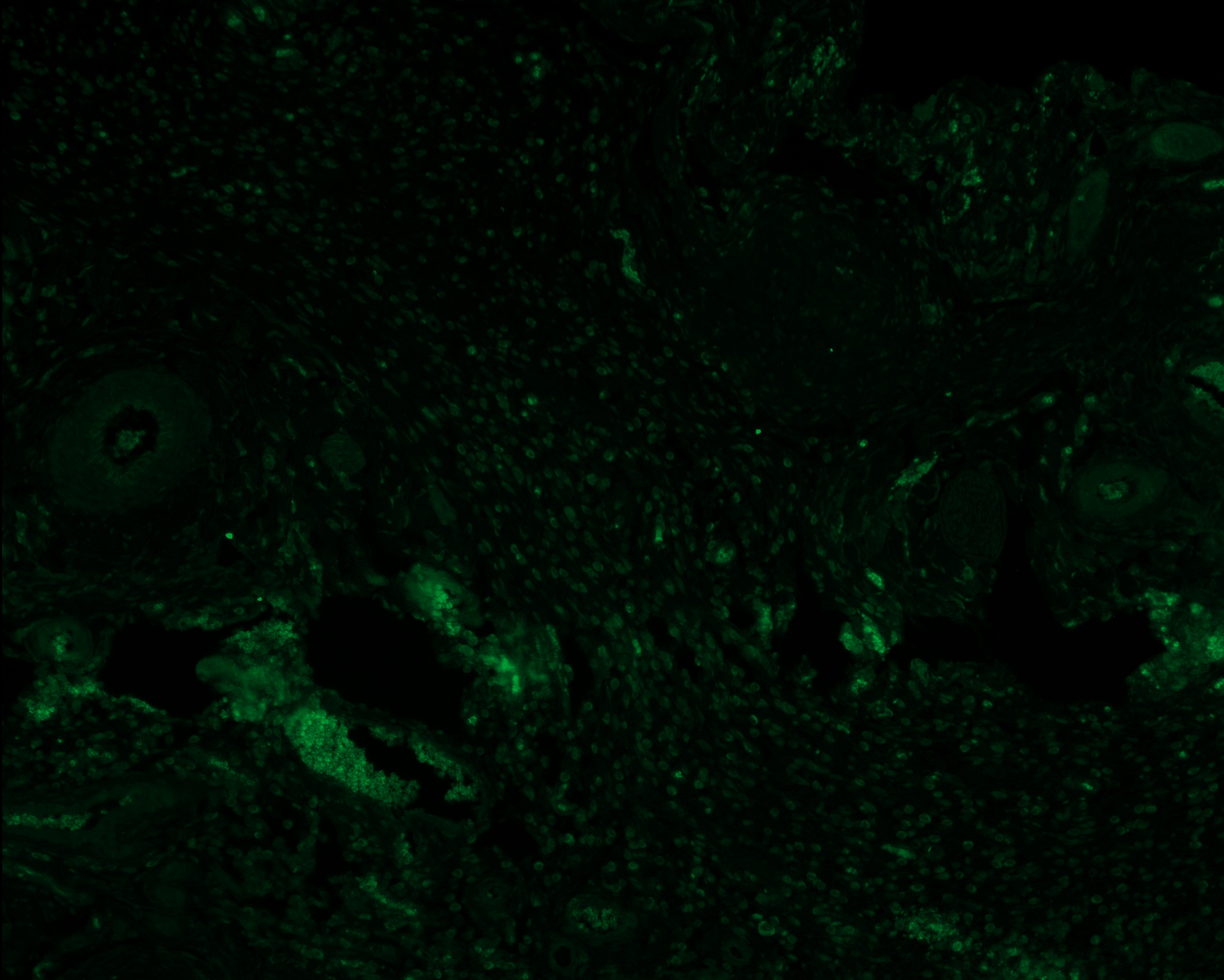

Supplement: Supplementary file 1 — Supplementary Information. [file 41598_2023_39765_MOESM1_ESM.zip › ╘¡╩╝╩2╛▌╒√└φ/tissue immunofluorescence/cd68/control/Snap-4098_c3.jpg]

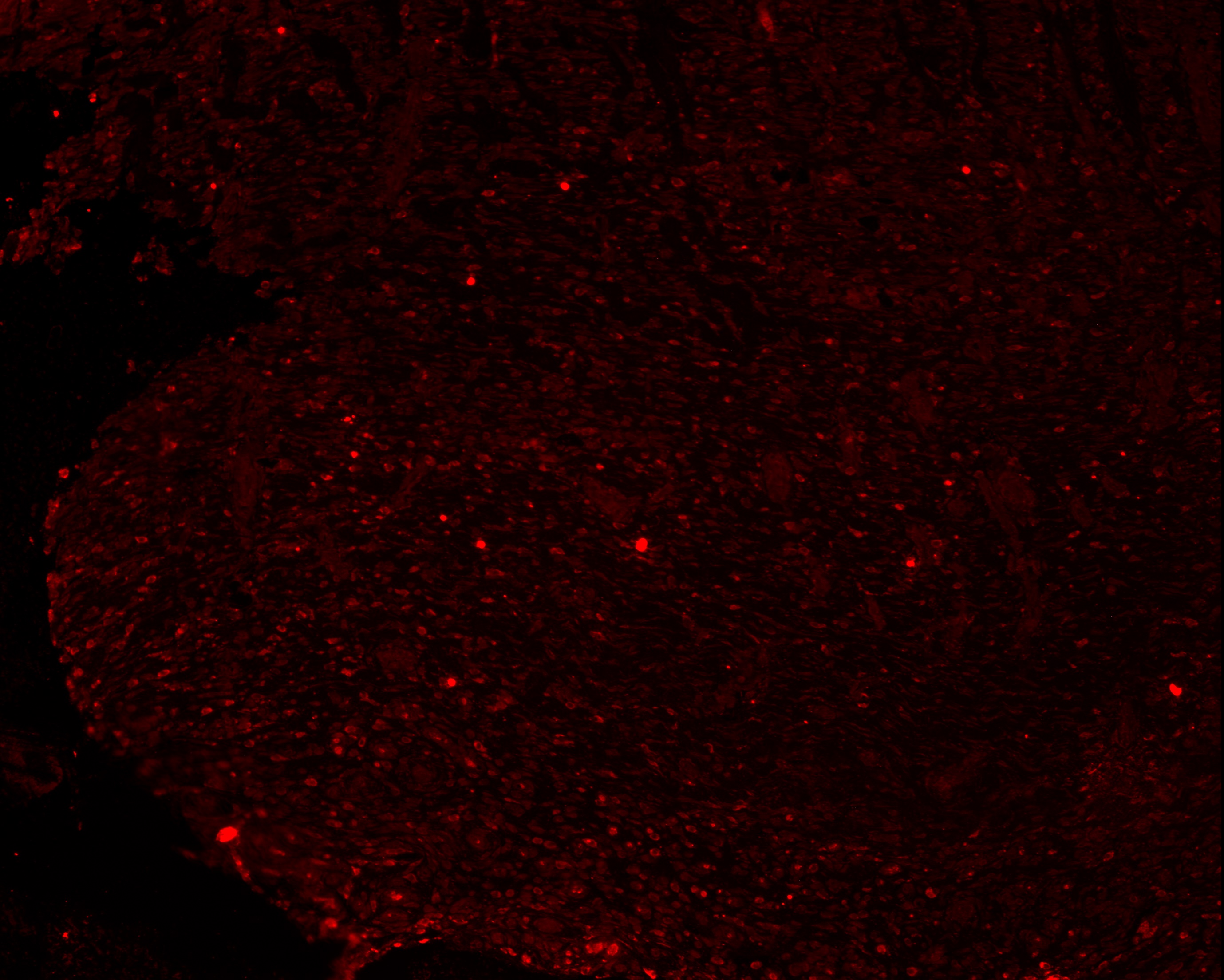

Supplement: Supplementary file 1 — Supplementary Information. [file 41598_2023_39765_MOESM1_ESM.zip › ╘¡╩╝╩2╛▌╒√└φ/tissue immunofluorescence/cd68/keratinase/Snap-4133/Snap-4133_c1.jpg]

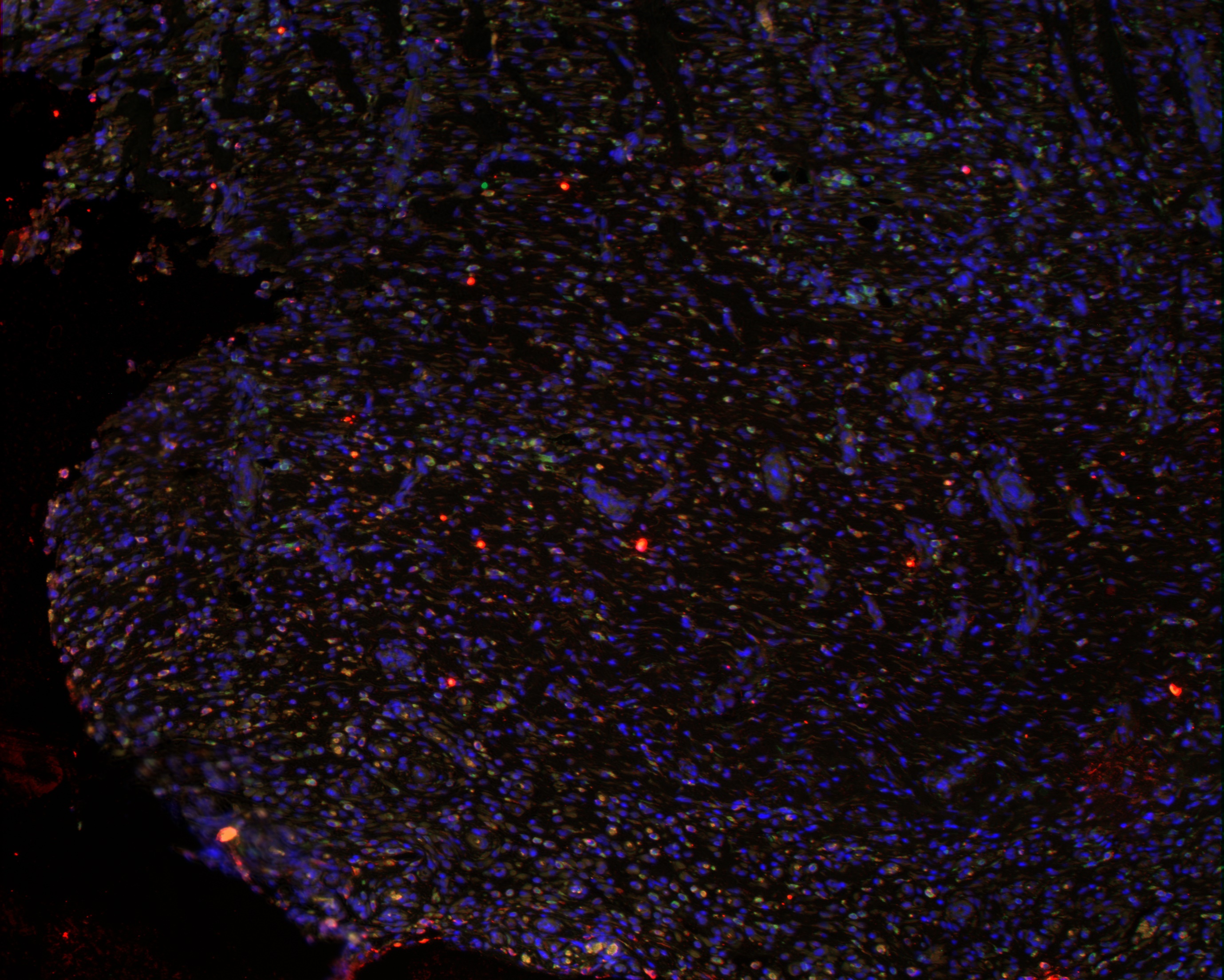

Supplement: Supplementary file 1 — Supplementary Information. [file 41598_2023_39765_MOESM1_ESM.zip › ╘¡╩╝╩2╛▌╒√└φ/tissue immunofluorescence/cd68/keratinase/Snap-4133/Snap-4133_c1+2+3.jpg]

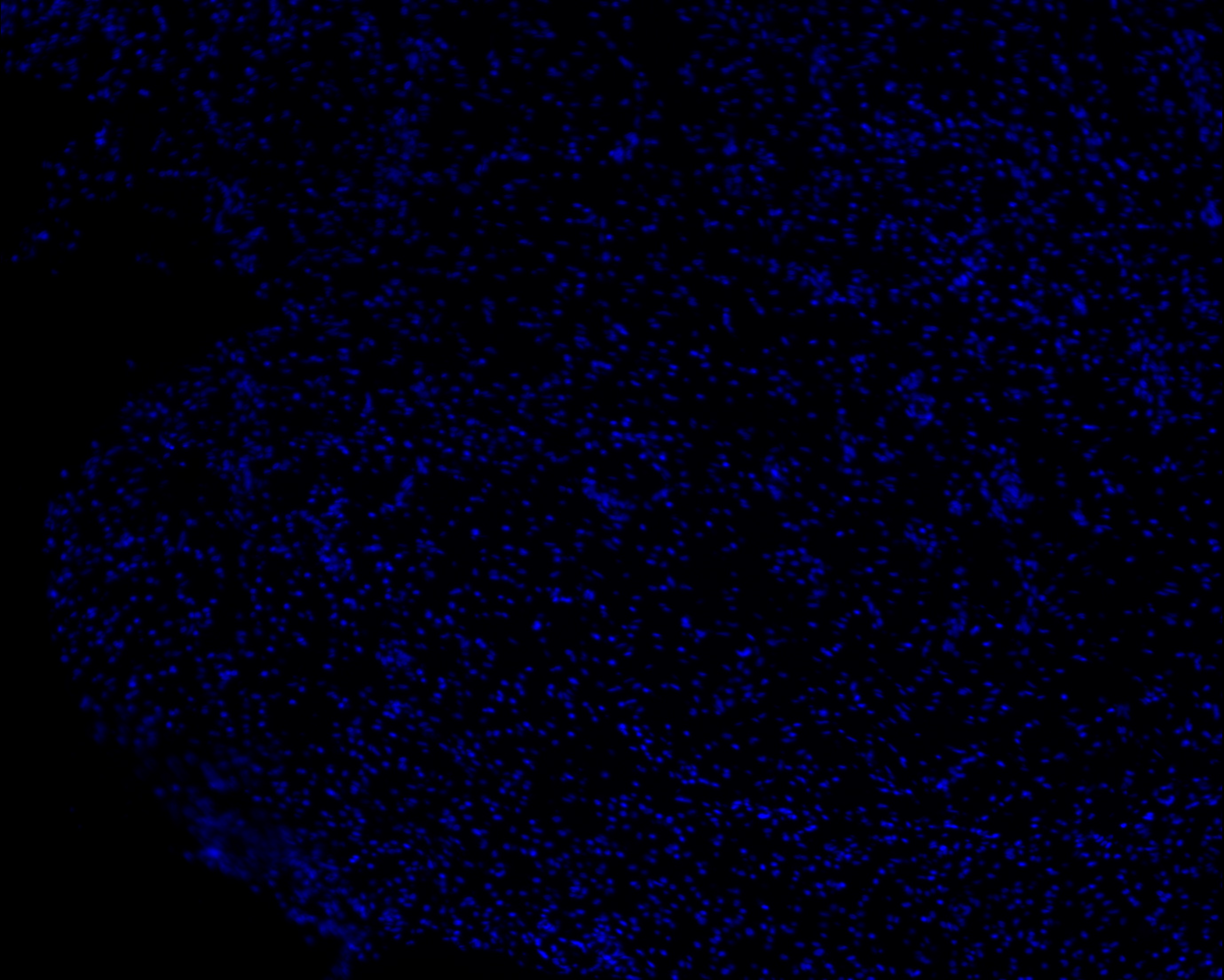

Supplement: Supplementary file 1 — Supplementary Information. [file 41598_2023_39765_MOESM1_ESM.zip › ╘¡╩╝╩2╛▌╒√└φ/tissue immunofluorescence/cd68/keratinase/Snap-4133/Snap-4133_c2.jpg]

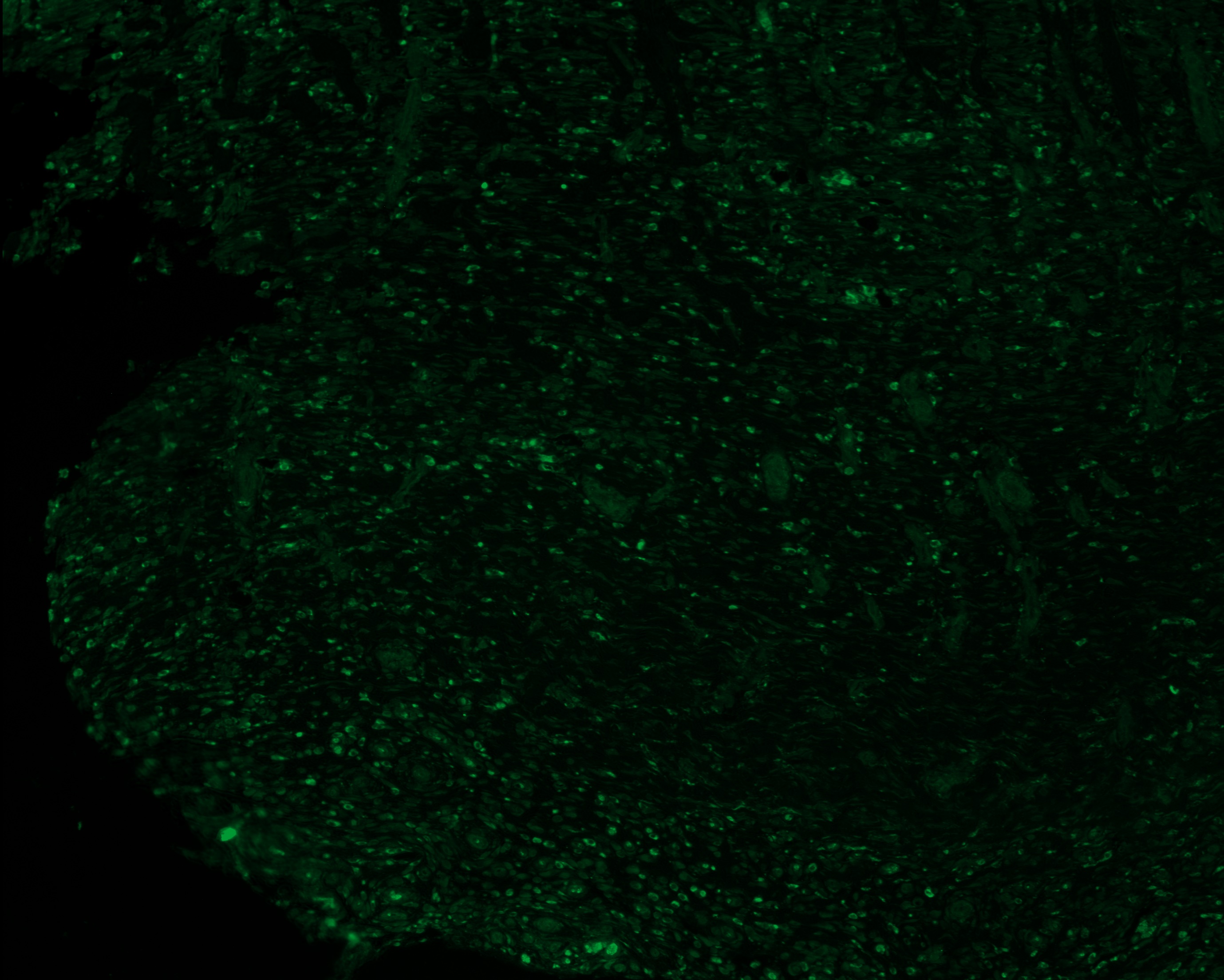

Supplement: Supplementary file 1 — Supplementary Information. [file 41598_2023_39765_MOESM1_ESM.zip › ╘¡╩╝╩2╛▌╒√└φ/tissue immunofluorescence/cd68/keratinase/Snap-4133/Snap-4133_c3.jpg]

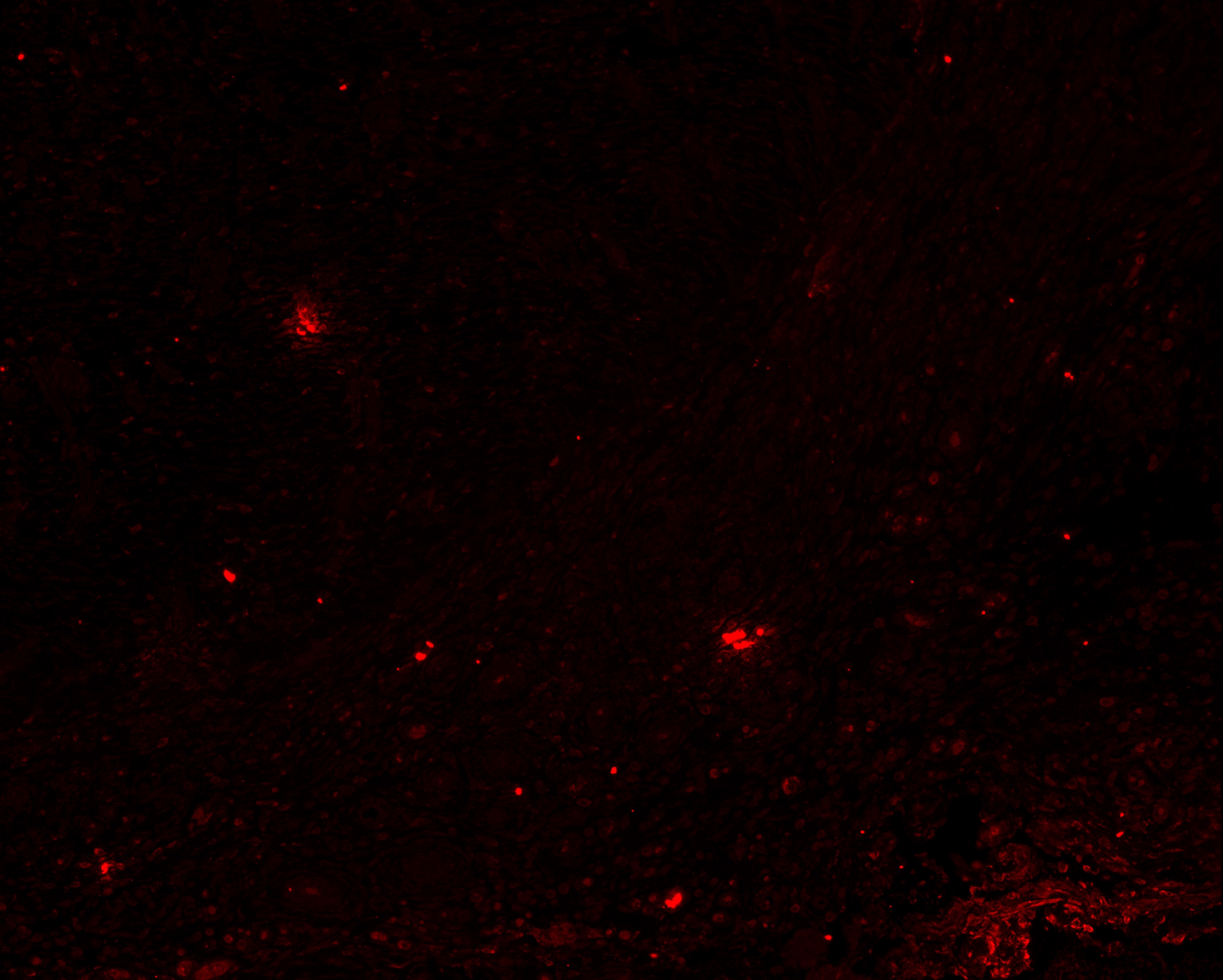

Supplement: Supplementary file 1 — Supplementary Information. [file 41598_2023_39765_MOESM1_ESM.zip › ╘¡╩╝╩2╛▌╒√└φ/tissue immunofluorescence/cd68/Snap-4134/Snap-4134_c1.jpg]

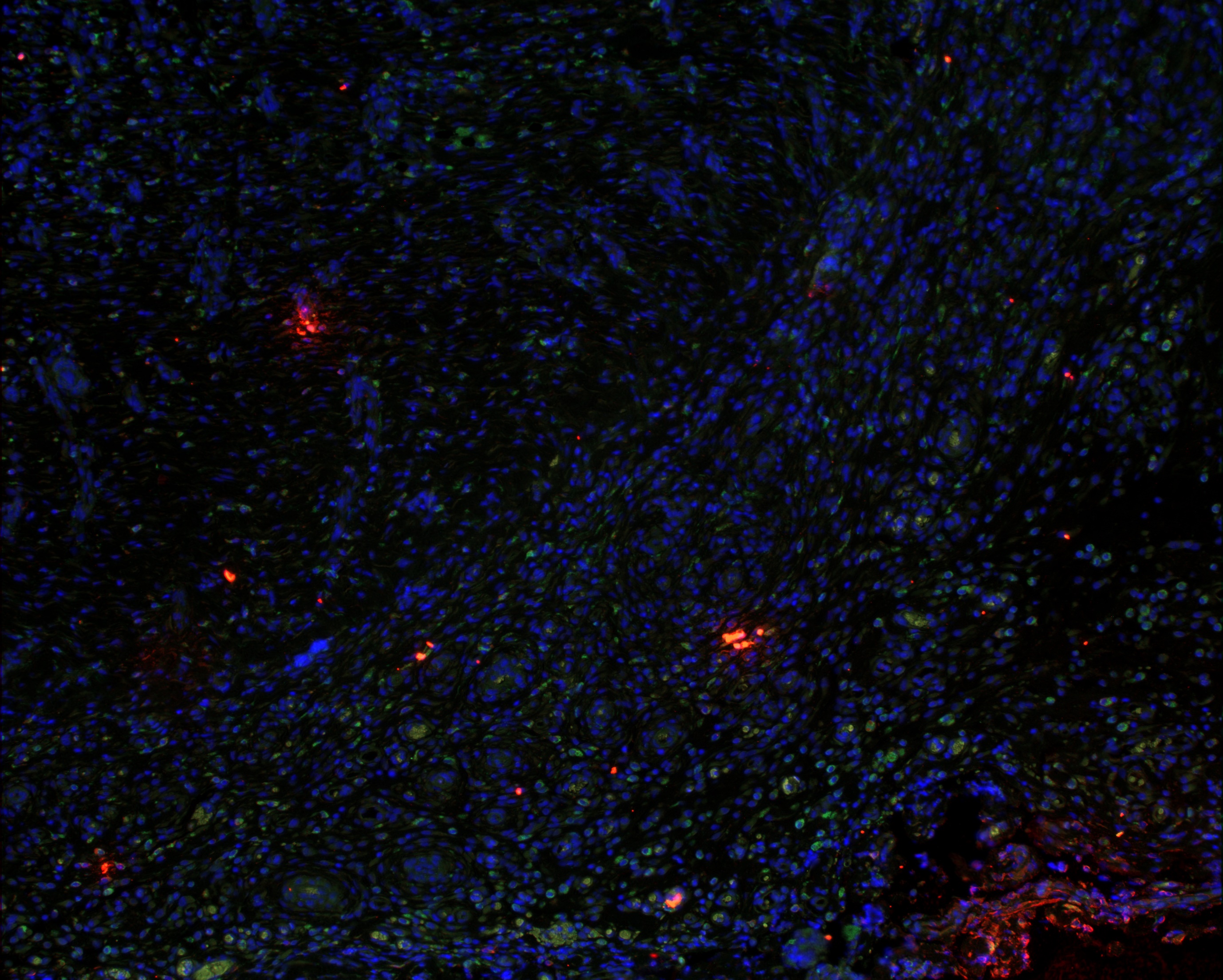

Supplement: Supplementary file 1 — Supplementary Information. [file 41598_2023_39765_MOESM1_ESM.zip › ╘¡╩╝╩2╛▌╒√└φ/tissue immunofluorescence/cd68/Snap-4134/Snap-4134_c1+2+3.jpg]

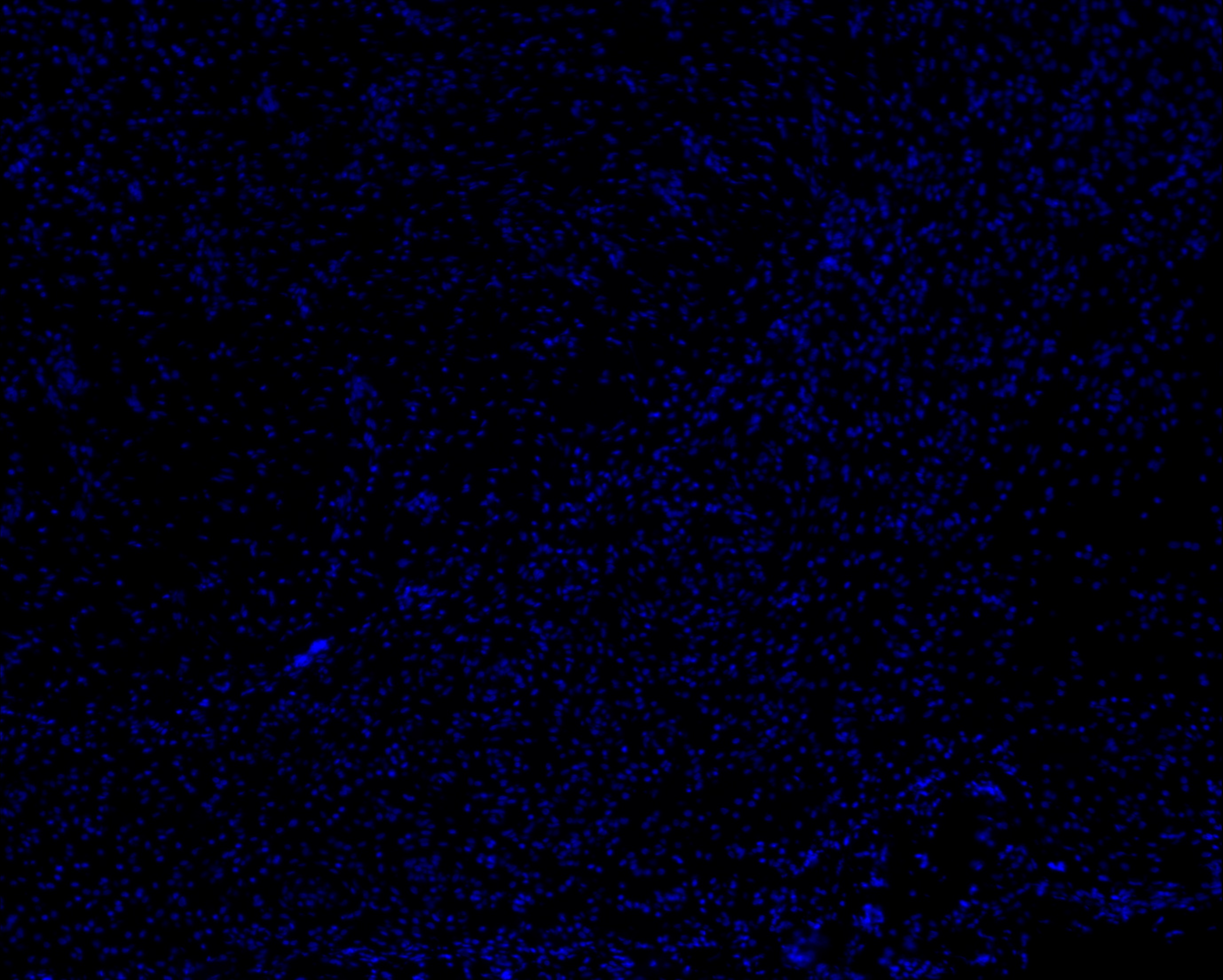

Supplement: Supplementary file 1 — Supplementary Information. [file 41598_2023_39765_MOESM1_ESM.zip › ╘¡╩╝╩2╛▌╒√└φ/tissue immunofluorescence/cd68/Snap-4134/Snap-4134_c2.jpg]

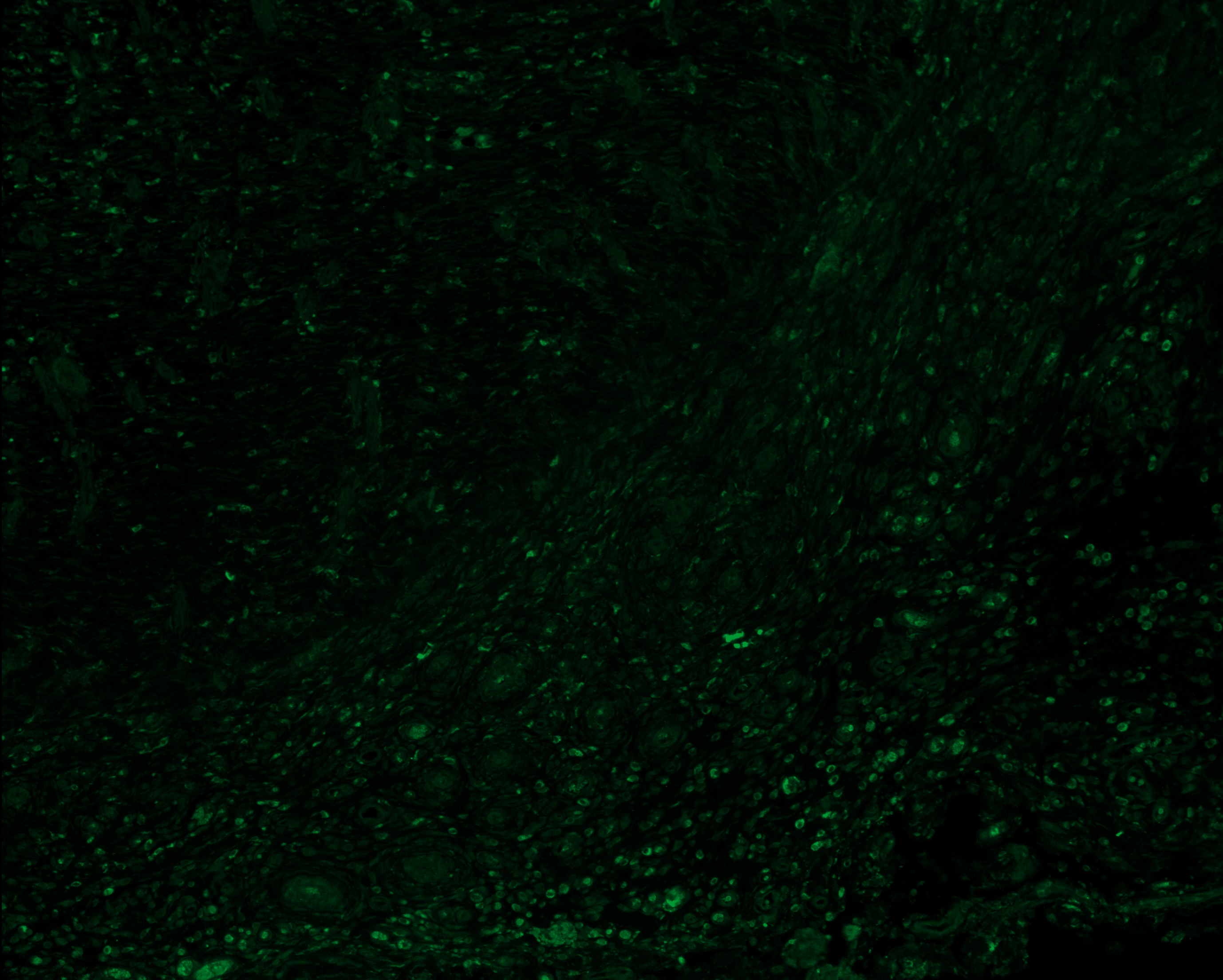

Supplement: Supplementary file 1 — Supplementary Information. [file 41598_2023_39765_MOESM1_ESM.zip › ╘¡╩╝╩2╛▌╒√└φ/tissue immunofluorescence/cd68/Snap-4134/Snap-4134_c3.jpg]

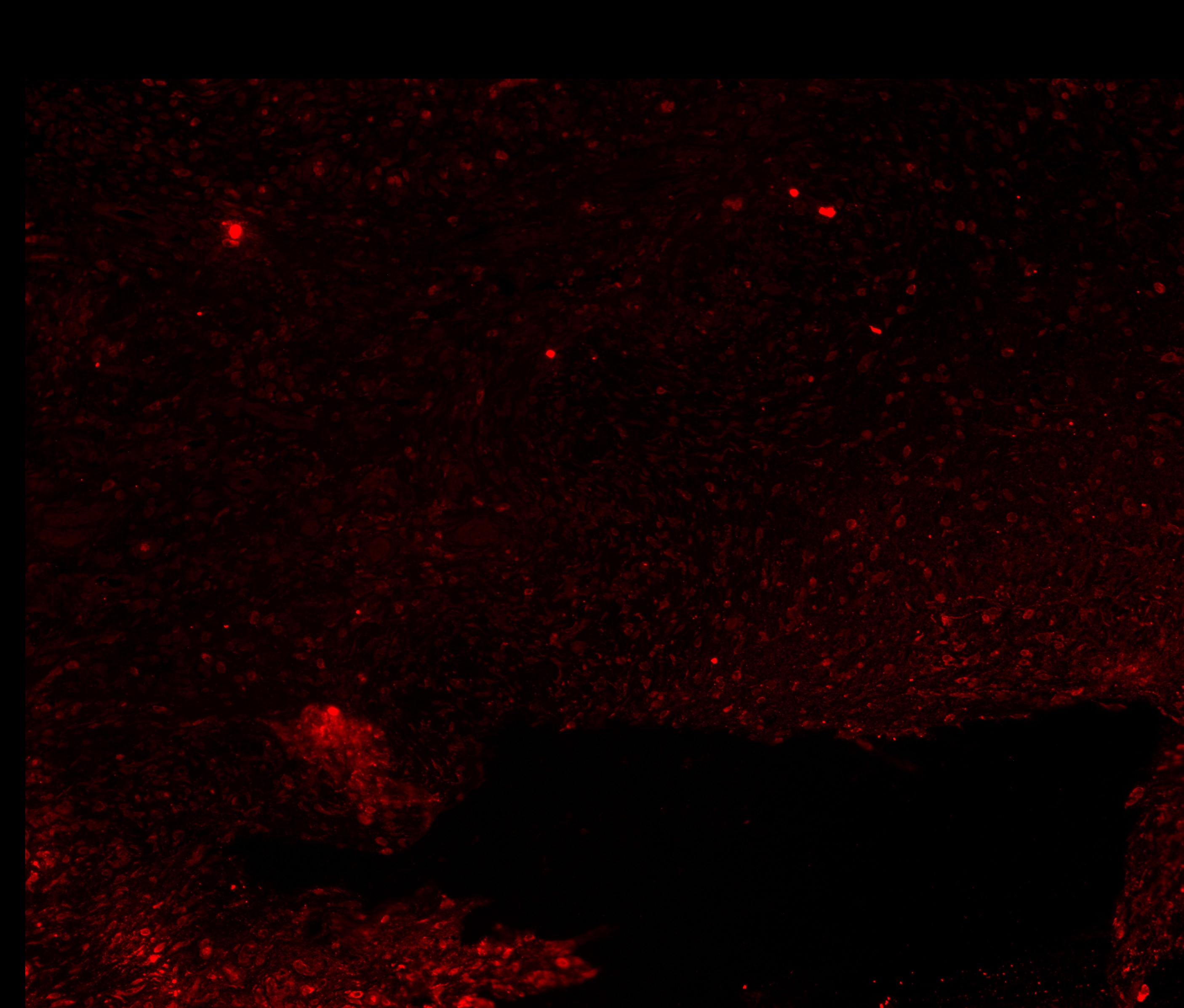

Supplement: Supplementary file 1 — Supplementary Information. [file 41598_2023_39765_MOESM1_ESM.zip › ╘¡╩╝╩2╛▌╒√└φ/tissue immunofluorescence/cd68/Snap-4139/Snap-4139_c1.jpg]

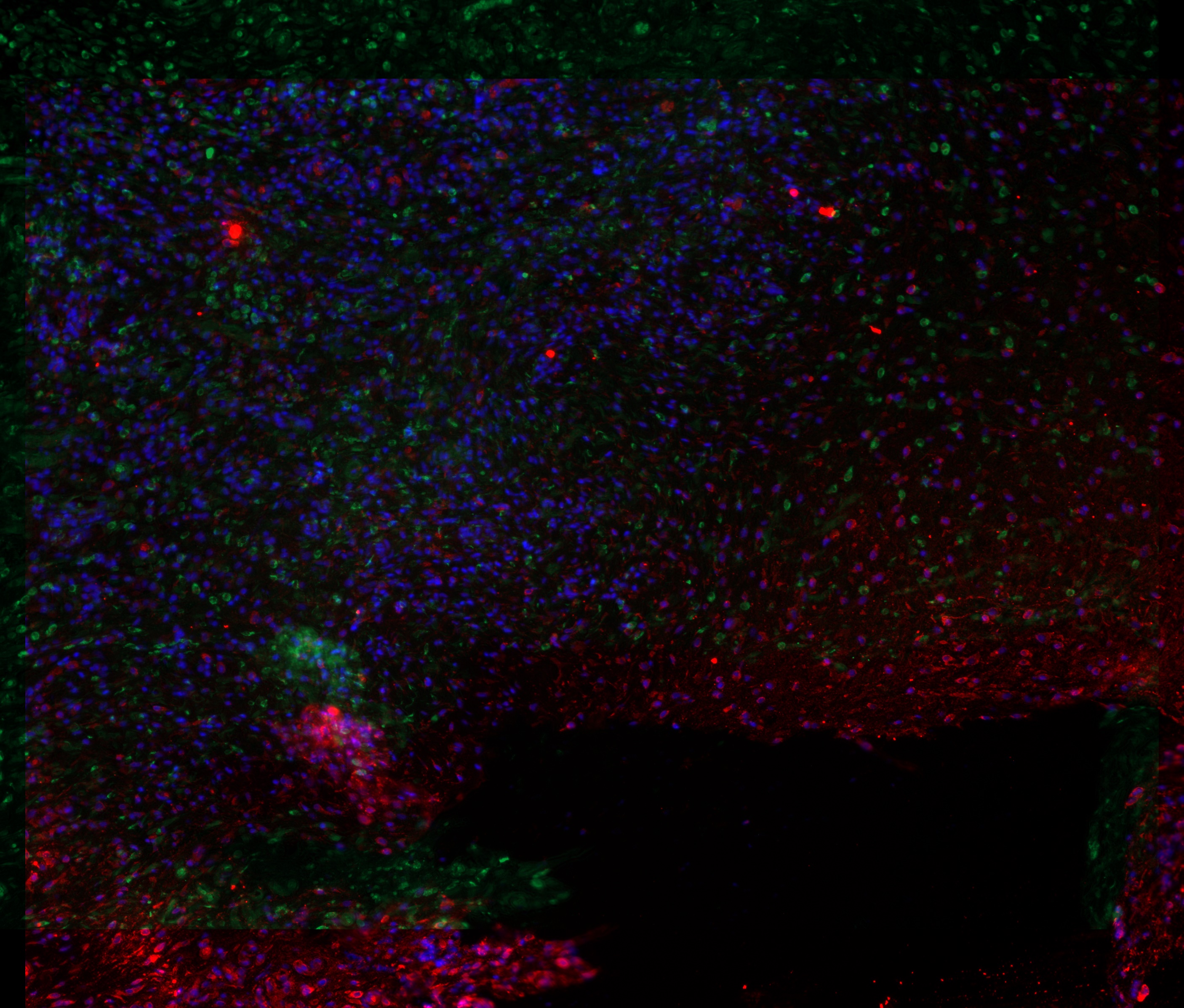

Supplement: Supplementary file 1 — Supplementary Information. [file 41598_2023_39765_MOESM1_ESM.zip › ╘¡╩╝╩2╛▌╒√└φ/tissue immunofluorescence/cd68/Snap-4139/Snap-4139_c1+2+3.jpg]

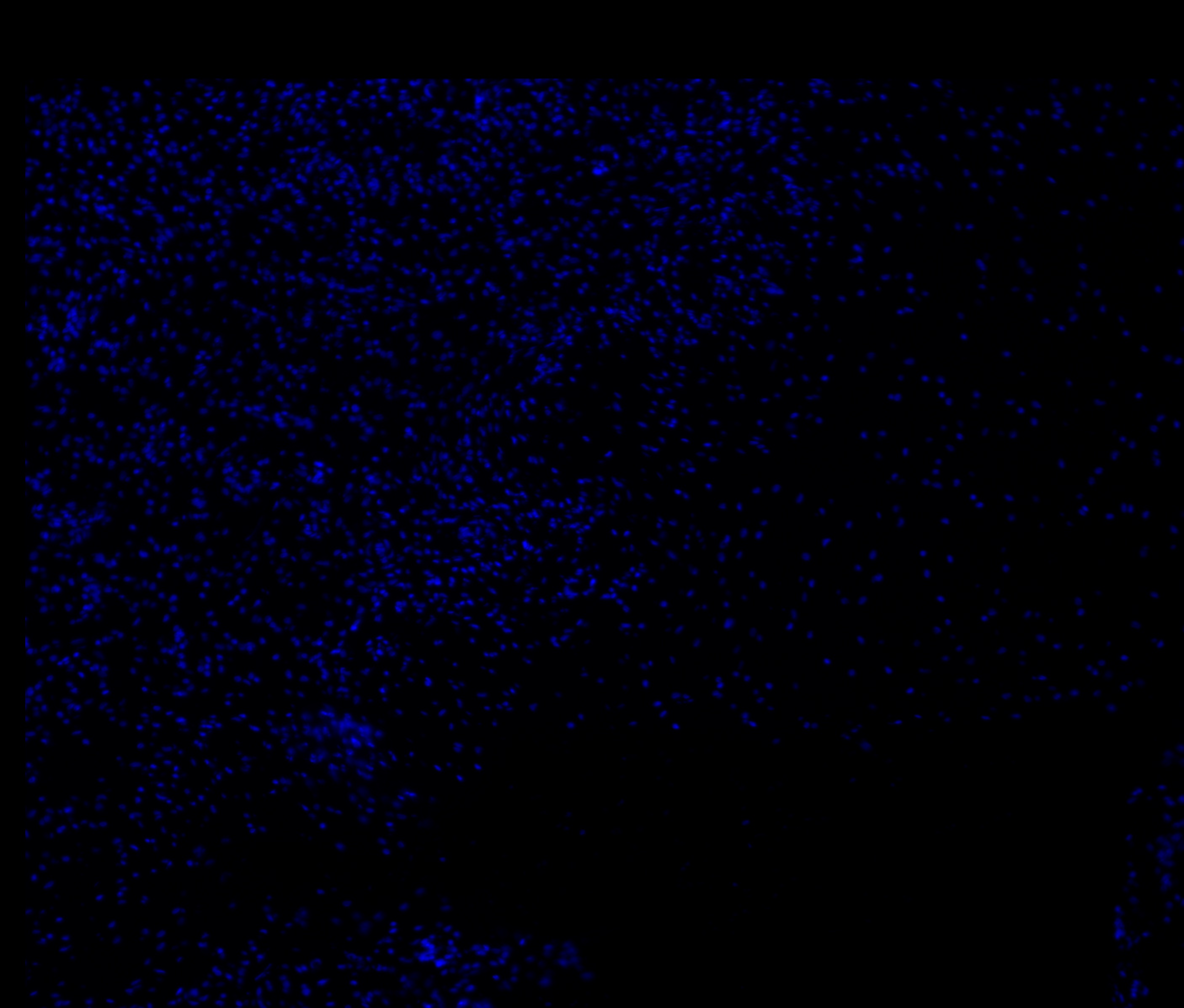

Supplement: Supplementary file 1 — Supplementary Information. [file 41598_2023_39765_MOESM1_ESM.zip › ╘¡╩╝╩2╛▌╒√└φ/tissue immunofluorescence/cd68/Snap-4139/Snap-4139_c2.jpg]

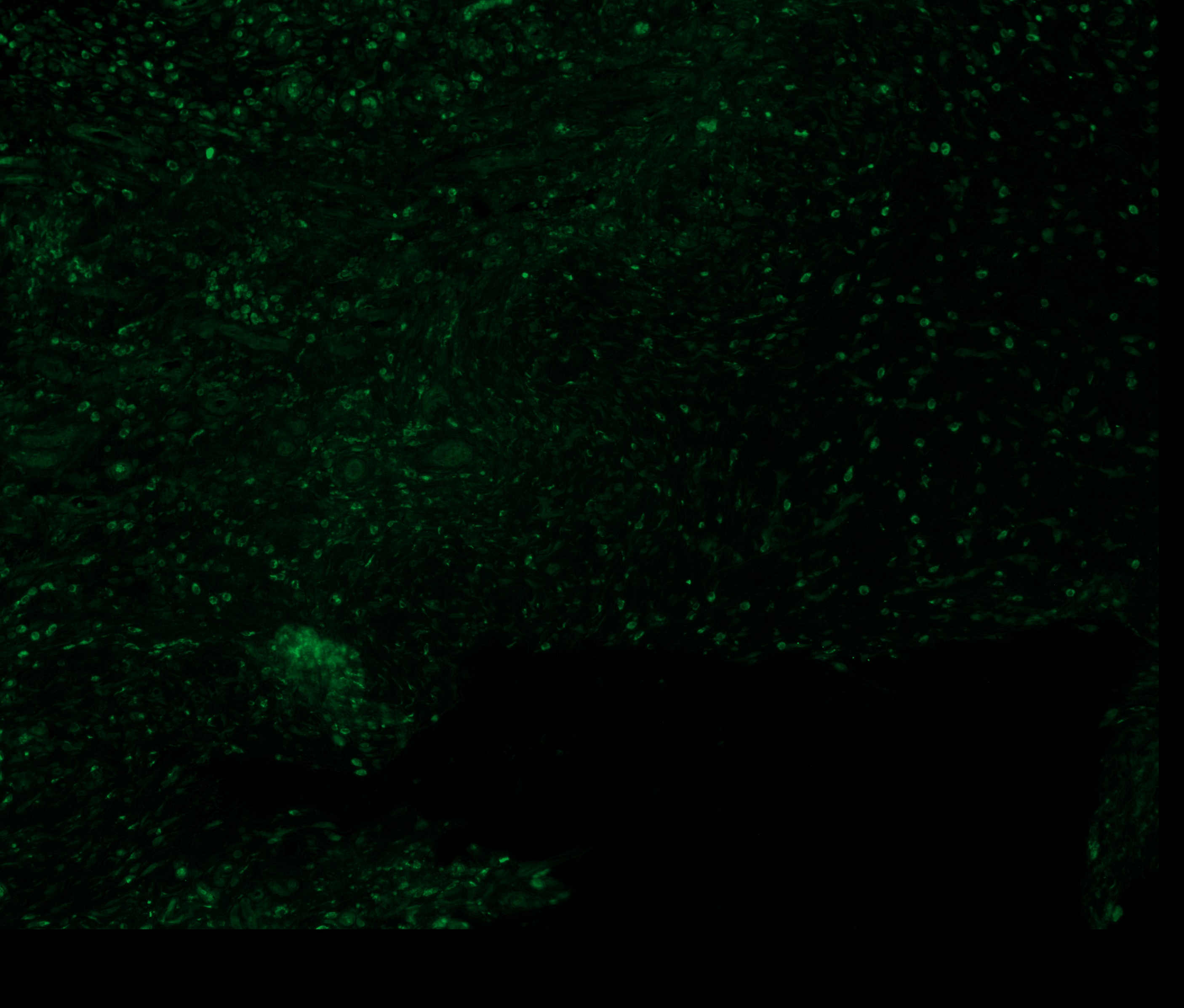

Supplement: Supplementary file 1 — Supplementary Information. [file 41598_2023_39765_MOESM1_ESM.zip › ╘¡╩╝╩2╛▌╒√└φ/tissue immunofluorescence/cd68/Snap-4139/Snap-4139_c3.jpg]

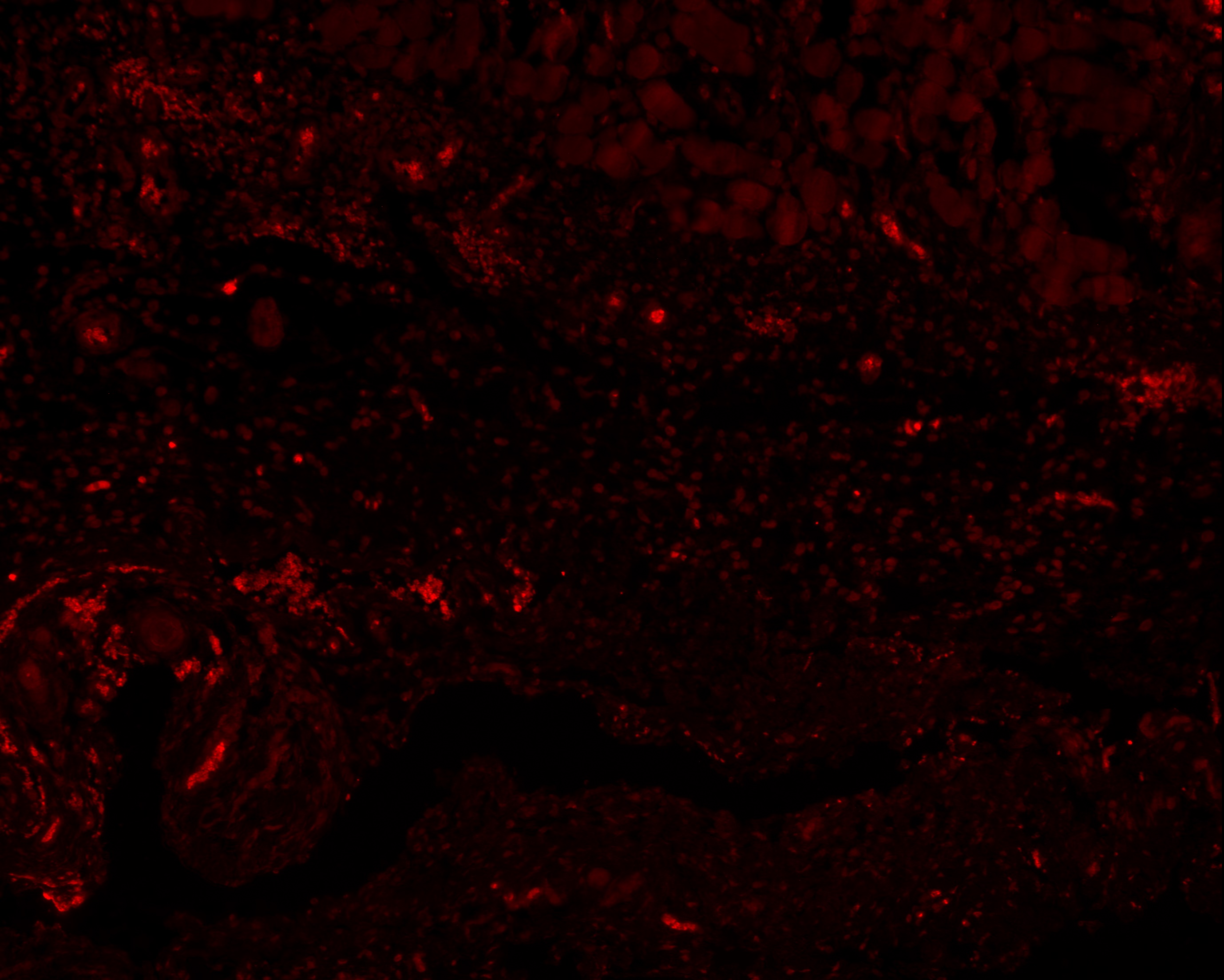

Supplement: Supplementary file 1 — Supplementary Information. [file 41598_2023_39765_MOESM1_ESM.zip › ╘¡╩╝╩2╛▌╒√└φ/tissue immunofluorescence/cd86ú║cd163/control (2)/Snap-4110_c1.jpg]

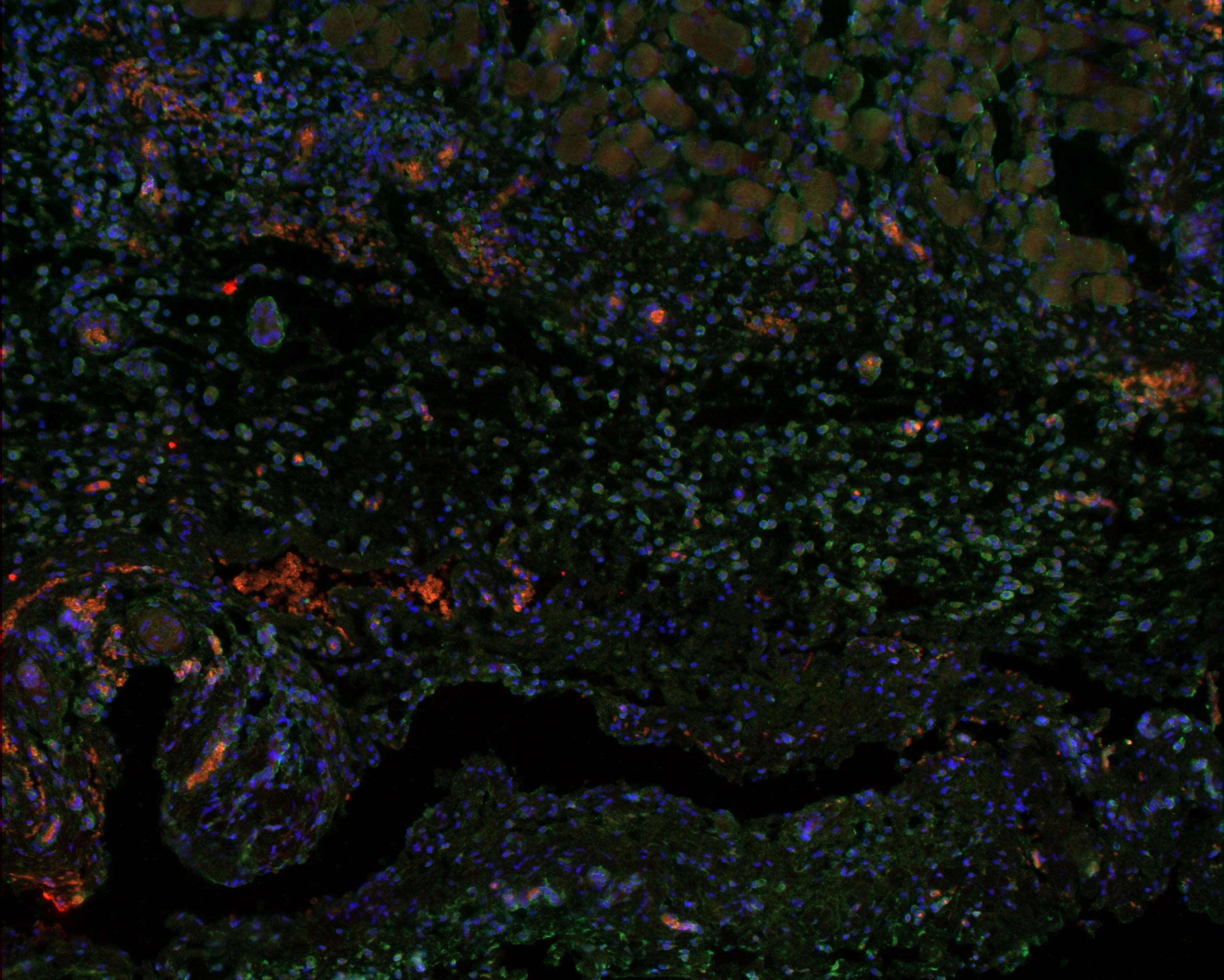

Supplement: Supplementary file 1 — Supplementary Information. [file 41598_2023_39765_MOESM1_ESM.zip › ╘¡╩╝╩2╛▌╒√└φ/tissue immunofluorescence/cd86ú║cd163/control (2)/Snap-4110_c1+2+3.jpg]

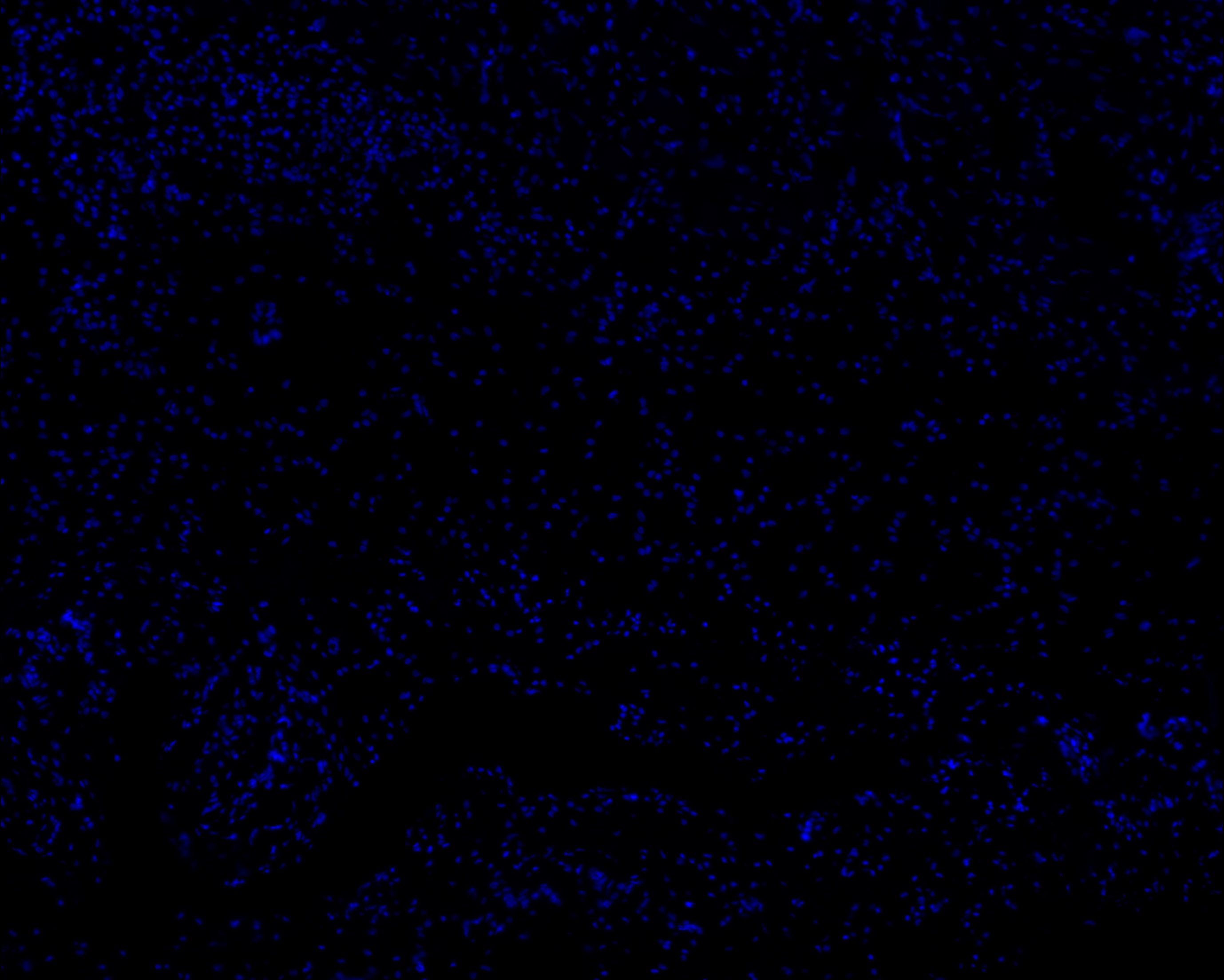

Supplement: Supplementary file 1 — Supplementary Information. [file 41598_2023_39765_MOESM1_ESM.zip › ╘¡╩╝╩2╛▌╒√└φ/tissue immunofluorescence/cd86ú║cd163/control (2)/Snap-4110_c2.jpg]

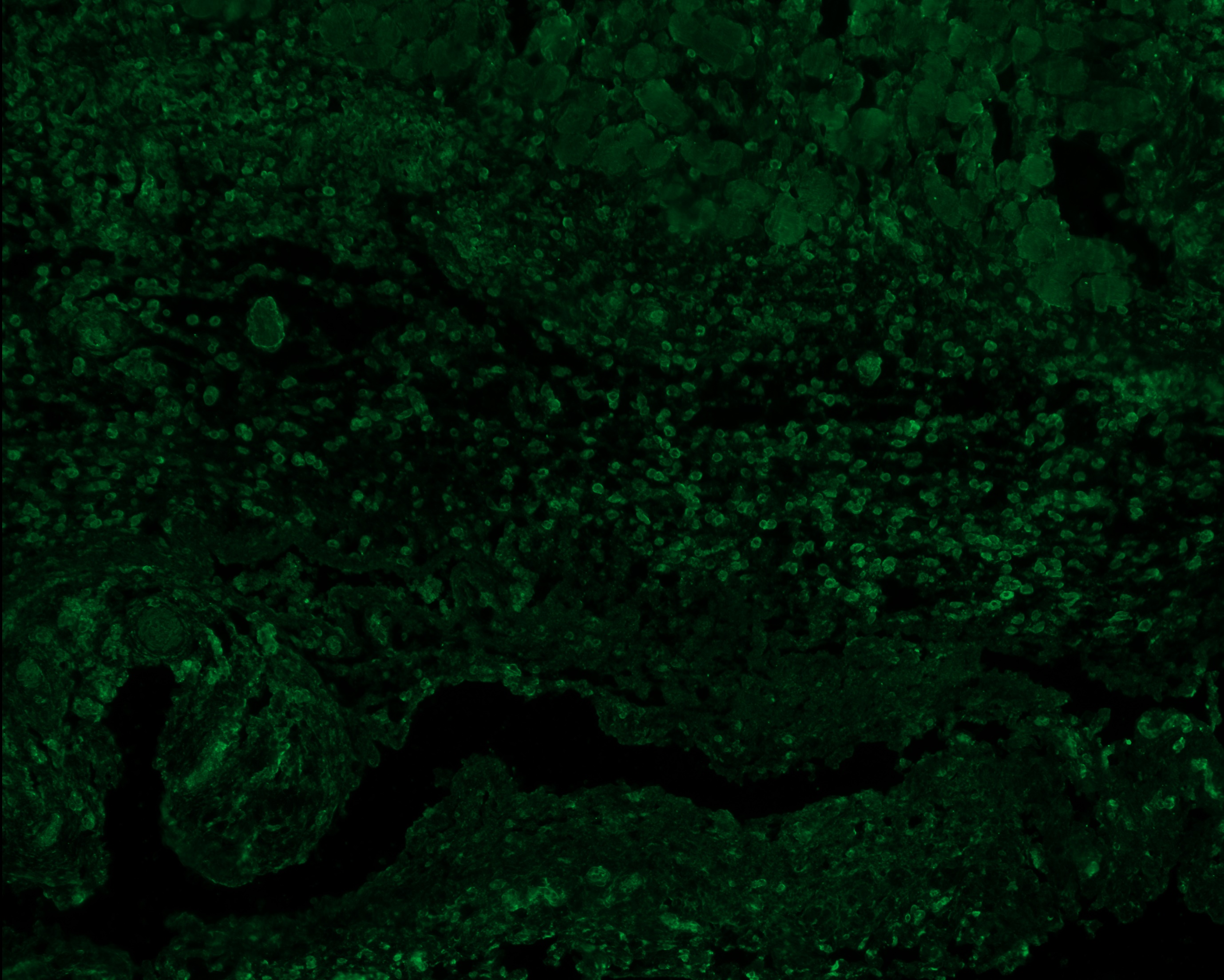

Supplement: Supplementary file 1 — Supplementary Information. [file 41598_2023_39765_MOESM1_ESM.zip › ╘¡╩╝╩2╛▌╒√└φ/tissue immunofluorescence/cd86ú║cd163/control (2)/Snap-4110_c3.jpg]

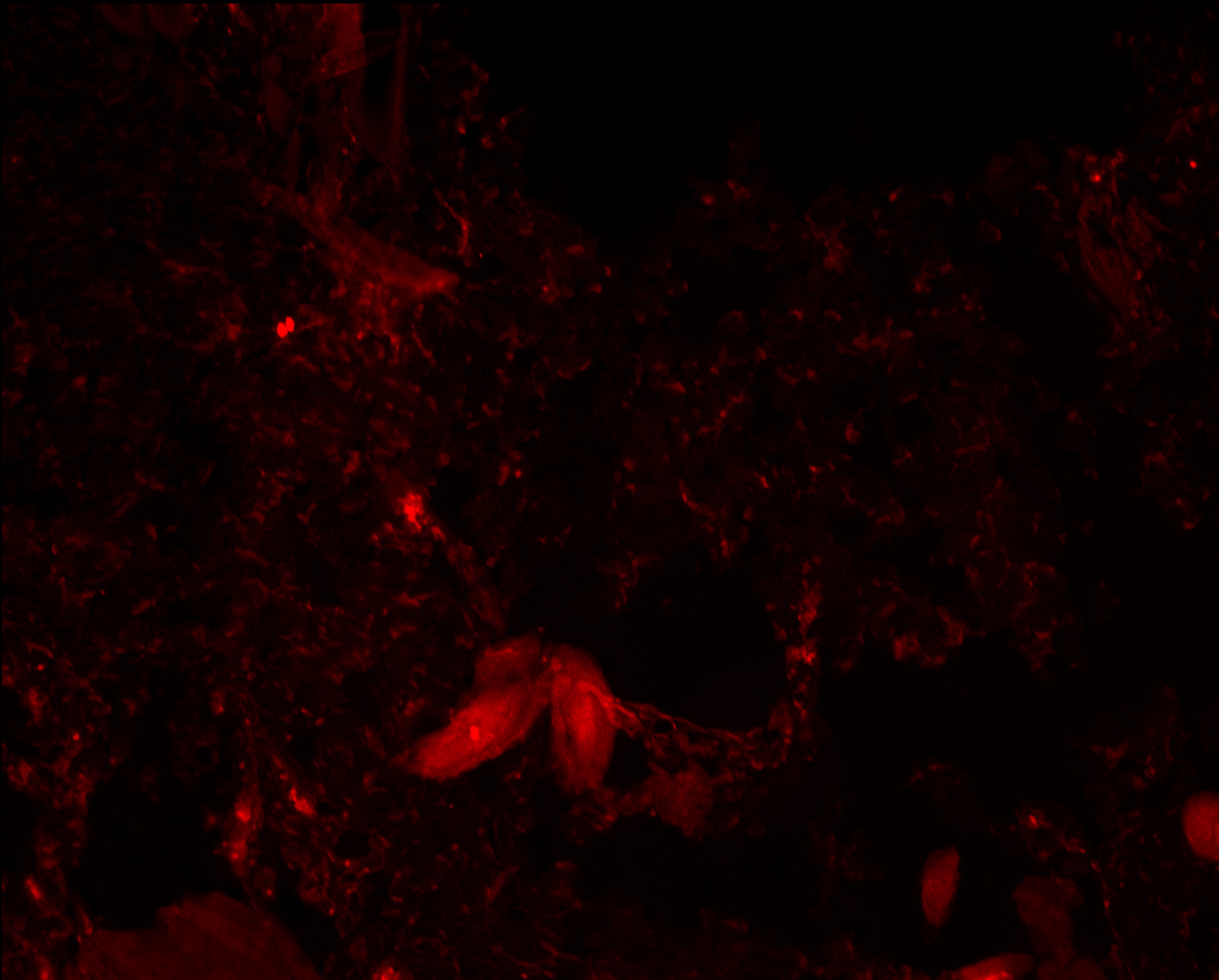

Supplement: Supplementary file 1 — Supplementary Information. [file 41598_2023_39765_MOESM1_ESM.zip › ╘¡╩╝╩2╛▌╒√└φ/tissue immunofluorescence/cd86ú║cd163/control (3)/Snap-4114_c1.jpg]

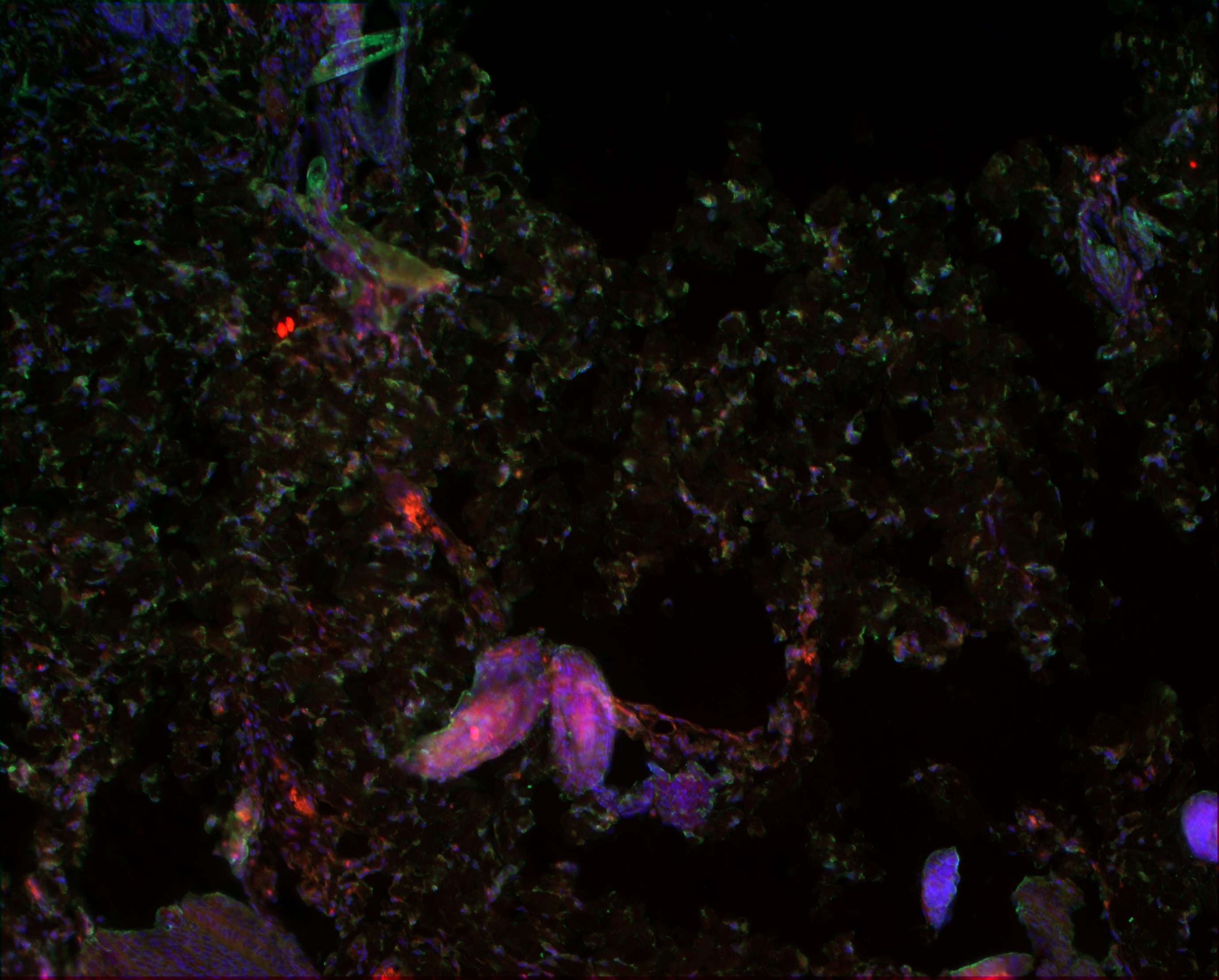

Supplement: Supplementary file 1 — Supplementary Information. [file 41598_2023_39765_MOESM1_ESM.zip › ╘¡╩╝╩2╛▌╒√└φ/tissue immunofluorescence/cd86ú║cd163/control (3)/Snap-4114_c1+2+3.jpg]

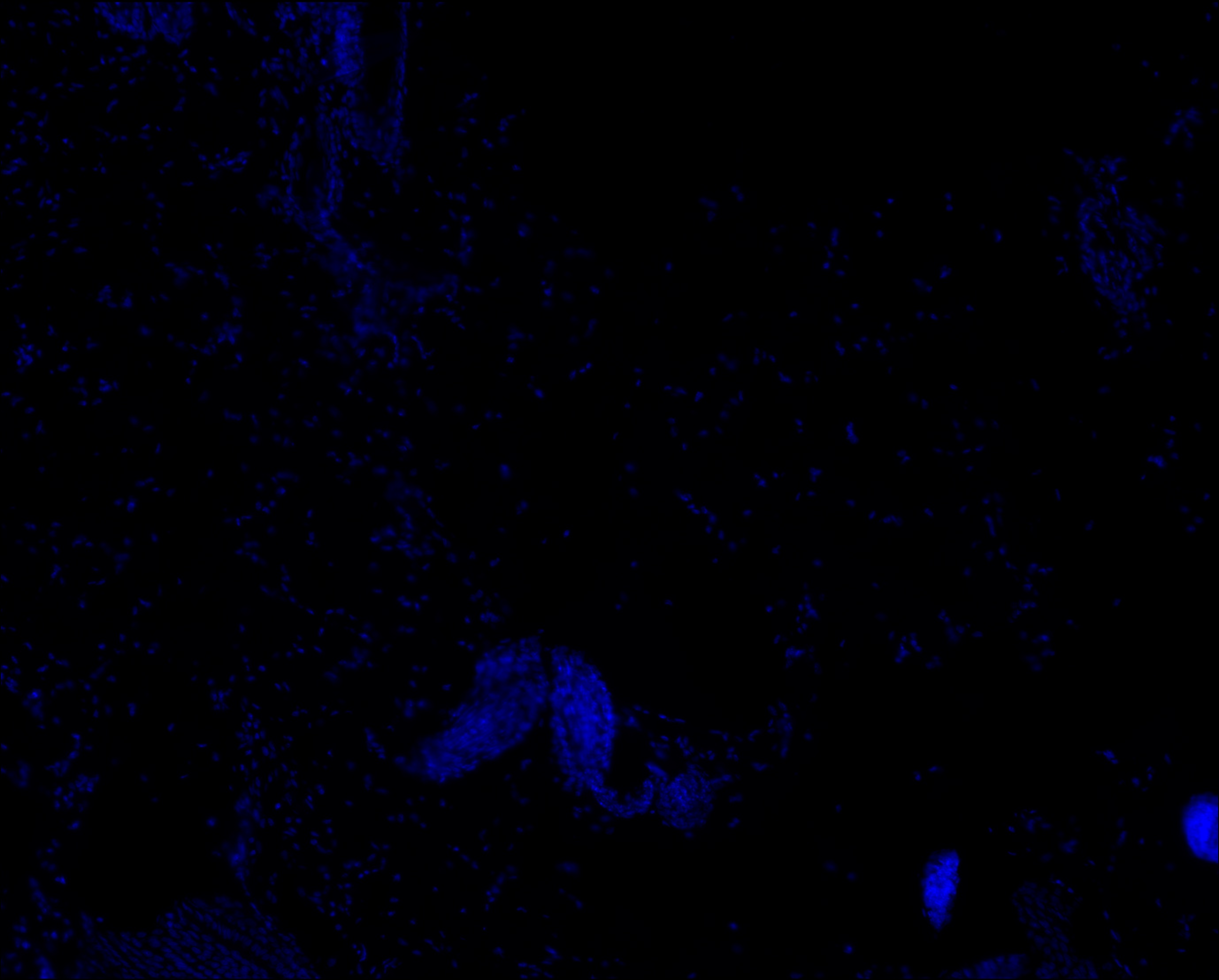

Supplement: Supplementary file 1 — Supplementary Information. [file 41598_2023_39765_MOESM1_ESM.zip › ╘¡╩╝╩2╛▌╒√└φ/tissue immunofluorescence/cd86ú║cd163/control (3)/Snap-4114_c2.jpg]

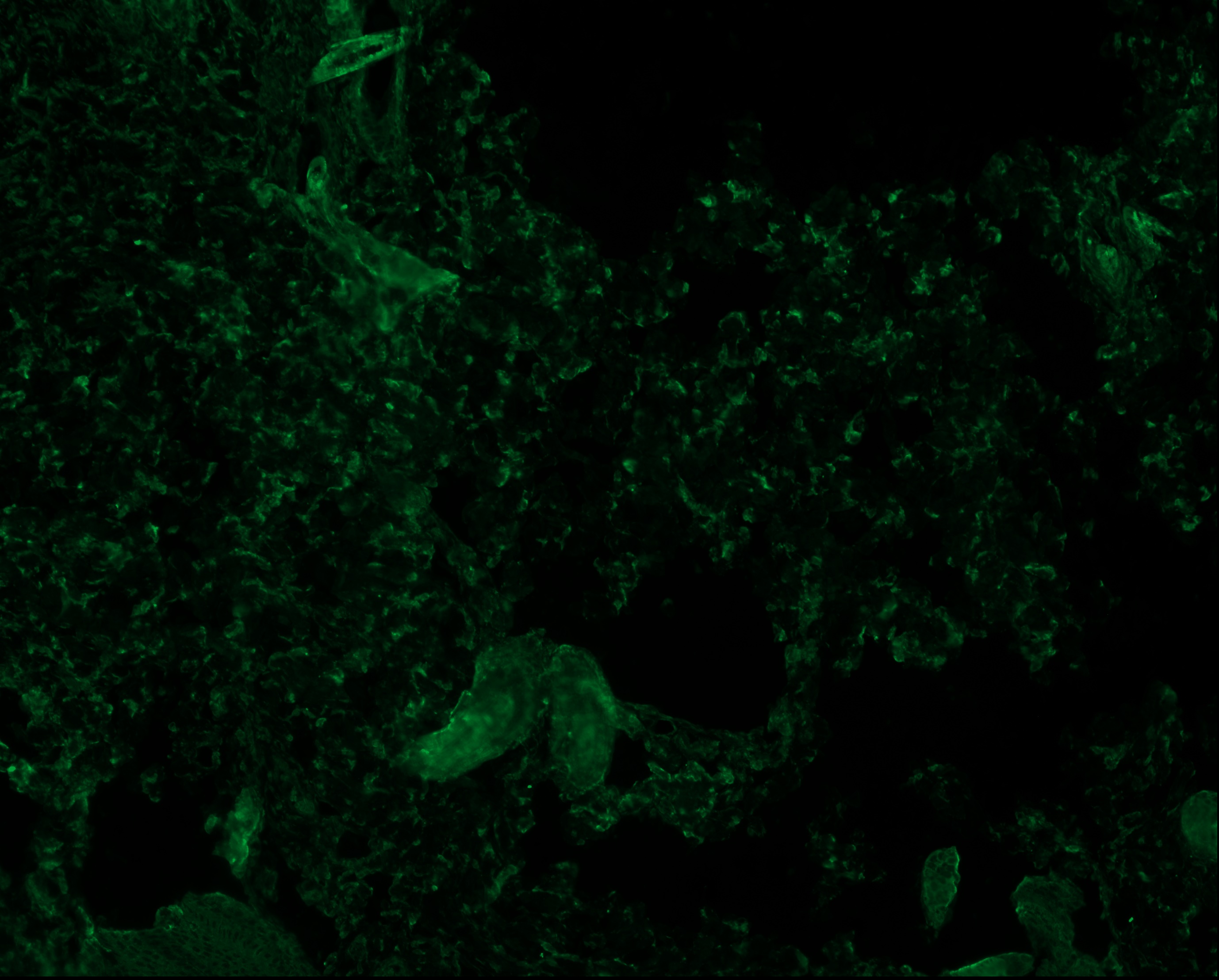

Supplement: Supplementary file 1 — Supplementary Information. [file 41598_2023_39765_MOESM1_ESM.zip › ╘¡╩╝╩2╛▌╒√└φ/tissue immunofluorescence/cd86ú║cd163/control (3)/Snap-4114_c3.jpg]

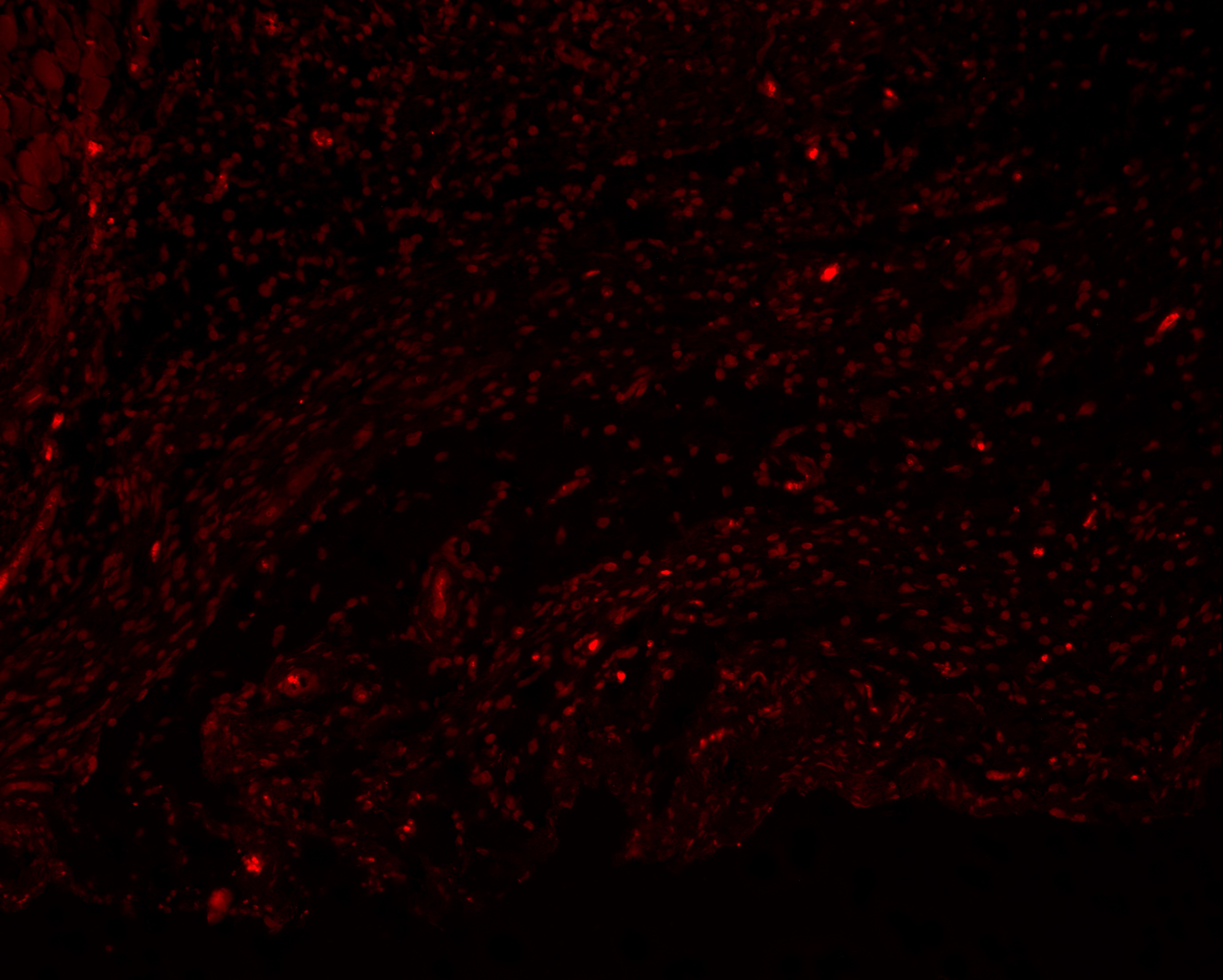

Supplement: Supplementary file 1 — Supplementary Information. [file 41598_2023_39765_MOESM1_ESM.zip › ╘¡╩╝╩2╛▌╒√└φ/tissue immunofluorescence/cd86ú║cd163/control/Snap-4106/Snap-4106_c1.jpg]

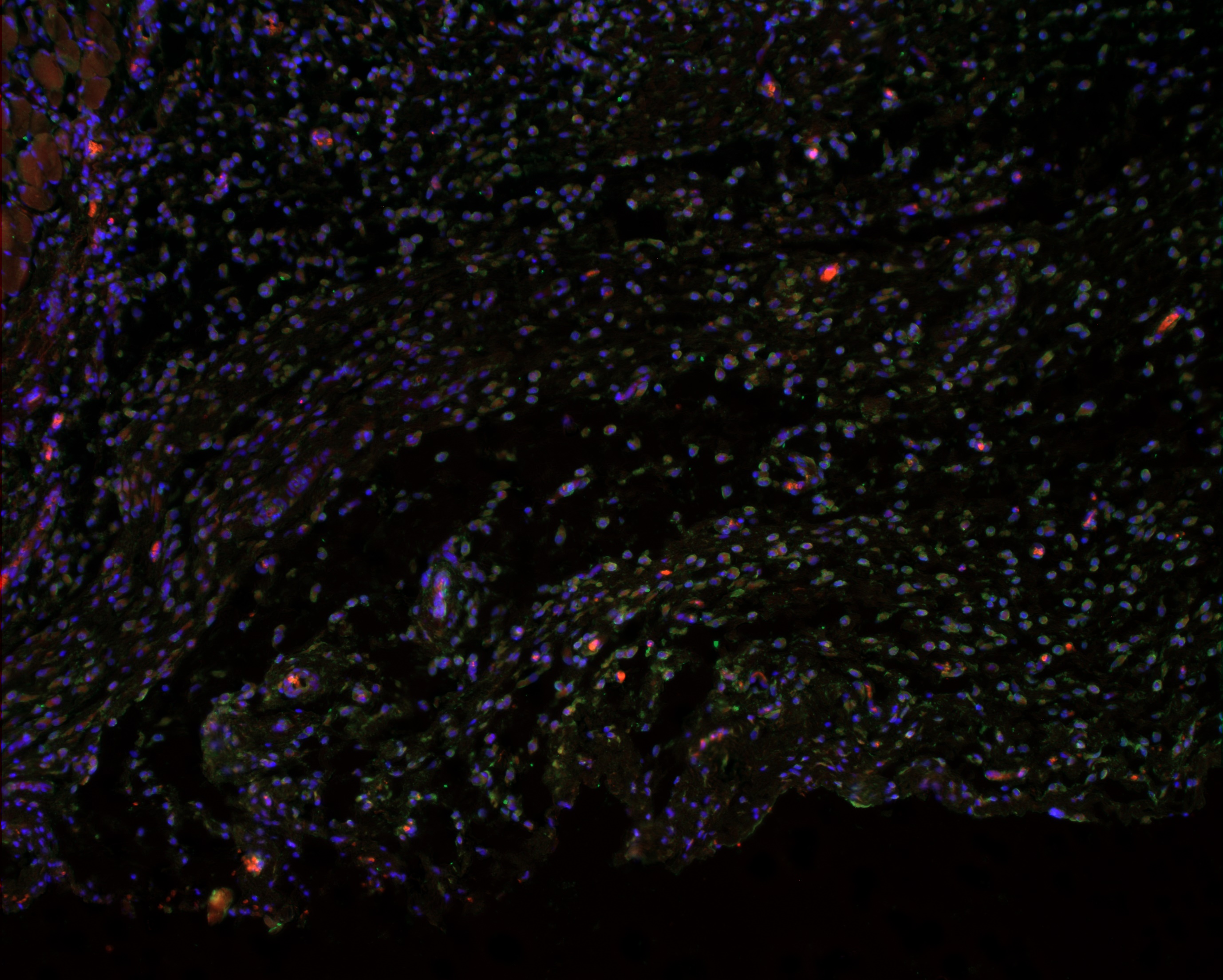

Supplement: Supplementary file 1 — Supplementary Information. [file 41598_2023_39765_MOESM1_ESM.zip › ╘¡╩╝╩2╛▌╒√└φ/tissue immunofluorescence/cd86ú║cd163/control/Snap-4106/Snap-4106_c1+2+3.jpg]

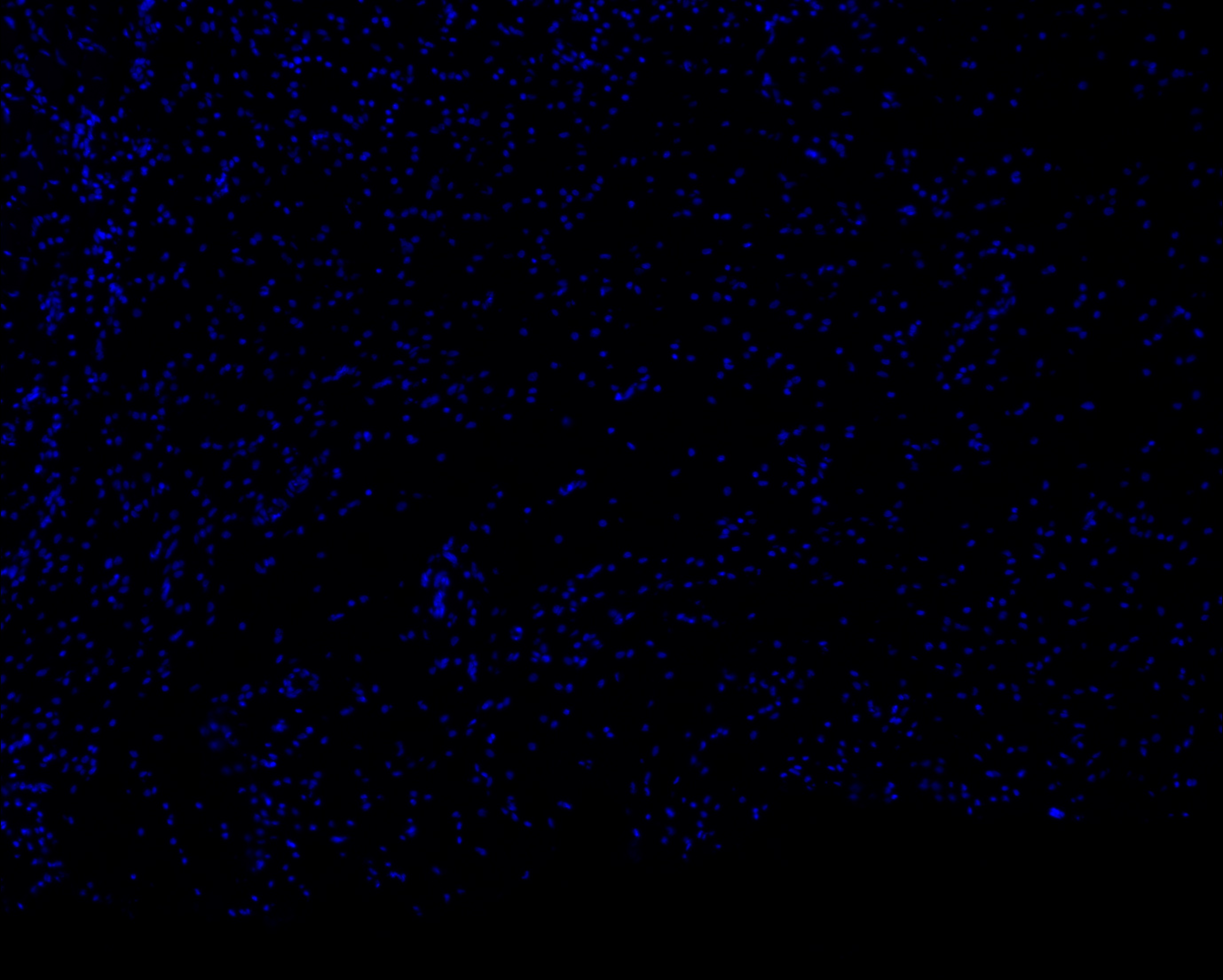

Supplement: Supplementary file 1 — Supplementary Information. [file 41598_2023_39765_MOESM1_ESM.zip › ╘¡╩╝╩2╛▌╒√└φ/tissue immunofluorescence/cd86ú║cd163/control/Snap-4106/Snap-4106_c2.jpg]

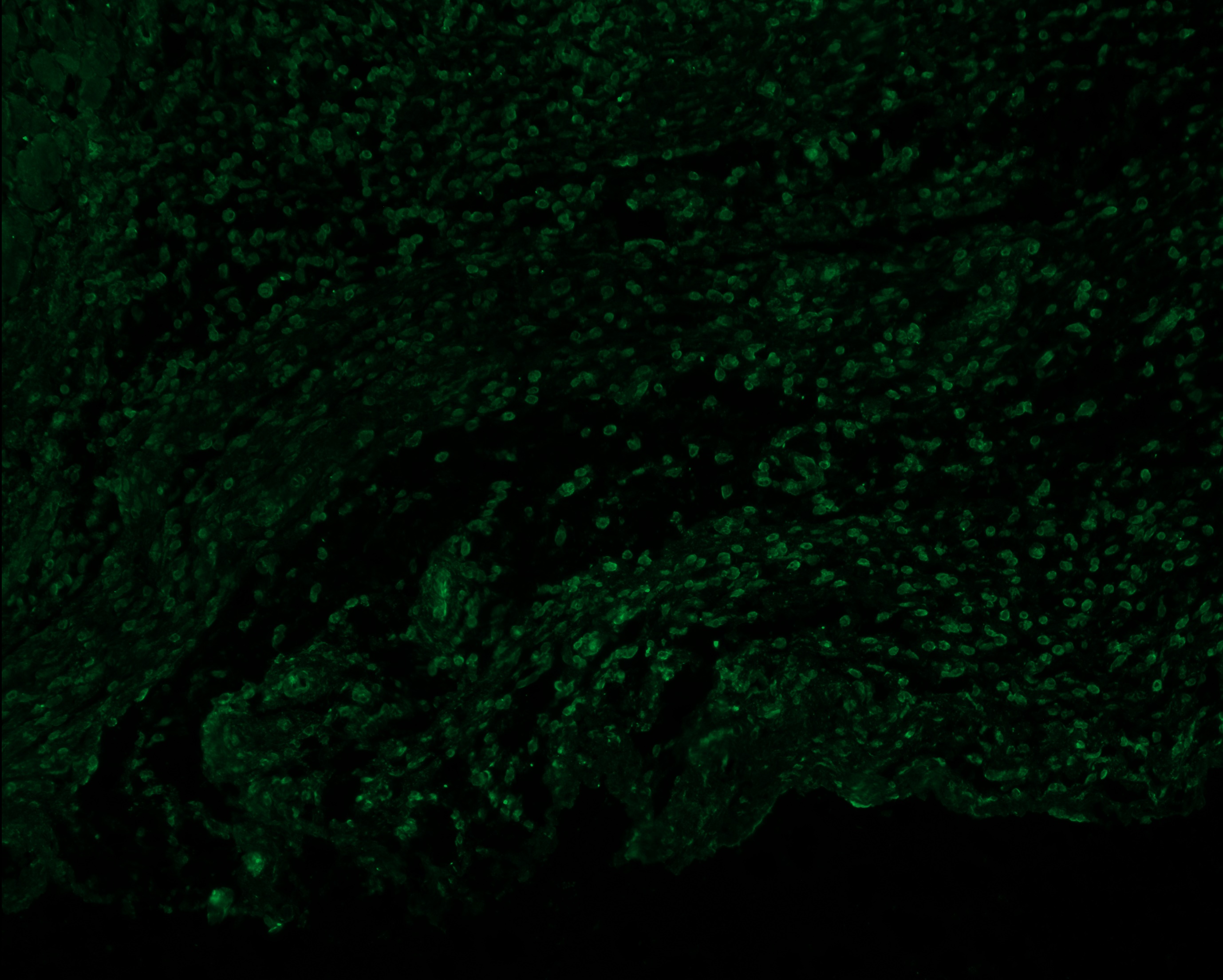

Supplement: Supplementary file 1 — Supplementary Information. [file 41598_2023_39765_MOESM1_ESM.zip › ╘¡╩╝╩2╛▌╒√└φ/tissue immunofluorescence/cd86ú║cd163/control/Snap-4106/Snap-4106_c3.jpg]

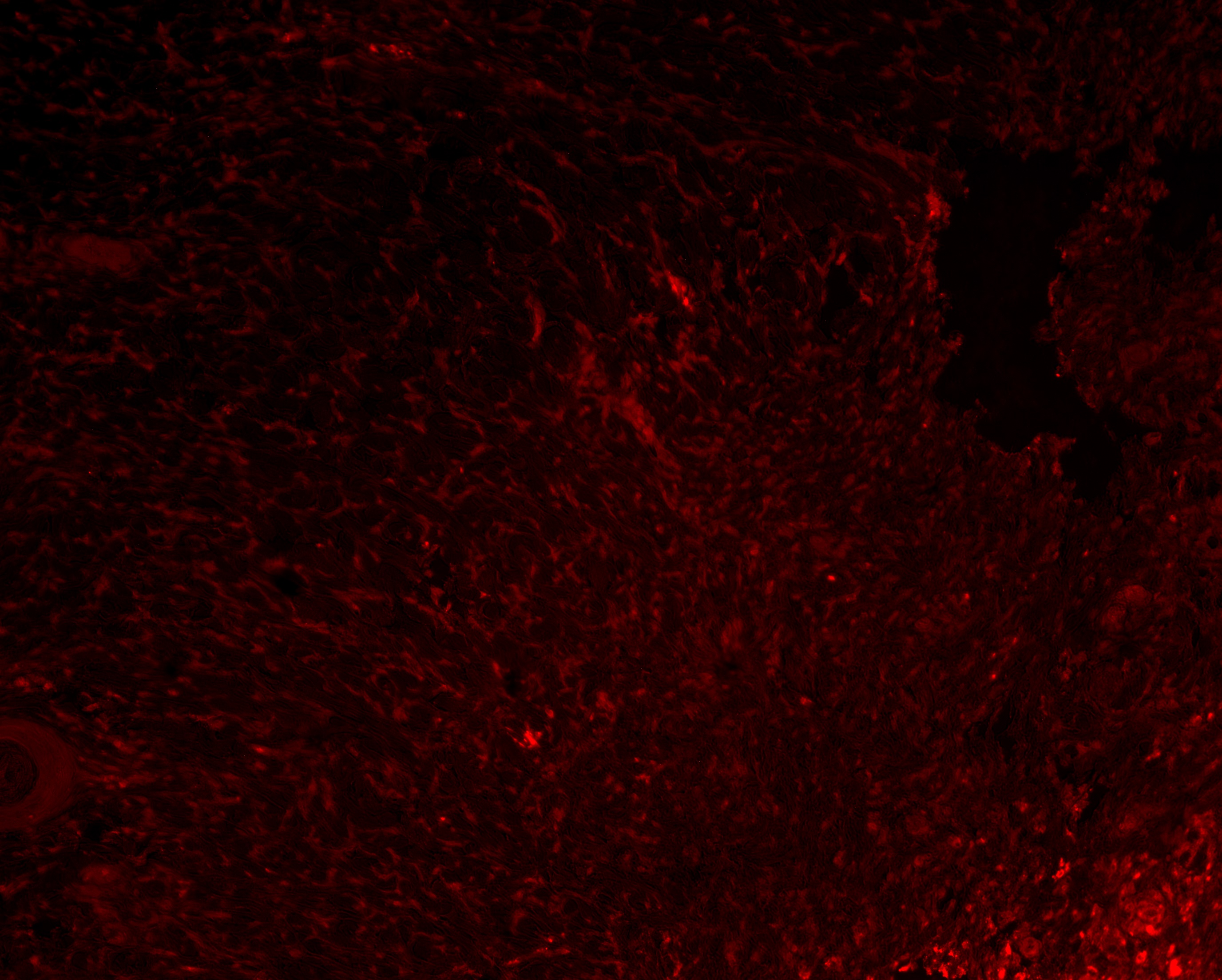

Supplement: Supplementary file 1 — Supplementary Information. [file 41598_2023_39765_MOESM1_ESM.zip › ╘¡╩╝╩2╛▌╒√└φ/tissue immunofluorescence/cd86ú║cd163/keratinase (2)/Snap-4154_c1.jpg]

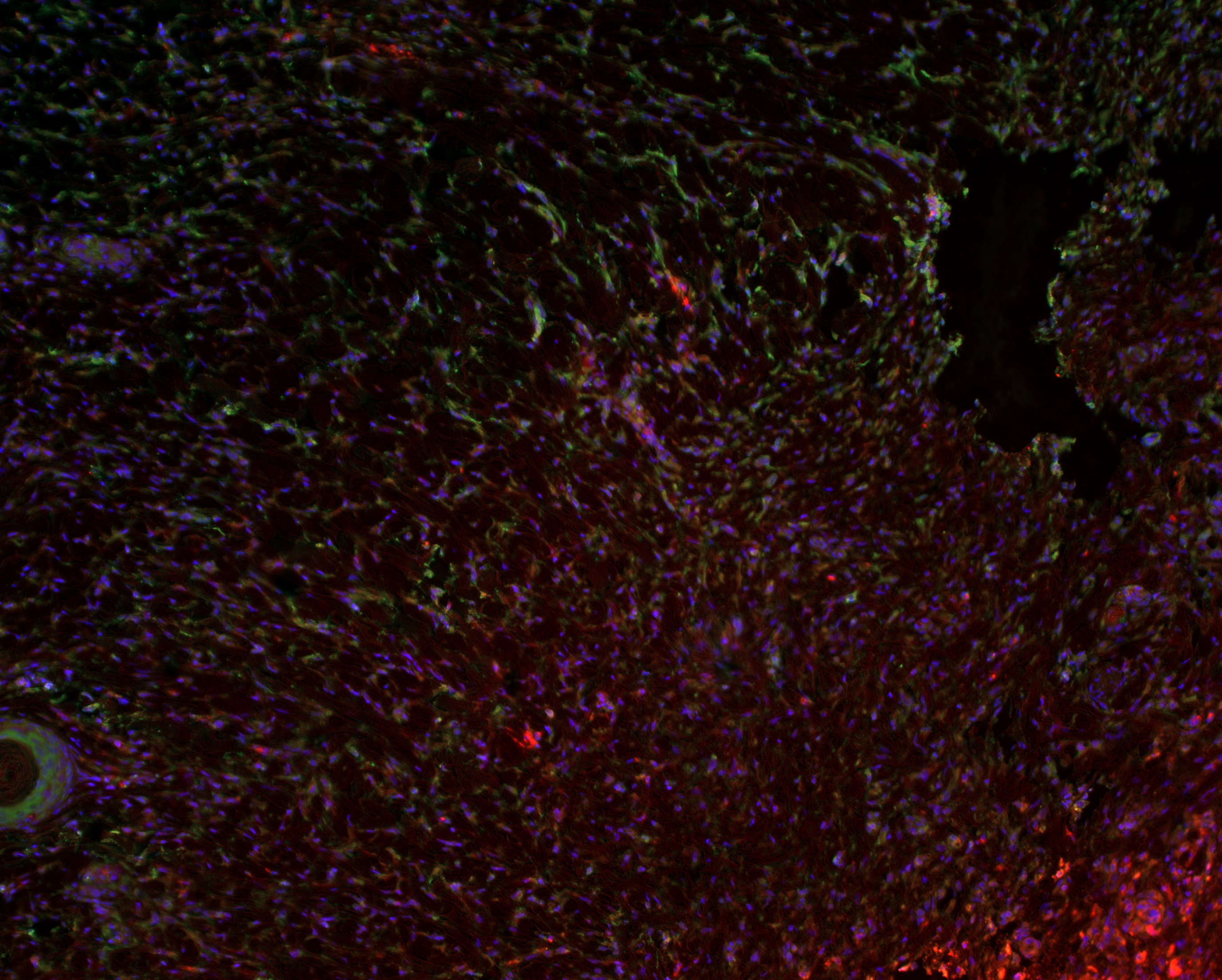

Supplement: Supplementary file 1 — Supplementary Information. [file 41598_2023_39765_MOESM1_ESM.zip › ╘¡╩╝╩2╛▌╒√└φ/tissue immunofluorescence/cd86ú║cd163/keratinase (2)/Snap-4154_c1+2+3.jpg]

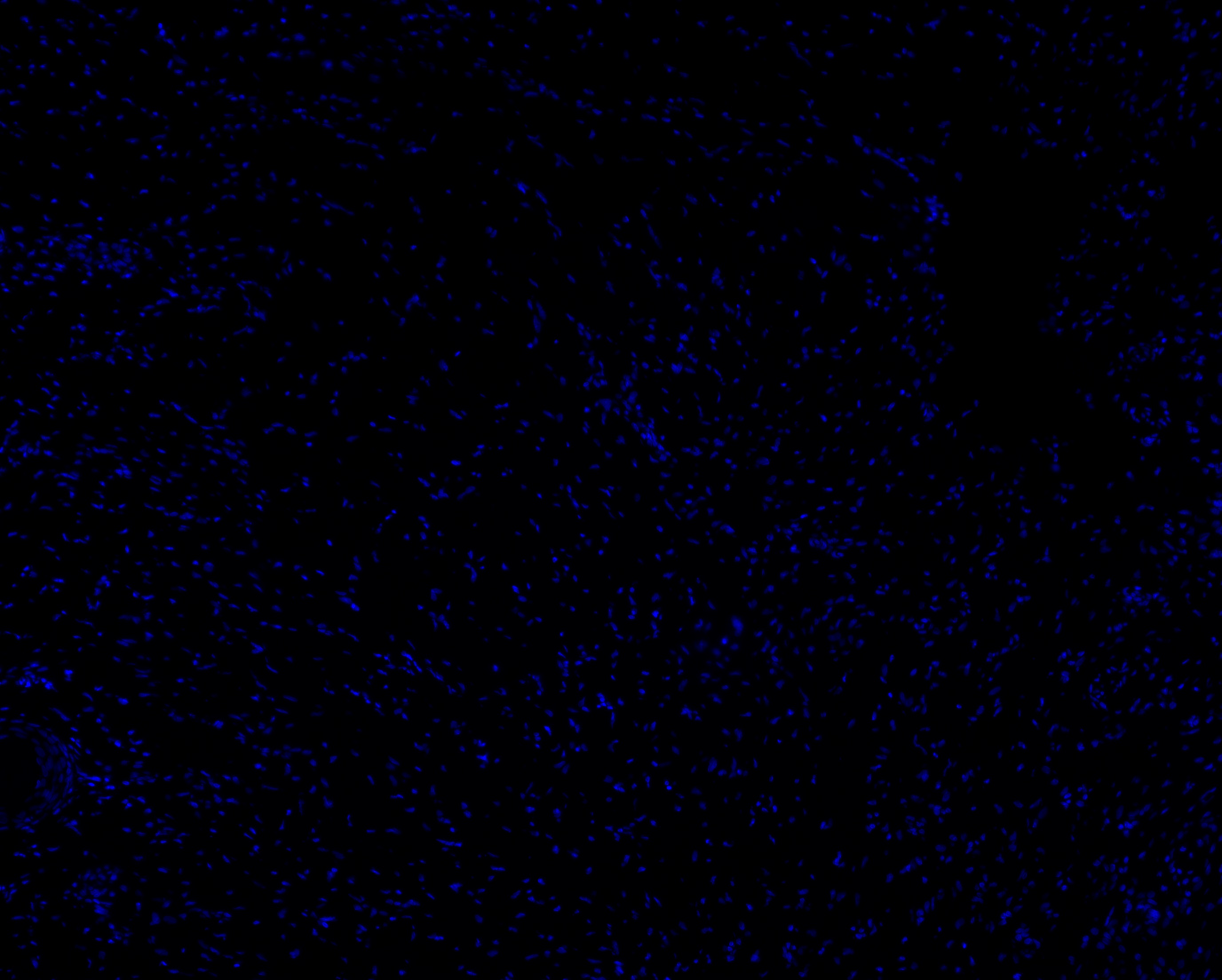

Supplement: Supplementary file 1 — Supplementary Information. [file 41598_2023_39765_MOESM1_ESM.zip › ╘¡╩╝╩2╛▌╒√└φ/tissue immunofluorescence/cd86ú║cd163/keratinase (2)/Snap-4154_c2.jpg]

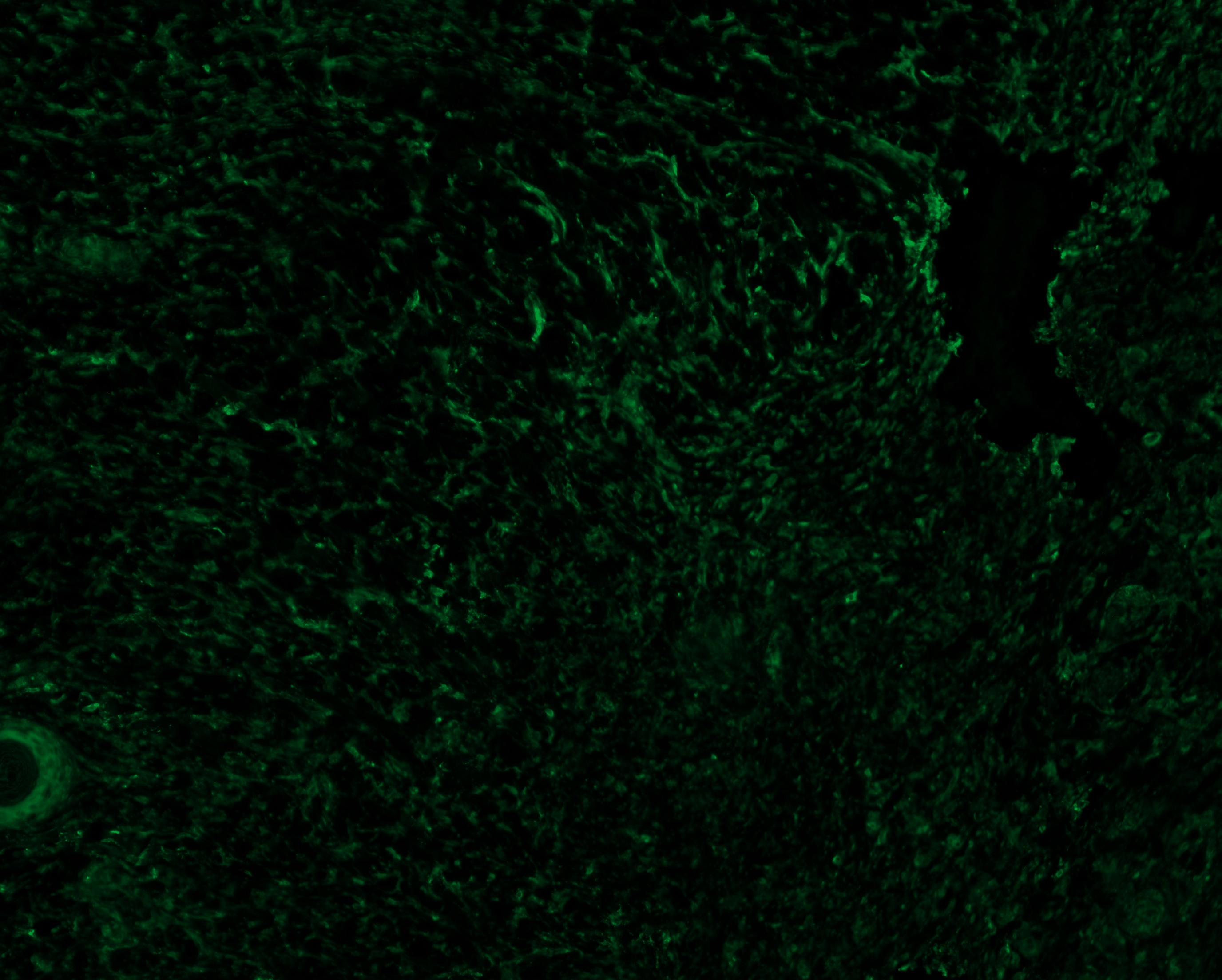

Supplement: Supplementary file 1 — Supplementary Information. [file 41598_2023_39765_MOESM1_ESM.zip › ╘¡╩╝╩2╛▌╒√└φ/tissue immunofluorescence/cd86ú║cd163/keratinase (2)/Snap-4154_c3.jpg]

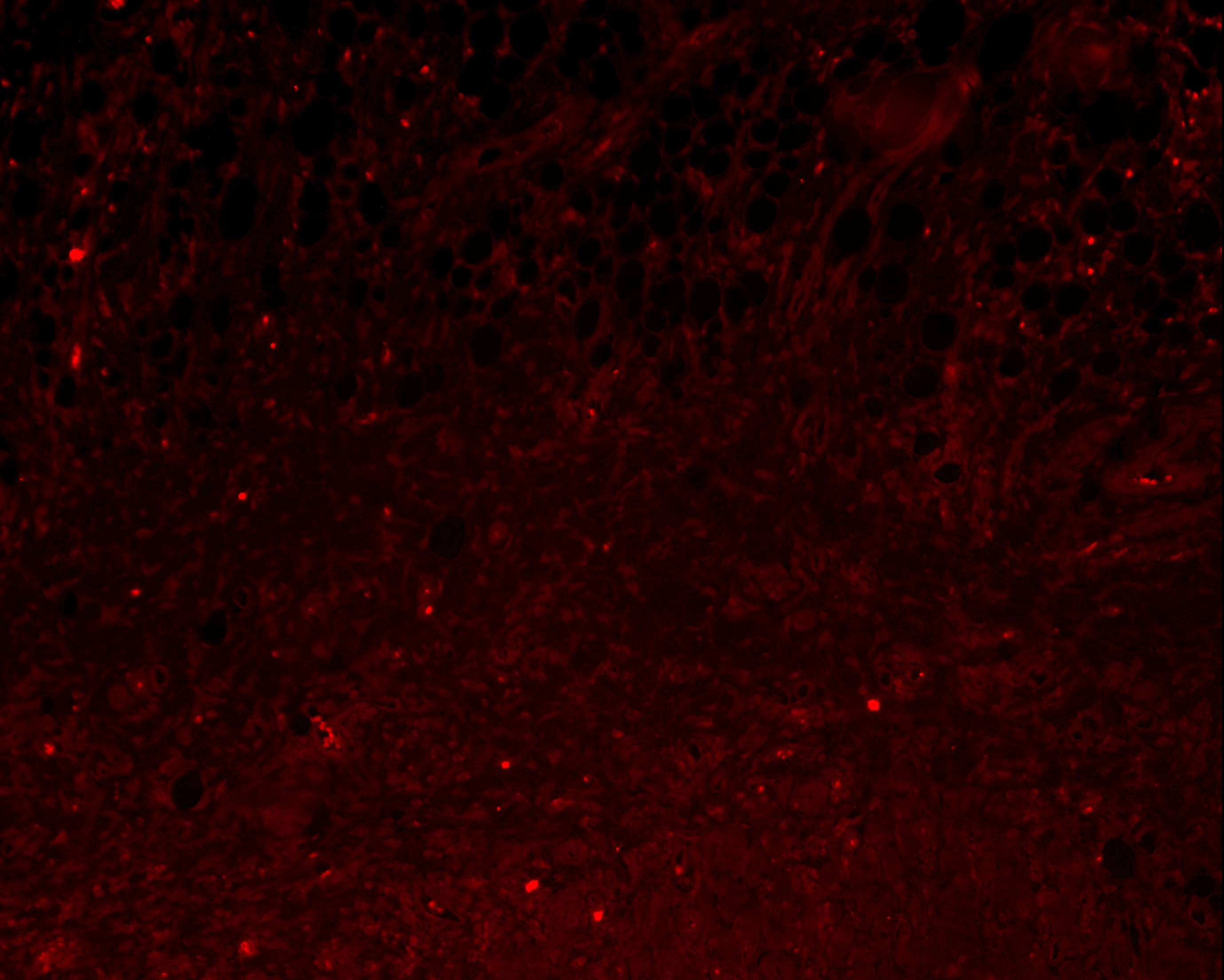

Supplement: Supplementary file 1 — Supplementary Information. [file 41598_2023_39765_MOESM1_ESM.zip › ╘¡╩╝╩2╛▌╒√└φ/tissue immunofluorescence/cd86ú║cd163/keratinase (3)/Snap-4148_c1.jpg]

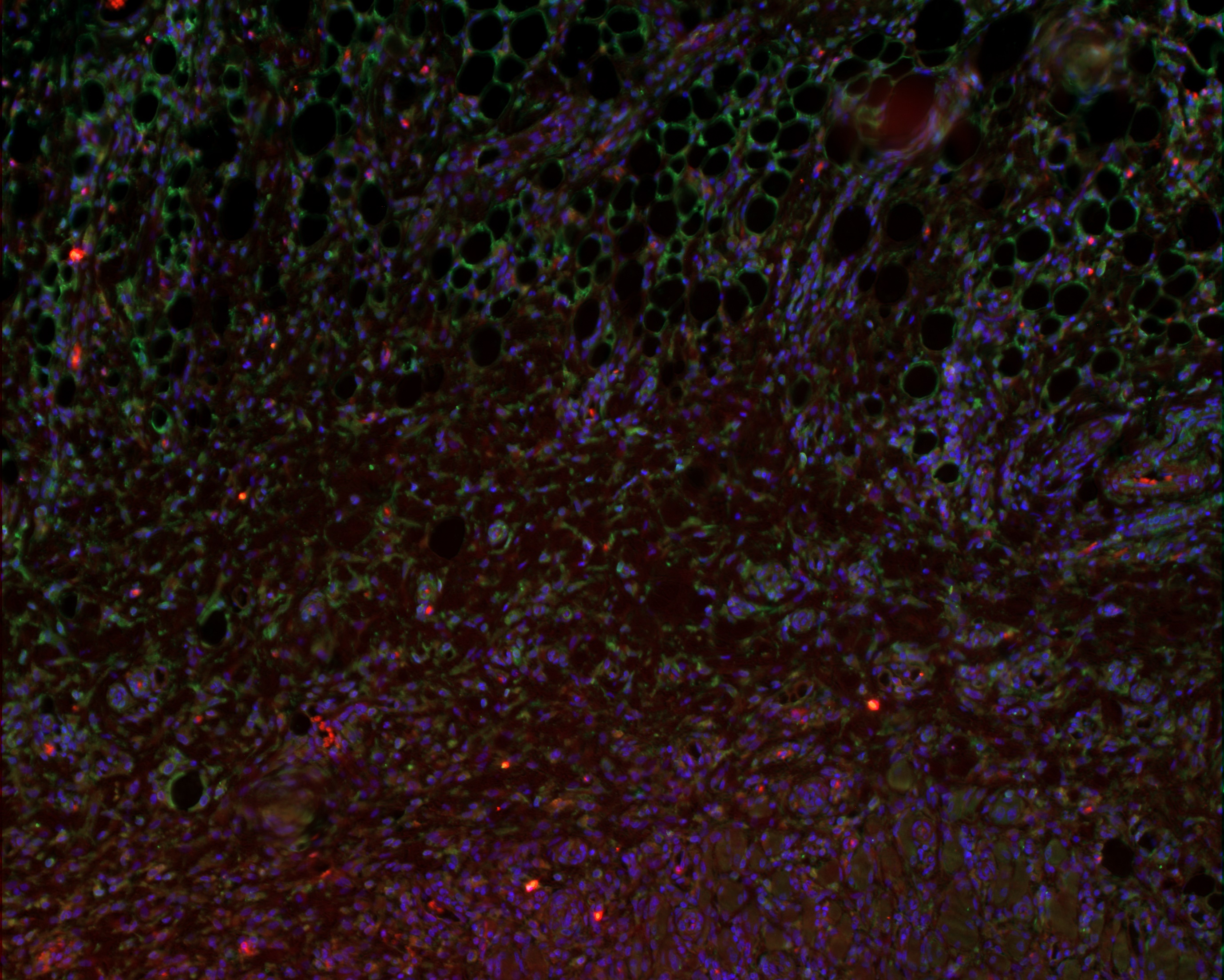

Supplement: Supplementary file 1 — Supplementary Information. [file 41598_2023_39765_MOESM1_ESM.zip › ╘¡╩╝╩2╛▌╒√└φ/tissue immunofluorescence/cd86ú║cd163/keratinase (3)/Snap-4148_c1+2+3.jpg]

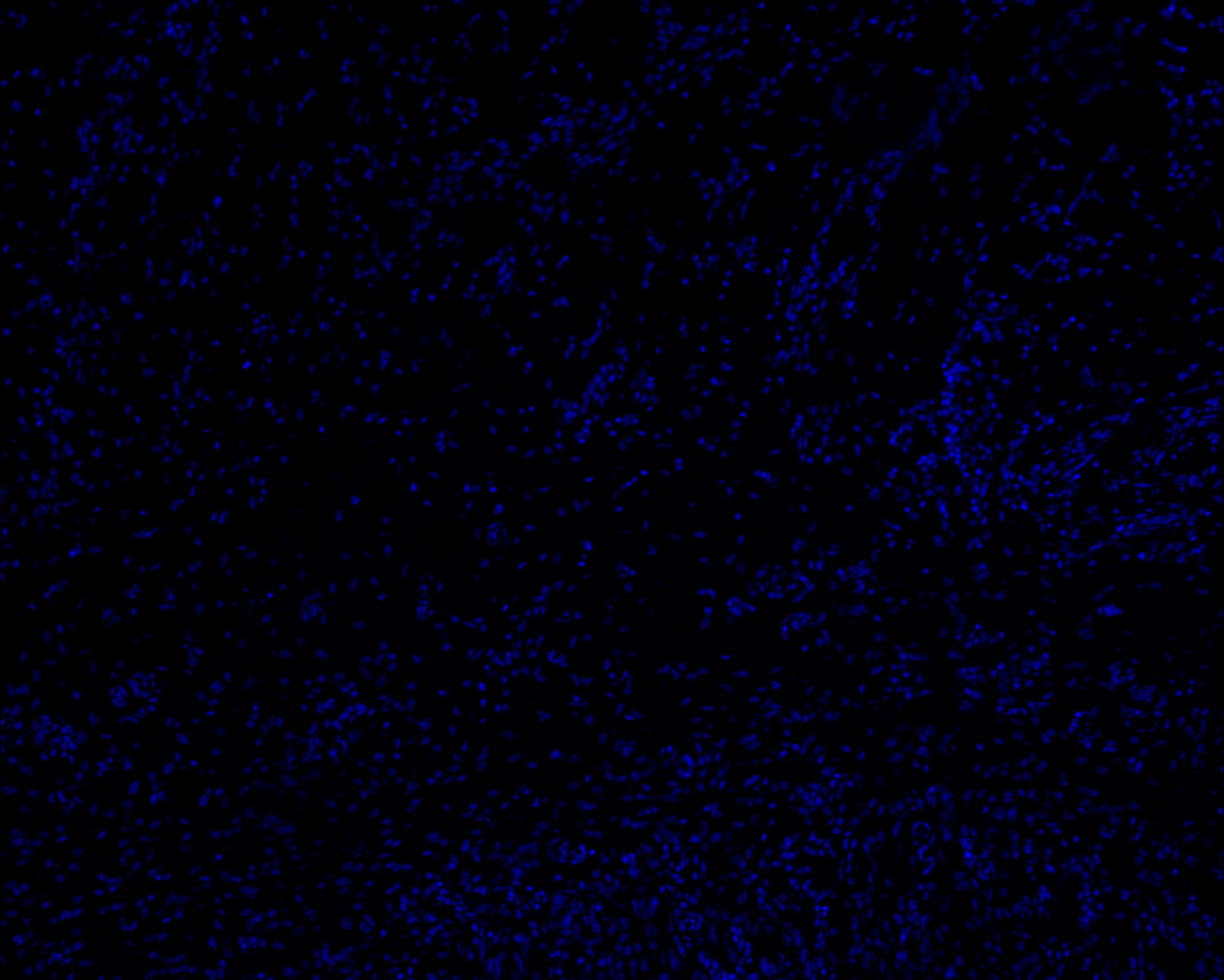

Supplement: Supplementary file 1 — Supplementary Information. [file 41598_2023_39765_MOESM1_ESM.zip › ╘¡╩╝╩2╛▌╒√└φ/tissue immunofluorescence/cd86ú║cd163/keratinase (3)/Snap-4148_c2.jpg]

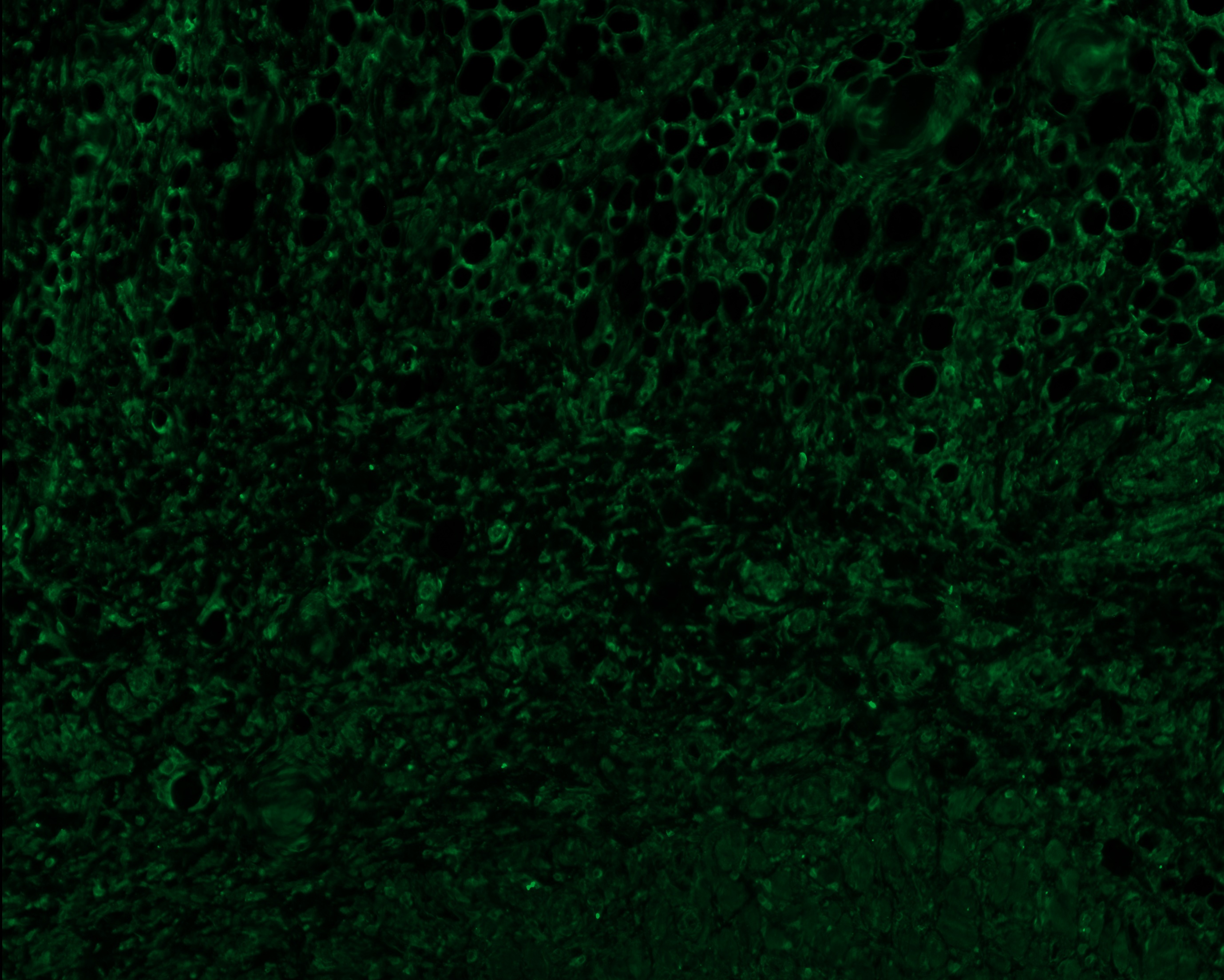

Supplement: Supplementary file 1 — Supplementary Information. [file 41598_2023_39765_MOESM1_ESM.zip › ╘¡╩╝╩2╛▌╒√└φ/tissue immunofluorescence/cd86ú║cd163/keratinase (3)/Snap-4148_c3.jpg]

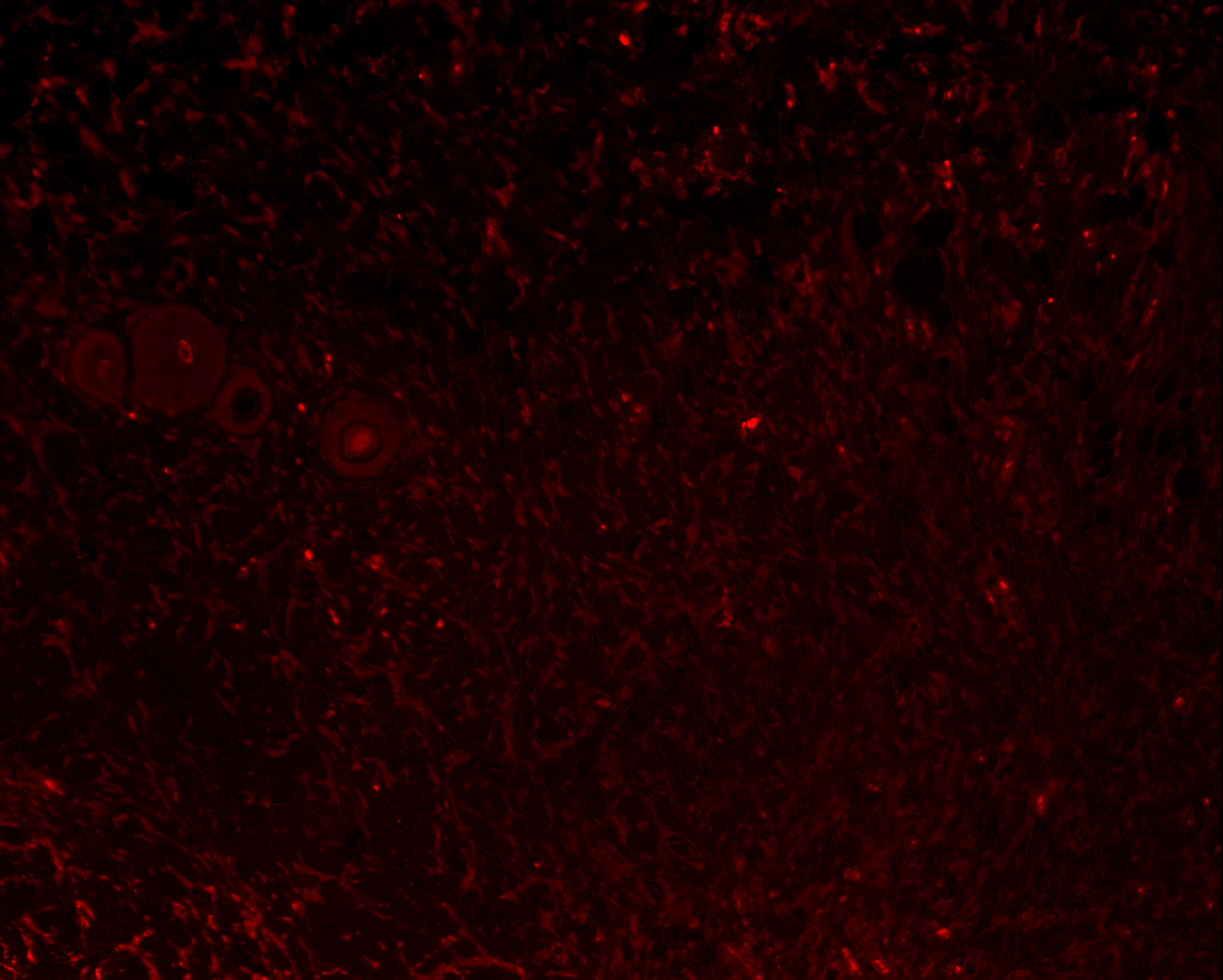

Supplement: Supplementary file 1 — Supplementary Information. [file 41598_2023_39765_MOESM1_ESM.zip › ╘¡╩╝╩2╛▌╒√└φ/tissue immunofluorescence/cd86ú║cd163/keratinase/Snap-4152_c1.jpg]

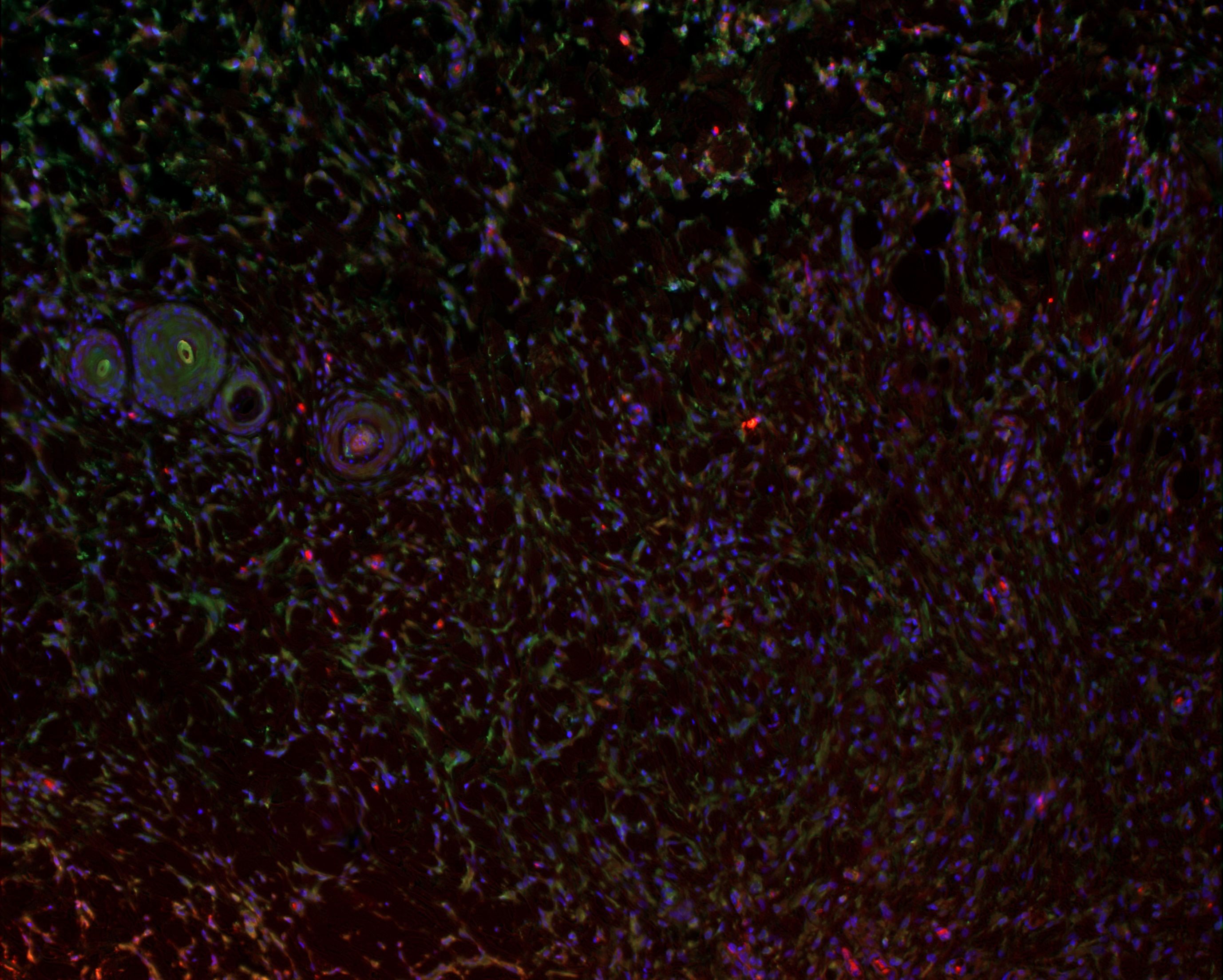

Supplement: Supplementary file 1 — Supplementary Information. [file 41598_2023_39765_MOESM1_ESM.zip › ╘¡╩╝╩2╛▌╒√└φ/tissue immunofluorescence/cd86ú║cd163/keratinase/Snap-4152_c1+2+3.jpg]

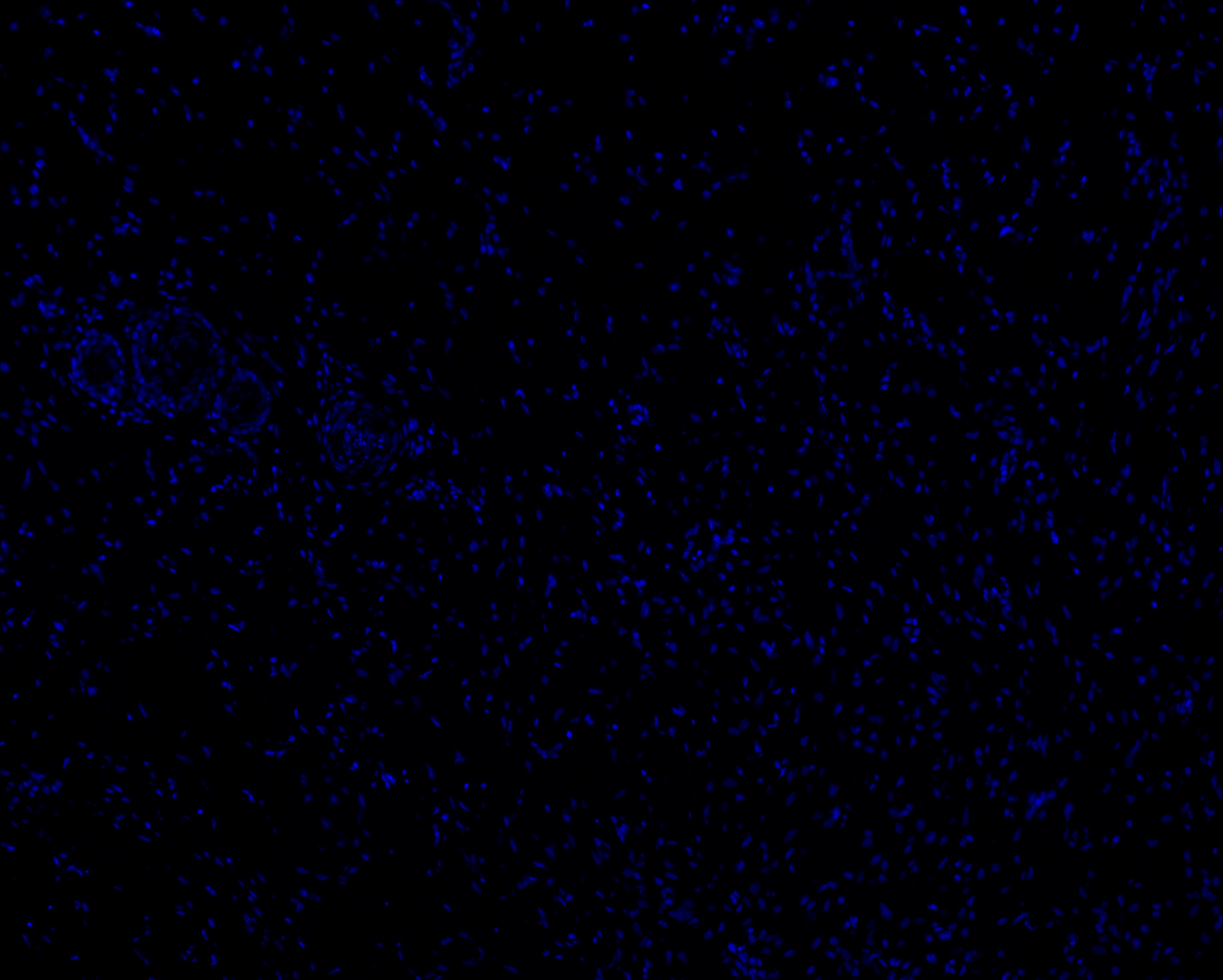

Supplement: Supplementary file 1 — Supplementary Information. [file 41598_2023_39765_MOESM1_ESM.zip › ╘¡╩╝╩2╛▌╒√└φ/tissue immunofluorescence/cd86ú║cd163/keratinase/Snap-4152_c2.jpg]

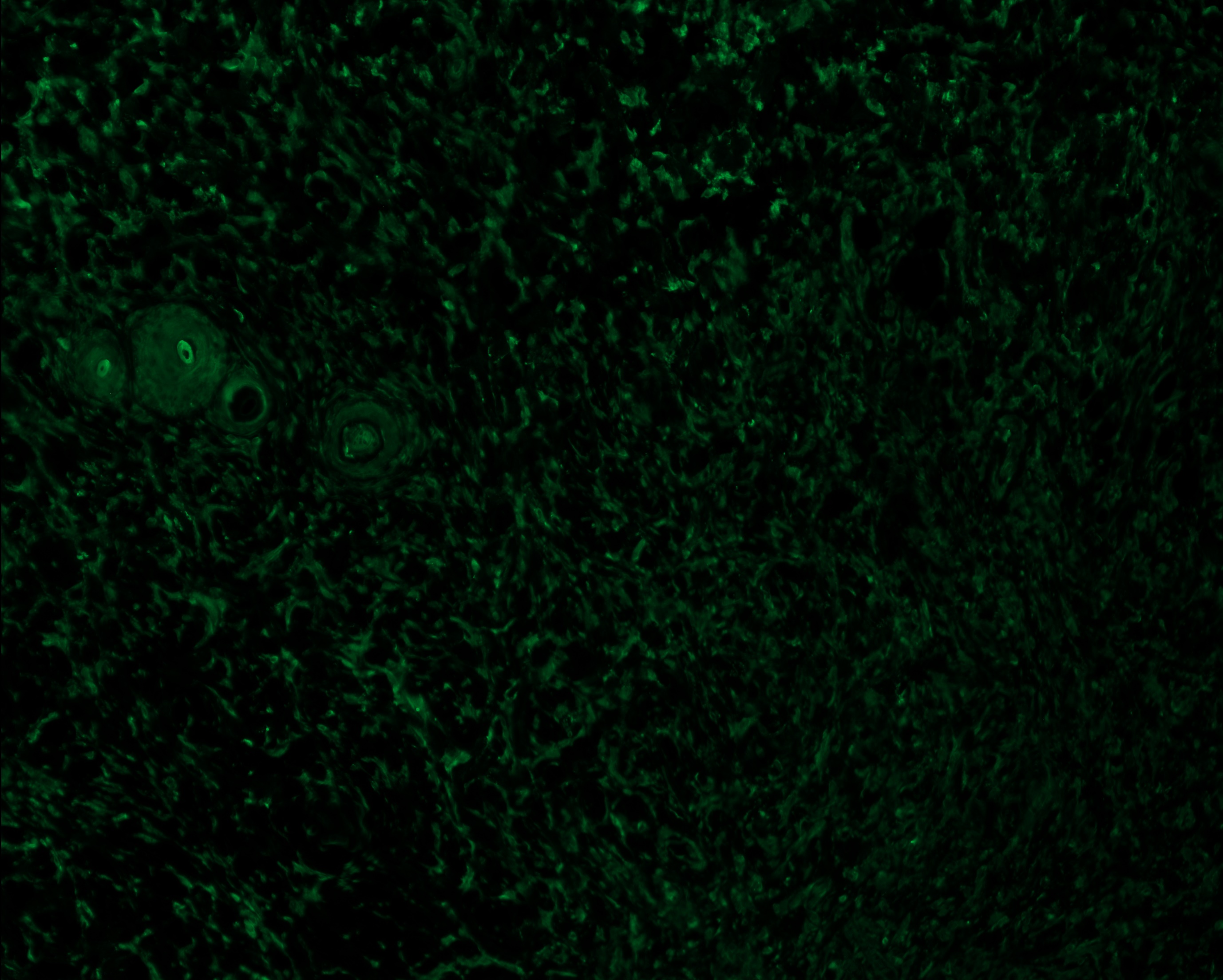

Supplement: Supplementary file 1 — Supplementary Information. [file 41598_2023_39765_MOESM1_ESM.zip › ╘¡╩╝╩2╛▌╒√└φ/tissue immunofluorescence/cd86ú║cd163/keratinase/Snap-4152_c3.jpg]

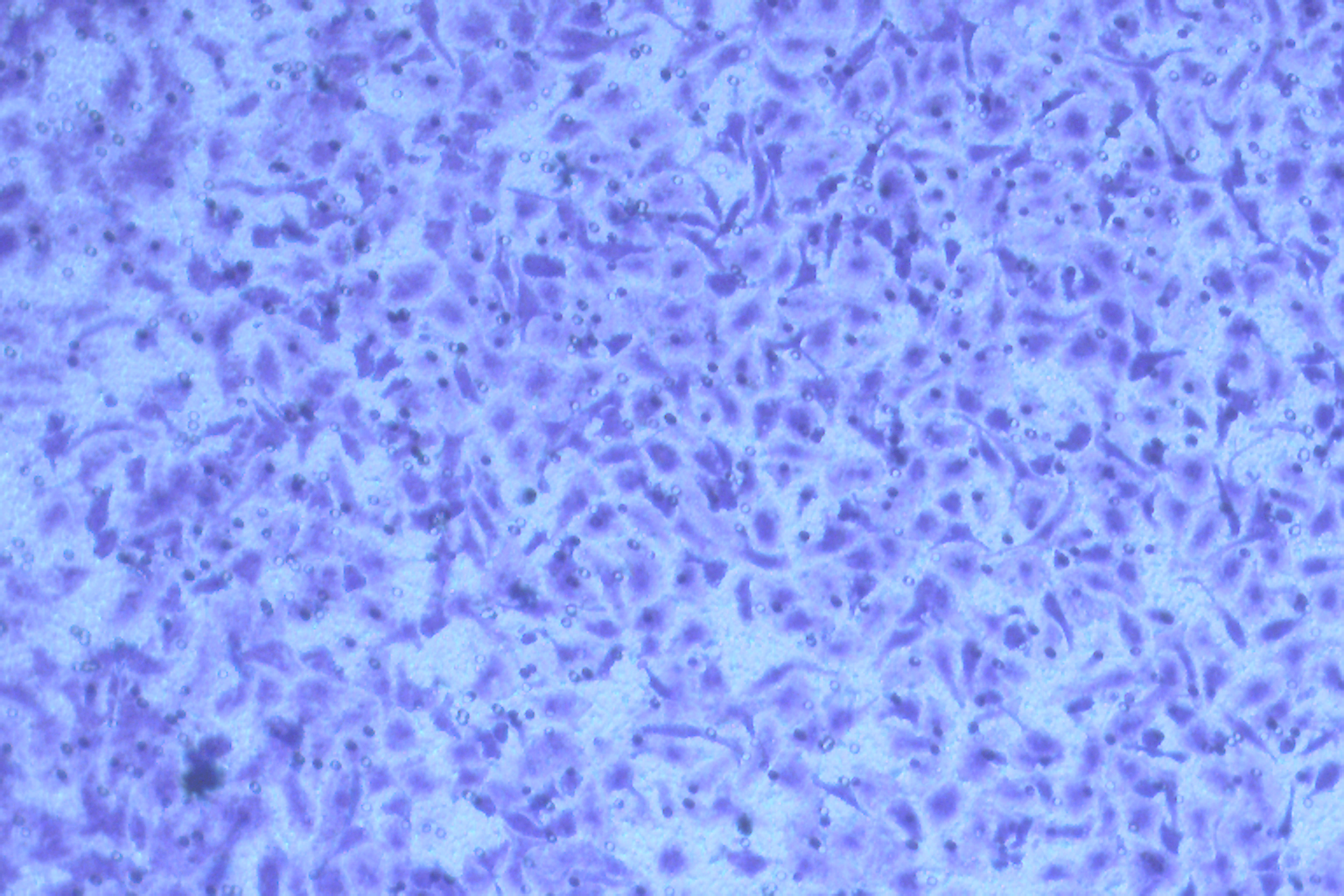

Supplement: Supplementary file 1 — Supplementary Information. [file 41598_2023_39765_MOESM1_ESM.zip › ╘¡╩╝╩2╛▌╒√└φ/transwell/Bromealin (1).bmp]

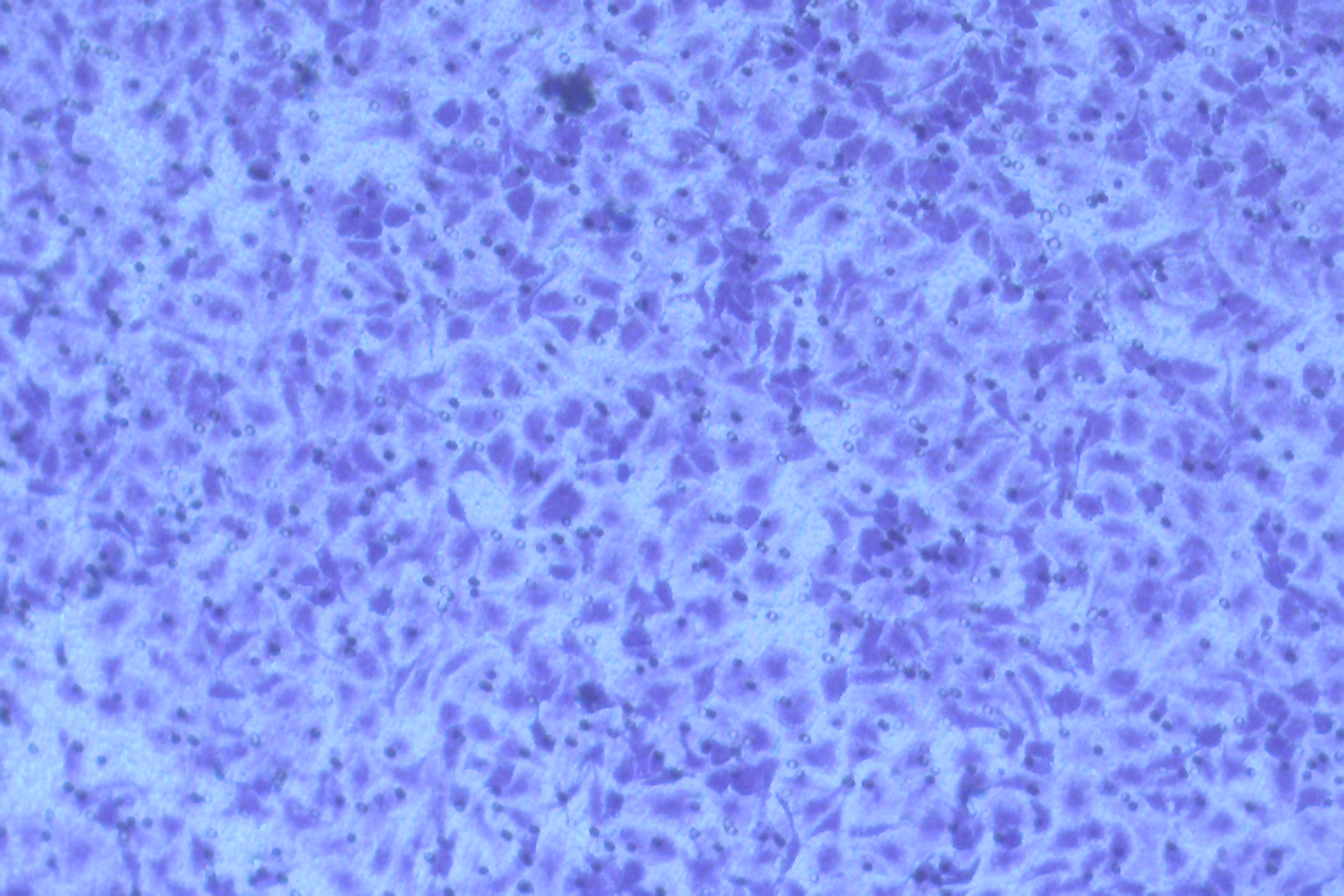

Supplement: Supplementary file 1 — Supplementary Information. [file 41598_2023_39765_MOESM1_ESM.zip › ╘¡╩╝╩2╛▌╒√└φ/transwell/Bromealin (2).bmp]

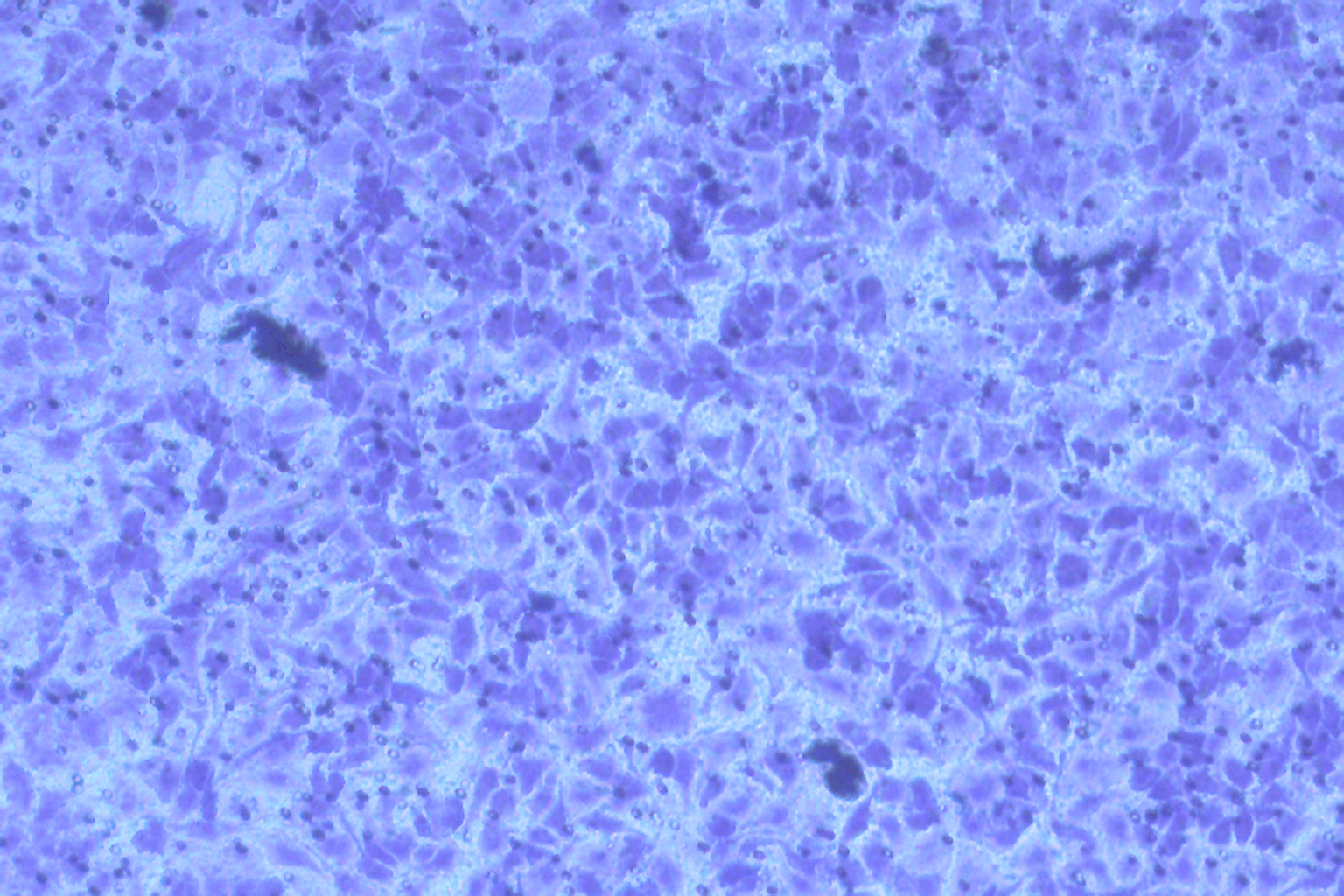

Supplement: Supplementary file 1 — Supplementary Information. [file 41598_2023_39765_MOESM1_ESM.zip › ╘¡╩╝╩2╛▌╒√└φ/transwell/Bromealin (3).bmp]

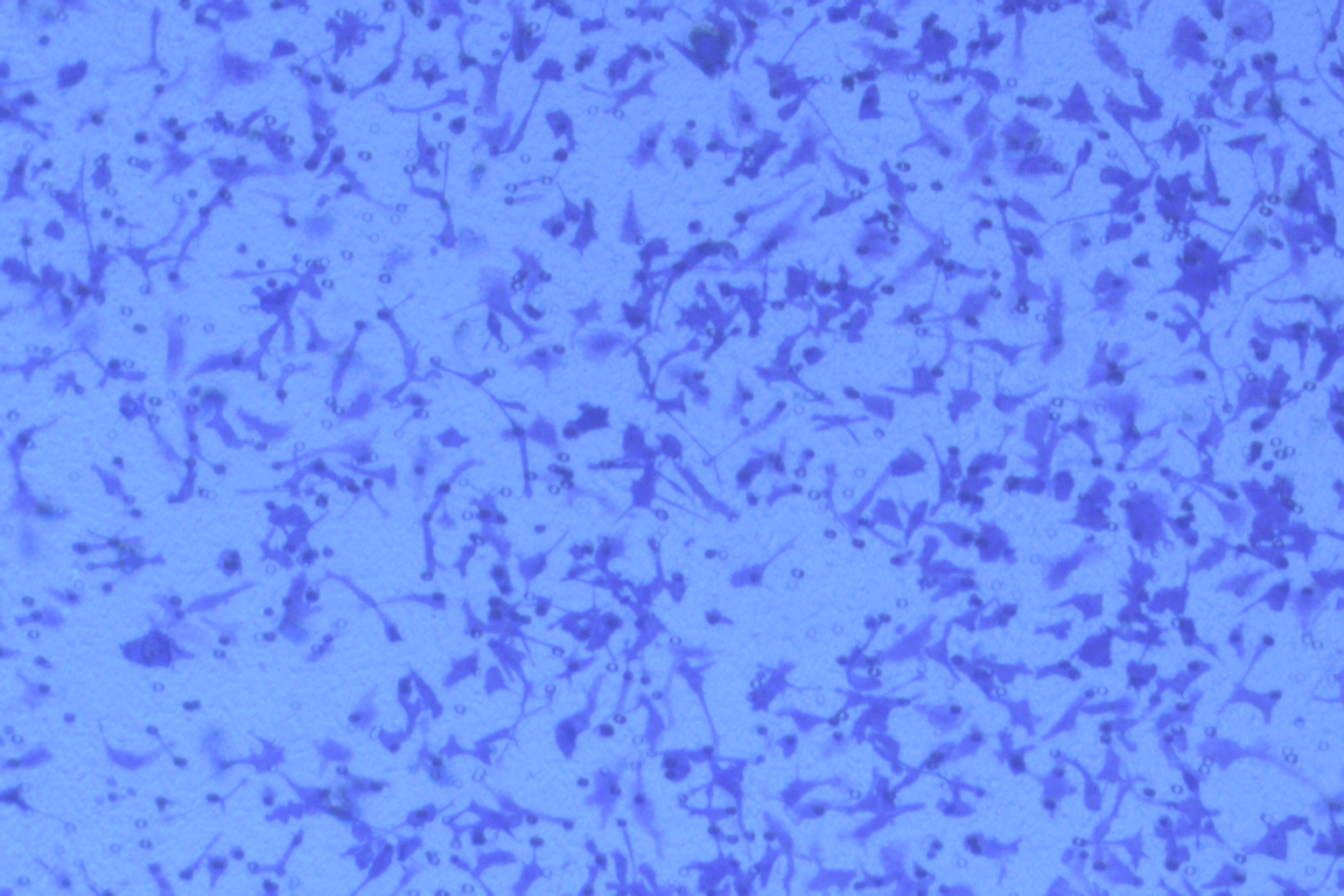

Supplement: Supplementary file 1 — Supplementary Information. [file 41598_2023_39765_MOESM1_ESM.zip › ╘¡╩╝╩2╛▌╒√└φ/transwell/Control (1).bmp]

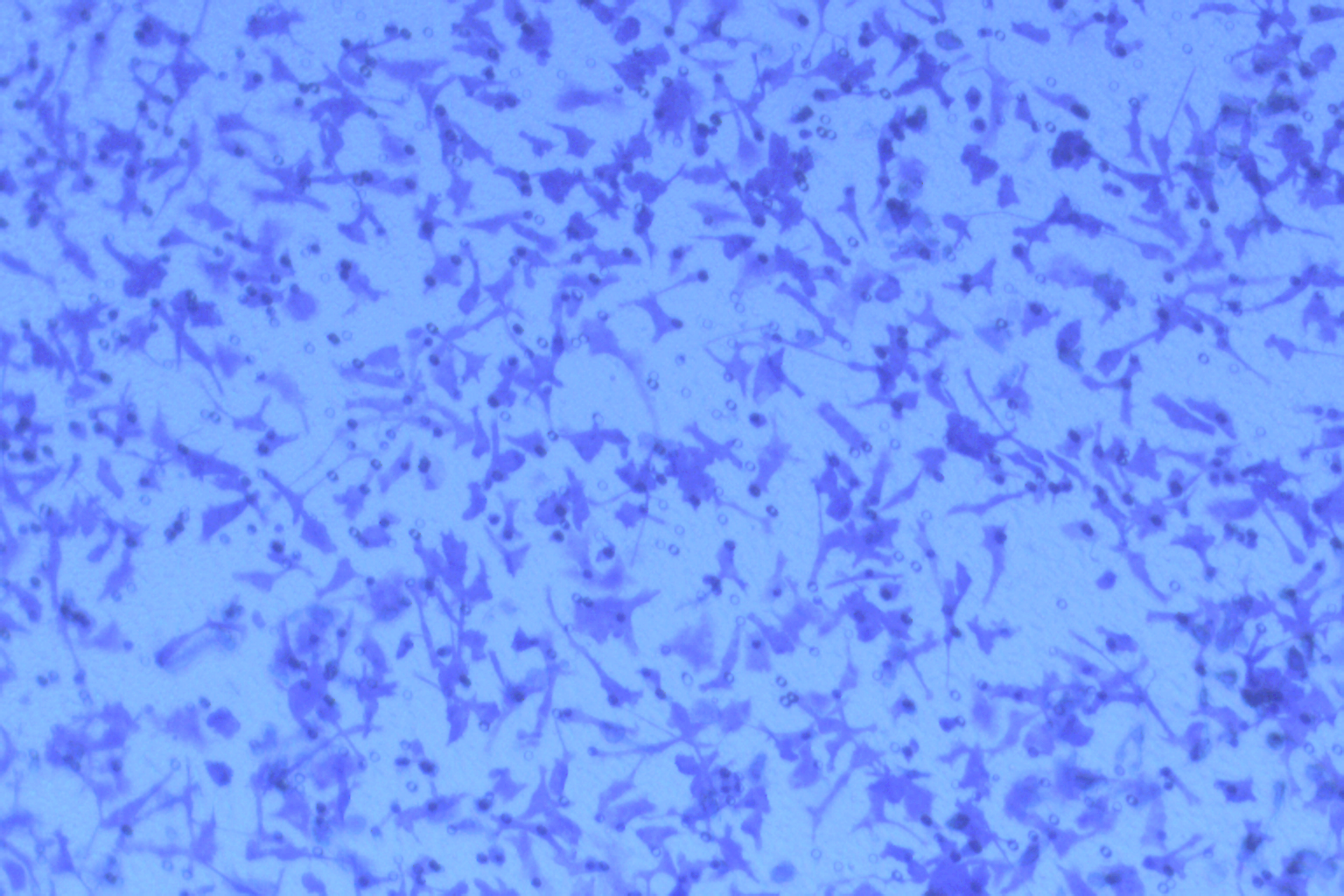

Supplement: Supplementary file 1 — Supplementary Information. [file 41598_2023_39765_MOESM1_ESM.zip › ╘¡╩╝╩2╛▌╒√└φ/transwell/Control (2).bmp]

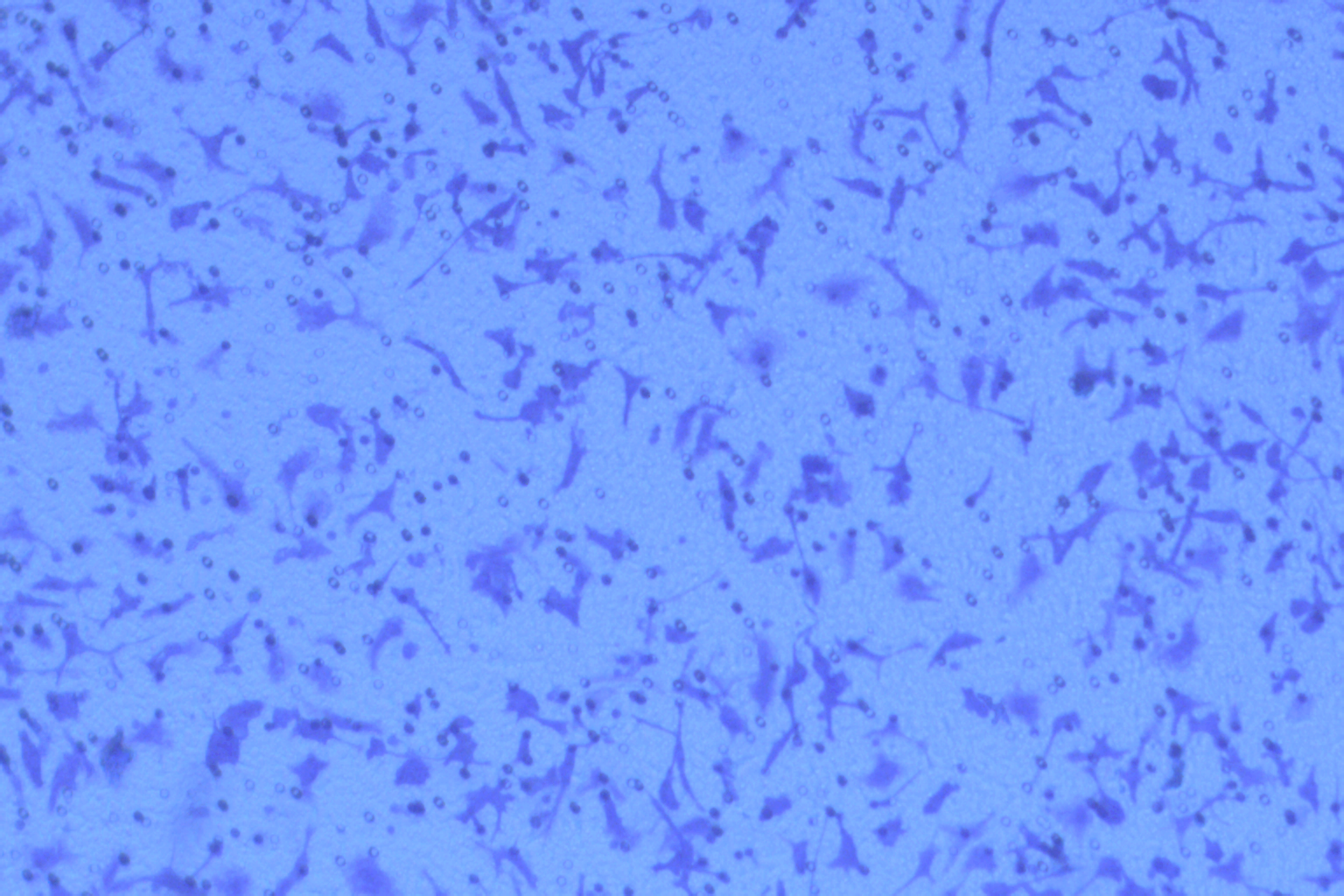

Supplement: Supplementary file 1 — Supplementary Information. [file 41598_2023_39765_MOESM1_ESM.zip › ╘¡╩╝╩2╛▌╒√└φ/transwell/Control (3).bmp]

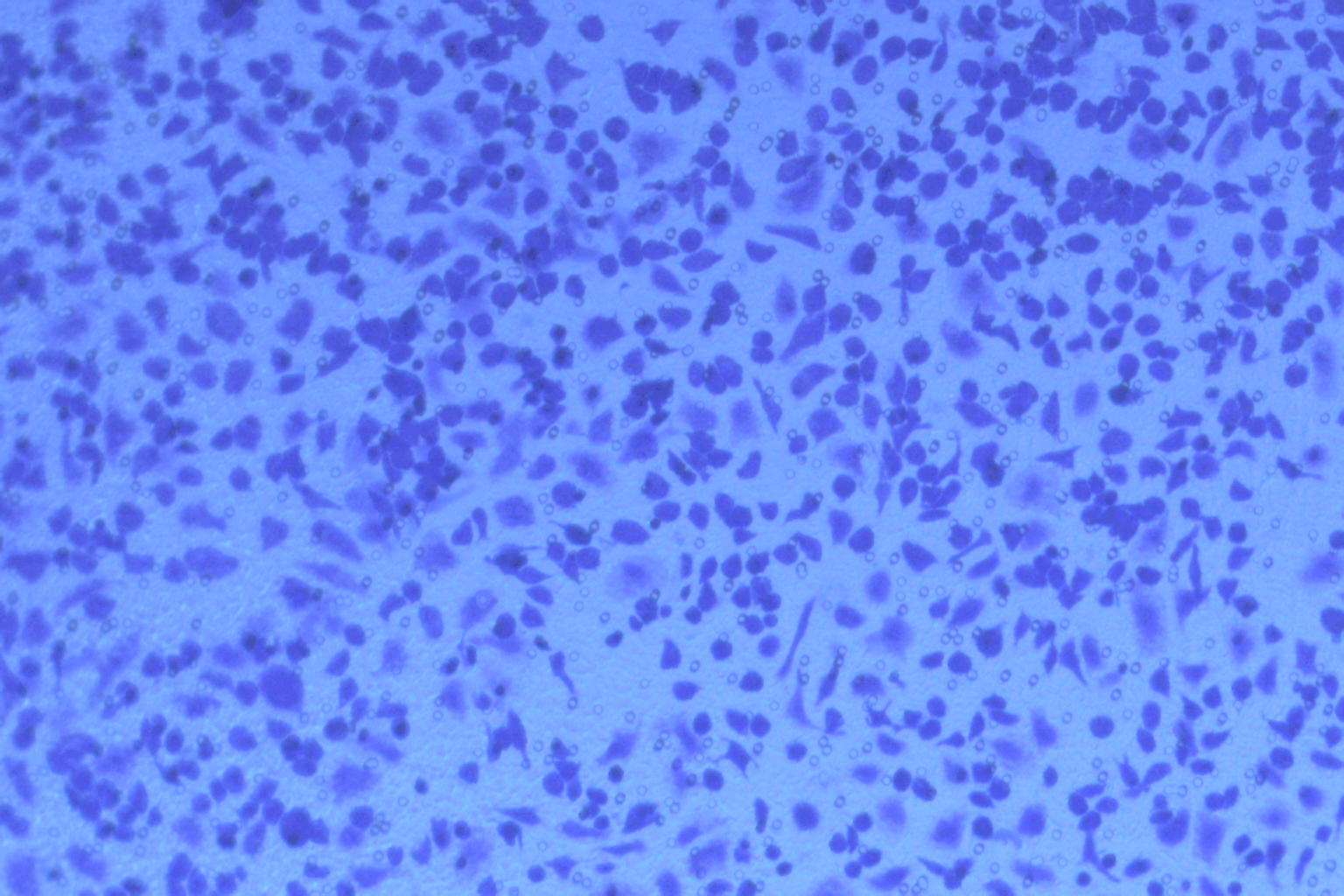

Supplement: Supplementary file 1 — Supplementary Information. [file 41598_2023_39765_MOESM1_ESM.zip › ╘¡╩╝╩2╛▌╒√└φ/transwell/keratinase (1).bmp]
